# Association of voltage-gated sodium channel mutations with field-evolved pyrethroid resistant phenotypes in soybean aphid and genetic markers for their detection

**DOI:** 10.1038/s41598-022-16366-1

**Published:** 2022-07-14

**Authors:** Ivair Valmorbida, Jessica D. Hohenstein, Brad S. Coates, Júlia G. Bevilaqua, James Menger, Erin W. Hodgson, Robert L. Koch, Matthew E. O’Neal

**Affiliations:** 1 Department of Entomology, Iowa State University, Ames, IA USA; 2 United States Department of Agriculture, Agricultural Research Service, Corn Insects and Crop Genetics Research Unit, Ames, IA USA; 3 Department of Crop Protection, Universidade Federal de Santa Maria, Santa Maria, RS Brazil; 4 Department of Entomology, University of Minnesota, Saint Paul, MN USA

**Keywords:** Entomology, PCR-based techniques

## Abstract

The frequent use of insecticides to manage soybean aphids, *Aphis glycines* (Hemiptera: Aphididae), in the United States has contributed to field-evolved resistance. Pyrethroid-resistant aphids have nonsynonymous mutations in the voltage-gated sodium channel (*vgsc*). We identified a leucine to phenylalanine mutation at position 1014 (L1014F) and a methionine to isoleucine mutation (M918I) of the *A. glycines vgsc,* both suspected of conferring knockdown resistance (*kdr*) to lambda-cyhalothrin. We developed molecular markers to identify these mutations in insecticide-resistant aphids. We determined that *A. glycines* which survived exposure to a diagnostic concentration of lambda-cyhalothrin and bifenthrin via glass-vial bioassays had these mutations, and showed significant changes in the resistance allele frequency between samples collected before and after field application of lambda-cyhalothrin. Thus, a strong association was revealed between aphids with L1014F and M918I *vgsc* mutations and survival following exposure to pyrethroids. Specifically, the highest survival was observed for aphids with the *kdr* (L1014F) and heterozygote *super-kdr* (L1014F + M918I) genotypes following laboratory bioassays and in-field application of lambda-cyhalothrin. These genetic markers could be used as a diagnostic tool for detecting insecticide-resistant *A. glycines* and monitoring the geographic distribution of pyrethroid resistance. We discuss how generating these types of data could improve our efforts to mitigate the effects of pyrethroid resistance on crop production.

## Introduction

The prevalence of insecticide resistance among arthropods continues to increase globally^1,2^, and can dramatically reduce the ability of farmers to control damage and manage the spread of insect-vectored diseases^3–5^. Additionally, increased production costs can be incurred when resistance evolves to less expensive active ingredients, necessitating a transition to more-expensive alternative chemistries^2,5^. Furthermore, higher insecticide application rates used to control more resistant insect populations have greater detrimental effects on the environment^6,7^. This scenario threatens the sustainability of crop production practices and global food security.

Pyrethroids function as neurotoxins through the strong binding and maintenance of an open state for the voltage-gated sodium channel (*vgsc*) protein^8^. Nucleotide mutations leading to non-synonymous changes to amino acids in or flanking the target site, alone or in combination, are associated with resistance to pyrethroids^9–12^. Knockdown resistance (*kdr*) genotypes with a leucine to phenylalanine amino acid substitution at positions orthologous to 1014 (L1014F) in the *vgsc* protein of house fly, *Musca domestica*^13^, are reported to confer low to moderate levels of pyrethroid resistance^12,14^. Additionally, this L1014F *kdr* mutation in combination with a methionine to threonine amino acid substitution at *vgsc* protein position 918 (M918T) is causal of the *super-kdr* phenotype in *M. domestica* that confers increased levels of resistance^15,16^.

Pyrethroid resistance in field populations of several aphid species including *Aphis glycines* is associated with non-synonymous mutations in the *vgsc*^17–23^. Despite these associations, pyrethroid resistance is reported to be multimodal^24^. In aphids, the up-regulation of detoxification genes can also contribute to field-evolved pyrethroid resistance^25–27^. Understanding the mechanism(s) by which resistance develops among pests can lead to increased capacity to evaluate the efficacy of insect resistance management (IRM) programs and track the spread of resistant phenotypes^28^, leading to improved locally appropriate control recommendations.

*Aphis glycines* was first observed in the United States in 2000 when populations were discovered on soybean, *Glycine max*, in Wisconsin^29^. By 2003, *A. glycines* was established throughout a 12-state region, including all 99 counties of Iowa. Foliar-applied insecticides are primarily used to prevent yield losses caused by *A. glycines* throughout the northcentral United States^30^. Failures of foliar-applied pyrethroids to control field outbreaks have been reported since 2015 across several northcentral states^31^. Subsequent laboratory bioassays confirmed a decreased susceptibility to pyrethroids (bifenthrin and lambda-cyhalothrin) among populations collected from soybean fields^31,32^.

The mechanism(s) of pyrethroid resistance in *A. glycines* is not fully understood, but the up-regulation of detoxification genes, cytochrome P450 monooxygenases and esterases, were detected among resistant populations in the United States^33^ and China^34^. In addition, *kdr* and *super-kdr* mutations were characterized in a field survey of 24 *A. glycines* collected in the northcentral United States^23^. We tested the hypothesis that *kdr* and *super-kdr* mutations have a role in conferring resistance to pyrethroids in *A. glycines* through the application of relatively high throughput molecular screening technique. We confirmed the presence of *kdr* and *super-kdr* genotypes, and observed a significant increase of *kdr* alleles in field populations following the application lambda-cyhalothrin. Our results revealed an association between these survivors and molecular markers for the *kdr* mutation, in particular a *super-kdr* (L1014F + M918I) genotype. The development and application of single-locus genetic markers as demonstrated in this study is not commonly undertaken for crop pests, and we discuss their potential use as diagnostic tools for predicting the occurrence of resistant phenotypes in field populations.

## Materials and methods

### Aphid populations

Laboratory colonies were established for susceptible and pyrethroid resistant *A. glycines*, and used to explore the occurrence and phenotype of *vgsc* mutations. For this, separate susceptible laboratory colonies of biotype 1 (SBA-ISU-B1) and biotype 3 (SBA-ISU-B3) were reared in a Percival growth chamber (Percival Scientific, Perry, Iowa, USA) on *G. max* cultivars LD14-8007 and LD14-8002, respectively, as described previously^35^. The biotypes of these colonies are based on their response to *G. max* containing *Rag* (Resistance to *A. glycines*) genes; Biotype 1 is avirulent on any *Rag* cultivar while Biotype 3 is virulent on *Rag2 G. max*. Both SBA-ISU-B1 and –B3 colonies were maintained at Iowa State University for ≥ 6 years, and never exposed to insecticides. Additionally, putatively resistant *A. glycines* populations were initiated from survivors collected after exposure to field-applied rates of lambda-cyhalothrin (Warrior II, Syngenta Crop Protection, Greensboro, NC) in Minnesota during 2017 (SBA-MN1-2017 and SBA-MN2-2017), and Iowa in 2017 (SBA-Sutherland-2017) and 2018 (SBA-Nashua-2018; Table 1). These populations were reared in separate growth chambers on *G. max* cultivar (NK S24-K2; Syngenta) without further exposure to insecticide. Insecticidal treatment free *G. max* seeds were sown into plastic pots filled with a soil mixture (Sungro Horticulture Products, SS#1-F1P) in plastic pots, and kept in a greenhouse at 25 ± 5 °C and a 16:8 [L:D]. Plants were watered three times per week and after emergence they were fertilized weekly with a water‐soluble formulation (Peters Excel Multi‐Purpose Fertilizer, 21‐5‐20 NPK). Aphid-free *G. max* at V2–V3 growth stage^36^ were added to population-specific lines weekly, whereon *A. glycines* propagated by parthenogenetic (clonal) reproduction.

**Table 1:** Estimates of pyrethroid (lambda-cyhalothrin) resistance among *Aphis glycines* at Iowa (Nashua and Sutherland) and Minnesota (MN1 and MN2) locations from leaf-dip bioassays, compared to susceptible controls from biotype 1 (SBA-ISU-B1) and biotype 3 (SBA-ISU-B3) specific laboratory colonies.

Collection	Origin	*n*	LC_50_^a^	95% CI^b^	Slope	χ^2c^	df	RR^d^
SBA-ISU-B1	Laboratory	480	0.38d	0.29–0.46	2.51 ± 0.45	5.08	5	-
SBA-ISU-B3	Laboratory	540	0.43d	0.36–0.50	4.22 ± 0.98	7.27	6	1.16
SBA-MN1-2017	Field	540	18.33a	13.92–22.74	2.08 ± 0.404	6.53	6	48.23
SBA-MN2-2017	Field	480	14.66a	10.42–18.91	1.91 ± 0.412	9.96	5	38.57
SBA-Nashua-2018	Field	1080	6.19b	4.49–7.90	1.25 ± 0.13	18.00	6	16.28
SBA-Sutherland-2017	Field	540	1.51c	1.18–1.83	2.86 ± 0.48	10.60	6	3.94

^a^LC_50_ values designated by different letters within a column are significantly different from each other through non‐overlap of 95% confidence intervals.

^b^CI, confidence interval.

^c^Chi‐square testing linearity of concentration‐mortality responses.

^d^Resistance ratio (RR): LC_50_ of individual test population divided by LC_50_ of susceptible SBA-ISU-B1.

### Estimates of pyrethroid survivorship among field-derived aphids

We assessed the susceptibility to lambda-cyhalothrin of *A. glycines* populations (Table 1) by comparing estimated lethal concentrations required to cause 50% mortality (LC_50_) following a leaf-dip bioassay^37^. A stock solution of technical grade lambda-cyhalothrin (97.7% purity, Control Solutions Inc., Pasadena, USA) was prepared in analytical acetone, and diluted into 7–8 treatment concentrations (0.0008–60 μg ml^−1^) with distilled water containing 0.05% (v/v) Triton X-100 (Alfa Aesar, Tewksbury, USA). The final concentration of acetone in any treatment was ≤ 0.5% (v/v). The control treatment contained distilled water, 0.05% (v/v) Triton X-100, and ≤ 0.5% (v/v) acetone. The *G. max* cultivar NK S24-K2 (Syngenta) was grown in a greenhouse at 25 ± 5 °C and a 16:8 [L:D] as described above. Leaflets from first and second trifoliate were excised from V3 to V4 *G. max*^36^, cut into disks (3.8-cm diameter) with a hole punch (Fiskars, Helsinki, Finland). Each disk was manually submerged in a solution at each treatment concentration for 10 s with gentle agitation, and then air dried on a paper towel at room temperature with the abaxial leaf side up. Subsequently, leaf disks were placed with their abaxial side up into 29.6 ml plastic souffle cups (Choice Paper Company, New York, USA) containing 1% w/v agar (Bacto™ Agar, Becton, Dickinson and Company, Franklin Lakes, USA) prior to congealing. Each cup was filled with approximately 20 ml of agar, leaving 10 ml to the top of the cups. A drop of distilled water was added to the agar bed to increase leaf disk adherence as described previously^31,37^.

Apterous, mixed-aged adult *A. glycines* from SBA-ISU-B1, SBA-ISU-B3, and field-derived populations (Table 1) were collected from leaves of laboratory-grown soybean plants and transferred to Petri plates containing a filter paper moistened with distilled water. Twenty uninjured aphids from each population were randomly selected and transferred separately onto each leaf disk. A cup was considered an experimental unit, and each treatment had three independent replications with 20 aphids each. Cups were sealed with a close-fitting, ventilated lid and stored in a growth chamber at 25 ± 2 °C, 70% RH and 16:8 L:D. Mortality was assessed 48 h post-treatment. Aphids unable to right themselves within 10 s once turned on their back were considered dead^31,37^. Slope, LC_50_ and 95% confidence interval (CI) were estimated for each population using a three-parameter log-logistic function of the ‘drc’ package in R^38^. LC_50_ values were considered different when there was no overlap of the 95% CI. A resistance ratio (RR) was calculated by dividing individual LC_50_ estimates for field collected populations or SBA-ISU-B3 by the LC_50_ of SBA-ISU-B1.

All the *G. max* plants used in the bioassays were grown from commercially available seeds. The experiments complied with relevant institutional, national, and international guidelines and legislation.

### Synergist and cross-resistance bioassays

We used the most resistant (SBA-MN1-2017) and susceptible (SBA-ISU-B1) populations from the previously described leaf-dip bioassay to explore for evidence of metabolic resistance. The effects of the cytochrome P450 monooxygenase inhibitor, piperonyl butoxide (PBO; 91.2% purity, EcoSMART Technologies, Inc., Roswell, GA), the carboxylesterase inhibitor, triphenyl phosphate (TPP) (> 99% purity, Sigma-Aldrich, St. Louis, MO, USA), and the esterase inhibitor, *S,S,S*-tributyl phosphorotrithioate (DEF) (96% purity, Crescent Chemical Co., Inc, Islandia, NY, USA), were evaluated. First, leaf-dip bioassays were performed at six concentrations of each synergist to determine the highest concentration which resulted in ≤ 10% mortality in SBA-ISU-B1. Leaf-dip assays were performed as described above, except leaf discs were treated with a constant rate of PBO, TPP, or DEF (100 μg ml^−1^) and a range of lambda-cyhalothrin (0.0008–60 μg ml^−1^). The synergist alone served as the treatment control. Mortality was measured after 48 h. A three-parameter log-logistic function of the ‘drc’ package in R^38^ was used to estimate the slope, LC_50_ and fiduciary 95% CI, and synergist ratio (SR; LC_50_ estimate lambda-cyhalothrin alone ÷ LC_50_ estimate lambda-cyhalothrin with PBO, TPP, or DEM) calculated for all dose-responses.

To assess patterns of cross-resistance to other insecticides, concentration–response leaf-dip bioassays were performed using SBA-MN1-2017 (pyrethroid-resistant) and SBA-ISU-B1 (susceptible) populations. We used a pyrethroid (bifenthrin, 98% purity, Chem Service, West Chester, PA), a pyridinecarboxamide (flonicamid, 99.5% purity, Sigma-Aldrich, St. Louis, MO, USA), a butenolide (flupyradifurone, 99.8% purity, Sigma-Aldrich, St. Louis, MO, USA), a sulfoximine (sulfoxaflor, analytical standard, Down Agrosciences, Indianapolis, IN, USA), and a tetramic acid derivative (spirotetramat, 99.6% purity, Sigma-Aldrich, St. Louis, MO, USA). All were conducted using leaf-dip bioassays as described above, except mortality was assessed at post-exposure periods of 48 h for bifenthrin, flupyradifurone and sulfoxaflor, 72 h for spirotetramat, and 96 h for flonicamid. Slope, LC_50_ and fiduciary 95% CI were estimated as described above, and resistance ratio (RR; LC_50_ estimate for SBA-MN1-2017 ÷ LC_50_ values for SBA-ISU-B1).

### cDNA sequencing of voltage-gated sodium channel alleles

The molecular basis for differing levels of pyrethroid resistance among the *A. glycines* laboratory populations (Table 1) estimated from between leaf-dip bioassays (above) was investigated next. This involved a candidate gene approach to predict the association of any nucleotide differences (mutations) in the full-length *A. glycines vgsc* transcript sequence in the pyrethroid-resistant populations. Since *vgsc* genes are not well annotated in the current *A. glycines* genome assembly, the 2105 amino acid conceptional translation (AAB47604.1) from the house fly, *Musca domestica, para*-type *vgsc* gene^39^ (accession U38813.1) was used as the query to search protein models from the official gene set (OGS) v 6.0 (Ag_bt1_v6.0) of the *A. glycines* biotype 1 genome^40^. We searched with the BLASTp algorithm at a web interface maintained at AphidBase (https://bipaa.genouest.org/sp/aphis_glycines/blast/)^41^, and filtered results for “hits” with *E*-values ≤ 1.0e^−100^. Genome scaffold positions of gene models were retrieved from the OGS6.0_20180125.gff3 file. BLAST output was used to define the targets for our subsequent confirmatory sequencing and generation of evidence for gene annotation.

To generate evidence for gene annotation, putative *vgsc* transcripts (cDNAs) corresponding to *A. glycines* Ag_bt1_v6.0 gene models AG6007485, AG6007488, and AG6007489 were sequenced from susceptible and field-derived resistant populations (Table 1). Specifically, oligonucleotide primer pairs AG6007485-F and -R, and AG6007489-F: (5′-ATG AGT GTG TAT AGT AGT GAG GAA CTC C-3’) and AG6007488-R (Table S1A: 5′-TTA GAC ATC GGC GAG TCT TGA G-3′) were designed using Primer3^42^ (start ATG codons and reverse complements of stop codons TAA and TAG are double underlined). Total RNA was extracted in duplicate from a pool of 2–3 mixed age apterous *A. glycines* from each population using the RNeasy® Plus Micro Kit (Qiagen Hilden, Germany) according to the manufacturer’s protocol. Genomic DNA contamination was removed using TURBO DNA-free™ kit (Ambion®, Life Technologies, Carlsbad, CA, USA) according to the manufacturer’s directions. First strand cDNA was synthesized from 30 to 200 ng DNase-treated total RNA in iScript™ Reverse Transcription Supermix (Bio-Rad, Hercules, California, USA) reactions with an extension of 30–45 min at 46 °C. RT-PCR reactions included 18.625 μl deionized H_2_0, 10.0 μl 5X GoTaq polymerase buffer (Promega, Madison, WI, USA), 3.75 µl 25.0 mM MgCl_,_ 0.3125 µl 25 mM dNTPs, 1.0 µl of each forward and reverse primer pair (10 µM), 0.3125 µl of 5U µl^−1^ Go*Taq* DNA polymerase (Promega), and 15.0 µl of 10 ng µl^−1^ first strand cDNA. Amplification was performed by a touchdown procedure: initial denaturation at 96 °C for 2.5 min, then seven cycles of 96 °C for 30 s, 66 °C for 30 s (decreasing 2 °C each cycle), 72 °C for 8 min, then 40 cycles of 96 °C for 30 s, 54 °C for 30 s, 72 °C for 8 min with a final extension at 72 °C for 10 min on a Tetrad2 thermocycler (BioRad). PCR products (10.0 µl each) were separated by 1% agarose gel electrophoresis, and residual primers dephosphorylated and degraded in the remaining PCR volume as described earlier^43^. Treated PCR products were diluted 1:8 with deionized H_2_O and submitted to the Iowa State University DNA Facility (Ames, IA, USA) for a bidirectional primer walking by Sanger sequencing on an ABI3700 (Applied Biosystems, Forest City, CA, USA) using internal oligos (Table S2).

Trimmed high quality Sanger reads were assembled into individual cDNAs using CAP3^44^, and conceptual translations were predicted for each using TransDecoder v5.5.0 (https://github.com/TransDecoder/TransDecoder/releases). A multiple translated protein sequence alignment between *M. domestica* (AAB47604.1), the *A. glycines* gene model AG6007485-RA, and our cDNAs was generated using the Clustal Omega algorithm^45^ (default parameters) with the web-based tool located at https://www.ebi.ac.uk/Tools/msa/clustalo/. Structural annotations consisting of four domains (DI-DIV) each with six transmembrane region (TMR) segments (S1–S6) from the *M. domestica vgsc* protein^16^ were used to identify orthologous positions in aligned *A. glycines* proteins. Multiple sequence alignments were similarly generated between the conceptual translations from *A. glycines* gene models and cDNAs, and orthologs in GenBank accessions from aphids (NCBI Taxonomy ID 27,482). These included recently published translations for *A. glycines* (QTJ01838.1–QTJ01843.1)^23^.

Comparisons among assembled *A. glycines vgsc* cDNAs were then used to predict variation with and between resistant and susceptible populations. An intraspecific multiple nucleotide sequence alignment among de novo assembled cDNAs and corresponding *A. glycines* gene models was performed using the Clustal Omega algorithm^45^ as described above. Alignments were overlaid with conceptual translations, structural annotations, and nucleotide variation within individual contigs predicted as co-occurring electropherogram peaks in constituent Sanger trace data using Pearl^46^.

### Prediction and validation of kdr and super-kdr mutations

Given that the putative *A. glycines vgsc* mutations are fixed differently between resistant and susceptible populations based on our preceding comparisons of cDNA sequencing, we subsequently (1) used direct Sanger sequencing of genomic DNA amplicons to verify these mutations within these regions and detect variation in a larger number of individual aphids from each of the laboratory populations (increased sample size compared to that for cDNA), and (2) developed and validated genetic makers to detect mutations in individual aphids. For the first goal we used predicted mutations in DIIS4-6 based on our cDNA data, and mutations in DIIIS6-DIVS1, and DIVS4-S6 previously reported to be associated with pyrethroid resistance in mosquitoes^47^, and mutations in DIIS4-S6 described in *A. glycines*^23^. Primers to amplify these regions were designed from SBAphidCtg1013 sequence data of the *A glycines* Ag_Bt_V6.0 genome assembly^40^ using Primer3^42^ (Table S1B). Individual aphids (4–8) were sampled from 1) four lines collected from fields with suspected pyrethroid resistance and susceptible laboratory populations (Tables 1) and 2) two laboratory populations previously shown to be susceptible to insecticides^23,48^ (Table S3).

**Table 2:** Levels of cross-resistance of *Aphis glycines* from field-collected SBA-MN1-2017 across different classes of insecticides; common name (IRAC classification).

Insecticide	Population	n^a^	LC_50_ (95% CI)	Slope ± SE	χ^2^ (df)^b^	RR^c^
Bifenthrin (pyrethroid)	SBA-ISU-B1	540	0.66 (0.52–0.81)	2.28 ± 0.37	11.18 (6)	33.90
	SBA-MN1-2017	540	22.38 (16.18–28.59)	1.99 ± 0.40	8.13 (6)	
Flonicamid (flonicamid)	SBA-ISU-B1	540	0.44 (0.22–0.65)	1.16 ± 0.29	4.49 (6)	0.97
	SBA-MN1-2017	540	0.43 (0.25–0.61)	1.22 ± 0.25	6.99 (6)	
Flupyradifurone (butenolides)	SBA-ISU-B1	840	0.07 (0.04–0.11)	0.65 ± 0.06	15.84 (10)	2.28
	SBA-MN1-2017	840	0.16 (0.10–0.23)	0.83 ± 0.07	10.95 (10)	
Sulfoxaflor (sulfoximines)	SBA-ISU-B1	540	0.02 (0.01–0.03)	1.07 ± 0.14	9.53 (6)	2.00
	SBA-MN1-2017	540	0.04 (0.02–0.05)	1.07 ± 0.12	3.85 (6)	
Spirotetramat (tetramic acid)	SBA-ISU-B1	540	55.07 (31.58–78.57)	1.02 ± 0.19	4.36 (6)	0.73
	SBA-MN1-2017	540	40.71 (25.40–56.01)	0.95 ± 0.13	9.51 (6)	

^a^Number of aphids tested.

^b^Chi-square (degrees of freedom).

^c^Resistance ratio (RR): LC_50_ of resistant SBA-MN1-2017 divided by LC_50_ of susceptible SBA-ISU-B1.

Genomic DNA was extracted from all aphids individually using QuickExtract™ DNA Extraction Solution (Lucigen, Middleton, WI) as described by the manufacturer, except volume adjusted to 50.0 µL per sample. DNA quantities were estimated on a DeNovix DS-11 (DeNovix Inc, Wilmington, DE, USA), and samples diluted to 10 ng µl^−1^ with deionized water. PCR reaction setup and touchdown amplification reactions were performed as described above, except reaction volumes scaled to 10 µl and included primers for DIIS4-6, DIIIS6-DIVS1, or DIVS4-S6 (Table S1B). Thermocycler extension times were reduced to 1 min. A 5.0 µl aliquot of each PCR reaction product was separated by 2% agarose gel electrophoresis. Residual primers digested and dephosphorylated in remaining product volumes, then diluted 1:8 or 1:10 and submitted for Sanger sequencing with corresponding forward or reverse primers as described above. Electropherogram data were aligned against genomic scaffold SBAphidCtg1013, trimmed, and variant nucleotide positions predicted at a Phred quality cutoff score of 20 using novoSNP^49^. Resulting trimmed sequences were aligned and annotated as described above and accessioned in the NCBI non-redundant nucleotide database.

The putative *A. glycines* L1014F *kdr* mutation was detected by a PCR-restriction enzyme fragment length polymorphism (RFLP) assay. Primers AG*kdr*-F and -R primers (Table S1C) were designed to amplify a 439 bp fragment in DII S6 (positions 2847–3286 of AG6007485-RA) with a single *Bst*EII restriction enzyme recognition site (GGTNAA[C/A]; variant nucleotides causing L1014F mutation in brackets) using Primer3^42^, and synthesized at Integrated DNA Technologies (IDT; Coralville, IA, USA). Individual reactions consisted of 3.75 μl deionized H_2_0, 2.0 μl 5X GoTaq polymerase buffer (Promega, Madison, WI), 0.75 µl 25.0 mM MgCl_2_, 0.0625 µl 25 mM dNTPs, 0.1875 µl of each forward and reverse primer at 10 µM, 0.0625 µl of 5U µl^−1^ Go*Taq* DNA polymerase (Promega), and 3.0 µl of 10 ng µl^−1^ gDNA. All gDNA templates used for *kdr* validation were from the same samples used in Sanger sequencing (above). Amplification of the locus used a touchdown procedure^43^. The entire volume of each PCR product (10.0 μl) was digested by addition of 8.9 μl deionized H_2_0, 1.0 μl 10X Buffer 3.1 (New England Biolabs, Ipswich, MA), and 0.1 μl *Bst*EII (0.1 U; New England Biolabs). Digestion reactions were incubated overnight at 60 °C, then separated by 2% agarose gel electrophoresis. Samples were genotyped based on two fragments (154 and 285 bp) among homozygotes for susceptible alleles, an undigested 439 bp fragment for homozygous resistant individuals, and heterozygotes defined by co-occurrence of resistant and susceptible alleles (154 bp, 285 bp, and 439 bp).

A ligase chain reaction (LCR) based marker assay was developed to detect the *A. glycines* M918I mutation. For this, a 151 bp region of the *vgsc* gene encompassing the M918I locus (positions 272–2876 of AG6007485-RA) was PCR amplified with primers AG*skdr*-F and -R (Table S1C). The same reaction and thermocycler parameters and samples were used as for the *kdr* amplicon (above). PCR reaction products were diluted 1:20 using deionized H_2_0, and used as template in subsequent LCR reactions.

LCR assays consisted of three separate oligonucleotide probes. The upstream allele-specific wildtype susceptible P1-918_Met_G and mutant P1-918_Ile_A probes (Table S1C). The 5′-phosphorylated P2-918_Phos probe annealing downstream and immediately adjacent to P1 probes. Individual 10.0 µl LCR reactions included 1.0 µl of 10X *Taq* DNA Ligase Reaction Buffer (New England Biolabs), 0.4 µl of *Taq* DNA Ligase (40U µl^−1^; New England Biolabs), 1.0 µl of each P1-918_Met_G, P1-918_Ile_A, and P2-Phos probes at 0.2 µM, and 5.0 µl of 1:20 diluted AG*skdr*-F and -R amplified PCR product as template. Ligation reactions were incubated at 94 °C for 2 min, followed by 2 cycles of 94 °C for 20 s and 75 °C for 10 min, and then held at 15 °C.

LCR reaction were diluted 1:20 with deionized H_2_O, and 2.0 µl used in PCR reaction and amplification conditions identical to those above except for use of M13_5p17nt-F and M13_5p18nt-R primers (Table S1C). Genotypes were determined according to predicted sizes from amplified probes specific for wildtype P1-918_Met_G (141 bp) and mutant P1-918_Ile_A alleles (165 bp) following 3% agarose gel electrophoresis.

### In-field association of pyrethroid resistant genotype to phenotype

We conducted two experiments to determine the relationship between markers for the *vgsc* mutations and *A. glycines* survival when exposed to pyrethroids. We used field collected aphids for both experiments. In the first experiment, we used a previously determined diagnostic concentration of lambda-cyhalothrin and bifenthrin developed for glass-vial bioassays to assign aphids to survivor (putative resistant) and moribund (susceptible) groups^32^. This diagnostic concentration is an accepted tool that can be used by field entomologists for making management decisions. Aphids tested within the bioassays were subsequently genotyped with our L1014F *Bst*EII PCR–RFLP and M918I LCR genetic markers. We tested the hypothesis that survivors of the glass-vial bioassay would have a higher frequency of mutations in the *vgsc* genes.

Individual *A. glycines* were collected from three locations (Darwin, Sutherland, and Kanawha) with a history of frequent pyrethroid use to manage *A. glycines* and one (Boone) with lack of this history. Aphids were collected during August 2019 and had not been treated with foliar insecticides. In 2020, aphids were similarly collected at two locations (Sutherland and Kanawha) in late July and early August (Table S3). Infested *G. max* leaflets were collected randomly from approximately 40 plants at each location, and transported to the laboratory. Leaflets were transferred to Petri plates containing a moistened filter paper, sealed with Parafilm and incubated in a growth room at 25 ± 2 °C, 50% RH and 16:8 [L:D]. Aphids were taken from leaflets for use in glass-vial bioassays within 1 week after collection. The bioassays were based on a previously published methodology^31,32^ using 20-ml glass-vials coated with technical grade of bifenthrin (0.0215 μg A.I./0.5 ml/vial) and lambda-cyhalothrin (0.2521 μg A.I./0.5 ml/vial), along with control treatment (acetone). Briefly, ten healthy apterous mixed-age adult aphids were transferred to the bottom of each treated glass-vial, capped, and incubated upright at room temperature. Mortality was assessed 4 h post-infestation^31,32^. Surviving and moribund (dead) aphids were collected, placed individually into 1.5 ml microcentrifuge tubes, and stored at − 20** °C**. DNA was extracted and genotypes were determined for survivor and moribund aphids using L1014F *Bst*EII PCR–RFLP and M918I LCR assays as described above. The association between genotypes and corresponding phenotype, surviving (resistant) *vs*. moribund (susceptible) following bioassay, was performed for each field collection site using Fisher’s exact tests implemented in R version 3.5.1^50^.

In a second experiment, changes in the frequency of genotypes before and after an application of lambda-cyhalothrin (Warrior II, Syngenta Crop Protection, Greensboro, NC; full rate of 0.14 l ha^−1^) were assessed at three locations in 2019 and at two locations in 2020 (Table 5; Table S3). For this, a “pre-application” sample was taken < 7 days prior to the foliar application of lambda-cyhalothrin, and a “post-application” sample taken 2–3 days after an application. L1014F *Bst*EII PCR–RFLP and M918I LCR assays were performed as described above on individual aphids from pre- and post-application samples. Differences in genotypic frequencies between pre- and post-application groups were analyzed for each field using Fisher’s exact tests implemented in R version 3.5.1^50^. A binomial generalized linear model (GLM) with a logit link function implemented in base R^50^ was used to evaluate changes in resistance allele frequency (RAF) between groups. The model included time (pre- and post-application) and location as predictor variables, and RAF for 1014F *kdr* and 918I compared to the wild type susceptible (S) alleles 1014L and 918 M, respectively, as explanatory variables. Estimates of allele frequencies, confidence intervals, contrasts, and odds ratios (OR) were computed using the R package ‘emmeans’^51^.

## Results

### Estimates of pyrethroid survivorship among field-derived aphids

Initial leaf-dip bioassay results revealed that populations collected from fields with a history of reduced pyrethroid efficacy had significantly higher estimated LC_50_ for lambda-cyhalothrin compared to susceptible controls. Specifically, the LC_50_ estimates for susceptible SBA-ISU-B1 (0.38 ± 0.09 μg ml^−1^) and SBA-ISU-B3 (0.43 ± 0.07 μg ml^−1^) were significantly lower than the LC_50_ estimated for all field-collected aphids (range 1.51 ± 0.32 to 18.33 ± 4.41 μg ml^−1^; Table 1). The corresponding RR of the field populations derived from the LC_50_ of the SBA-ISU-B1 ranged from 3.94 to 48.23.

### Synergist and cross-resistance bioassays

Bioassays that included a pyrethroid with a detoxification enzyme inhibitor (i.e. synergists) did not significantly affect the estimated LC_50_ for SBA-MN1-2017 based on the non-overlapping 95% confidence intervals (CI). The synergist ratio for PBO, TPP and DEF was estimated at 1.27, 1.28 and 1.30, and 1.31, 1.26, and 1.31 for SBA-MN1-2017 and SBA-ISU-B1, respectively (Table S4). The exposure of pyrethroid-resistant and susceptible populations to other insecticides revealed limited variation, indicating no cross-resistant to insecticides with a different mode of action (MoA; Table 2). SBA-MN1-2017 was 33.90-fold more resistant to bifenthrin compared to the susceptible control. There were no significant differences in estimated LC_50_ between SBA-MN1-2017 and SBA-ISU-B1 for the other insecticides.

### cDNA sequencing voltage-gated sodium channel alleles

**Figure 1:**
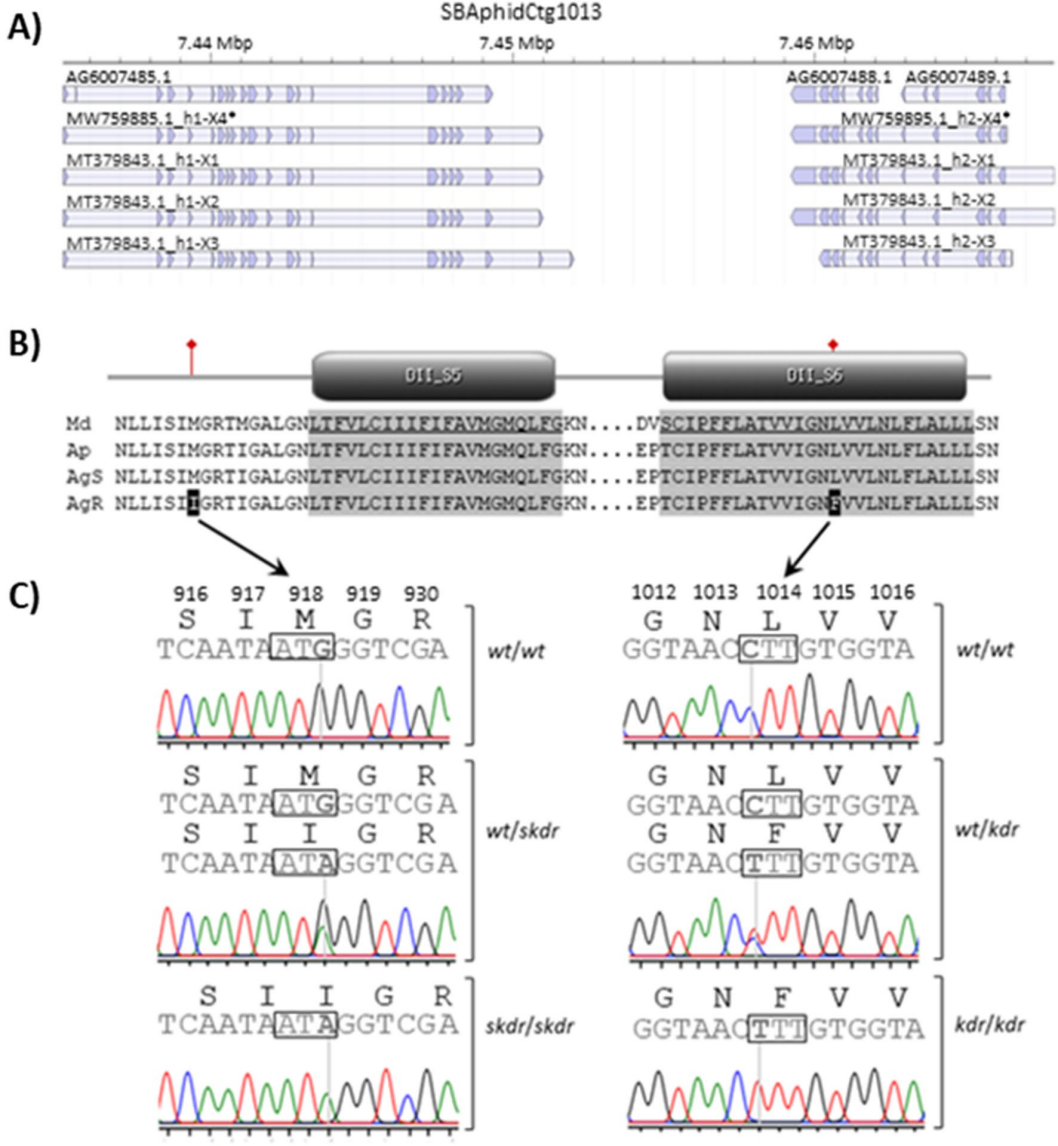
Structural variation in the *Aphis glycines* voltage gated sodium channel gene, *vgsc*, and validation of mutations associated with pyrethroid resistance within field populations. (**A**) Genome organization of the *A. glycines vgsc* on SBAphidCtg1013 of the Ag_bt1_v6 assembly^40^ with predicted gene models AG60074085.1, AG60074088.1 and AG60074089.1. The *vgsc* heterodimer (*vgsc*-*h*1 and -*h*2) isoforms X1, X2, and X3 were annotated based on previous evidence (GenBank accession MT379843.1)^23^, and isoform X4 supported by sequence assemblies in this study (* representative full-length cDNA accession; MW759883.1 to MW759893.1). (**B**) Partial alignment of conceptual protein translations for *vgsc* orthologs from *Musca domestica* (Md; GenBank accession AAB47604.1)*, Acyrthosiphon pisum* (Ap; XP_008183365.1), and *A. glycines* pyrethroid resistant (AgR; MW75988.1–MW759893.1) and susceptible (AgS; MW759883.1 and MW759884.1) alleles showing domain II (DII) segment 5 (S5) and 6 (S6), and positions of knockdown (*kdr*) and super-*kdr* (*skdr*) mutations. (**C**) Representative electropherograms from Sanger sequencing with arrows showing substitution mutations predicted to cause amino acid variation at positions 918 (M918I) and 1014 (L1014F; *kdr*), where pyrethroid resistant *A. glycines* are homozygous (*kdr*/*kdr*) or heterozygous for the alleles encoding 1014F allele (*wt*/*kdr*). A portion of resistant *A. glycines* genotypes show *kdr* mutations in combination with the M918I mutation. Electropherograms from *A. glycines* populations shown in Fig. S4.

Results of BLAST searches and evidence from our full-length cDNA sequencing defined two *A. glycines vgsc* heterodimers that together comprise the complete coding sequence that was lacking from the current gene models. A search of proteins from the Ag_bt1_v6 OGS with the translated *M. domestica vgsc* protein sequence, AAB47604.1, identified three putative hits; the 1174 aa AG6007485-PA, 649 aa AG6007488-PA, and 359 aa AG6007489-PA (*E*-values ≤ 2.0e^−103^, identities ≥ 52.7%). Parent transcripts AG6007485-RA, AG6007488-RA, and AG6007489-RA were 3525, 1950, and 1080 bp, respectively, and were encoded on contig SBAphidCtg1013 Ag_bt1_v6 (Fig. 1A). These results demonstrated that the *A. glycines* gene model is fragmented. Our annotation data came from full-length cDNA amplification products which provided evidence for two distinct *A. glycines vgsc* transcripts. Transcript sizes did not vary within or between susceptible and resistant individuals; an ~ 3,500 bp product from AG6007485-RA and an ~ 3,100 bp cDNA produced from primers annealing to the C-terminal CDS of AG6007488-RA and N-terminal CDS of AG6077489-RA (results not shown). AG6007485-RA and combined AG6007488-RA and AG6077489-RA gene models were referred to a heterodimer H1 (*vgsc-h1*) and H2 (*vgsc-h2*), respectively, following convention for heterodimeric *vgsc* among aphids. Assembly of 2749–3453 bp cDNA sequences (contigs) for AG6077485-RA from twelve individuals resulted in six with a putative full-length 1150 aa translated open reading frame (ORF) (GenBank accessions MW759883.1–MW759893.1; Fig. S1). The 3453 bp consensus *vgsc-h1* cDNA showed ≥ 99.8% nucleotide similarity to the 3525 bp gene model AG6007485-RA, and 3489, 3453, and 3588 bp isoforms, X1, X2, and X3, respectively, previously predicted in accession MT379843.1. Intraspecific splice variation involved 33 bp (11 aa) of exon 2 and the initial 39 bp of exon 16 in AG6007485-RA compared to all cDNAs in this study, and inclusion of a single valine (GTA) in MT379843.1 isoform X2 (Fig. S1).

Translated *A. glycines vgsc-h1* transcript variants showed ≥ 68.48% identity when aligned to *M*. *domestica* AAB47605.1 wherein residues putatively orthologous to DI S1-S6 and DII S1-S6 were identified in *A. glycines* (Fig. S2) and in other aphid species (Fig. S3). This orthology was also used to define location of putative variation among *A. glycines*. Five putative nucleotide variant sites (e.g. single nucleotide polymorphisms, SNPs) were predicted among *A. glycines vgsc-h1* cDNAs (Fig. S1), two that putatively cause amino acid changes in DII S1-S6; a C–T transition mutation at AG6007485-RA position 3070 (1st codon position of residue 1014) leads to a nonsynonymous leucine to phenylalanine change (L1014F), and a G–A transition at 2784 was predicted to cause a methionine to isoleucine change at amino acid position 918 (M918I) in AG6007485-PA (Fig. 1B). Corresponding electropherograms showed co-occurring C and T signals (pyrimidine; Y) at position 3070 (Fig. S4A), and G and A (purine; R) at AG6007485-RA position 2784 (Fig. S4B). Electropherograms with one or both of these co-occurring signals (Fig. 1C) were only observed in *A. glycines* resistant to the pyrethroid lambda-cyhalothrin. No other amino acid changing mutations were detected.

The 13 independently assembled *A. glycines vgsc-h2* cDNAs were between 2682 and 2877 bp (MW759894.1–MW759904.1), wherein six encoded a complete 958 aa ORF and all encoded DIII S1-6 and DIV S1-6 (named isoform *vgsc-h2* X5; Fig. S5). Assemblies provided evidence for a merger of gene models AG6007488-RA and AG6077489-RA (Fig. 1A), predicted to encode DIII S1-6 and DIV S1-6. Our cDNAs encoded a protein within the 813–960 aa range and showed ≥ 92.9% identity to other aphid *vgsc* proteins (Fig. S6). N-terminal residues in previously defined isoforms X1 and X2 predicted in QTJ01841.1 and QTJ01842.1 (*n* = 36), respectively, or isoform X3 in QTJ01843.1 (*n* = 9) were not encoded in our isoform X4 nor shared with any aphid orthologs (Fig. S6). Additionally, isoforms X1 and X3 encode a 41 aa insertion that putatively interrupted DIIIS4. A multiple sequence alignment among *A. glycines vgsc-h2* transcripts identified 13 variant sites, 10 of which were in 3rd codon positions and not predicted to cause amino acid changes (Fig. S5). Of the three nonsynonymous changes, those orthologous to *M. domestica* positions 1219, 1424, and 1430 caused putative glycine to serine, leucine to valine, and valine to glycine changes, respectively. The G1219S mutation was in a non-conserved linker region between DIIS6 and DIIIS1. V1424G and L1430V mutations are both in the DIII S5 α-helix, of which the former is only predicted in two resistant aphids to involve residues with short chain aliphatic side changes. The L1430V mutation was predicted in resistant as well as susceptible aphids.

### Prediction and validation of kdr and super-kdr mutations

Results of comparisons made among Sanger reads generated from short genomic DNA amplicons from a larger sample set provided secondary confirmation of cDNA-based predictions. Specifically, comparison of aligned Sanger sequence from DIIS4-S6 (range 1323–949 bp; GenBank accessions MW846869-MN846958; Fig. S7), DIIIS6-DIVS1 (414 bp; MW847052-MW847146; Fig. S8), and DIVS4-S6 (536 bp; MW846959-MW847051; Fig. S9) predicted four, one, and two nucleotide substitutions, respectively. Of these seven variable sites, five were predicted in 3rd codon positions and to be synonymous (non-amino acid changing). Two of four variants among *vgsc-h1* DIIS4-S6 fragments were in introns, and two putatively causing amino acid changes when comparing among susceptible SBA-ISU-B1 -B3, and -B4 and field collected resistant SBA-MN1-2017, SBA-MN2-2017, SBA-Sutherland-2017, and SBA-Nashua-2018 populations (Tables 1, 3). Specifically, a putative G–A transition in the extracellular loop region between DII S4 and S5 at position 87 of the 1323 bp consensus alignment (Fig. S7A; partial alignments shown) was predicted to cause a methionine to isoleucine mutation at aa position 918 (Fig. S1; Fig. 1B). The M918I locus was predicted to be homozygous for the wildtype G allele (single electropherogram peak) among all 21 susceptible and 21 resistant aphids, but heterozygous with co-occurring G and A nucleotides (Fig. 1C) for 17 aphids from the resistant lines. No A nucleotides were predicted among susceptible aphids. This DIIS4-S6 fragment also showed a C–T transition (Fig. S7B; Table 3), leading to a putative L1014F *kdr* mutation in the *A. glycines vgsc-h1* DII S6 transmembrane region (Fig. S1; Fig. 1B). All aphids from susceptible lines were homozygous for the C allele. In contrast, 33 and 5 aphids from the resistant lines were heterozygotes (co-occurring C and T peaks) and homozygous for the T allele, respectively (Fig. 1C).

**Table 3:** Nonsynonymous mutations associated with pyrethroid resistance identified in the voltage-gated sodium channel genes of *Aphis glycines* at positions 1014 (L1014F) and 918 (M918I) from laboratory and field-collected aphids. Genotypes predicted from Sanger sequences from individual aphids (Fig. S7–S9).

Collection	Origin	M918I	L1014F
SBA-ISU-B1	Laboratory	SS	SS
SBA-ISU-B3	Laboratory	SS	SS
SBA-ISU-B4	Laboratory	SS	SS
SBA-UIL-B1	Laboratory	SS	SS
SBA-MN1-2017	Field	SS and RS	RS and RR
SBA-MN2-2017	Field	SS and RS	RS and RR
SBA-Nashua-2018	Field	SS and RS	RS
SBA-Sutherland-2017	Field	SS and RS	RS

Furthermore, our results validated two single locus genetic markers that detect nonsynonymous (amino acid changing) mutations in the *A. glycines vgsc-h1* DII S1-S6. Preliminary validation of the L1014F *kdr* mutation by a *Bst*EII PCR–RFLP assay resulted in digestion reaction fragments of 154 bp and 285 bp across all individuals from the SBA-ISU-B1 population (*n* = 16), and corresponded to homozygotes for the C nucleotide that retain the *Bst*EII recognition sequence (5′-GGTNAC**C**-3′) and fixed for the leucine amino acid (Fig. 1B). In contrast, all 16 aphids from the SBA-MN1-2017 population were heterozygotes showing three gel fragments in *Bst*EII PCR–RFLP assays; 154, 285, and 439 bp. These corresponded to an overlap in 154 and 285 bp fragments indicative of the C nucleotide alleles and the non-digested 439 bp fragment derived from alleles with a T nucleotide that removes the *Bst*EII recognition site (5′-GGTNACT-3′) and encoded a phenylalanine (F) amino acid.

The LCR assay resulted in a single 141 bp amplified fragment derived from ligation of P1-918_Met_Gand P2-918_Phos probes among all susceptible SBA-ISU-B1 individuals, and corresponded to predicted susceptible G allele homozygotes encoding a methionine at *A. glycines vgsc-h1* position 918. This 141 bp fragment was also amplified along with a 165 bp fragment among all resistant SBA-MN1-2017 individuals, where the latter corresponded to predicted size of the P1-918_Ile_A P2-918_Phos probe ligation product. This co-amplification represented heterozygous genotypes, with alternate alleles encoding methionine (M) and isoleucine (I) amino acids at position 918. A single 165 bp LCR product was not generated among pyrethroid-resistant SBA-MN1-2017 individuals.

### In-field association of pyrethroid resistant genotype to phenotype

In our first experiment, we observed significant changes in the frequency of *vgsc* mutations in *A. glycines* following exposure to a diagnostic concentration of bifenthrin and lambda-cyhalothrin (*n* = 462 aphids tested across both insecticides and all locations; Table 4). For all aphids, the frequency of *kdr* mutations was greater in survivors than moribund aphids (Table 4). The *super-kdr* heterozygote genotype (1014 L/F:918 M/I) showed the greatest proportional increase (≥ 16.9%) among survivors across locations in both years, whereas wild type aphids (L/L:M/M) decreased ≥ 10.7%. We also observed variation by year and active ingredient, for example, genotype frequency changed significantly after bifenthrin was applied only in 2019. In 2020, the proportions of *A. glycines* with a mutation changed significantly between survivor and moribund aphids exposed to lambda-cyhalothrin (*P*-value = 0.024), but not bifenthrin (*P*-value = 0.722; Table 4). We did not observe a significant change in genotypic frequency between survivor and moribund aphids following bifenthrin exposure despite 94.4% of survivors being homozygous *kdr* (F/F:M/M) (Table 4).

**Table 4:** Association of survival among randomly sampled *Aphis glycines* from field populations with different voltage-gated sodium channel (vgsc) amino acid (aa) changes (L1014F:M918I). Phenotypes defined as survivor and moribund following exposure to bifenthrin and lambada-cyhalothrin at LC_99_ levels in glass-vial bioassays.

Location	Phenotype	n	*A. glycines vgsc* genotype (L1014F:M918I) ^a^									
			L/L:M/M	L/L:M/I	L/L:I/I	L/F:M/M	L/F:M/I	L/F:I/I	F/F:M/M	F/F:M/I	F/F:I/I	*P*
**2019—Bifenthrin**												
Boone-IA	Moribund	32	2 (6.3)	10 (31.2)	0 (0.0)	19 (59.4)	1 (3.1)	0 (0.0)	0 (0.0)	0 (0.0)	0 (0.0)	0.048
	Survivor	8	1 (12.5)	2 (25.0)	0 (0.0)	5 (62.5)	0 (0.0)	0 (0.0)	0 (0.0)	0 (0.0)	0 (0.0)	
Kanawha-IA	Moribund	29	0 (0.0)	0 (0.0)	0 (0.0)	28 (96.6)	0 (0.0)	0 (0.0)	1 (3.4)	0 (0.0)	0 (0.0)	0.083
	Survivor	11	0 (0.0)	0 (0.0)	0 (0.0)	8 (72.7)	1 (9.1)	0 (0.0)	2 (18.2)	0 (0.0)	0 (0.0)	
Sutherland-IA	Moribund	37	5 (13.5)	1 (2.7)	0 (0.0)	27 (73.0)	4 (10.8)	0 (0.0)	0 (0.0)	0 (0.0)	0 (0.0)	0.444
	Survivor	2	0 (0.0)	0 (0.0)	0 (0.0)	0 (0.0)	2 (100)	0 (0.0)	0 (0.0)	0 (0.0)	0 (0.0)	
Darwin-MN	Moribund	20	4 (20.0)	3 (15.0)	0 (0.0)	7 (35.0)	3 (15.0)	0 (0.0)	3 (15.0)	0 (0.0)	0 (0.0)	0.012
	Survivor	17	0 (0.0)	1 (5.9)	0 (0.0)	9 (52.9)	6 (35.3)	0 (0.0)	1 (5.9)	0 (0.0)	0 (0.0)	
All locations	Moribund	118	11 (9.3)	14 (11.9)	0 (0.0)	81 (68.6)	8 (6.8)	0 (0.0)	4 (3.4)	0 (0.0)	0 (0.0)	0.008
	Survivor	38	1 (2.6)	3 (7.9)	0 (0.0)	22 (57.9)	9 (23.7)	0 (0.0)	3 (7.9)	0 (0.0)	0 (0.0)	
**2019—Lambda-cyhalothrin**												
Boone-IA	Moribund	40	12 (30.0)	3 (7.5)	0 (0.0)	23 (57.5)	2 (5.0)	0 (0.0)	0 (0.0)	0 (0.0)	0 (0.0)	N/A
	Survivor	0	0 (0.0)	0 (0.0)	0 (0.0)	0 (0.0)	0 (0.0)	0 (0.0)	0 (0.0)	0 (0.0)	0 (0.0)	
Kanawha-IA	Moribund	35	0 (0.0)	0 (0.0)	0 (0.0)	34 (97.1)	0 (0.0)	0 (0.0)	1 (2.9)	0 (0.0)	0 (0.0)	0.083
	Survivor	5	0 (0.0)	0 (0.0)	0 (0.0)	1 (20.0)	2 (40.0)	0 (0.0)	2 (40.0)	0 (0.0)	0 (0.0)	
Sutherland-IA	Moribund	15	5 (33.3)	1 (6.7)	0 (0.0)	5 (33.3)	4 (26.7)	0 (0.0)	0 (0.0)	0 (0.0)	0 (0.0)	0.079
	Survivor	22	3 (13.6)	2 (9.1)	0 (0.0)	8 (36.4)	9 (40.9)	0 (0.0)	0 (0.0)	0 (0.0)	0 (0.0)	
Darwin-MN	Moribund	20	4 (20.0)	6 (30.0)	0 (0.0)	8 (40.0)	1 (5.0)	0 (0.0)	1 (5.0)	0 (0.0)	0 (0.0)	0.040
	Survivor	20	1 (5.0)	5 (25.0)	0 (0.0)	5 (25.0)	9 (45.0)	0 (0.0)	0 (0.0)	0 (0.0)	0 (0.0)	
All locations	Moribund	110	21 (19.1)	10 (9.1)	0 (0.0)	70 (63.6)	7 (6.4)	0 (0.0)	2 (1.8)	0 (0.0)	0 (0.0)	0.008
	Survivor	47	4 (8.5)	7 (14.9)	0 (0.0)	14 (29.8)	20 (42.6)	0 (0.0)	2 (4.3)	0 (0.0)	0 (0.0)	
**2020—Bifenthrin**												
Nashua-IA	Moribund	38	27 (71.0)	0 (0.0)	0 (0.0)	9 (23.7)	2 (5.3)	0 (0.0)	0 (0)	0 (0.0)	0 (0.0)	N/A
	Survivor	0	0 (0.0)	0 (0.0)	0 (0.0)	0 (0.0)	0 (0.0)	0 (0.0)	0 (0.0)	0 (0.0)	0 (0.0)	
Sutherland-IA	Moribund	20	0 (0.0)	0 (0.0)	0 (0.0)	0 (0.0)	0 (0.0)	0 (0.0)	20 (100)	0 (0.0)	0 (0.0)	0.222
	Survivor	18	0 (0.0)	1 (5.6)	0 (0.0)	0 (0.0)	0 (0.0)	0 (0.0)	17 (94.4)	0 (0.0)	0 (0.0)	
All locations	Moribund	58	27 (46.6)	0 (0.0)	0 (0.0)	9 (15.5)	2 (3.4)	0 (0.0)	20 (34.5)	0 (0.0)	0 (0.0)	0.722
	Survivor	18	0 (0.0)	1 (5.6)	0 (0.0)	0 (0.0)	0 (0.0)	0 (0.0)	17 (94.4)	0 (0.0)	0 (0.0)	
**2020—Lambda-cyhalothrin**												
Nashua-IA	Moribund	29	17 (58.6)	1 (3.4)	0 (0.0)	10 (34.5)	1 (3.4)	0 (0.0)	0 (0.00)	0 (0.0)	0 (0.0)	0.107
	Survivor	8	3 (37.5)	0 (0.0)	0 (0.0)	4 (50.0)	0 (0.0)	0 (0.0)	1 (12.5)	0 (0.0)	0 (0.0)	
Sutherland-IA	Moribund	33	1 (3.0)	0 (0.0)	0 (0.0)	3 (9.1)	0 (0.0)	0 (0.0)	29 (87.9)	0 (0.0)	0 (0.0)	0.333
	Survivor	3	0 (0.0)	0 (0.0)	0 (0.0)	0 (0.0)	0 (0.0)	0 (0.0)	3 (100.0)	0 (0.0)	0 (0.0)	
All locations	Moribund	62	18 (29.0)	1 (1.6)	0 (0.0)	13 (21.0)	1 (1.6)	0 (0.0)	29 (46.8)	0 (0.0)	0 (0.0)	0.024
	Survivor	11	3 (27.3)	0 (0.0)	0 (0.0)	4 (36.4)	0 (0.0)	0 (0.0)	4 (36.4)	0 (0.0)	0 (0.0)	

Counts given for diploid individuals with predicted amino acids (aa/aa) at each locus (1014:918). Numbers in parenthesis represent the percentage of each genotype. Significance of association between encoded aa and aphid survival determined using Fisher’s exact tests.

^a^Alleles at the *A. glycines* vgsc 1014 *kdr* locus encoding leucine (L; CTT codon) or phenylalanine (F; TTT codon) at amino acid position 1014: homozygous susceptible (L/L), heterozygote (L/F), or homozygous resistant (F/F); Alleles at encoding methionine (M; ATG codon) or isoleucine (I; ATA codon) at amino acid position 918: homozygous susceptible (M/M), heterozygote (M/I), or homozygous resistant (I/I).

In our second experiment, we observed significant changes in the RAF for aphids collected pre- and post-application of a pyrethroid in the field (Table 5). Based on a total of 575 pre-application and 378 post-application *A. glycines* collected and genotyped from fields in Iowa*,* we observed a significant difference in genotypic frequencies between pre- and post-application in 2019 and 2020 when pooled across locations (*P*-values ≤ 0.0397; Table S5). Significant changes were detected at Boone and Kanawha in 2019, and Sutherland in 2020. The heterozygote *kdr* genotype was the most prevalent among survivors after insecticide was sprayed across all locations in 2019 (55.1%) and homozygote *kdr* was correspondingly most prevalent in 2020 (Table S5). However, the *super-kdr* heterozygote genotype showed the greatest proportional increase across locations in 2019 (+ 17.2%) and 2020 (+ 29.7%). There were significant changes in the RAF for 1014 *kdr* and 918 loci between pre- and post- application in 2019 and 2020 (*P*-values ≤ 0.0174; Table 5). A significant increase in *kdr* allele frequency was detected at two of three Iowa locations in 2019 (*P*-values ≤ 0.0240) and one of two locations in 2020 (*P*-value < 0.0001; Table 5). There was no significant change in the RAF for 1014 *kdr* at Kanawha in 2019 and Sutherland in 2020. This is likely due to a high occurrence of mutations before insecticide was applied as the RAF was high in the pre-application sample (≥ 57.5%) and nearly equal to post-application estimate (≥ 60.7%) at these locations. Combined across all locations, the odds of the 1014F *kdr* allele being found among *A. glycines* post-application was 1.73- and 1.56-times greater compared to pre-application in 2019 and 2020, respectively. Significant differences were also predicted in RAF of M918I between pre- and post-application aphids when pooled across all locations in 2019 and 2020 (*P*-values ≤ 0.0002; Table 5), but individually only at Kanawha in 2019 and Nashua in 2020. Odds ratio for 918I allele presence in post- compared to pre-application aphids was 2.15 and 3.63-times greater across locations in 2019 and 2020, respectively. The RAF for 918I (range 2.9 to 14.8) was lower compared to that of 1014F among pre-application sampled aphids (23.2 to 82.7), as well as among post- application samples (918I: 5.3–50.0; 1014F: 50.0–77.9).

**Table 5:** Association of genotypes defining *Aphis glycines* amino acids at voltage-gated sodium channel (vgsc) positions 1014 (L1014F; kdr mutation) and 918 (M918I) with of field applied rates of lambda-cyhalothrin. Genotypes shown for *A. glycines *collected pre- and post-application of lambda-cyhalothrin, and significance of corresponding changes in resistant allele frequency (RAF) at *A. glycines* vgsc positions 1014 (1014F allele) and 918 (918I allele) in 2019 and 2020 determined using a binomial GLM.

Location	Application collection	n	Genotype (1014)^a^			RAF^b^	Model (95% CI)	Odds Ratio	*P*-value	Genotype (918)^d^			RAF	Model (95% CI)^c^	Odds Ratio	*P*-value
			L/L	L/F	F/F					M/M	M/I	I/I				
**2019**																
Boone-IA	Pre	120	51	69	0	28.8	28.7 (23.4–34.8)	2.94	< 0.0001	92	28	0	11.7	11.7 (8.1–16.3)	1.55	0.1957
	Post	47	4	35	8	54.3	54.3 (44.1–64.0)			31	16	0	17.0	17.2 (10.7–26.0)		
Kanawha-IA	Pre	120	0	102	18	57.5	57.5 (51.2–63.6)	1.14	0.4960	113	7	0	2.9	2.9 (1.4–5.9)	6.12	< 0.0001
	Post	103	1	79	23	60.7	60.7 (53.8–67.1)			71	32	0	15.5	15.5 (11.2–21.1)		
Sutherland-IA	Pre	115	25	90	0	39.1	39.1 (33.0–45.6)	1.56	0.0240	81	34	0	14.8	14.8 (10.7–19.9)	1.04	0.8908
	Post	95	0	95	0	50.0	50.0 (43.0–57.0)			66	29	0	15.3	15.2 (10.8–21.1)		
All locations	Pre	355	76	261	18	41.8	41.4 (37.7–45.1)	1.73	< 0.0001	286	69	0	9.7	8.1 (6.1–10.7)	2.15	0.0002
	Post	245	5	209	31	55.3	55.0 (50.3–59.6)			168	77	0	15.7	15.9 (12.7–19.7)		
**2020**																
Nashua-IA	Pre	110	61	47	2	23.2	23.2 (18.1–29.2)	3.34	< 0.0001	98	12	0	5.5	5.4 (3.1–9.3)	17.33	< 0.0001
	Post	38	0	38	0	50.0	50.0 (38.9–61.1)			0	38	0	50.0	50.0 (38.9–61.0)		
Sutherland-IA	Pre	110	14	10	86	82.7	82.7 (77.2–87.2)	0.736	0.2192	95	15	0	6.8	6.8 (4.1–11.0)	0.75	0.5128
	Post	95	1	40	54	77.9	77.9 (71.4–83.2)			85	10	0	5.3	5.2 (2.8–9.5)		
All locations	Pre	220	75	57	88	53.0	54.6 (48.7–60.3)	1.56	0.0174	193	27	0	6.1	6.1 (4.2–8.77)	3.63	< 0.0001
	Post	133	1	78	54	69.9	65.2 (58.6–71.3)			85	48	0	18.0	19.1 (13.7–25.8)		

^a^L/L, wild type (susceptible); L/F, *kdr* heterozygote; F/F, *kdr* homozygous resistance allele.

^b^RAF = Resistant allele frequency (((2 × RR + SR)/2n) × 100).

^c^Binomial generalized linear model with a logit link function.

^d^M/M wild type (susceptible); M/I heterozygote; I/I homozygous resistance allele.

## Discussion

We identified populations of field-collected *A. glycines* with a resistant phenotype as determined by estimating the LC_50_ using a leaf-dip bioassay. Two non-synonymous mutations in *vgsc* genes that are known to confer the knockdown resistant (*kdr*) phenotype in other insects were found in pyrethroid-resistant *A. glycines*. This phenotype has reduced sensitivities to paralysis caused by pyrethroids and DDT^52,53^, which are linked to mutations in the α subunit of the *vgsc* gene expressed in neurological tissues^12^. Insect *vgsc* genes encode four protein domains (DI–DIV), each containing six α-helical transmembrane segments (S1–S6)^54^, where S5-S6 and their linker region form the sodium pore channel. In most insects, a single *vgsc* gene encodes all functional domains, with the exception of species in the Aphididae that encode DI-DII and DIII-DIV in separate heterodimers referred to as H1 and H2, respectively^55,56^. Our cDNA evidence and prior sequence data^23^ support the presence of *A. glycines vgsc-h1* and *-h2*, where the latter is a revision of two Ag_bt1_v6 gene models into a single 958 aa *A. glycines vgsc-h2* protein encoding DIII-DIV. Multiple *vgsc* isoforms may arise via extensive alternate transcript splicing^57^ with up to six *vgsc* isoforms predicted for a given aphid species, and four isoforms for both *A. glycines vgsc-h1* and -*h2*.

The structure of *A. glycines vgsc-h1* and -*h2* heterodimers defined in this study partially differs with recent *A. glycines* transcript models^23^_,_ where the latter predicts splice variation and coding sequence that lacks homology and is not supported by comparative analyses to aphid orthologs. Specifically, N- and C-terminal coding regions of the *vgsc-h2* isoforms X1, X2 and X3 and *vgsc-h1* isoform X3, respectively, are not present among orthologs from other aphids, suggesting validation of these prior transcript models^23^ may be warranted. There may be a range of diversity in *vgsc* isoforms among *A. glycines* but we did not observe differences in splice variation between pyrethroid-resistant and susceptible *A. glycines*. Regardless, the specific nonsynonymous mutations we identified within the *vgsc* genes appear to be at least associated with, if not responsible for, resistance to pyrethroids.

The substitution mutations, L1014F and M918I, are among 61 in the *vgsc* gene found to be associated with varying levels of pyrethroid resistance in other insect species^10,58^. Our sequence data from cDNA and genomic DNA fragments of *A*. *glycines vgsc-h2* show no variation at positions orthologous to some of these other mutations (i.e. 1524, 1528, 1538, or 1549 in DIIIS6, or 1752 or 1821 in DIVS5-S6)^16^. The M918T and L925M mutations previously detected in a survey of *A. glycines*^23^ were not present in *vgsc-h1* sequences sampled in this study, which suggests that multiple genotypes may lead to the general phenotype of pyrethroid resistance. The L1014F mutation in the *M. domestica* is associated with pyrethroid resistance, but higher resistance levels were observed when it co-occurs with the M918T mutation (e.g. *super*-*kdr* M918T + L1014F genotype)^15,16^. Functional studies demonstrate resistance is conferred by the *vgsc* 918 T variant alone^59^, but the greatest resistance has been observed for the 918 T + 1014F *super-kdr* variant in the *Drosophila vgsc* (paralytic, *para*) protein^60^. Analogously, the M918I mutation is present in pyrethroid resistant tropical beg bug, *Cimex hemipterus*^61^, but only the *super-kdr* genotype (L1014F + M918I) was identified in resistance populations from Hawaii^62^ and China^63^. When considering each of these independent cases (*C. hemipterus*, *M. domestica*, and *Drosophila*), the combined evidence suggests an interdependence of mutations at 918 and 1014 positions which can produce high levels of resistance. A previous study suggested a similar phenomenon may occur with *A. glycines*. The M918I + L1014F genotype was described in five *A. glycines* from three Minnesota populations with a history of pyrethroid resistance^23^. Data reported herein suggest that the heterozygote *kdr* (L1014F) and *super-kdr* (L1014F + M918I) genotypes are associated with survival of *A. glycines* exposed to bifenthrin and lambda-cyhalothrin. We observed a significant increase in RAF for alleles encoding both 918I and 1014F, which was directly connected to more aphids with the *super*-*kdr* (918I + 1014F) genotype in field populations.

This study demonstrated the significant increase in survival among *A. glycines* with a homozygous *kdr* genotype, which partially agrees with evidence that the L1014F *kdr* mutation alone is associated with pyrethroid resistance in field-derived strains of *M. persicae*^17^, *A. gossypii*^22^_,_ and *S. avenae*^21^. Furthermore, genotypes homozygous for 1014F are more resistance than the heterozygous genotypes^14,64^. This study revealed that field-collected resistant *A. glycines* were mostly either *kdr* or *super-kdr* heterozygotes. Specifically, pyrethroid resistant phenotypes were likely conferred by the M918I + L1014F *super-kdr* genotype, but a smaller proportion were heterozygote and homozygote 1014F *kdr* genotypes. This pattern is consistent with phenotypic evidence derived from *M. domestica*^15,16^ and assays that evaluated how different mutations in the *vgsc* affect pyrethroid efficacy^60^.

There was a stronger association between our genetic markers and a resistant phenotype when aphids were screened using in-field application versus a diagnostic concentration via glass vials treated with insecticides (Table 4 and 5, respectively). These differences can be attributed to two sources of variation. The first is the concentration of insecticide that the aphids were exposed to in the glass vials compared to the field. The exposure and conditions in the vials are different compared with field conditions, given that aphids are unable to feed and are consistently exposed to the insecticide. Second, a limited number of individuals survived the glass vial assays due to likelihood that the diagnostic concentration used could have resulted in the death of a high number of resistant individuals^65,66^. This result from the bioassay may not accurately or consistently define field-resistant phenotypes. Therefore, a significant increase in the M918I + L1014F *super-kdr* genotype following a field application of pyrethroids is likely more relevant to field scenarios.

Pyrethroid resistance in *A. glycines* has been previously associated with an increase in the expression of detoxification enzymes, including cytochrome P450 monooxygenases^33,34^. When we combined synergists that inhibit detoxification enzymes to a pyrethroid, we did not observe a significant change in mortality of the SBA-MN1-2017 population (Table S4). While these results suggest that enhanced detoxification may not be involved in the pyrethroid resistance observed in SBA-MN1-2017, we did not confirm that the synergists inhibited enzyme activity. In addition, PBO is known not to inhibit all P450 monooxygenases equally^67^, which could further influence the ability to accurately evaluate the role of P450s in pyrethroid resistance*.* Future studies including companion measurements of enzyme activity and transcript expression likely will provide more definitive results.

The increased frequency of pyrethroid resistance among *A. glycines* populations in the northcentral United States^31,32^ likely evolved due to strong selection pressure from prophylactic application of insecticides with a single mode of action^68^. This resistance is an economic and environmental concern, and arguably would benefit from the implementation of IRM strategies. Specifically, IRM prescribes the application of measures and tactics that delay or prevent the onset of insecticide resistance, or mitigate the effects resistance that has developed to one class of insecticides by maintaining susceptibly to alternative control measures that remain efficacious^69^. Monitoring changes in the frequency of resistance using genetic markers is feasible^70,71^. Due to the likely involvement of multiple *vgsc* mutations in *A. glycines* resistance, markers in addition to ours for M918I and L1014F may be required to account for *vgsc*-based resistance in all populations. Incorporation of such molecular-based diagnostic data into crop management decisions and pest management strategies is yet to be fully realized. Genetic markers are arguably better suited for detecting resistance because they do not require the use of living insects and may be more efficient in processing larger sample sizes compared to using diagnostic bioassays^72^. Additional research is necessary to determine the contribution of other mechanisms and traits that confer resistance in *A. glycines* (e.g. detoxification enzyme production) alone or in association with *kdr*, *super-kdr*, or other genotypes. Despite this partial knowledge regarding the mechanism(s) of resistance, the genetic markers developed in this study are resources for estimating the frequency and tracking the spread of resistance in field populations of *A. glycines*.

## Supplementary Information

Supplementary Information.
