# Supplementary material for: Association of voltage-gated sodium channel mutations with field-evolved pyrethroid resistant phenotypes in soybean aphid and genetic markers for their detection
Source: Sci Rep. 2022 Jul 14;12:12020. doi: 10.1038/s41598-022-16366-1 (PMC9283502; doi:10.1038/s41598-022-16366-1)
Supplement: Supplementary file 1 — Supplementary Information. [file 41598_2022_16366_MOESM1_ESM.pdf]

SUPPLEMENTARY INFORMATION

Association of voltage-gated sodium channel mutations with field-evolved pyrethroid resistant phenotypes in soybean aphid and genetic markers for their detection

Ivair Valmorbida[a#], Jessica D. Hohenstein[a#], Brad S. Coates[b#], Júlia G. Bevilaqua[c], James Menger[d], Erin W. Hodgson[a], Robert L. Koch[d], and Matthew E. O'Neal[a]

[a] Department of Entomology, Iowa State University, Ames, IA, USA

[b] United States Department of Agriculture, Agricultural Research Service, Corn Insects & Crop Genetics Research Unit, Ames, IA, USA

[c] Department of Crop Protection, Universidade Federal de Santa Maria, Santa Maria, RS, Brazil

[d] Department of Entomology, University of Minnesota, Saint Paul, MN, USA

# contributed equally to the completion of this work.

Corresponding authors: Ivair Valmorbida, ivairvalmorbida@gmail.com; Matthew E. O'Neal, oneal@iastate.edu

Figure S1: Multiple nucleic acid sequence alignment among *Aphis glycines para*-type voltage-gated sodium channel heterodimer 1 (*vgsc-h1*) transcript variants. Alignments made with respect to the *A. glycines* gene model AG6007485-RA (Giordano et al. 2020) for isoforms X1 to X3 defined in sequence accession MT379843.1 (Paula et al., 2021) and isoform X4 uniquely defined among pyrethroid (lambda-cyhalothrin) susceptible (SUS; accessions MW759883.1 and MW759884.1) or resistant (RES; GenBank accessions MW75985.1 – MW759893.1) are based on leaf-dip bioassay results (IRAC, 2019) in this study (populations abbreviations defined in **Table 1**). Coding sequence translations are overlay the nucleotide alignment with structural domains I and II (D1 and DII) each comprised of six transmembrane α-helical segments (S1-6) are shown as predicted by orthology to the *Musca domestica* VGSC (GenBank accession AAB47604.1; Williamson et al., 1996; **Figure S1**), are overlaid and highlighted grey. Methionine start (ATG) and stop codons (TAG) are shown. Locations of putative splice variation in the cDNA consensus among isoforms are indicated as a plus (+). Partial cDNA sequences gaps corresponding to missing nucleotides are indicated as a dash (-). The predicted point mutations are enclosed in boxes, where those at nucleotide positions 2784 (G to A transition), and 3070 (C to T transition) cause changes at amino acid positions 918 (methionine to isoleucine; M918I) and 1014 (leucine to phenylalanine; L1014F), respectively. Mutations causing amino acid change in a homozygote and heterozygote genotypes are highlighted black, and grey, respectively. Amino acid positions with respect to *M. domestica* VGSC ortholog in *M. domestica* VGSC (Alignment with *M. domestica* VGSC in **Figure S2**). Genotype determined from corresponding electropherograms (**Figure S4**) with R = purine (co-occurring A and G) and Y = pyrimidine (C and T).

```
                                 [M  S  I  A  D  T  D  S  S  F  S  S  E  E  K  S  L  F  R  P  F  T  R  E  S  L  R  A  I  E  Q  R  I  A  E  D  H  A  K  Q   40
                               1 [         .         .         .         .         :         .         .         .         .         1         .         . 120
 1 AG6007485-RA                   ATGTCCATTGCTGACACCGATTCTTCATTCTCCAGTGAAGAAAAAAGCCTATTCCGTCCGTTCACCCGCGAATCACTGAGGGCCATCGAACAACGCATAGCTGAAGATCATGCTAAACAG
 2 MT379843.1_X1                 ATGTCCATTGCTGACACCGATTCTTCATTCTCCAGTGAAGAAAAAAGCCTATTCCGTCCGTTCACCCGCGAATCACTGAGGGCCATCGAACAACGCATAGCTGAAGATCATGCTAAACAG
 3 MT379843.1_X2                 ATGTCCATTGCTGACACCGATTCTTCATTCTCCAGTGAAGAAAAAAGCCTATTCCGTCCGTTCACCCGCGAATCACTGAGGGCCATCGAACAACGCATAGCTGAAGATCATGCTAAACAG
 4 MT379843.1_X3                 ATGTCCATTGCTGACACCGATTCTTCATTCTCCAGTGAAGAAAAAAGCCTATTCCGTCCGTTCACCCGCGAATCACTGAGGGCCATCGAACAACGCATAGCTGAAGATCATGCTAAACAG SUS
 5 MW759883.1_X4_B1B-12          ------------------------------------------------------------------------------------------------ATAGCTGAAGATCATGCTAAACAG SUS
 6 MW759884.1_X4_B3A-12          ATGTCCATTGCTGACACCGATTCTTCATTCTCCAGTGAAGAAAAAAGCCTATTCCGTCCGTTCACCCGCGAATCACTGAGGGCCATCGAACAACGCATAGCTGAAGATCATGCTAAACAG RES
 7 MW759885.1_X4_MN1-4-12        ATGTCCATTGCTGACACCGATTCTTCATTCTCCAGGGAAGAAAAAAGCCTATTCCGTCCGTTCACCCGCGAATCACTGAGGGCCATCGAACAACGCATAGCTGAAGATCATGCTAAACAG RES
 8 MW759886.1_nd_MN1-5-12        ------------------------------------------------------------------------------------------------------------------------ RES
 9 MW759887.1_X4_MN1-7-12        ATGTCCATTGCTGACACCGATTCTTCATTCTCCAGTGAAGAAAAAAGCCTATTCCGTCCGTTCACCCGCGAATCACTGAGGGCCATCGAACAACGCATAGCTGAAGATCATGCTAAACAG RES
10 MW759888.1_nd_MN2-2-12        ------------------------------------------------------------------------------------------------------------------------ RES
11 MW759889.1_X4_MN2-6-12        ATGTCCATTGCTGACACCGATTCTTCATTCTCCAGTGAAGAAAAAAGCCTATTCCGTCCGTTCACCCGCGAATCACTGAGGGCCATCGAACAACGCATAGCTGAAGATCATGCTAAACAG RES
12 MW759890.1_nd_MN2-9-12        ------------------------------------------------------------------------------------------------------------------------ RES
13 MW759892.1_X4_Suth5-12        ------------------------------------------------------------------------------------------------------------------------ RES
14 MW759893.1_X4_Suth6-12        ATGTCCATTGCTGACACCGATTCTTCATTCTCCAGTGAAGAAAAAAGCCTATTCCGTCCGTTCACCCGCGAATCACTGAGGGCCATCGAACAACGCATAGCTGAAGATCATGCTAAACAG RES


                                  K  E  L  E  E  K  R  A  E  G  E  P  L  P  W  K  R  N  K  N  K  D  V  R  Y  E  D  E  D  E  D  E  G  P  Q  P  D  A  T  L   80
                             121           .         .         :         .         .         .         .         2         .         .         .         . 240
 1 AG6007485-RA                   AAAGAACTTGAGGAAAAAAGAGCTGAAGGGGAGCCCTTGCCATGGAAGAGAAATAAAAATAAAGACGTTCGGTATGAGGACGAGGACGAAGATGAAGGTCCCCAGCCGGACGCTACTCTG
 2 MT379843.1_X1                 AAAGAACTTGAGGAAAAAAGAGCTGAAGGGGAGCCCTTGCCATGGAAGAGAAATAAAAATAAAGACGTTCGGTATGAGGACGAGGACGAAGATGAAGGTCCCCAGCCGGACGCTACTCTG
 3 MT379843.1_X2                 AAAGAACTTGAGGAAAAAAGAGCTGAAGGGGAG+++++++++++++++++++++++++++++++++GTTCGGTATGAGGACGAGGACGAAGATGAAGGTCCCCAGCCGGACGCTACTCTG
 4 MT379843.1_X3                 AAAGAACTTGAGGAAAAAAGAGCTGAAGGGGAG+++++++++++++++++++++++++++++++++GTTCGGTATGAGGACGAGGACGAAGATGAAGGTCCCCAGCCGGACGCTACTCTG
 5 MW759883.1_B1B-12             AAAGAACTTGAGGAAAAAAGAGCTGAAGGGGAG+++++++++++++++++++++++++++++++++GTTCGGTATGAGGACGAGGACGAAGATGAAGGTCCCCAGCCGGACGCTACTCTG SUS
 6 MW759884.1_B3A-12             AAAGAACTTGAGGAAAAAAGAGCTGAAGGGGAG+++++++++++++++++++++++++++++++++GTTCGGTATGAGGACGAGGACGAAGATGAAGGTCCCCAGCCGGACGCTACTCTG SUS
 7 MW759885.1_MN1-4-12           AAAGAACTTGAGGAAAAAAGAGCTGAAGGGGAG+++++++++++++++++++++++++++++++++GTTCGGTATGAGGACGAGGACGAAGATGAAGGTCCCCAGCCGGACGCTACTCTG RES
 8 MW759886.1_MN1-5-12           ------------------------------------------------------------------------------------------------------------------------ RES
 9 MW759887.1_MN1-7-12           AAAGAACTTGAGGAAAAAAGAGCTGAAGGGGAG+++++++++++++++++++++++++++++++++GTTCGGTATGAGGACGAGGACGAAGATGAAGGTCCCCAGCCGGACGCTACTCTG RES
10 MW759888.1_MN2-2-12           ------------------------------------------------------------------GTTCGGTATGAGGACGAGGACGAAGATGAAGGTCCCCAGCCGGACGCTACTCTG RESS
11 MW759889.1_MN2-6-12           AAAGAACTTGAGGAAAAAAGAGCTGAAGGGGAG+++++++++++++++++++++++++++++++++GTTCGGTATGAGGACGAGGACGAAGATGAAGGTCCCCAGCCGGACGCTACTCTG RES
12 MW759890.1_MN2-9-12           ------------------------------------------------------------------------------------------------------------------------ RES
13 MW759892.1_Suth5-12           -------------------------------------------------------------------TTCGGTATGAGGACGAGGACGAAGATGAAGGTCCCCAGCCGGACGCTACTCTG RES
14 MW759893.1_Suth6-12           AAAGAACTTGAGGAAAAAAGAGCTGAAGGGGAG+++++++++++++++++++++++++++++++++GTTCGGTATGAGGACGAGGACGAAGATGAAGGTCCCCAGCCGGACGCTACTCTG RES
```

```
                            E  Q  G  A  P  L  P  V  R  L  V  G  T  F  P  P  E  L  A  S  V  P  L  E  D  I  D  P  Y  Y  H  N  Q  K  T  F  V  V  I  S  120
                      241   .        :         .         .         .         3         .         .         .         .         :         .  360
 1 AG6007485-RA            GAACAAGGAGCTCCGCTGCCGGTCCGTTTGGTCGGCACGTTTCCACCGGAATTAGCGTCCGTACCGCTCGAGGATATCGACCCTTACTATCACAATCAAAAAACTTTTGTAGTAATTAGC
 2 MT379843.1_X1           GAACAAGGAGCTCCGCTGCCGGTCCGTTTGGTCGGCACGTTTCCACCGGAATTAGCGTCCGTACCGCTCGAGGATATCGACCCTTACTATCACAATCAAAAAACTTTTGTAGTAATTAGC
 3 MT379843.1_X2           GAACAAGGAGCTCCGCTGCCGGTCCGTTTGGTCGGCACGTTTCCACCGGAATTAGCGTCCGTACCGCTCGAGGATATCGACCCTTACTATCACAATCAAAAAACTTTTGTAGTAATTAGC
 4 MT379843.1_X3           GAACAAGGAGCTCCGCTGCCGGTCCGTTTGGTCGGCACGTTTCCACCGGAATTAGCGTCCGTACCGCTCGAGGATATCGACCCTTACTATCACAATCAAAAAACTTTTGTAGTAATTAGC
 5 MW759883.1_B1B-12       GAACAAGGAGCTCCGCTGCCGGTCCGTTTGGTCGGCACGTTTCCACCGGAATTAGCGTCCGTACCGCTCGAGGATATCGACCCTTACTATCACAATCAAAAAACTTTTGTAGTAATTAGC SUS
 6 MW759884.1_B3A-12       GAACAAGGAGCTCCGCTGCCGGTCCGTTTGGTCGGCACGTTTCCACCGGAATTAGCGTCCGTACCGCTCGAGGATATCGACCCTTACTATCACAATCAAAAAACTTTTGTAGTAATTAGC SUS
 7 MW759885.1_MN1-4-12     GAACAAGGAGCTCCGCTGCCGGTCCGTTTGGTCGGCACGTTTCCACCGGAATTAGCGTCCGTACCGCTCGAGGATATCGACCCTTACTATCACAATCAAAAAACTTTTGTAGTAATTAGC RES
 8 MW759886.1_MN1-5-12     ------------------------------------------------------------------------------------------------------------------------ RES
 9 MW759887.1_MN1-7-12     GAACAAGGAGCTCCGCTGCCGGTCCGTTTGGTCGGCACGTTTCCACCGGAATTAGCGTCCGTACCGCTCGAGGATATCGACCCTTACTATCACAATCAAAAAACTTTTGTAGTAATTAGC RES
10 MW759888.1_MN2-2-12     GAACAAGGAGCTCCGCTGCCGGTCCGTTTGGTCGGCACGTTTCCACCGGAATTAGCGTCCGTACCGCTCGAGGATATCGACCCTTACTATCACAATCAAAAAACTTTTGTAGTAATTAGC RES
11 MW759889.1_MN2-6-12     GAACAAGGAGCTCCGCTGCCGGTCCGTTTGGTCGGCACGTTTCCACCGGAATTAGCGTCCGTACCGCTCGAGGATATCGACCCTTACTATCACAATCAAAAAACTGTTGTAGTAATTAGC RES
12 MW759890.1_MN2-9-12     ------------------------------------------------------------------------------------------------------------------------ RES
13 MW759892.1_Suth5-12     GAACAAGGAGCTCCGCTGCCGGTCCGATTGGTCGGCACGTTTCCACCGGAATTAGCGTCCGTACCGCTCGAGGATATCGACCCTAACTATCACAATCAAAAAACTTTTGTAGTAATTAGC RES
14 MW759893.1_Suth6-12     GAACAAGGAGCTCCGCTGCCGGTCCGTTTGGTCGGCACGTTTCCACCGGAATTAGCGTCCGTACCGCTCGAGGATATCGACCCTTACTATCACAATCAAAAAACTTTTGTAGTAATTAGC RES

                                                                                                                 .............DI S1.............
                            K  G  K  D  I  F  R  F  S  A  T  D  G  L  W  A  L  D  P  F  N  P  I  R  R  V  A  I  Y  I  L  V  H  P  I  F  S  V  T  I  160
                      361   .        .         .         .         4         .         .         .         .         :         .         .  480
 1 AG6007485-RA            AAAGGAAAAGATATCTTCAGGTTCAGCGCCACCGATGGCCTCTGGGCACTCGACCCCTTCAACCCTATCCGCCGGGTGGCTATTTACATATTAGTCCACCCGATATTTTCTGTAACAATC
 2 MT379843.1_X1           AAAGGAAAAGATATCTTCAGGTTCAGCGCCACCGATGGCCTCTGGGCACTCGACCCCTTCAACCCTATCCGCCGGGTGGCTATTTACATATTAGTCCACCCGATATTTTCTGTAACAATC
 3 MT379843.1_X2           AAAGGAAAAGATATCTTCAGGTTCAGCGCCACCGATGGCCTCTGGGCACTCGACCCCTTCAACCCTATCCGCCGGGTGGCTATTTACATATTAGTCCACCCGATATTTTCTGTAACAATC
 4 MT379843.1_X3           AAAGGAAAAGATATCTTCAGGTTCAGCGCCACCGATGGCCTCTGGGCACTCGACCCCTTCAACCCTATCCGCCGGGTGGCTATTTACATATTAGTCCACCCGATATTTTCTGTAACAATC
 5 MW759883.1_B1B-12       AAAGGAAAAGATATCTTCAGGTTCAGCGCCACCGATGGCCTCTGGGCACTCGACCCCTTCAACCCTATCCGCCGGGTGGCTATTTACATATTAGTCCACCCGATATTTTCTGTAACAATC SUS
 6 MW759884.1_B3A-12       AAAGGAAAAGATATCTTCAGGTTCAGCGCCACCGATGGCCTCTGGGCACTCGACCCCTTCAACCCTATCCGCCGGGTGGCTATTTACATATTAGTCCACCCGATATTTTCTGTAACAATC SUS
 7 MW759885.1_MN1-4-12     AAAGGAAAAGATATCTTCAGGTTCAGCGCCACCGATGGCCTCTGGGCACTCGACCCCTTCAACCCTATCCGCCGGGTGGCTATTTACATATTAGTCCACCCGATATTTTCTGTAACAATC RES
 8 MW759886.1_MN1-5-12     ------------------------------------------------------------------------------------------------------------------------ RES
 9 MW759887.1_MN1-7-12     AAAGGAAAAGATATCTTCAGGTTCAGCGCCACCGATGGCCTCTGGGCACTCGACCCCTTCAACCCTATCCGCCGGGTGGCTATTTACATATTAGTCCACCCGATATTTTCTGTAACAATC RES
10 MW759888.1_MN2-2-12     AAAGGAAAAGATATCTTCAGGTTCAGCGCCACCGATGGCCTCTGGGCACTCGACCCCTTCAACCCTATCCGCCGGGTGGCTATTTACATATTAGTCCACCCGATATTTTCTGTAACAATC RES
11 MW759889.1_MN2-6-12     AAAGGAAAAGATATCTTCAGGTTCAGCGCCACCGATGGCCTCTGGGCACTCGACCCCTTCAACCCTATCCGCCGGGTGGCTATTTACATATTAGTCCACCCGATATTTTCTGTAACAATC RES
12 MW759890.1_MN2-9-12     ------------------------------------------------------------------------------------------------------------------------ RES
13 MW759892.1_Suth5-12     AAAGGAAAAGATATCTTCAGGTTCAGCGCCACCGATGGCCTCGGGGCACTCGACCCCTTCAACCCTATCCGCCGGGTGGCTATTTACATATTAGTCCACCCGATATTTACTGTAACAATC RES
14 MW759893.1_Suth6-12     AAAGGAAAAGATATCTTCAGGTTCAGCGCCACCGATGGCCTCTGGGCACTCGACCCCTTCAACCCTATCCGCCGGGTGGCTATTTACATATTAGTCCACCCGATATTTTCTGTAACAATC RES

                        ............DI S1.................                     ..........................DI S2..........................
                            I  T  T  I  L  T  N  C  V  F  M  I  M  P  P  T  P  T  I  E  A  S  E  V  I  F  T  G  I  Y  T  F  E  S  A  V  K  V  M  A  200
                      481   .        5         .         .         .         .         :         .         .         .         .         6  600
 1 AG6007485-RA            ATCACTACTATACTTACAAACTGTGTGTTCATGATAATGCCCCCAACTCCGACTATTGAAGCGTCTGAAGTAATATTTACCGGCATCTACACATTCGAATCGGCTGTGAAAGTAATGGCC
 2 MT379843.1_X1           ATCACTACTATACTTACAAACTGTGTGTTCATGATAATGCCCCCAACTCCGACTATTGAAGCGTCTGAAGTAATATTTACCGGCATCTACACATTCGAATCGGCTGTGAAAGTAATGGCC
 3 MT379843.1_X2           ATCACTACTATACTTACAAACTGTGT+++++++++++++++++++++++++++++++++++++++AGTAATATTTACCGGCATCTACACATTCGAATCGGCTGTGAAAGTAATGGCC
 4 MT379843.1_X3           ATCACTACTATACTTACAAACTGTGTGTTCATGATAATGCCCCCAACTCCGACTATTGAAGCGTCTGAAGTAATATTTACCGGCATCTACACATTCGAATCGGCTGTGAAAGTAATGGCC
 5 MW759883.1_B1B-12       ATCACTACTATACTTACAAACTGTGTGTTCATGATAATGCCCCCAACTCCGACTATTGAAGCGTCTGAAGTAATATTTACCGGCATCTACACATTCGAATCGGCTGTGAAAGTAATGGCC SUS
 6 MW759884.1_B3A-12       ATCACTACTATACTTACAAACTGTGTGTTCATGATAATGCCCCCAACTCCGACTATTGAAGCGTCTGAAGTAATATTTACCGGCATCTACACATTCGAATCGGCTGTGAAAGTAATGGCC SUS
 7 MW759885.1_MN1-4-12     ATCACTACTATACTTACAAACTGTGTGTTCATGATAATGCCCCCAACTCCGACTATTGAAGCGTCTGAAGTAATATTTACCGGCATCTACACATTCGAATCGGCTGTGAAAGTAATGGCC RES
 8 MW759886.1_MN1-5-12     ------------------------------------------------------------------------------------------------------------------------ RES
 9 MW759887.1_MN1-7-12     ATCACTACTATACTTACAAACTGTGTGTTCATGATAATGCCCCCAACTCCGACTATTGAAGCGTCTGAAGTAATATTTACCGGCATCTACACATTCGAATCGGCTGTGAAAGTAATGGCC RES
10 MW759888.1_MN2-2-12     ATCACTACTATACTTACAAACTGTGTGTTCATGATAATGCCCCCAACTCCGACTATTGAAGCGTCTGAAGTAATATTTACCGGCATCTACACATTCGAATCGGCTGTGAAAGTAATGGCC RES
11 MW759889.1_MN2-6-12     ATCACTACTATACTTACAAACTGTGTGTTCATGATAATGCCCCCAACTCCGACTATTGAAGCGTCTGAAGTAATATTTACCGGCATCTACACATTCGAATCGGCTGTGAAAGTAATGGCC RES
12 MW759890.1_MN2-9-12     ------------------------------------------------------------------------------------------------------------------------ RES
13 MW759892.1_Suth5-12     ATCACTACTATACTTACAAACTGTGTGTTCATGATAATGCCCCCAACTCCGAGTATTGAAGCGTCTGAAGTAATATTTACCGGCATCTACACATTCGAATCGGCTGTGAAAGTAATGGCC RES
14 MW759893.1_Suth6-12     ATCACTACTATACTTACAAACTGTGTGTTCATGATAATGCCCCCAACTCCGACTATTGAAGCGTCTGAAGTAATATTTACCGGCATCTACACATTCGAATCGGCTGTGAAAGTAATGGCC RES
```

```
                              ........................DI S3........................    .........
                              R  G  F  I  L  E  H  F  T  Y  L  R  D  A  W  N  W  L  D  F  I  V  I  A  L  A  Y  V  T  M  G  I  E  L  G  N  L  A  V  L  240
                         601           .         .         .         :         .         .         .         .         7         .         .  720
 1 AG6007485-RA           CGAGGTTTCATATTAGAACACTTCACCTATCTTAGAGATGCATGGAATTGGCTAGACTTCATTGTTATTGCATTAGCTTACGTTACTATGGGTATAGAACTTGGAAATTTAGCGGTTCTT
 2 MT379843.1_X1          CGAGGTTTCATATTAGAACACTTCACCTATCTTAGAGATGCATGGAATTGGCTAGACTTCATTGTTATTGCATTAGCTTACGTTACTATGGGTATAGAACTTGGAAATTTAGCGGTTCTT
 3 MT379843.1_X2          CGAGGTTTCATATTAGAACACTTCACCTATCTTAGAGATGCATGGAATTGGCTAGACTTCATTGTTATTGCATTAGCTTACGTTACTATGGGTATAGAACTTGGAAATTTAGCGGTTCTT
 4 MT379843.1_X3          CGAGGTTTCATATTAGAACACTTCACCTATCTTAGAGATGCATGGAATTGGCTAGACTTCATTGTTATTGCATTAGCTTACGTTACTATGGGTATAGAACTTGGAAATTTAGCGGTTCTT
 5 MW759883.1_B1B-12      CGAGGTTTCATATTAGAACACTTCACCTATCTTAGAGATGCATGGAATTGGCTAGACTTCATTGTTATTGCATTAGCTTACGTTACTATGGGTATAGAACTTGGAAATTTAGCGGTTCTT SUS
 6 MW759884.1_B3A-12      CGAGGTTTCATATTAGAACACTTCACCTATCTTAGAGATGCATGGAATTGGCTAGACTTCATTGTTATTGCATTAGCTTACGTTACTATGGGTATAGAACTTGGAAATTTAGCGGTTCTT SUS
 7 MW759885.1_MN1-4-12    CGAGGTTTCATATTAGAACACTTCACCTATCTTAGAGATGCATGGAATTGGCTAGACTTCATTGTTATTGCATTAGCTTACGTTACTATGGGTATAGAACTTGGAAATTTAGCGGTTCTT RES
 8 MW759886.1_MN1-5-12    ---------------------------------------------------------------------GCATTAGCTTACGTTACTATGGGTATAGAACTTGGAAATTTAGCGGTTCTT RES
 9 MW759887.1_MN1-7-12    CGAGGTTTCATATTAGAACACTTCACCTATCTTAGAGATGCATGGAATTGGCTAGACTTCATTGTTATTGCATTAGCTTACGTTACTATGGGTATAGAACTTGGAAATTTAGCGGTTCTT RES
10 MW759888.1_MN2-2-12    CGAGGTTTCATATTAGAACACTTCACCTATCTTAGAGATGCATGGAATTGGCTAGACTTCATTGTTATTGCATTAGCTTACGTTACTATGGGTATAGAACTTGGAAATTTAGCGGTTCTT RES
11 MW759889.1_MN2-6-12    CGAGGTTTCATATTAGAACACTTCACCTATCTTAGAGATGCATGGAATTGGCTAGACTTCATTGTTATTGCATTAGCTTACGTTACTATGGGTATAGAACTTGGAAATTTAGCGGTTCTT RES
12 MW759890.1_MN2-9-12    ---------------------------------------------------------------------GCATTAGCTTACGTTACTATGGGTATAGAACTTGGAAATTTAGCGGTTCTT RES
13 MW759892.1_Suth5-12    GGAGGTTTCATATTAGAACACTTCACCTATCTTAGAGATGCATGGAATTGGCTAGACTTCATTGTTATAGCATTAGCTTACGTTACTATGGGTATAGAACTTGGAAATTTAGCGGTTCTT RES
14 MW759893.1_Suth6-12    CGAGGTTTCATATTAGAACACTTCACCTATCTTAGAGATGCATGGAATTGGCTAGACTTCATTGTTATTGCATTAGCTTACGTTACTATGGGTATAGAACTTGGAAATTTAGCGGTTCTT RES

                          ......................DI S4.......................                                  .......DI S5.........
                              R  T  F  R  V  L  R  A  L  K  T  V  A  I  V  P  G  L  K  T  I  V  G  A  V  I  E  S  V  K  N  L  R  D  V  I  I  L  T  I  280
                         721           .         .         .         :         .         .         .         8         .         .         .  840
 1 AG6007485-RA           CGAACATTTCGAGTACTGCGAGCGCTCAAAACTGTAGCCATTGTGCCTGGATTAAAGACTATCGTTGGAGCTGTGATAGAATCCGTGAAAAACCTCAGGGATGTCATTATATTAACAATA
 2 MT379843.1_X1          CGAACATTTCGAGTACTGCGAGCGCTCAAAACTGTAGCCATTGTGCCTGGATTAAAGACTATCGTTGGAGCTGTGATAGAATCCGTGAAAAACCTCAGGGATGTCATTATATTAACAATA
 3 MT379843.1_X2          CGAACATTTCGAGTACTGCGAGCGCTCAAAACTGTAGCCATTGTGCCTGGATTAAAGACTATCGTTGGAGCTGTGATAGAATCCGTGAAAAACCTCAGGGATGTCATTATATTAACAATA
 4 MT379843.1_X3          CGAACATTTCGAGTACTGCGAGCGCTCAAAACTGTAGCCATTGTGCCTGGATTAAAGACTATCGTTGGAGCTGTGATAGAATCCGTGAAAAACCTCAGGGATGTCATTATATTAACAATA
 5 MW759883.1_B1B-12      CGAACATTTCGAGTACTGCGAGCGCTCAAAACTGTAGCCATTGTGCCTGGATTAAAGACTATCGTTGGAGCTGTGATAGAATCCGTGAAAAACCTCAGGGATGTCATTATATTAACAATA SUS
 6 MW759884.1_B3A-12      CGAACATTTCGAGTACTGCGAGCGCTCAAAACTGTAGCCATTGTGCCTGGATTAAAGACTATCGTTGGAGCTGTGATAGAATCCGTGAAAAACCTCAGGGATGTCATTATATTAACAATA SUS
 7 MW759885.1_MN1-4-12    CGAACATTTCGAGTACTGCGAGCGCTCAAAACTGTAGCCATTGTGCCTGGATTAAAGACTATCGTTGGAGCTGTGATAGAATCCGTGAAAAACCTCAGGGATGTCATTATATTAACAATA RES
 8 MW759886.1_MN1-5-12    CGAACATTTCGAGTACTGCGAGCGCTCAAAACTGTAGCCATTGTGCCTGGATTAAAGACTATCGTTGGAGCTGTGATAGAATCCGTGAAAAACCTCAGGGATGTCATTATATTAACAATA RES
 9 MW759887.1_MN1-7-12    CGAACATTTCGAGTACTGCGAGCGCTCAAAACTGTAGCCATTGTGCCTGGATTAAAGACTATCGTTGGAGCTGTGATAGAATCCGTGAAAAACCTCAGGGATGTCATTATATTAACAATA RES
10 MW759888.1_MN2-2-12    CGAACATTTCGAGTACTGCGAGCGCTCAAAACTGTAGCCATTGTGCCTGGATTAAAGACTATCGTTGGAGCTGTGATAGAATCCGTGAAAAACCTCAGGGATGTCATTATATTAACAATA RES
11 MW759889.1_MN2-6-12    CGAACATTTCGAGTACTGCGAGCGCTCAAAACTGTAGCCATTGGGCCTGGATTAAAGACTATCGTTGGAGCTGTGATAGAATCCGTGAAAAACCTCAGGGATGTCATTATATTAACAATA RES
12 MW759890.1_MN2-9-12    CGAACATTTCGAGTACTGCGAGCGCTCAAAACTGTAGCCATTGTGCCTGGATTAAAGACTATCGTTGGAGCTGTGATAGAATCCGTGAAAAACCTCAGGGATGTCATTATATTAACAATA RES
13 MW759892.1_Suth5-12    CGAACATTTCGAGTACTGCGAGCGCTCAAAACTGTAGCCATTGTGCCTGGATTAAAGACTATCGTTGGAGCTGTGATAGAATCCGTGAAAAACCTCAGGGATGTCATTATATTAACAATA RES
14 MW759893.1_Suth6-12    CGAACATTTCGAGTACTGCGAGCGCTCAAAACTGTAGCCATTGTGCCTGGATTAAAGACTATCGTTGGAGCTGTGATAGAATCCGTGAAAAACCTCAGGGATGTCATTATATTAACAATA RES

                          ............DI S5.................
                              F  S  L  S  V  F  A  L  L  G  L  Q  I  Y  M  G  V  L  T  Q  K  C  I  K  Y  F  P  L  D  G  S  A  G  N  L  T  N  E  N  W  320
                         841           :         .         .         .         .         .         9         .         .         .         :         .  960
 1 AG6007485-RA           TTTTCACTATCTGTGTTCGCATTACTGGGATTACAAATTTATATGGGCGTATTAACACAAAAATGTATAAAATATTTTCCTCTTGACGGCTCAGCTGGAAATTTAACCAATGAAAATTGG
 2 MT379843.1_X1          TTTTCACTATCTGTGTTCGCATTACTGGGATTACAAATTTATATGGGCGTATTAACACAAAAATGTATAAAATATTTTCCTCTTGACGGCTCAGCTGGAAATTTAACCAATGAAAATTGG
 3 MT379843.1_X2          TTTTCACTATCTGTGTTCGCATTACTGGGATTACAAATTTATATGGGCGTATTAACACAAAAATGTATAAAATATTTTCCTCTTGACGGCTCAGCTGGAAATTTAACCAATGAAAATTGG
 4 MT379843.1_X3          TTTTCACTATCTGTGTTCGCATTACTGGGATTACAAATTTATATGGGCGTATTAACACAAAAATGTATAAAATATTTTCCTCTTGACGGCTCAGCTGGAAATTTAACCAATGAAAATTGG
 5 MW759883.1_B1B-12      TTTTCACTATCTGTGTTCGCATTACTGGGATTACAAATTTATATGGGCGTATTAACACAAAAATGTATAAAATATTTTCCTCTTGACGGCTCAGCTGGAAATTTAACCAATGAAAATTGG SUS
 6 MW759884.1_B3A-12      TTTTCACTATCTGTGTTCGCATTACTGGGATTACAAATTTATATGGGCGTATTAACACAAAAATGTATAAAATATTTTCCTCTTGACGGCTCAGCTGGAAATTTAACCAATGAAAATTGG SUS
 7 MW759885.1_MN1-4-12    TTTTCACTATCTGTGTTCGCATTACTGGGATTACAAATTTATATGGGCGTATTAACACAAAAATGTATAAAATATTTTCCTCTTGACGGCTCAGCTGGAAATTTAACCAATGAAAATTGG RES
 8 MW759886.1_MN1-5-12    TTTTCACTATCTGTGTTCGCATTACTGGGATTACAAATTTATATGGGCGTATTAACACAAAAATGTATAAAATATTTTCCTCTTGACGGCTCAGCTGGAAATTTAACCAATGAAAATTGG RES
 9 MW759887.1_MN1-7-12    TTTTCACTATCTGTGTTCGCATTACTGGGATTACAAATTTATATGGGCGTATTAACACAAAAATGTATAAAATATTTTCCTCTTGACGGCTCAGCTGGAAATTTAACCAATGAAAATTGG RES
10 MW759888.1_MN2-2-12    TTTTCACTATCTGTGTTCGCATTACTGGGATTACAAATTTATATGGGCGTATTAACACAAAAATGTATAAAATATTTTCCTCTTGACGGCTCAGCTGGAAATTTAACCAATGAAAATTGG RES
11 MW759889.1_MN2-6-12    TTTTCACTATCTGTGTTCGCATTACTGGGATTACAAATTTATATGGGCGTATTAACACAAAAATGTATAAAATATTTTCCTCTTGACGGCTCAGCTGGAAATTTAACCAATGAAAATTGG RES
12 MW759890.1_MN2-9-12    TTTTCACTATCTGTGTTCGCATTACTGGGATTACAAATTTATATGGGCGTATTAACACAAAAATGTATAAAATATTTTCCTCTTGACGGCTCAGCTGGAAATTTAACCAATGAAAATTGG RES
13 MW759892.1_Suth5-12    TTTTCACTATCTGTGTTCGCATTACTGGGATTACAAATTTATATGGGCGTATTAACACAAAAATGTATAAAATATTTTCCTCTTGACGGCTCAGCTGGAAATTTAACCAATGAAAATTGG RES
14 MW759893.1_Suth6-12    TTTTCACTATCTGTGTTCGCATTACTGGGATTACAAATTTATATGGGCGTATTAACACAAAAATGTATAAAATATTTTCCTCTTGACGGCTCAGCTGGAAATTTAACCAATGAAAATTGG RES
```

```
                                              F  A  F  M  S  N  K  S  N  W  Q  P  N  D  D  E  P  D  E  Y  R  L  C  G  N  G  T  G  A  G  Q  C  D  E  G  Y  M  C  I  Q  360
                                       961          .           .           .           0           .           .           .           .           :           .           .           . 1080
 1 AG6007485-RA                         TTTGCGTTCATGTCGAATAAATCTAACTGGCAACCTAATGACGATGAACCAGATGAGTATCGATTATGTGGGAACGGTACTGGCGCTGGACAATGCGATGAAGGTTATATGTGTATCCAA
 2 MT379843.1_X1                        TTTGCGTTCATGTCGAATAAATCTAACTGGCAACCTAATGACGATGAACCAGATGAGTATCGATTATGTGGGAACGGTACTGGCGCTGGACAATGCGATGAAGGTTATATGTGTATCCAA
 3 MT379843.1_X2                        TTTGCGTTCATGTCGAATAAATCTAACTGGCAACCTAATGACGATGAACCAGATGAGTATCGATTATGTGGGAACGGTACTGGCGCTGGACAATGCGATGAAGGTTATATGTGTATCCAA
 4 MT379843.1_X3                        TTTGCGTTCATGTCGAATAAATCTAACTGGCAACCTAATGACGATGAACCAGATGAGTATCGATTATGTGGGAACGGTACTGGCGCTGGACAATGCGATGAAGGTTATATGTGTATCCAA
 5 MW759883.1_B1B-12                    TTTGCGTTCATGTCGAATAAATCTAACTGGCAACCTAATGACGATGAACCAGATGAGTATCGATTATGTGGGAACGGTACTGGCGCTGGACAATGCGATGAAGGTTATATGTGTATCCAA SUS
 6 MW759884.1_B3A-12                    TTTGCGTTCATGTCGAATAAATCTAACTGGCAACCTAATGACGATGAACCAGATGAGTATCGATTATGTGGGAACGGTACTGGCGCTGGACAATGCGATGAAGGTTATATGTGTATCCAA SUS
 7 MW759885.1_MN1-4-12                  TTTGCGTTCATGTCGAATAAATCTAACTGGCAACCTAATGACGATGAACCAGATGAGTATCGATTATGTGGGAACGGTACTGGCGCTGGACAATGCGATGAAGGTTATATGTGTATCCAA RES
 8 MW759886.1_MN1-5-12                  TTTGCGTTCATGTCGAATAAATCTAACTGGCAACCTAATGACGATGAACCAGATGAGTATCGATTATGTGGGAACGGTACTGGCGCTGGACAATGCGATGAAGGTTATATGTGTATCCAA RES
 9 MW759887.1_MN1-7-12                  TTTGCGTTCATGTCGAATAAATCTAACTGGCAACCTAATGACGATGAACCAGATGAGTATCGATTATGTGGGAACGGTACTGGCGCTGGACAATGCGATGAAGGTTATATGTGTATCCAA RES
10 MW759888.1_MN2-2-12                  TTTGCGTTCATGTCGAATAAATCTAACTGGCAACCTAATGACGATGAACCAGATGAGTATCGATTATGTGGGAACGGTACTGGCGCTGGACAATGCGATGAAGGTTATATGTGTATCCAA RES
11 MW759889.1_MN2-6-12                  TTTGCGTTCATGTCGAATAAATCTAACTGGCAACCTAATGACGATGAACCAGATGAGTATCGATTATGTGGGAACGGTACTGGCGCTGGACAATGCGATGAAGGTTATATGTGTATCCAA RES
12 MW759890.1_MN2-9-12                  TTTGCGTTCATGTCGAATAAATCTAACTGGCAACCTAATGACGATGAACCAGATGAGTATCGATTATGTGGGAACGGTACTGGCGCTGGACAATGCGATGAAGGTTATATGTGTATCCAA RES
13 MW759892.1_Suth5-12                  TTTGCGTTCATGTCGAATAAATCTAACTGGCAACCTAATGACGATGAACCAGATGAGTATCGATTATGTGGGAACGGTACTGGCGCTGGACAATGCGATGAAGGTTATATGTGTATCCAA RES
14 MW759893.1_Suth6-12                  TTTGCGTTCATGTCGAATAAATCTAACTGGCAACCTAATGACGATGAACCAGATGAGTATCGATTATGTGGGAACGGTACTGGCGCTGGACAATGCGATGAAGGTTATATGTGTATCCAA RES

                                              G  F  G  G  N  P  N  Y  G  Y  T  S  F  D  T  F  A  W  A  L  L  S  A  F  R  L  M  T  Q  D  N  W  E  A  L  Y  Q  Q  V  L  400
                                      1081          .           1           .           .           .           .           :           .           .           .           .           2 1200
 1 AG6007485-RA                         GGCTTTGGAGGAAATCCTAACTATGGATACACAAGTTTTGATACATTTGCGTGGGCTTTACTATCGGCATTTAGATTAATGACTCAAGACAACTGGGAAGCATTATACCAACAGGTGTTA
 2 MT379843.1_X1                        GGCTTTGGAGGAAATCCTAACTATGGATACACAAGTTTTGATACATTTGCGTGGGCTTTACTATCGGCATTTAGATTAATGACTCAAGACAACTGGGAAGCATTATACCAACAGT+++++
 3 MT379843.1_X2                        GGCTTTGGAGGAAATCCTAACTATGGATACACAAGTTTTGATACATTTGCGTGGGCTTTACTATCGGCATTTAGATTAATGACTCAAGACAACTGGGAAGCATTATACCAACAGGTGTTA
 4 MT379843.1_X3                        GGCTTTGGAGGAAATCCTAACTATGGATACACAAGTTTTGATACATTTGCGTGGGCTTTACTATCGGCATTTAGATTAATGACTCAAGACAACTGGGAAGCATTATACCAACAGGTGTTA
 5 MW759883.1_B1B-12                    GGCTTTGGAGGAAATCCTAACTATGGATACACAAGTTTTGATACATTTGCGTGGGCTTTACTATCGGCATTTAGATTAATGACTCAAGACAACTGGGAAGCATTATACCAACAGGTGTTA SUS
 6 MW759884.1_B3A-12                    GGCTTTGGAGGAAATCCTAACTATGGATACACAAGTTTTGATACATTTGCGTGGGCTTTACTATCGGCATTTAGATTAATGACTCAAGACAACTGGGAAGCATTATACCAACAGGTGTTA SUS
 7 MW759885.1_MN1-4-12                  GGCTTTGGAGGAAATCCTAACTATGGATACACAAGTTTTGATACATTTGCGTGGGCTTTACTATCGGCATTTAGATTAATGACTCAAGACAACTGGGAAGCATTATACCAACAGGTGTTA RES
 8 MW759886.1_MN1-5-12                  GGCTTTGGAGGAAATCCTAACTATGGATACACAAGTTTTGATACATTTGCGTGGGCTTTACTATCGGCATTTAGATTAATGACTCAAGACAACTGGGAAGCATTATACCAACAGGTGTTA RES
 9 MW759887.1_MN1-7-12                  GGCTTTGGAGGAAATCCTAACTATGGATACACAAGTTTTGATACATTTGCGTGGGCTTTACTATCGGCATTTAGATTAATGACTCAAGACAACTGGGAAGCATTATACCAACAGGTGTTA RES
10 MW759888.1_MN2-2-12                  GGCTTTGGAGGAAATCCTAACTATGGATACACAAGTTTTGATACATTTGCGTGGGCTTTACTATCGGCATTTAGATTAATGACTCAAGACAACTGGGAAGCATTATACCAACAGGTGTTA RES
11 MW759889.1_MN2-6-12                  GGCTTTGGAGGAAATCCTAACTATGGATACACAAGTTTTGATACATCTGCGTGGGCTTTACTATCGGCATTTAGATTAATGACTCAAGACAACTGGGAAGCATTATACCAACAGGTGTTA RES
12 MW759890.1_MN2-9-12                  GGCTTTGGAGGAAATCCTAACTATGGATACACAAGTTTTGATACATTTGCGTGGGCTTTACTATCGGCATTTAGATTAATGACTCAAGACAACTGGGAAGCATTATACCAACAGGTGTTA RES
13 MW759892.1_Suth5-12                  GGCTTTGGAGGAAATCCTAACTATGGATACACAAGTTTTGATACATTTGCGTGGGCTTTACTATCGGCATTTAGATTAATGACTCAAGACAACTGGGAAGCATTATACCAACAGGTGTTA RES
14 MW759893.1_Suth6-12                  GGCTTTGGAGGAAATCCTAACTATGGATACACAAGTTTTGATACATTTGCGTGGGCTTTACTATCGGCATTTAGATTAATGACTCAAGACAACTGGGAAGCATTATACCAACAGGTGTTA RES

                                                                   ..................................DI S6...................................
                                              R  A  A  G  P  W  H  M  F  F  F  I  V  I  I  F  L  G  S  F  Y  L  V  N  L  I  L  A  I  V  A  M  S  Y  D  E  L  Q  K  K  440
                                      1201          .           .           .           .           :           .           .           .           .           3           .           . 1320
 1 AG6007485-RA                         AGAGCTGCCGGACCATGGCATATGTTCTTCTTCATTGTGATTATATTTCTCGGTTCGTTTTATCTTGTCAATTTGATATTAGCCATCGTAGCAATGTCGTACGACGAATTGCAGAAAAAA
 2 MT379843.1_X1                        +++++++++++++++++++++++++++++++TCATTGTGATTATATTTCTCGGTTCGTTTTATCTTGTCAATTTGATATTAGCCATCGTAGCAATGTCGTACGACGAATTGCAGAAAAAA
 3 MT379843.1_X2                        AGAGCTGCCGGACCATGGCATATGTTCTTCTTCATTGTGATTATATTTCTCGGTTCGTTTTATCTTGTCAATTTGATATTAGCCATCGTAGCAATGTCGTACGACGAATTGCAGAAAAAA
 4 MT379843.1_X3                        AGAGCTGCCGGACCATGGCATATGTTCTTCTTCATTGTGATTATATTTCTCGGTTCGTTTTATCTTGTCAATTTGATATTAGCCATCGTAGCAATGTCGTACGACGAATTGCAGAAAAAA
 5 MW759883.1_B1B-12                    AGAGCTGCCGGACCATGGCATATGTTCTTCTTCATTGTGATTATATTTCTCGGTTCGTTTTATCTTGTCAATTTGATATTAGCCATCGTAGCAATGTCGTACGACGAATTGCAGAAAAAA SUS
 6 MW759884.1_B3A-12                    AGAGCTGCCGGACCATGGCATATGTTCTTCTTCATTGTGATTATATTTCTCGGTTCGTTTTATCTTGTCAATTTGATATTAGCCATCGTAGCAATGTCGTACGACGAATTGCAGAAAAAA SUS
 7 MW759885.1_MN1-4-12                  AGAGCTGCCGGACCATGGCATATGTTCTTCTTCATTGTGATTATATTTCTCGGTTCGTTTTATCTTGTCAATTTGATATTAGCCATCGTAGCAATGTCGTACGACGAATTGCAGAAAAAA RES
 8 MW759886.1_MN1-5-12                  AGAGCTGCCGGACCATGGCATATGTTCTTCTTCATTGTGATTATATTTCTCGGTTCGTTTTATCTTGTCAATTTGATATTAGCCATCGTAGCAATGTCGTACGACGAATTGCAGAAAAAA RES
 9 MW759887.1_MN1-7-12                  AGAGCTGCCGGACCATGGCATATGTTCTTCTTCATTGTGATTATATTTCTCGGTTCGTTTTATCTTGTCAATTTGATATTAGCCATCGTAGCAATGTCGTACGACGAATTGCAGAAAAAA RES
10 MW759888.1_MN2-2-12                  AGAGCTGCCGGACCATGGCATATGTTCTTCTTCATTGTGATTATATTTCTCGGTTCGTTTTATCTTGTCAATTTGATATTAGCCATCGTAGCAATGTCGTACGACGAATTGCAGAAAAAA RES
11 MW759889.1_MN2-6-12                  AGAGCTGCCGGACCATGGCATATGTTCTTCTTCATTGTGATTATATTTCTCGGTTCGTTTTATCTTGTCAATTTGATATTAGCCATCGTAGCAATGTCGTACGACGAATTGCAGAAAAAA RES
12 MW759890.1_MN2-9-12                  AGAGCTGCCGGACCATGGCATATGTTCTTCTTCATTGTGATTATATTTCTCGGTTCGTTTTATCTTGTCAATTTGATATTAGCCATCGTAGCAATGTCGTACGACGAATTGCAGAAAAAA RES
13 MW759892.1_Suth5-12                  AGAGCTGCCGGACCATGGCATATGTTCTTCTTCATTGTGATTATATTTCTCGGTTCGTTTTATCTTGTCAATTTGATATTAGCCATCGTAGCAATGTCGTACGACGAATTGCAGAAAAAA RES
14 MW759893.1_Suth6-12                  AGAGCTGCCGGACCATGGCATATGTTCTTCTTCATTGTGATTATATTTCTCGGTTCGTTTTATCTTGTCAATTTGATATTAGCCATCGTAGCAATGTCGTACGACGAATTGCAGAAAAAA RES
```

```
                           A  E  E  E  E  A  A  E  E  E  A  I  R  E  A  E  Q  A  A  K  D  R  E  V  R  R  Q  A  H  E  E  R  V  A  E  R  A  E  R  A   480
                    1321            .          .          :          .          .          .          .          4          .          .          .          . 1440
 1 AG6007485-RA            GCTGAAGAAGAAGAGGCAGCCGAAGAAGAAGCTATCAGGGAAGCGGAACAAGCGGCTAAAGACAGAGAGGTCCGGAGGCAAGCTCACGAGGAAAGAGTGGCGGAACGTGCGGAGAGAGCG
 2 MT379843.1_X1           GCTGAAGAAGAAGAGGCAGCCGAAGAAGAAGCTATCAGGGAAGCGGAACAAGCGGCTAAAGACAGAGAGGTCCGGAGGCAAGCTCACGAGGAAAGAGTGGCGGAACGTGCGGAGAGAGCG
 3 MT379843.1_X2           GCTGAAGAAGAAGAGGCAGCCGAAGAAGAAGCTATCAGGGAAGCGGAACAAGCGGCTAAAGACAGAGAGGTCCGGAGGCAAGCTCACGAGGAAAGAGTGGCGGAACGTGCGGAGAGAGCG
 4 MT379843.1_X3           GCTGAAGAAGAAGAGGCAGCCGAAGAAGAAGCTATCAGGGAAGCGGAACAAGCGGCTAAAGACAGAGAGGTCCGGAGGCAAGCTCACGAGGAAAGAGTGGCGGAACGTGCGGAGAGAGCG
 5 MW759883.1_B1B-12       GCTGAAGAAGAAGAGGCAGCCGAAGAAGAAGCTATCAGGGAAGCGGAACAAGCGGCTAAAGACAGAGAGGTCCGGAGGCAAGCTCACGAGGAAAGAGTGGCGGAACGTGCGGAGAGAGCG SUS
 6 MW759884.1_B3A-12       GCTGAAGAAGAAGAGGCAGCCGAAGAAGAAGCTATCAGGGAAGCGGAACAAGCGGCTAAAGACAGAGAGGTCCGGAGGCAAGCTCACGAGGAAAGAGTGGCGGAACGTGCGGAGAGAGCG SUS
 7 MW759885.1_MN1-4-12     GCTGAAGAAGAAGAGGCAGCCGAAGAAGAAGCTATCAGGGAAGCGGAACAAGCGGCTAAAGACAGAGAGGTCCGGAGGCAAGCTCACGAGGAAAGAGTGGCGGAACGTGCGGAGAGAGCG RES
 8 MW759886.1_MN1-5-12     GCTGAAGAAGAAGAGGCAGCCGAAGAAGAAGCTATCAGGGAAGCGGAACAAGCGGCTAAAGACAGAGAGGTCCGGAGGCAAGCTCACGAGGAAAGAGTGGCGGAACGTGCGGAGAGAGCG RES
 9 MW759887.1_MN1-7-12     GCTGAAGAAGAAGAGGCAGCCGAAGAAGAAGCTATCAGGGAAGCGGAACAAGCGGCTAAAGACAGAGAGGTCCGGAGGCAAGCTCACGAGGAAAGAGTGGCGGAACGTGCGGAGAGAGCG RES
10 MW759888.1_MN2-2-12     GCTGAAGAAGAAGAGGCAGCCGAAGAAGAAGCTATCAGGGAAGCGGAACAAGCGGCTAAAGACAGAGAGGTCCGGAGGCAAGCTCACGAGGAAAGAGTGGCGGAACGTGCGGAGAGAGCG RES
11 MW759889.1_MN2-6-12     GCTGAAGAAGAAGAGGCAGCCGAAGAAGAAGCTATCAGGGAAGCGGAACAAGCGGCTAAAGACAGAGAGGTCCGGAGGCAAGCTCACGAGGAAAGAGTGGCGGAACGTGCGGAGAGAGCG RES
12 MW759890.1_MN2-9-12     GCTGAAGAAGAAGAGGCAGCCGAAGAAGAAGCTATCAGGGAAGCGGAACAAGCGGCTAAAGACAGAGAGGTCCGGAGGCAAGCTCACGAGGAAAGAGTGGCGGAACGTGCGGAGAGAGCG RES
13 MW759892.1_Suth5-12     GCTGAAGAAGAAGAGGCAGCCGAAGAAGAAGCTATCAGGGAAGCGGAACAAGCGGCTAAAGACAGAGAGGTCCGGAGGCAAGCTCACGAGGAAAGAGTGGCGGAACGTGCGGAGAGAGCG RES
14 MW759893.1_Suth6-12     GCTGAAGAAGAAGAGGCAGCCGAAGAAGAAGCTATCAGGGAAGCGGAACAAGCGGCTAAAGACAGAGAGGTCCGGAGGCAAGCTCACGAGGAAAGAGTGGCGGAACGTGCGGAGAGAGCG RES

                           R  H  V  T  Q  H  P  K  S  P  S  D  F  S  S  Q  S  Y  D  N  M  F  G  G  G  Q  D  R  G  I  G  N  D  H  H  R  E  K  N  M   520
                    1441            :          .          .          .          .          5          .          .          .          .          :          . 1560
 1 AG6007485-RA            CGACACGTCACCCAACATCCGAAATCGCCTTCGGACTTTTCGAGCCAAAGTTACGACAATATGTTTGGCGGTGGTCAAGACCGAGGGATTGGCAATGATCATCATAGAGAGAAAAATATG
 2 MT379843.1_X1           CGACACGTCACCCAACATCCGAAATCGCCTTCGGACTTTTCGAGCCAAAGTTACGACAATATGTTTGGCGGTGGTCAAGACCGAGGGATTGGCAATGATCATCATAGAGAGAAAAATATG
 3 MT379843.1_X2           CGACACGTCACCCAACATCCGAAATCGCCTTCGGACTTTTCGAGCCAAAGTTACGACAATATGTTTGGCGGTGGTCAAGACCGAGGGATTGGCAATGATCATCATAGAGAGAAAAATATG
 4 MT379843.1_X3           CGACACGTCACCCAACATCCGAAATCGCCTTCGGACTTTTCGAGCCAAAGTTACGACAATATGTTTGGCGGTGGTCAAGACCGAGGGATTGGCAATGATCATCATAGAGAGAAAAATATG
 5 MW759883.1_B1B-12       CGACACGTCACCCAACATCCGAAATCGCCTTCGGACTTTTCGAGCCAAAGTTACGACAATATGTTTGGCGGTGGTCAAGACCGAGGGATTGGCAATGATCATCATAGAGAGAAAAATATG SUS
 6 MW759884.1_B3A-12       CGACACGTCACCCAACATCCGAAATCGCCTTCGGACTTTTCGAGCCAAAGTTACGACAATATGTTTGGCGGTGGTCAAGACCGAGGGATTGGCAATGATCATCATAGAGAGAAAAATATG SUS
 7 MW759885.1_MN1-4-12     CGACACGTCACCCAACATCCGAAATCGCCTTCGGACTTTTCGAGCCAAAGTTACGACAATATGTTTGGCGGTGGTCAAGACCGAGGGATTGGCAATGATCATCATAGAGAGAAAAATATG RES
 8 MW759886.1_MN1-5-12     CGACACGTCACCCAACATCCGAAATCGCCTTCGGACTTTTCGAGCCAAAGTTACGACAATATGTTTGGCGGTGGTCAAGACCGAGGGATTGGCAATGATCATCATAGAGAGAAAAATATG RES
 9 MW759887.1_MN1-7-12     CGACACGTCACCCAACATCCGAAATCGCCTTCGGACTTTTCGAGCCAAAGTTACGACAATATGTTTGGCGGTGGTCAAGACCGAGGGATTGGCAATGATCATCATAGAGAGAAAAATATG RES
10 MW759888.1_MN2-2-12     CGACACGTCACCCAACATCCGAAATCGCCTTCGGACTTTTCGAGCCAAAGTTACGACAATATGTTTGGCGGTGGTCAAGACCGAGGGATTGGCAATGATCATCATAGAGAGAAAAATATG RES
11 MW759889.1_MN2-6-12     CGACACGTCACCCAACATCCGAAATCGCCTTCGGACTTTTCGAGCCAAAGTTACGACAATATGTTTGGCGGTGGTCAAGACCGAGGGATTGGCAATGATCATCATAGAGAGAAAAATATG RES
12 MW759890.1_MN2-9-12     CGACACGTCACCCAACATCCGAAATCGCCTTCGGACTTTTCGAGCCAAAGTTACGACAATATGTTTGGCGGTGGTCAAGACCGAGGGATTGGCAATGATCATCATAGAGAGAAAAATATG RES
13 MW759892.1_Suth5-12     CGACACGTCACCCAACATCCGAAATCGCCTTCGGACTTTTCGAGCCAAAGTTACGACAATATGTTTGGCGGTGGTCAAGACCGAGGGATTGGCAATGATCATCATAGAGAGAAAAATATG RES
14 MW759893.1_Suth6-12     CGACACGTCACCCAACATCCGAAATCGCCTTCGGACTTTTCGAGCCAAAGTTACGACAATATGTTTGGCGGTGGTCAAGACCGAGGGATTGGCAATGATCATCATAGAGAGAAAAATATG RES

                           S  L  R  S  M  S  I  T  S  H  D  K  H  S  D  T  G  S  V  D  R  Q  S  G  K  T  R  K  A  S  L  S  L  P  G  S  P  F  N  I   560
                    1561            .          .          .          6          .          .          .          .          :          .          .          . 1680
 1 AG6007485-RA            AGCCTGCGATCGATGTCGATCACGAGCCATGACAAACATTCGGACACCGGGAGTGTGGATAGGCAGAGCGGCAAAACAAGAAAAGCAAGCCTCAGCCTGCCGGGATCGCCGTTCAACATT
 2 MT379843.1_X1           AGCCTGCGATCGATGTCGATCACGAGCCATGACAAACATTCGGACACCGGGAGTGTGGATAGGCAGAGCGGCAAAACAAGAAAAGCAAGCCTCAGCCTGCCGGGATCGCCGTTCAACATT
 3 MT379843.1_X2           AGCCTGCGATCGATGTCGATCACGAGCCATGACAAACATTCGGACACCGGGAGTGTGGATAGGCAGAGCGGCAAAACAAGAAAAGCAAGCCTCAGCCTGCCGGGATCGCCGTTCAACATT
 4 MT379843.1_X3           AGCCTGCGATCGATGTCGATCACGAGCCATGACAAACATTCGGACACCGGGAGTGTGGATAGGCAGAGCGGCAAAACAAGAAAAGCAAGCCTCAGCCTGCCGGGATCGCCGTTCAACATT
 5 MW759883.1_B1B-12       AGCCTGCGATCGATGTCGATCACGAGCCATGACAAACATTCGGACACCGGGAGTGTGGATAGGCAGAGCGGCAAAACAAGAAAAGCAAGCCTCAGCCTGCCGGGATCGCCGTTCAACATT SUS
 6 MW759884.1_B3A-12       AGCCTGCGATCGATGTCGATCACGAGCCATGACAAACATTCGGACACCGGGAGTGTGGATAGGCAGAGCGGCAAAACAAGAAAAGCAAGCCTCAGCCTGCCGGGATCGCCGTTCAACATT SUS
 7 MW759885.1_MN1-4-12     AGCCTGCGATCGATGTCGATCACGAGCCATGACAAACATTCGGACACCGGGAGTGTGGATAGGCAGAGCGGCAAAACAAGAAAAGCAAGCCTCAGCCTGCCGGGATCGCCGTTCAACATT RES
 8 MW759886.1_MN1-5-12     AGCCTGCGATCGATGTCGATCACGAGCCATGACAAACATTCGGACACCGGGAGTGTGGATAGGCAGAGCGGCAAAACAAGAAAAGCAAGCCTCAGCCTGCCGGGATCGCCGTTAAACATT RES
 9 MW759887.1_MN1-7-12     AGCCTGCGATCGATGTCGATCACGAGCCATGACAAACATTCGGACACCGGGAGTGTGGATAGGCAGAGCGGCAAAACAAGAAAAGCAAGCCTCAGCCTGCCGGGATCGCCGTTCAACATT RES
10 MW759888.1_MN2-2-12     AGCCTGCGATCGATGTCGATCACGAGCCATGACAAACATTCGGACACCGGGAGTGTGGATAGGCAGAGCGGCAAAACAAGAAAAGCAAGCCTCAGCCTGCCGGGATCGCCGTTCAACATT RES
11 MW759889.1_MN2-6-12     AGCCTGCGATCGATGTCGATCACGAGCCATGACAAACATTCGGACACCGGGAGTGTGGATAGGCAGAGCGGCAAAACAAGAAAAGCAAGCCTCAGCCTGCCGGGATCGCCGTTCAACATT RES
12 MW759890.1_MN2-9-12     AGCCTGCGATCGATGTCGATCACGAGCCATGACAAACATTCGGACACCGGGAGTGTGGATAGGCAGAGCGGCAAAACAAGAAAAGCAAGCCTCAGCCTGCCGGGATCGCCGTTCAACATT RES
13 MW759892.1_Suth5-12     AGCCTGCGATCGATGTCGATCACGAGCCATGACAAACATTCGGACACCGGGAGTGTGGATAGGCAGAGCGGCAAAACAAGAAAAGCAAGCCTCAGCCTGCCGGGATCGCCGTTCAACATT RES
14 MW759893.1_Suth6-12     AGCCTGCGATCGATGTCGATCACGAGCCATGACAAACATTCGGACACCGGGAGTGTGGATAGGCAGAGCGGCAAAACAAGAAAAGCAAGCCTCAGCCTGCCGGGATCGCCGTTCAACATT RES
```

```
                                 R  R  A  S  R  G  S  H  Q  L  S  H  R  N  G  R  P  R  F  T  G  A  D  T  K  P  L  V  L  N  T  L  L  D  A  E  E  H  L  P   600
                             1681      .        7        .         .         .         .         :         .         .         .         .        8 1800
 1 AG6007485-RA                   CGGCGGGCGTCGCGTGGCAGTCACCAACTGTCACATCGGAACGGTAGACCGAGGTTTACCGGAGCGGATACCAAGCCGTTAGTCTTAAACACGTTACTTGACGCGGAAGAACATTTACCG
 2 MT379843.1_X1                  CGGCGGGCGTCGCGTGGCAGTCACCAACTGTCACATCGGAACGGTAGACCGAGGTTTACCGGAGCGGATACCAAGCCGTTAGTCTTAAACACGTTACTTGACGCGGAAGAACATTTACCG
 3 MT379843.1_X2                  CGGCGGGCGTCGCGTGGCAGTCACCAACTGTCACATCGGAACGGTAGACCGAGGTTTACCGGAGCGGATACCAAGCCGTTAGTCTTAAACACGTTACTTGACGCGGAAGAACATTTACCG
 4 MT379843.1_X3                  CGGCGGGCGTCGCGTGGCAGTCACCAACTGTCACATCGGAACGGTAGACCGAGGTTTACCGGAGCGGATACCAAGCCGTTAGTCTTAAACACGTTACTTGACGCGGAAGAACATTTACCG
 5 MW759883.1_B1B-12              CGGCGGGCGTCGCGTGGCAGTCACCAACTGTCACATCGGAACGGTAGACCGAGGTTTACCGGAGCGGATACCAAGCCGTTAGTCTTAAACACGTTACTTGACGCGGAAGAACATTTACCG SUS
 6 MW759884.1_B3A-12              CGGCGGGCGTCGCGTGGCAGTCACCAACTGTCACATCGGAACGGTAGACCGAGGTTTACCGGAGCGGATACCAAGCCGTTAGTCTTAAACACGTTACTTGACGCGGAAGAACATTTACCG SUS
 7 MW759885.1_MN1-4-12            CGGCGGGCGTCGCGTGGCAGTCACCAACTGTCACATCGGAACGGTAGACCGAGGTTTACCGGAGCGGATACCAAGCCGTTAGTCTTAAACACGTTACTTGACGCGGAAGAACATTTACCG RES
 8 MW759886.1_MN1-5-12            CGGCGGGCGTCGCGTGGCAGTCACCAACTGTCACATCGGAACGGTAGACCGAGGTTTACCGGAGCGGATACCAAGCCGTTAGTCTTAAACACGTTACTTGACGCGGAAGAACATTTACCG RES
 9 MW759887.1_MN1-7-12            CGGCGGGCGTCGCGTGGCAGTCACCAACTGTCACATCGGAACGGTAGACCGAGGTTTACCGGAGCGGATACCAAGCCGTTAGTCTTAAACACGTTACTTGACGCGGAAGAACATTTACCG RES
10 MW759888.1_MN2-2-12            CGGCGGGCGTCGCGTGGCAGTCACCAACTGTCACATCGGAACGGTAGACCGAGGTTTACCGGAGCGGATACCAAGCCGTTAGTCTTAAACACGTTACTTGACGCGGAAGAACATTTACCG RES
11 MW759889.1_MN2-6-12            CGGCGGGCGTCGCGTGGCAGTCACCAACTGTCACATCGGAACGGTAGACCGAGGTTTACCGGAGCGGATACCAAGCCGTTAGTCTTAAACACGTTACTTGACGCGGAAGAACATTTACCG RES
12 MW759890.1_MN2-9-12            CGGCGGGCGTCGCGTGGCAGTCACCAACTGTCACATCGGAACGGTAGACCGAGGTTTACCGGAGCGGATACCAAGCCGTTAGTCTTAAACACGTTACTTGACGCGGAAGAACATTTACCG RES
13 MW759892.1_Suth5-12            CGGCGGGCGTCGCGTGGCAGTCACCAACTGTCACATCGGAACGGTAGACCGAGGTTTACCGGAGCGGATACCAAGCCGTTAGTCTTAAACACGTTACTTGACGCGGAAGAACATTTACCG RES
14 MW759893.1_Suth6-12            CGGCGGGCGTCGCGTGGCAGTCACCAACTGTCACATCGGAACGGTAGACCGAGGTTTACCGGAGCGGATACCAAGCCGTTAGTCTTAAACACGTTACTTGACGCGGAAGAACATTTACCG RES

                                 Y  A  D  D  S  N  A  V  T  P  M  S  E  E  N  G  A  I  I  V  P  V  S  Y  A  N  F  G  S  R  H  S  S  Y  T  S  H  T  S  R   640
                             1801      .         .         .         .         :         .         .         .         .        9         .        . 1920
 1 AG6007485-RA                   TATGCCGACGACTCAAACGCAGTGACGCCCATGAGTGAGGAGAACGGTGCCATCATCGTACCCGTATCATACGCGAACTTCGGTTCGAGGCACAGCAGTTACACGTCACACACGTCCCGA
 2 MT379843.1_X1                  TATGCCGACGACTCAAACGCAGTGACGCCCATGAGTGAGGAGAACGGTGCCATCATCGTACCCGTATCATACGCGAACTTCGGTTCGAGGCACAGCAGTTACACGTCACACACGTCCCGA
 3 MT379843.1_X2                  TATGCCGACGACTCAAACGCAGTGACGCCCATGAGTGAGGAGAACGGTGCCATCATCGTACCCGTATCATACGCGAACTTCGGTTCGAGGCACAGCAGTTACACGTCACACACGTCCCGA
 4 MT379843.1_X3                  TATGCCGACGACTCAAACGCAGTGACGCCCATGAGTGAGGAGAACGGTGCCATCATCGTACCCGTATCATACGCGAACTTCGGTTCGAGGCACAGCAGTTACACGTCACACACGTCCCGA
 5 MW759883.1_B1B-12              TATGCCGACGACTCAAACGCAGTGACGCCCATGAGTGAGGAGAACGGTGCCATCATCGTACCCGTATCATACGCGAACTTCGGTTCGAGGCACAGCAGTTACACGTCACACACGTCCCGA SUS
 6 MW759884.1_B3A-12              TATGCCGACGACTCAAACGCAGTGACGCCCATGAGTGAGGAGAACGGTGCCATCATCGTACCCGTATCATACGCGAACTTCGGTTCGAGGCACAGCAGTTACACGTCACACACGTCCCGA SUS
 7 MW759885.1_MN1-4-12            TATGCCGACGACTCAAACGCAGTGACGCCCATGAGTGAGGAGAACGGTGCCATCATCGTACCCGTATCATACGCGAACTTCGGTTCGAGGCACAGCAGTTACACGTCACACACGTCCCGA RES
 8 MW759886.1_MN1-5-12            TATGCCGACGACTCAAACGCAGTGACGCCCATGAGTGAGGAGAACGGTGCCATCATCGTACCCGTATCATACGCGAACTTCGGTTCGAGGCACAGCAGTTACACGTCACACACGTCCCGA RES
 9 MW759887.1_MN1-7-12            TATGCCGACGACTCAAACGCAGTGACGCCCATGAGTGAGGAGAACGGTGCCATCATCGTACCCGTATCATACGCGAACTTCGGTTCGAGGCACAGCAGTTACACGTCACACACGTCCCGA RES
10 MW759888.1_MN2-2-12            TATGCCGACGACTCAAACGCAGTGACGCCCATGAGTGAGGAGAACGGTGCCATCATCGTACCCGTATCATACGCGAACTTCGGTTCGAGGCACAGCAGTTACACGTCACACACGTCCCGA RES
11 MW759889.1_MN2-6-12            TATGCCGACGACTCAAACGCAGTGACGCCCATGAGTGAGGAGAACGGTGCCATCATCGTACCCGTATCATACGCGAACTTCGGTTCGAGGCACAGCAGTTACACGTCACACACGTCCCGA RES
12 MW759890.1_MN2-9-12            TATGCCGACGACTCAAACGCAGTGACGCCCATGAGTGAGGAGAACGGTGCCATCATCGTACCCGTATCATACGCGAACTTCGGTTCGAGGCACAGCAGTTACACGTCACACACGTCCCGA RES
13 MW759892.1_Suth5-12            TATGCCGACGACTCAAACGCAGTGACGCCCATGAGTGAGGAGAACGGTGCCATCATCGTACCCGTATCATACGCGAACTTCGGTTCGAGGCACAGCAGTTACACGTCACACACGTCCCGA RES
14 MW759893.1_Suth6-12            TATGCCGACGACTCAAACGCAGTGACGCCCATGAGTGAGGAGAACGGTGCCATCATCGTACCCGTATCATACGCGAACTTCGGTTCGAGGCACAGCAGTTACACGTCACACACGTCCCGA RES

                                 I  T  Y  T  S  H  A  D  L  F  K  P  P  M  T  K  E  R  Q  L  R  S  R  S  A  R  N  Y  F  N  P  S  D  Q  R  Y  H  R  D  D   680
 1 AG6007485-RA                   ATTACGTATACGTCGCATGCGGATCTGTTCAAACCGCCTATGACCAAAGAGAGGCAGTTGAGGTCGCGGTCGGCCAGAAACTATTTCAATCCATCTGATCAGAGATACCATCGAGACGAC
 2 MT379843.1_X1                  ATTACGTATACGTCGCATGCGGATCTGTTCAAACCGCCTATGACCAAAGAGAGGCAGTTGAGGTCGCGGTCGGCCAGAAACTATTTCAATCCATCTGATCAGAGATACCATCGAGACGAC
 3 MT379843.1_X2                  ATTACGTATACGTCGCATGCGGATCTGTTCAAACCGCCTATGACCAAAGAGAGGCAGTTGAGGTCGCGGTCGGCCAGAAACTATTTCAATCCATCTGATCAGAGATACCATCGAGACGAC
 4 MT379843.1_X3                  ATTACGTATACGTCGCATGCGGATCTGTTCAAACCGCCTATGACCAAAGAGAGGCAGTTGAGGTCGCGGTCGGCCAGAAACTATTTCAATCCATCTGATCAGAGATACCATCGAGACGAC
 5 MW759883.1_B1B-12              ATTACGTATACGTCGCATGCGGATCTGTTCAAACCGCCTATGACCAAAGAGAGGCAGTTGAGGTCGCGGTCGGCCAGAAACTATTTCAATCCATCTGATCAGAGATACCATCGAGACGAC SUS
 6 MW759884.1_B3A-12              ATTACGTATACGTCGCATGCGGATCTGTTCAAACCGCCTATGACCAAAGAGAGGCAGTTGAGGTCGCGGTCGGCCAGAAACTATTTCAATCCATCTGATCAGAGATACCATCGAGACGAC SUS
 7 MW759885.1_MN1-4-12            ATTACGTATACGTCGCATGCGGATCTGTTCAAACCGCCTATGACCAAAGAGAGGCAGTTGAGGTCGCGGTCGGCCAGAAACTATTTCAATCCATCTGATCAGAGATACCATCGAGACGAC RES
 8 MW759886.1_MN1-5-12            ATTACGTATACGTCGCATGCGGATCTGTTCAAACCGCCTATGACCAAAGAGAGGCAGTTGAGGTCGCGGTCGGCCAGAAACTATTTCAATCCATCTGATCAGAGATACCATCGAGACGAC RES
 9 MW759887.1_MN1-7-12            ATTACGTATACGTCGCATGCGGATCTGTTCAAACCGCCTATGACCAAAGAGAGGCAGTTGAGGTCGCGGTCGGCCAGAAACTATTTCAATCCATCTGATCAGAGATACCATCGAGACGAC RES
10 MW759888.1_MN2-2-12            ATTACGTATACGTCGCATGCGGATCTGTTCAAACCGCCTATGACCAAAGAGAGGCAGTTGAGGTCGCGGTCGGCCAGAAACTATTTCAATCCATCTGATCAGAGATACCATCGAGACGAC RES
11 MW759889.1_MN2-6-12            ATTACGTATACGTCGCATGCGGATCTGTTCAAACCGCCTATGACCAAAGAGAGGCAGTTGAGGTCGCGGTCGGCCAGAAACTATTTCAATCCATCTGATCAGAGATACCATCGAGACGAC RES
12 MW759890.1_MN2-9-12            ATTACGTATACGTCGCATGCGGATCTGTTCAAACCGCCTATGACCAAAGAGAGGCAGTTGAGGTCGCGGTCGGCCAGAAACTATTTCAATCCATCTGATCAGAGATACCATCGAGACGAC RES
13 MW759892.1_Suth5-12            ATTACGTATACGTCGCATGCGGATCTGTTCAAACCGCCTATGACCAAAGAGAGGCAGTTGAGGTCGCGGTCGGCCAGAAACTATTTCAATCCATCTGATCAGAGATACCATCGAGACGAC RES
14 MW759893.1_Suth6-12            ATTACGTATACGTCGCATGCGGATCTGTTCAAACCGCCTATGACCAAAGAGAGGCAGTTGAGGTCGCGGTCGGCCAGAAACTATTTCAATCCATCTGATCAGAGATACCATCGAGACGAC RES
```

```
                            D  Y  D  T  S  S  M  S  K  S  K  Q  K  V  D  E  C  G  Y  S  D  S  Q  K  H  T  V  V  D  M  R  D  V  M  V  L  N  D  I  I  720
                       2041          :         .         .         .         .         1         .         .         .         .         :         . 2160
 1 AG6007485-RA            GATTACGATACGAGTTCCATGTCCAAATCGAAACAAAAAGTGGACGAGTGCGGTTACAGTGATTCACAAAAGCACACGGTCGTCGACATGAGAGACGTTATGGTGTTGAACGATATCATC
 2 MT379843.1_X1           GATTACGATACGAGTTCCATGTCCAAATCGAAACAAAAAGTGGACGAGTGCGGTTACAGTGATTCACAAAAGCACACGGTCGTCGACATGAGAGACGTTATGGTGTTGAACGATATCATC
 3 MT379843.1_X2           GATTACGATACGAGTTCCATGTCCAAATCGAAACAAAAAGTGGACGAGTGCGGTTACAGTGATTCACAAAAGCACACGGTCGTCGACATGAGAGACGTTATGGTGTTGAACGATATCATC
 4 MT379843.1_X3           GATTACGATACGAGTTCCATGTCCAAATCGAAACAAAAAGTGGACGAGTGCGGTTACAGTGATTCACAAAAGCACACGGTCGTCGACATGAGAGACGTTATGGTGTTGAACGATATCATC
 5 MW759883.1_B1B-12       GATTACGATACGAGTTCCATGTCCAAATCGAAACAAAAAGTGGACGAGTGCGGTTACAGTGATTCACAAAAGCACACGGTCGTCGACATGAGAGACGTTATGGTGTTGAACGATATCATC SUS
 6 MW759884.1_B3A-12       GATTACGATACGAGTTCCATGTCCAAATCGAAACAAAAAGTGGACGAGTGCGGTTACAGTGATTCACAAAAGCACACGGTCGTCGACATGAGAGACGTTATGGTGTTGAACGATATCATC SUS
 7 MW759885.1_MN1-4-12     GATTACGATACGAGTTCCATGTCCAAATCGAAACAAAAAGTGGACGAGTGCGGTTACAGTGATTCACAAAAGCACACGGTCGTCGACATGAGAGACGTTATGGTGTTGAACGATATCATC RES
 8 MW759886.1_MN1-5-12     GATTACGATACGAGTTCCATGTCCAAATCGAAACAAAAAGTGGACGAGTGCGGTTACAGTGATTCACAAAAGCACACGGTCGTCGACATGAGAGACGTTATGGTGTTGAACGATATCATC RES
 9 MW759887.1_MN1-7-12     GATTACGATACGAGTTCCATGTCCAAATCGAAACAAAAAGTGGACGAGTGCGGTTACAGTGATTCACAAAAGCACACGGTCGTCGACATGAGAGACGTTATGGTGTTGAACGATATCATC RES
10 MW759888.1_MN2-2-12     GATTACGATACGAGTTCCATGTCCAAATCGAAACAAAAAGTGGACGAGTGCGGTTACAGTGATTCACAAAAGCACACGGTCGTCGACATGAGAGACGTTATGGTGTTGAACGATATCATC RES
11 MW759889.1_MN2-6-12     GATTACGATACGAGTTCCATGTCCAAATCGAAACAAAAAGTGGACGAGTGCGGTTACAGTGATTCACAAAAGCACACGGTCGTCGACATGAGAGACGTTATGGTGTTGAACGATATCATC RES
12 MW759890.1_MN2-9-12     GATTACGATACGAGTTCCATGTCCAAATCGAAACAAAAAGTGGACGAGTGCGGTTACAGTGATTCACAAAAGCACACGGTCGTCGACATGAGAGACGTTATGGTGTTGAACGATATCATC RES
13 MW759892.1_Suth5-12     GATTACGATACGAGTTCCATGTCCAAATCGAAACAAAAAGTGGACGAGTGCGGTTACAGTGATTCACAAAAGCACACGGTCGTCGACATGAGAGACGTTATGGTGTTGAACGATATCATC RES
14 MW759893.1_Suth6-12     GATTACGATACGAGTTCCATGTCCAAATCGAAACAAAAAGTGGACGAGTGCGGTTACAGTGATTCACAAAAGCACACGGTCGTCGACATGAGAGACGTTATGGTGTTGAACGATATCATC RES

                            E  Q  A  A  G  R  Q  S  R  G  S  E  K  A  V  I  T  G  G  T  L  V  G  A  W  G  T  V  S  T  V  Y  V  F  P  T  D  E  D  A  760
                       2161          .         .         .         2         .         .         .         .         :         .         .         . 2280
 1 AG6007485-RA            GAACAGGCAGCCGGACGTCAGAGTAGGGGTAGCGAAAAAGCAGTAATAACGGGGGGTACTCTCGTGGGTGCATGGGGTACAGTGTCCACGGTGTACGTGTTCCCAACAGATGAAGATGCG
 2 MT379843.1_X1           GAACAGGCAGCCGGACGTCAGAGTAGGGGTAGCGAAAAAGCAGTAATAACGGGGGGTACTCTCGTGGGTGCATGGGGTACAGTGTCCACGGTGTACGTGTTCCCAACAGATGAAGATGCG
 3 MT379843.1_X2           GAACAGGCAGCCGGACGTCAGAGTAGGGGTAGCGAAAAAGCAGTAATAACGGGGGGTACTCTCGTGGGTGCATGGGGTACAGTGTCCACGGTGTACGTGTTCCCAACAGATGAAGATGCG
 4 MT379843.1_X3           GAACAGGCAGCCGGACGTCAGAGTAGGGGTAGCGAAAAAGCAGTAATAACGGGGGGTACTCTCGTGGGTGCATGGGGTACAGTGTCCACGGTGTACGTGTTCCCAACAGATGAAGATGCG
 5 MW759883.1_B1B-12       GAACAGGCAGCCGGACGTCAGAGTAGGGGTAGCGAAAAAGC+++++++++++++++++++++++++++++++++++++++AGTGTCCACGGTGTACGTGTTCCCAACAGATGAAGATGCG SUS
 6 MW759884.1_B3A-12       GAACAGGCAGCCGGACGTCAGAGTAGGGGTAGCGAAAAAGC+++++++++++++++++++++++++++++++++++++++AGTGTCCACGGTGTACGTGTTCCCAACAGATGAAGATGCG SUS
 7 MW759885.1_MN1-4-12     GAACAGGCAGCCGGACGTCAGAGTAGGGGTAGCGAAAAAGC+++++++++++++++++++++++++++++++++++++++AGTGTCCACGGTGTACGTGTTCCCAACAGATGAAGATGCG RES
 8 MW759886.1_MN1-5-12     GAACAGGCAGCCGGACGTCAGAGTAGGGGTAGCGAAAAAGC+++++++++++++++++++++++++++++++++++++++AGTGTCCACGGTGTACGTGTTCCCAACAGATGAAGATGCG RES
 9 MW759887.1_MN1-7-12     GAACAGGCAGCCGGACGTCAGAGTAGGGGTAGCGAAAAAGC+++++++++++++++++++++++++++++++++++++++AGTGTCCACGGTGTACGTGTTCCCAACAGATGAAGATGCG RES
10 MW759888.1_MN2-2-12     GAACAGGCAGCCGGACGTCAGAGTAGGGGTAGCGAAAAAGC+++++++++++++++++++++++++++++++++++++++AGTGTCCACGGTGTACGTGTTCCCAACAGATGAAGATGCG RES
11 MW759889.1_MN2-6-12     GAACAGGCAGCCGGACGTCAGAGTAGGGGTAGCGAAAAAGC+++++++++++++++++++++++++++++++++++++++AGTGTCCACGGTGTACGTGTTCCCAACAGATGAAGATGCG RES
12 MW759890.1_MN2-9-12     GAACAGGCAGCCGGACGTCAGAGTAGGGGTAGCGAAAAAGC+++++++++++++++++++++++++++++++++++++++AGTGTCCACGGTGTACGTGTTCCCAACAGATGAAGATGCG RES
13 MW759892.1_Suth5-12     GAACAGGCAGCCGGACGTCAGAGTAGGGGTAGCGAAAAAGC+++++++++++++++++++++++++++++++++++++++AGTGTCCACGGTGTACGTGTTCCCAACAGATGAAGATGCG RES
14 MW759893.1_Suth6-12     GAACAGGCAGCCGGACGTCAGAGTAGGGGTAGCGAAAAAGC+++++++++++++++++++++++++++++++++++++++AGTGTCCACGGTGTACGTGTTCCCAACAGATGAAGATGCG RES

                            V  D  G  E  D  E  E  E  E  N  E  E  P  T  F  R  E  K  F  Q  V  W  L  L  K  F  I  D  T  F  C  V  W  D  C  G  W  P  W  L  800
                       2281          .         3         .         .         .         .         :         .         .         .         .         4 2400
 1 AG6007485-RA            GTCGACGGAGAAGACGAAGAGGAAGAAAATGAGGAACCCACTTTTCGTGAAAAGTTCCAAGTATGGTTATTGAAGTTCATCGACACGTTTTGTGTCTGGGACTGTGGATGGCCGTGGCTC
 2 MT379843.1_X1           GTCGACGGAGAAGACGAAGAGGAAGAAAATGAGGAACCCACTTTTCGTGAAAAGTTCCAAGTATGGTTATTGAAGTTCATCGACACGTTTTGTGTCTGGGACTGTGGATGGCCGTGGCTC
 3 MT379843.1_X2           GTCGACGGAGAAGACGAAGAGGAAGAAAATGAGGAACCCACTTTTCGTGAAAAGTTCCAAGTATGGTTATTGAAGTTCATCGACACGTTTTGTGTCTGGGACTGTGGATGGCCGTGGCTC
 4 MT379843.1_X3           GTCGACGGAGAAGACGAAGAGGAAGAAAATGAGGAACCCACTTTTCGTGAAAAGTTCCAAGTATGGTTATTGAAGTTCATCGACACGTTTTGTGTCTGGGACTGTGGATGGCCGTGGCTC
 5 MW759883.1_B1B-12       GTCGACGGAGAAGACGAAGAGGAAGAAAATGAGGAACCCACTTTTCGTGAAAAGTTCCAAGTATGGTTATTGAAGTTCATCGACACGTTTTGTGTCTGGGACTGTGGATGGCCGTGGCTC SUS
 6 MW759884.1_B3A-12       GTCGACGGAGAAGACGAAGAGGAAGAAAATGAGGAACCCACTTTTCGTGAAAAGTTCCAAGTATGGTTATTGAAGTTCATCGACACGTTTTGTGTCTGGGACTGTGGATGGCCGTGGCTC SUS
 7 MW759885.1_MN1-4-12     GTCGACGGAGAAGACGAAGAGGAAGAAAATGAGGAACCCACTTTTCGTGAAAAGTTCCAAGTATGGTTATTGAAGTTCATCGACACGTTTTGTGTCTGGGACTGTGGATGGCCGTGGCTC RES
 8 MW759886.1_MN1-5-12     GTCGACGGAGAAGACGAAGAGGAAGAAAATGAGGAACCCACTTTTCGTGAAAAGTTCCAAGTATGGTTATTGAAGTTCATCGACACGTTTTGTGTCTGGGACTGTGGATGGCCGTGGCTC RES
 9 MW759887.1_MN1-7-12     GTCGACGGAGAAGACGAAGAGGAAGAAAATGAGGAACCCACTTTTCGTGAAAAGTTCCAAGTATGGTTATTGAAGTTCATCGACACGTTTTGTGTCTGGGACTGTGGATGGCCGTGGCTC RES
10 MW759888.1_MN2-2-12     GTCGACGGAGAAGACGAAGAGGAAGAAAATGAGGAACCCACTTTTCGTGAAAAGTTCCAAGTATGGTTATTGAAGTTCATCGACACGTTTTGTGTCTGGGACTGTGGATGGCCGTGGCTC RES
11 MW759889.1_MN2-6-12     GTCGACGGAGAAGACGAAGAGGAAGAAAATGAGGAACCCACTTTTCGTGAAAAGTTCCAAGTATGGTTATTGAAGTTCATCGACACGTTTTGTGTCTGGGACTGTGGATGGCCGTGGCTC RES
12 MW759890.1_MN2-9-12     GTCGACGGAGAAGACGAAGAGGAAGAAAATGAGGAACCCACTTTTCGTGAAAAGTTCCAAGTATGGTTATTGAAGTTCATCGACACGTTTTGTGTCTGGGACTGTGGATGGCCGTGGCTC RES
13 MW759892.1_Suth5-12     GTCGACGGAGAAGACGAAGAGGAAGAAAATGAGGAACCCACTTTTCGTGAAAAGTTCCAAGTATGGTTATTGAAGTTCATCGACACGTTTTGTGTCTGGGACTGTGGATGGCCGTGGCTC RES
14 MW759893.1_Suth6-12     GTCGACGGAGAAGACGAAGAGGAAGAAAATGAGGAACCCACTTTTCGTGAAAAGTTCCAAGTATGGTTATTGAAGTTCATCGACACGTTTTGTGTCTGGGACTGTGGATGGCCGTGGCTC RES
```

```
                                                      .............................DII S1...............................
                                 K  F  Q  Q  G  L  A  F  I  V  F  D  P  F  V  E  L  Y  I  T  L  C  I  V  V  N  T  L  F  M  A  L  D  H  H  E  M  D  P  K  840
                           2401                   .         .         .         .         :         .         .         .         .         5         .         . 2520
 1 AG6007485-RA                  AAGTTTCAGCAAGGACTCGCATTCATAGTGTTTGATCCATTCGTCGAACTCTACATCACCCTGTGTATTGTAGTTAACACGTTATTTATGGCGCTCGATCATCATGAAATGGATCCCAAA
 2 MT379843.1_X1                 AAGTTTCAGCAAGGACTCGCATTCATAGTGTTTGATCCATTCGTCGAACTCTACATCACCCTGTGTATTGTAGTTAACACGTTATTTATGGCGCTCGATCATCATGAAATGGATCCCAAA
 3 MT379843.1_X2                 AAGTTTCAGCAAGGACTCGCATTCATAGTGTTTGATCCATTCGTCGAACTCTACATCACCCTGTGTATTGTAGTTAACACGTTATTTATGGCGCTCGATCATCATGAAATGGATCCCAAA
 4 MT379843.1_X3                 AAGTTTCAGCAAGGACTCGCATTCATAGTGTTTGATCCATTCGTCGAACTCTACATCACCCTGTGTATTGTAGTTAACACGTTATTTATGGCGCTCGATCATCATGAAATGGATCCCAAA
 5 MW759883.1_B1B-12             AAGTTTCAGCAAGGACTCGCATTCATAGTGTTTGATCCATTCGTCGAACTCTACATCACCCTGTGTATTGTAGTTAACACGTTATTTATGGCGCTCGATCATCATGAAATGGATCCCAAA SUS
 6 MW759884.1_B3A-12             AAGTTTCAGCAAGGACTCGCATTCATAGTGTTTGATCCATTCGTCGAACTCTACATCACCCTGTGTATTGTAGTTAACACGTTATTTATGGCGCTCGATCATCATGAAATGGATCCCAAA SUS
 7 MW759885.1_MN1-4-12           AAGTTTCAGCAAGGACTCGCATTCATAGTGTTTGATCCATTCGTCGAACTCTACATCACCCTGTGTATTGTAGTTAACACGTTATTTATGGCGCTCGATCATCATGAAATGGATCCCAAA RES
 8 MW759886.1_MN1-5-12           AAGTTTCAGCAAGGACTCGCATTCATAGTGTTTGATCCATTCGTCGAACTCTACATCACCCTGTGTATTGTAGTTAACACGTTATTTATGGCGCTCGATCATCATGAAATGGATCCCAAA RES
 9 MW759887.1_MN1-7-12           AAGTTTCAGCAAGGACTCGCATTCATAGTGTTTGATCCATTCGTCGAACTCTACATCACCCTGTGTATTGTAGTTAACACGTTATTTATGGCGCTCGATCATCATGAAATGGATCCCAAA RES
10 MW759888.1_MN2-2-12           AAGTTTCAGCAAGGACTCGCATTCATAGTGTTTGATCCATTCGTCGAACTCTACATCACCCTGTGTATTGTAGTTAACACGTTATTTATGGCGCTCGATCATCATGAAATGGATCCCAAA RES
11 MW759889.1_MN2-6-12           AAGTTTCAGCAAGGACTCGCATTCATAGTGTTTGATCCATTCGTCGAACTCTACATCACCCTGTGTATTGTAGTTAACACGTTATTTATGGCGCTCGATCATCATGAAATGGATCCCAAA RES
12 MW759890.1_MN2-9-12           AAGTTTCAGCAAGGACTCGCATTCATAGTGTTTGATCCATTCGTCGAACTCTACATCACCCTGTGTATTGTAGTTAACACGTTATTTATGGCGCTCGATCATCATGAAATGGATCCCAAA RES
13 MW759892.1_Suth5-12           AAGTTTCAGCAAGGACTCGCATTCATAGTGTTTGATCCATTCGTCGAACTCTACATCACCCTGTGTATTGTAGTTAACACGTTATTTATGGCGCTCGATCATCATGAAATGGATCCCAAA RES
14 MW759893.1_Suth6-12           AAGTTTCAGCAAGGACTCGCATTCATAGTGTTTGATCCATTCGTCGAACTCTACATCACCCTGTGTATTGTAGTTAACACGTTATTTATGGCGCTCGATCATCATGAAATGGATCCCAAA RES
15 Suth4-12: MW759891.1          ----------------------------------------------------------------------------------TATTTATGGCGCTCGATCATCATGAAATGGATCCCAAA RES

                                          ............................DII S2..................................          .....DII S3....
                                 L  D  F  I  L  N  K  A  N  V  F  F  S  A  T  F  G  V  E  A  A  L  K  L  M  A  M  S  P  K  Y  Y  F  Q  M  G  W  N  I  F  880
                           2521                   .         .         :         .         .         .         .         6         .         .         . 2640
 1 AG6007485-RA                  TTGGATTTCATACTCAACAAGGCTAATGTTTTTTTCAGTGCTACGTTCGGCGTTGAAGCAGCTCTGAAACTTATGGCTATGAGTCCTAAGTATTACTTCCAAATGGGCTGGAACATCTTT
 2 MT379843.1_X1                 TTGGATTTCATACTCAACAAGGCTAATGTTTTTTTCAGTGCTACGTTCGGCGTTGAAGCAGCTCTGAAACTTATGGCTATGAGTCCTAAGTATTACTTCCAAATGGGCTGGAACATCTTT
 3 MT379843.1_X2                 TTGGATTTCATACTCAACAAGGCTAATGTTTTTTTCAGTGCTACGTTCGGCGTTGAAGCAGCTCTGAAACTTATGGCTATGAGTCCTAAGTATTACTTCCAAATGGGCTGGAACATCTTT
 4 MT379843.1_X3                 TTGGATTTCATACTCAACAAGGCTAATGTTTTTTTCAGTGCTACGTTCGGCGTTGAAGCAGCTCTGAAACTTATGGCTATGAGTCCTAAGTATTACTTCCAAATGGGCTGGAACATCTTT
 5 MW759883.1_B1B-12             TTGGATTTCATACTCAACAAGGCTAATGTTTTTTTCAGTGCTACGTTCGGCGTTGAAGCAGCTCTGAAACTTATGGCTATGAGTCCTAAGTATTACTTCCAAATGGGCTGGAACATCTTT SUS
 6 MW759884.1_B3A-12             TTGGATTTCATACTCAACAAGGCTAATGTTTTTTTCAGTGCTACGTTCGGCGTTGAAGCAGCTCTGAAACTTATGGCTATGAGTCCTAAGTATTACTTCCAAATGGGCTGGAACATCTTT SUS
 7 MW759885.1_MN1-4-12           TTGGATTTCATACTCAACAAGGCTAATGTTTTTTTCAGTGCTACGTTCGGCGTTGAAGCAGCTCTGAAACTTATGGCTATGAGTCCTAAGTATTACTTCCAAATGGGCTGGAACATCTTT RES
 8 MW759886.1_MN1-5-12           TTGGATTTCATACTCAACAAGGCTAATGTTTTTTTCAGTGCTACGTTCGGCGTTGAAGCAGCTCTGAAACTTATGGCTATGAGTCCTAAGTATTACTTCCAAATGGGCTGGAACATCTTT RES
 9 MW759887.1_MN1-7-12           TTGGATTTCATACTCAACAAGGCTAATGTTTTTTTCAGTGCTACGTTCGGCGTTGAAGCAGCTCTGAAACTTATGGCTATGAGTCCTAAGTATTACTTCCAAATGGGCTGGAACATCTTT RES
10 MW759888.1_MN2-2-12           TTGGATTTCATACTCAACAAGGCTAATGTTTTTTTCAGTGCTACGTTCGGCGTTGAAGCAGCTCTGAAACTTATGGCTATGAGTCCTAAGTATTACTTCCAAATGGGCTGGAACATCTTT RES
11 MW759889.1_MN2-6-12           TTGGATTTCATACTCAACAAGGCTAATGTTTTTTTCAGTGCTACGTTCGGCGTTGAAGCAGCTCTGAAACTTATGGCTATGAGTCCTAAGTATTACTTCCAAATGGGCTGGAACATCTTT RES
12 MW759890.1_MN2-9-12           TTGGATTTCATACTCAACAAGGCTAATGTTTTTTTCAGTGCTACGTTCGGCGTTGAAGCAGCTCTGAAACTTATGGCTATGAGTCCTAAGTATTACTTCCAAATGGGCTGGAACATCTTT RES
13 MW759892.1_Suth5-12           TTGGATTTCATACTCAACAAGGCTAATGTTTTTTTCAGTGCTACGTTCGGCGTTGAAGCAGCTCTGAAACTTATGGCTATGAGTCCTAAGTATTACTTCCAAATGGGCTGGAACATCTTT RES
14 MW759893.1_Suth6-12           TTGGATTTCATACTCAACAAGGCTAATGTTTTTTTCAGTGCTACGTTCGGCGTTGAAGCAGCTCTGAAACTTATGGCTATGAGTCCTAAGTATTACTTCCAAATGGGCTGGAACATCTTT RES
15 Suth4-12: MW759891.1          TTGGATTTCATACTCAACAAGGCTAATGTTTTTTTCAGTGCTACGTTCGGCGTTGAAGCAGCTCTGAAACTTATGGCTATGAGTCCTAAGTATTACTTCCAAATGGGCTGGAACATCTTT RES

                                 .............DII S3...................              ...........................DII S4.......................
                                 D  F  I  I  V  I  L  S  V  V  E  L  L  S  A  G  Y  Q  G  L  S  V  L  R  S  F  R  L  L  R  V  F  K  L  A  K  S  W  P  T  920
                           2641                   :         .         .         .         .         7         .         .         .         .         :         . 2760
 1 AG6007485-RA                  GACTTCATCATCGTAATTCTTTCCGTAGTAGAATTGCTCTCCGCGGGTTACCAAGGACTTTCCGTATTGCGTTCATTTCGTTTGCTTCGAGTATTTAAGTTGGCAAAATCTTGGCCCACA
 2 MT379843.1_X1                 GACTTCATCATCGTAATTCTTTCCGTAGTAGAATTGCTCTCCGCGGGTTACCAAGGACTTTCCGTATTGCGTTCATTTCGTTTGCTTCGAGTATTTAAGTTGGCAAAATCTTGGCCCACA
 3 MT379843.1_X2                 GACTTCATCATCGTAATTCTTTCCGTAGTAGAATTGCTCTCCGCGGGTTACCAAGGACTTTCCGTATTGCGTTCATTTCGTTTGCTTCGAGTATTTAAGTTGGCAAAATCTTGGCCCACA
 4 MT379843.1_X3                 GACTTCATCATCGTAATTCTTTCCGTAGTAGAATTGCTCTCCGCGGGTTACCAAGGACTTTCCGTATTGCGTTCATTTCGTTTGCTTCGAGTATTTAAGTTGGCAAAATCTTGGCCCACA
 5 MW759883.1_B1B-12             GACTTCATCATCGTAATTCTTTCCGTAGTAGAATTGCTCTCCGCGGGTTACCAAGGACTTTCCGTATTGCGTTCATTTCGTTTGCTTCGAGTATTTAAGTTGGCAAAATCTTGGCCCACA SUS
 6 MW759884.1_B3A-12             GACTTCATCATCGTAATTCTTTCCGTAGTAGAATTGCTCTCCGCGGGTTACCAAGGACTTTCCGTATTGCGTTCATTTCGTTTGCTTCGAGTATTTAAGTTGGCAAAATCTTGGCCCACA SUS
 7 MW759885.1_MN1-4-12           GACTTCATCATCGTAATTCTTTCCGTAGTAGAATTGCTCTCCGCGGGTTACCAAGGACTTTCCGTATTGCGTTCATTTCGTTTGCTTCGAGTATTTAAGTTGGCAAAATCTTGGCCCACA RES
 8 MW759886.1_MN1-5-12           GACTTCATCATCGTAATTCTTTCCGTAGTAGAATTGCTCTCCGCGGGTTACCAAGGACTTTCCGTATTGCGTTCATTTCGTTTGCTTCGAGTATTTAAGTTGGCAAAATCTTGGCCCACA RES
 9 MW759887.1_MN1-7-12           GACTTCATCATCGTAATTCTTTCCGTAGTAGAATTGCTCTCCGCGGGTTACCAAGGACTTTCCGTATTGCGTTCATTTCGTTTGCTTCGAGTATTTAAGTTGGCAAAATCTTGGCCCACA RES
10 MW759888.1_MN2-2-12           GACTTCATCATCGTAATTCTTTCCGTAGTAGAATTGCTCTCCGCGGGTTACCAAGGACTTTCCGTATTGCGTTCATTTCGTTTGCTTCGAGTATTTAAGTTGGCAAAATCTTGGCCCACA RES
11 MW759889.1_MN2-6-12           GACTTCATCATCGTAATTCTTTCCGTAGTAGAATTGCTCTCCGCGGGTTACCAAGGACTTTCCGTATTGCGTTCATTTCGTTTGCTTCGAGTATTTAAGTTGGCAAAATCTTGGCCCACA RES
12 MW759890.1_MN2-9-12           GACTTCATCATCGTAATTCTTTCCGTAGTAGAATTGCTCTCCGCGGGTTACCAAGGACTTTCCGTATTGCGTTCATTTCGTTTGCTTCGAGTATTTAAGTTGGCAAAATCTTGGCCCACA RES
13 MW759892.1_Suth5-12           GACTTCATCATCGTAATTCTTTCCGTAGTAGAATTGCTCTCCGCGGGTTACCAAGGACTTTCCGTATTGCGTTCATTTCGTTTGCTTCGAGTATTTAAGTTGGCAAAATCTTGGCCCACA RES
14 MW759893.1_Suth6-12           GACTTCATCATCGTAATTCTTTCCGTAGTAGAATTGCTCTCCGCGGGTTACCAAGGACTTTCCGTATTGCGTTCATTTCGTTTGCTTCGAGTATTTAAGTTGGCAAAATCTTGGCCCACA RES
```

```
15 Suth4-12: MW759891.1      GACTTCATCATCGTAATTCTTTCCGTAGTAGAATTGCTCTCCGCGGGTTACCAAGGACTTTCCGTATTGCGTTCATTTCGTTTGCTTCGAGTATTTAAGTTGGCAAAATCTTGGCCCACA RES

                             ...                                         ..........................DII S5...........................
                              L  N  L  L  I  S  I  MI G  R  T  I  G  A  L  G  N  L  T  F  V  L  C  I  I  I  F  I  F  A  V  M  G  M  Q  L  F  G  K  N   960
                       2761           .         .M918I    .         8         .         .         .         .         :         .         .         . 2880
 1 AG6007485-RA                CTTAATCTTTTAATATCAATAATGGGTCGAACCATTGGTGCTTTGGGTAACCTAACGTTTGTGTTGTGCATAATCATATTTATATTCGCCGTTATGGGTATGCAGTTATTTGGAAAAAAC
 2 MT379843.1_X1              CTTAATCTTTTAATATCAATAATGGGTCGAACCATTGGTGCTTTGGGTAACCTAACGTTTGTGTTGTGCATAATCATATTTATATTCGCCGTTATGGGTATGCAGTTATTTGGAAAAAAC
 3 MT379843.1_X2              CTTAATCTTTTAATATCAATAATGGGTCGAACCATTGGTGCTTTGGGTAACCTAACGTTTGTGTTGTGCATAATCATATTTATATTCGCCGTTATGGGTATGCAGTTATTTGGAAAAAAC
 4 MT379843.1_X3              CTTAATCTTTTAATATCAATAATGGGTCGAACCATTGGTGCTTTGGGTAACCTAACGTTTGTGTTGTGCATAATCATATTTATATTCGCCGTTATGGGTATGCAGTTATTTGGAAAAAAC
 5 MW759883.1_B1B-12          CTTAATCTTTTAATATCAATAATGGGTCGAACCATTGGTGCTTTGGGTAACCTAACGTTTGTGTTGTGCATAATCATATTTATATTCGCCGTTATGGGTATGCAGTTATTTGGAAAAAAC SUS
 6 MW759884.1_B3A-12          CTTAATCTTTTAATATCAATAATGGGTCGAACCATTGGTGCTTTGGGTAACCTAACGTTTGTGTTGTGCATAATCATATTTATATTCGCCGTTATGGGTATGCAGTTATTTGGAAAAAAC SUS
 7 MW759885.1_MN1-4-12        CTTAATCTTTTAATATCAATAATRGGTCGAACCATTGGTGCTTTGGGTAACCTAACGTTTGTGTTGTGCATAATCATATTTATATTCGCCGTTATGGGTATGCAGTTATTTGGAAAAAAC RES
 8 MW759886.1_MN1-5-12        CTTAATCTTTTAATATCAATAATRGGTCGAACCATTGGTGCTTTGGGTAACCTAACGTTTGTGTTGTGCATAATCATATTTATATTCGCCGTTATGGGTATGCAGTTATTTGGAAAAAAC RES
 9 MW759887.1_MN1-7-12        CTTAATCTTTTAATATCAATAATRGGTCGAACCATTGGTGCTTTGGGTAACCTAACGTTTGTGTTGTGCATAATCATATTTATATTCGCCGTTATGGGTATGCAGTTATTTGGAAAAAAC RES
10 MW759888.1_MN2-2-12        CTTAATCTTTTAATATCAATAATAGGTCGAACCATTGGTGCTTTGGGTAACCTAACGTTTGTGTTGTGCATAATCATATTTATATTCGCCGTTATAGGTATGCAGTTATTTGGAAAAAAC RES
11 MW759889.1_MN2-6-12        CTTAATCTTTTAATATCAATAATGGGTCGAACCATTGGTGCTTTGGGTAACCTAACGTTTGTGTTGTGCATAATCATATTTATATTCGCCGTTATGGGTATGCAGTTATTTGGAAAAAAC RES
12 MW759890.1_MN2-9-12        CTTAATCTTTTAATATCAATAATRGGTCGAACCATTGGTGCTTTGGGTAACCTAACGTTTGTGTTGTGCATAATCATATTTATATTCGCCGTTATGGGTATGCAGTTATTTGGAAAAAAC RES
13 MW759892.1_Suth5-12        CTTAATCTTTTAATATCAATAATRGGTCGAACCATTGGTGCTTTGGGTAACCTAACGTTTGTGTTGTGCATAATCATATTTATATTCGCCGTTATGGGTATGCAGTTATTTGGAAAAAAC RES
14 MW759893.1_Suth6-12        CTTAATCTTTTAATATCAATAATRGGTCGAACCATTGGTGCTTTGGGTAACCTAACGTTTGTGTTGTGCATAATCATATTTATATTCGCCGTTATGGGTATGCAGTTATTTGGAAAAAAC RES
15 Suth4-12: MW759891.1      CTTAATCTTTTAATATCAATAATGGGTCGAACCATTGGTGCTTTGGGTAACCTAACGTTTGTGTTGTGCATAATCATATTTATATTCGCCGTTATGGGTATGCAGTTATTTGGAAAAAAC RES

                              Y  T  E  K  M  Y  L  F  K  D  H  E  L  P  R  W  N  F  T  D  F  L  H  S  F  M  I  V  F  R  V  L  C  G  E  W  I  E  S  M  1000
                       2881           .         9         .         .         .         .         :         .         .         .         .         0 3000
 1 AG6007485-RA                TACACAGAAAAAAATGTACTTATTCAAAGACCACGAGCTTCCCCGGTGGAACTTCACCGATTTTTTGCACTCGTTTATGATAGTATTTCGAGTATTATGTGGTGAATGGATTGAATCAATG
 2 MT379843.1_X1              TACACAGAAAAAAATGTACTTATTCAAAGACCACGAGCTTCCCCGGTGGAACTTCACCGATTTTTTGCACTCGTTTATGATAGTATTTCGAGTATTATGTGGTGAATGGATTGAATCAATG
 3 MT379843.1_X2              TACACAGAAAAAAATGTACTTATTCAAAGACCACGAGCTTCCCCGGTGGAACTTCACCGATTTTTTGCACTCGTTTATGATAGTATTTCGAGTATTATGTGGTGAATGGATTGAATCAATG
 4 MT379843.1_X3              TACACAGAAAAAAATGTACTTATTCAAAGACCACGAGCTTCCCCGGTGGAACTTCACCGATTTTTTGCACTCGTTTATGATAGTATTTCGAGTATTATGTGGTGAATGGATTGAATCAATG
 5 MW759883.1_B1B-12          TACACAGAAAAAAATGTACTTATTCAAAGACCACGAGCTTCCCCGGTGGAACTTCACCGATTTTTTGCACTCGTTTATGATAGTATTTCGAGTATTATGTGGTGAATGGATTGAATCAATG SUS
 6 MW759884.1_B3A-12          TACACAGAAAAAAATGTACTTATTCAAAGACCACGAGCTTCCCCGGTGGAACTTCACCGATTTTTTGCACTCGTTTATGATAGTATTTCGAGTATTATGTGGTGAATGGATTGAATCAATG SUS
 7 MW759885.1_MN1-4-12        TACACAGAAAAAAATGTACTTATTCAAAGACCACGAGCTTCCCCGGTGGAACTTCACCGATTTTTTGCACTCGTTTATGATAGTATTTCGAGTATTATGTGGTGAATGGATTGAATCAATG RES
 8 MW759886.1_MN1-5-12        TACACAGAAAAAAATGTACTTATTCAAAGACCACGAGCTTCCCCGGTGGAACTTCACCGATTTTTTGCACTCGTTTATGATAGTATTTCGAGTATTATGTGGTGAATGGATTGAATCAATG RES
 9 MW759887.1_MN1-7-12        TACACAGAAAAAAATGTACTTATTCAAAGACCACGAGCTTCCCCGGTGGAACTTCACCGATTTTTTGCACTCGTTTATGATAGTATTTCGAGTATTATGTGGTGAATGGATTGAATCAATG RES
10 MW759888.1_MN2-2-12        TACACAGAAAAAAATGTACTTATTCAAAGACCACGAGCTTCCCCGGTGGAACTTCACCGATTTTTTGCACTCGTTTATGATAGTATTTCGAGTATTATGTGGTGAATGGATTGAATCAATG RES
11 MW759889.1_MN2-6-12        TACACAGAAAAAAATGTACTTATTCAAAGACCACGAGCTTCCCCGGTGGAACTTCACCGATTTTTTGCACTCGTTTATGATAGTATTTCGAGTATTATGTGGTGAATGGATTGAATCAATG RES
12 MW759890.1_MN2-9-12        TACACAGAAAAAAATGTACTTATTCAAAGACCACGAGCTTCCCCGGTGGAACTTCACCGATTTTTTGCACTCGTTTATGATAGTATTTCGAGTATTATGTGGTGAATGGATTGAATCAATG RES
13 MW759892.1_Suth5-12        TACACAGAAAAAAATGTACTTATTCAAAGACCACGAGCTTCCCCGGTGGAACTTCACCGATTTTTTGCACTCGTTTATGATAGTATTTCGAGTATTATGTGGTGAATGGATTGAATCAATG RES
14 MW759893.1_Suth6-12        TACACAGAAAAAAATGTACTTATTCAAAGACCACGAGCTTCCCCGGTGGAACTTCACCGATTTTTTGCACTCGTTTATGATAGTATTTCGAGTATTATGTGGTGAATGGATTGAATCAATG RES
15 Suth4-12: MW759891.1      TACACAGAAAAAAATGTACTTATTCAAAGACCACGAGCTTCCCCGGTGGAACTTCACCGATTTTTTGCACTCGTTTATGATAGTATTTCGAGTATTATGTGGTGAATGGATTGAATCAATG RES

                                                        ................................DII S6.................................
                              W  D  C  L  H  V  G  E  P  T  C  I  P  F  F  L  A  T  V  V  I  G  N  LF V  V  V  L  N  L  F  L  A  L  L  L  S  N  F  G  1040
                       3001           .         .         .         .         :         .         .L1014F    .         .         1         .         . 3120
 1 AG6007485-RA                TGGGACTGCTTACACGTTGGAGAACCAACGTGTATACCATTCTTCTTGGCTACTGTTGTCATCGGTAACCTTGTGGTA+++CTTAATCTTTTCTTGGCGTTGTTGCTGAGTAATTTTGGC
 2 MT379843.1_X1              TGGGACTGCTTACACGTTGGAGAACCAACGTGTATACCATTCTTCTTGGCTACTGTTGTCATCGGTAACCTTGTGGTA+++CTTAATCTTTTCTTGGCGTTGTTGCTGAGTAATTTTGGC
 3 MT379843.1_X2              TGGGACTGCTTACACGTTGGAGAACCAACGTGTATACCATTCTTCTTGGCTACTGTTGTCATCGGTAACCTTGTGGTAGTACTTAATCTTTTCTTGGCGTTGTTGCTGAGTAATTTTGGC
 4 MT379843.1_X3              TGGGACTGCTTACACGTTGGAGAACCAACGTGTATACCATTCTTCTTGGCTACTGTTGTCATCGGTAACCTTGTGGTA+++CTTAATCTTTTCTTGGCGTTGTTGCTGAGTAATTTTGGC
 5 MW759883.1_B1B-12          TGGGACTGCTTACACGTTGGAGAACCAACGTGTATACCATTCTTCTTGGCTACTGTTGTCATCGGTAACCTTGTGGTA+++CTTAATCTTTTCTTGGCGTTGTTGCTGAGTAATTTTGGC SUS
 6 MW759884.1_B3A-12          TGGGACTGCTTACACGTTGGAGAACCAACGTGTATACCATTCTTCTTGGCTACTGTTGTCATCGGTAACCTTGTGGTA+++CTTAATCTTTTCTTGGCGTTGTTGCTGAGTAATTTTGGC SUS
 7 MW759885.1_MN1-4-12        TGGGACTGCTTACACGTTGGAGAACCAACGTGTATACCATTCTTCTTGGCTACTGTTGTCATCGGTAACYTTGTGGTA+++CTTAATCTTTTCTTGGCGTTGTTGCTGAGTAATTTTGGC RES
 8 MW759886.1_MN1-5-12        TGGGACTGCTTACACGTTGGAGAACCAACGTGTATACCATTCTTCTTGGCTACTGTTGTCATCGGTAACYTTGTGGTA+++CTTAATCTTTTCTTGGCGTTGTTGCTGAGTAATTTTGGC RES
 9 MW759887.1_MN1-7-12        TGGGACTGCTTACACGTTGGAGAACCAACGTGTATACCATTCTTCTTGGCTACTGTTGTCATCGGTAACYTTGTGGTA+++CTTAATCTTTTCTTGGCGTTGTTGCTGAGTAATTTTGGC RES
10 MW759888.1_MN2-2-12        TGGGACTGCTTACACGTTGGAGAACCAACGTGTATACCATTCTTCTTGGCTACTGTTGTCATCGGTAACYTTGTGGTA+++CTTAATCTTTTCTTGGCGTTGTTGCTGAGTAATTTTGGC RES
11 MW759889.1_MN2-6-12        TGGGACTGCTTACACGTTGGAGAACCAACGTGTATACCATTCTTCTTGGCTACTGTTGTCATCGGTAACTTTGTGGTA+++CTTAATCTTTTCTTGGCGTTGTTGCTGAGTAATTTTGGC RES
12 MW759890.1_MN2-9-12        TGGGACTGCTTACACGTTGGAGAACCAACGTGTATACCATTCTTCTTGGCTACTGTTGTCATCGGTAACYTTGTGGTA+++CTTAATCTTTTCTTGGCGTTGTTGCTGAGTAATTTTGGC RES
13 MW759892.1_Suth5-12        TGGGACTGCTTACACGTTGGAGAACCAACGTGTATACCATTCTTCTTGGCTACTGTTGTCATCGGTAACYTTGTGGTA+++CTTAATCTTTTCTTGGCGTTGTTGCTGAGTAATTTTGGC RES
14 MW759893.1_Suth6-12        TGGGACTGCTTACACGTTGGAGAACCAACGTGTATACCATTCTTCTTGGCTACTGTTGTCATCGGTAACYTTGTGGTA+++CTTAATCTTTTCTTGGCGTTGTTGCTGAGTAATTTTGGC RES
15 Suth4-12: MW759891.1      TGGGACTGCTTACACGTTGGAGAACCAACGTGTATACCATTCTTCTTGGCTACTGTTGTCATCGGTAACTTTGTGGTA+++CTTAATCTTTTCTTGGCGTTGTTGCTGAGTAATTTTGGC RES
```

```
                                  S  S  N  L  S  V  P  T  A  D  S  D  T  N  K  I  T  E  A  F  E  R  I  G  R  F  N  K  W  V  K  T  H  I  L  N  F  L  K  V  1080
                         3121     .         .         :         .         .         .         .         2         .         .         .         .   3240
 1 AG6007485-RA                   TCGTCTAATTTATCGGTGCCTACGGCTGATAGCGACACAAACAAGATCACAGAAGCATTTGAACGTATTGGCCGATTCAATAAATGGGTGAAAACGCATATTCTGAACTTCCTCAAAGTA
 2 MT379843.1_X1                  TCGTCTAATTTATCGGTGCCTACGGCTGATAGCGACACAAACAAGATCACAGAAGCATTTGAACGTATTGGCCGATTCAATAAATGGGTGAAAACGCATATTCTGAACTTCCTCAAAGTA
 3 MT379843.1_X2                  TCGTCTAATTTATCGGTGCCTACGGCTGATAGCGACACAAACAAGATCACAGAAGCATTTGAACGTATTGGCCGATTCAATAAATGGGTGAAAACGCATATTCTGAACTTCCTCAAAGTA
 4 MT379843.1_X3                  TCGTCTAATTTATCGGTGCCTACGGCTGATAGCGACACAAACAAGATCACAGAAGCATTTGAACGTATTGGCCGATTCAATAAATGGGTGAAAACGCATATTCTGAACTTCCTCAAAGTA
 5 MW759883.1_B1B-12              TCGTCTAATTTATCGGTGCCTACGGCTGATAGCGACACAAACAAGATCACAGAAGCATTTGAACGTATTGGCCGATTCAATAAATGGGTGAAAACGCATATTCTGAACTTCCTCAAAGTA SUS
 6 MW759884.1_B3A-12              TCGTCTAATTTATCGGTGCCTACGGCTGATAGCGACACAAACAAGATCACAGAAGCATTTGAACGTATTGGCCGATTCAATAAATGGGTGAAAACGCATATTCTGAACTTCCTCAAAGTA SUS
 7 MW759885.1_MN1-4-12            TCGTCTAATTTATCGGTGCCTACGGCTGATAGCGACACAAACAAGATCACAGAAGCATTTGAACGTATTGGCCGATTCAATAAATGGGTGAAAACGCATATTCTGAACTTCCTCAAAGTA RES
 8 MW759886.1_MN1-5-12            TCGTCTAATTTATCGGTGCCTACGGCTGATAGCGACACAAACAAGATCACAGAAGCATTTGAACGTATTGGCCGATTCAATAAATGGGTGAAAACGCATATTCTGAACTTCCTCAAAGTA RES
 9 MW759887.1_MN1-7-12            TCGTCTAATTTATCGGTGCCTACGGCTGATAGCGACACAAACAAGATCACAGAAGCATTTGAACGTATTGGCCGATTCAATAAATGGGTGAAAACGCATATTCTGAACTTCCTCAAAGTA RES
10 MW759888.1_MN2-2-12            TCGTCTAATTTATCGGTGCCTACGGCTGATAGCGACACAAACAAGATCACAGAAGCATTTGAACGTATTGGCCGATTCAATAAATGGGTGAAAACGCATATTCTGAACTTCCTCAAAGTA RES
11 MW759889.1_MN2-6-12            TCGTCTAATTTATCGGTGCCTACGGCTGATAGCGACACAAACAAGATCACAGAAGCATTTGAACGTATTGGCCGATTCAATAAATGGGTGAAAACGCATATTCTGAACTTCCTCAAAGTA RES
12 MW759890.1_MN2-9-12            TCGTCTAATTTATCGGTGCCTACGGCTGATAGCGACACAAACAAGATCACAGAAGCATTTGAACGTATTGGCCGATTCAATAAATGGGTGAAAACGCATATTCTGAACTTCCTCAAAGTA RES
13 MW759892.1_Suth5-12            TCGTCTAATTTATCGGTGCCTACGGCTGATAGCGACACAAACAAGATCACAGAAGCATTTGAACGTATTGGCCGATTCAATAAATGGGTGAAAACGCATATTCTGAACTTCCTCAAAGTA RES
14 MW759893.1_Suth6-12            TCGTCTAATTTATCGGTGCCTACGGCTGATAGCGACACAAACAAGATCACAGAAGCATTTGAACGTATTGGCCGATTCAATAAATGGGTGAAAACGCATATTCTGAACTTCCTCAAAGTA RES
15 Suth4-12: MW759891.1           TCGTCTAATTTATCGGTGCCTACGGCTGATAGCGACACAAACAAGATCACAGAAGCATTTGAACGTATTGGCCGATTCAATAAATGGGTGAAAACGCATATTCTGAACTTCCTCAAAGTA RES

                                  L  R  L  K  I  T  N  Q  I  S  V  Q  V  S  G  R  D  R  D  I  D  L  P  V  D  E  T  I  V  D  V  I  A  P  F  K  D  T  K  E  1120
                         3241               :         .         .         .         .         3         .         .         .         .         :   .   3360
 1 AG6007485-RA                   CTCCGACTGAAAATCACCAATCAAATATCGGTTCAAGTGTCAGGTCGTGACAGAGATATTGACTTGCCTGTTGACGAAACAATTGTAGATGTTATTGCGCCATTTAAAGACACTAAGGAA
 2 MT379843.1_X1                  CTCCGACTGAAAATCACCAATCAAATATCGGTTCAAGTGTCAGGTCGTGACAGAGATATTGACTTGCCTGTTGACGAAACAATTGTAGATGTTATTGCGCCATTTAAAGACACTAAGGAA
 3 MT379843.1_X2                  CTCCGACTGAAAATCACCAATCAAATATCGGTTCAAGTGTCAGGTCGTGACAGAGATATTGACTTGCCTGTTGACGAAACAATTGTAGATGTTATTGCGCCATTTAAAGACACTAAGGAA
 4 MT379843.1_X3                  CTCCGACTGAAAATCACCAATCAAATATCGGTTCAAGTGTCAGGTCGTGACAGAGATATTGACTTGCCTGTTGACGAAACAATTGTAGATGTTATTGCGCCATTTAAAGACACTAAGGAA
 5 MW759883.1_B1B-12              CTCCGACTGAAAATCACCAATCAAATATCGGTTCAAGTGTCAGGTCGTGACAGAGATATTGACTTGCCTGTTGACGAAACAATTGTAGATGTTATTGCGCCATTTAAAGACACTAAGGAA SUS
 6 MW759884.1_B3A-12              CTCCGACTGAAAATCACCAATCAAATATCGGTTCAAGTGTCAGGTCGTGACAGAGATATTGACTTGCCTGTTGACGAAACAATTGTAGATGTTATTGCGCCATTTAAAGACACTAAGGAA SUS
 7 MW759885.1_MN1-4-12            CTCCGACTGAAAATCACCAATCAAATATCGGTTCAAGTGTCAGGTCGTGACAGAGATATTGACTTGCCTGTTGACGAAACAATTGTAGATGTTATTGCGCCATTTAAAGACACTAAGGAA RES
 8 MW759886.1_MN1-5-12            CTCCGACTGAAAATCACCAATCAAATATCGGTTCAAGTGTCAGGTCGTGACAGAGATATTGACTTGCCTGTTGACGAAACAATTGTAGATGTTATTGCGCCATTTAAAGACACTAAGGAA RES
 9 MW759887.1_MN1-7-12            CTCCGACTGAAAATCACCAATCAAATATCGGTTCAAGTGTCAGGTCGTGACAGAGATATTGACTTGCCTGTTGACGAAACAATTGTAGATGTTATTGCGCCATTTAAAGACACTAAGGAA RES
10 MW759888.1_MN2-2-12            CTCCGACTGAAAATCACCAATCAAATATCGGTTCAAGTGTCAGGTCGTGACAGAGATATTGACTTGCCTGTTGACGAAACAATTGTAGATGTTATTGCGCCATTTCAAGACACTAAGGAA RES
11 MW759889.1_MN2-6-12            CTCCGACTGAAAATCACCAATCAAATATCGGTTCAAGTGTCAGGTCGTGACAGAGATATTGACTTGCCTGTTGACGAAACAATTGTAGATGTTATTGCGCCATTTAAAGACACTAAGGAA RES
12 MW759890.1_MN2-9-12            CTCCGACTGAAAATCACCAATCAAATATCGGTTCAAGTGTCAGGTCGTGACAGAGATATTGACTTGCCTGTTGACGAAACAATTGTAGATGTTATTGCGCCATTTAAAGACACTAAGGAA RES
13 MW759892.1_Suth5-12            CTCCGACTGAAAATCACCAATCAAATATCGGTTCAAGTGTCAGGTCGTGACAGAGATATTGACTTGCCTGTTGACGAAACAATTGTAGATGTTATTGCGCCATTTAAAGACACTAAGGAA RES
14 MW759893.1_Suth6-12            CTCCGACTGAAAATCACCAATCAAATATCGGTTCAAGTGTCAGGTCGTGACAGAGATATTGACTTGCCTGTTGACGAAACAATTGTAGATGTTATTGCGCCATTTAAAGACACTAAGGAA RES
15 Suth4-12: MW759891.1           CTCCGACTGAAAATCACCAATCAAATATCGGTTCAAGTGTCAGGTCGTGACAGAGATATTGACTTGCCTGTTGACGAAACAATTGTAGATGTTATTGCGCCATTTAAAGACACTAAGGAA RES

                                  P  V  E  M  T  I  G  D  G  M  E  F  T  I  P  G  D  V  K  Q  K  I  K  K  N  Q  V  G  N  S  I  G  N  H  Q  G  N  K  V  G  1160
                         3361               .         .         .         4         .         .         .         .         :         .         .   .   3480
 1 AG6007485-RA                   CCAGTAGAGATGACAATTGGTGACGGGATGGAATTCACGATTCCGGGTGATGTAAAACAAAAAATAAAAAAGAACCAAGTTGGGAATTCAATTGGAAATCATCAAGGGAACAAAGTTGGA
 2 MT379843.1_X1                  CCAGTAGAGATGACAATTGGTGACGGGATGGAATTCACGATTCCGGGTGATGTAAAACAAAAAATAAAAAAGAACCAAGTTGGGAATTCAATTGGAAATCATCAAGGGAACAAAGTTGGA
 3 MT379843.1_X2                  CCAGTAGAGATGACAATTGGTGACGGGATGGAATTCACGATTCCGGGTGATGTAAAACAAAAAATAAAAAAGAACCAAGTTGGGAATTCAATTGGAAATCATCAAGGGAACAAAGTTGGA
 4 MT379843.1_X3                  CCAGTAGAGATGACAATTGGTGACGGGATGGAATTCACGATTCCGGGTGATGTAAAACAAAAAATAAAAAAGAACCAAGTTGGGAATTCAATTGGAAATCATCAAGGGAACAAAGTTGGA
 5 MW759883.1_B1B-12              CCAGTAGAGATGACAATTGGTGACGGGATGGAATTCACGATTCCGGGTGATGTAAAACAAAAAATAAAAAAAAACCAAGTTGGGAATTCAATTGGAAATCATCAAGGGAACAAAATTGGA SUS
 6 MW759884.1_B3A-12              CCAGTAGAGATGACAATTGGTGACGGGATGGAATTCACGATTCCGGGTGATGTAAAACAAAAAATAAAAAAGAACCAAGTTGGGAATTCAATTGGAAATCATCAAGGGAACAAAGTTGGA SUS
 7 MW759885.1_MN1-4-12            CCAGTAGAGATGACAATTGGTGACGGGATGGAATTCACGATTCCGGGTGATGTAAAACAAAAAATAAAAAAGAACCAAGTTGGGAATTCAATTGGAAATCATCAAGGGAACAAAGTTGGA RES
 8 MW759886.1_MN1-5-12            CCAGTAGAGATGACAATTGGTGACGGGATGGAATTCACGATTCCGGGTGATGTAAAACAAAAAATAAAAAAGAACCAAGTTGGGAATTCAATTGGAAATC-------------------- RES
 9 MW759887.1_MN1-7-12            CCAGTAGAGATGACAATTGGTGACGGGATGGAATTCACGATTCCGGGTGATGTAAAACAAAAAATAAAAAAGAACCAAGTTGGGAATTCAATTGGAAATCATCAAGGGAACAAAGTTGGA RES
10 MW759888.1_MN2-2-12            CCAGTAGAGATGACAATTGGTGACGGGATGGAATTCACGATTCCGGGTGATGTAAAACAAAAAATAAAAAAGAACCAAGTTGGGAATTCAATTGGAAATC-------------------- RES
11 MW759889.1_MN2-6-12            CCAGTAGAGATGACAATTGGTGACGGGATGGAATTCACGATTCCGGGTGATGTAAAACAAAAAATAAAAAAGAACCAAGTTGGGAATTCAATTGGAAATC-------------------- RES
12 MW759890.1_MN2-9-12            CCAGTAGAGATGACAATTGGTGACGGGATGGAATTCACGATTCCGGGTGATGTAAAACAAAAAATAAAAAAGAACCAAGTT--------------------------------------- RES
13 MW759892.1_Suth5-12            CCAGTAGAGATGACAATTGGTGACGGGATGGAATTCACGATTCCGGGTGATGTAAAACAAAAAATAAAAAAGAACCAAGTTGGGAATTCAATTGGAAATC-------------------- RES
14 MW759893.1_Suth6-12            CCAGTAGAGATGACAATTGGTGACGGGATGGAATTCACGATTCCGGGTGATGTAAAACAAAAAATAAAAAAGAACCAAGTTGGGAATTCAATTGGAAATCATCAAGGGAACAAAGTTGGA RES
15 Suth4-12: MW759891.1           CCAGTAGAGATGACAATTGGTGACGGGATGGAATTCACGATTCCGGGTGATGTAAAACAAAAAATAAAAAAGAACCAAGTTGGGAATTCAATT--------------------------- RES
```

```
                                  N  D  Y  K  K  E  S  F  D  L  D  S  L  K  CT K  E  Q  K  K  T  L  D  S  I  T  K  K  R  T  K  F  C  N  D  A  F  L  L  E  1200
                             3481          .         5         .         .                                                                                  3600
 1 AG6007485-RA                   AACGATTACAAAAAGGAAAGTTTCGACCTGGATAGTTTAAAATGCTAG------------------------------------------------------------------------
 2 MT379843.1_X1                  AACGATTACAAAAAGGAAAGTTTCGACCTGGATAGTTTAAAATGCTAG------------------------------------------------------------------------
 3 MT379843.1_X2                  AACGATTACAAAAAGGAAAGTTTCGACCTGGATAGTTTAAAATGCTAG------------------------------------------------------------------------
 4 MT379843.1_X3                  AACGATTACAAAAAGGAAAGTTTCGACCTGGATAGTTTAAAAACAAAAGAACAAAAAAAAACGCTTGACTCAATCACCAAAAAACGCACAAAATTTTGTAACGATGCATTTTTATTGGAA
 5 MW759883.1_B1B-12              AAC--------------------------------------------------------------------------------------------------------------------- SUS
 6 MW759884.1_B3A-12              AACGATTACAAAAAGGAAAGTTTCGACCTGGATAGTTTAAAATGCTAG------------------------------------------------------------------------ SUS
 7 MW759885.1_MN1-4-12            AACGATTACAAAAAGGAAAGTTTCGACCTGGATAGTTTAAAATGCTAG------------------------------------------------------------------------ RES
 8 MW759886.1_MN1-5-12            ------------------------------------------------------------------------------------------------------------------------ RES
 9 MW759887.1_MN1-7-12            AACGATTACAAAAAGGAAAGTTTCGACCTGGATAGTTTAAAATGCTAG------------------------------------------------------------------------ RES
10 MW759888.1_MN2-2-12            ------------------------------------------------------------------------------------------------------------------------ RES
11 MW759889.1_MN2-6-12            ------------------------------------------------------------------------------------------------------------------------ RES
12 MW759890.1_MN2-9-12            ------------------------------------------------------------------------------------------------------------------------ RES
13 MW759892.1_Suth5-12            ------------------------------------------------------------------------------------------------------------------------ RES
14 MW759893.1_Suth6-12            AACGATTACAAAAAGGAAAGTTTCGACCTGGATAGTTTAAAATGCTAG------------------------------------------------------------------------ RES

                                  N  N  Q  L  N  S  S  * ] 1207
                                                         ] 3624
 1 AG6007485-RA                   ------------------------
 2 MT379843.1_X1                  ------------------------
 3 MT379843.1_X2                  ------------------------
 4 MT379843.1_X3                  AACAACCAATTAAACTCGTCCTAG
 5 MW759883.1_B1B-12              ------------------------ SUS
 6 MW759884.1_B3A-12              ------------------------ SUS
 7 MW759885.1_MN1-4-12            ------------------------ RES
 8 MW759886.1_MN1-5-12            ------------------------ RES
 9 MW759887.1_MN1-7-12            ------------------------ RES
10 MW759888.1_MN2-2-12            ------------------------ RES
11 MW759889.1_MN2-6-12            ------------------------ RES
12 MW759890.1_MN2-9-12            ------------------------ RES
13 MW759892.1_Suth5-12            ------------------------ RES
14 MW759893.1_Suth6-12            ------------------------ RES
```

Figure S2. Multiple sequence alignment of *para*-type voltage gated sodium channel (VGSC) proteins from the *Musca domestica* (Md) (GenBank accession AAB47604.1) and putative orthologs encoded by *Aphis glycines*. As in other aphids, the *A. glycines* VGSC a heterodimer. The *A. glycines* VGSC subunit I (VGSC-H1) has two predicted isoforms; a 1174 amino acid (aa) isoform X1 from *Aphis glycines* gene model AG6007485-RA (obtained from Ag_bt1_v6 OGS; Giordano et al. 2020), or a 1150 aa isoform X2 *in silico* translated from a consensus of cDNA sequences obtained in this study (accessions MW759883.1 – MW759893.1). The *A. glycines* VGSC-H1 aligns to the N-terminal region of Md AAB47604.1 with regions orthologous to domains I (DI) and II, each comprised of six α-helical segments (S1-6) that form transmembrane regions (TMRs) of the VGSC protein (Williamson et al., 1996). The *A. glycines* VGSC subunit 2 (VGSC-H2) is a merger of gene models AG6007488-RA and AG6007489-RA based on direct Sanger sequencing of cDNA amplicons (accessions MW759894.1 – MW759904.1). The *A. glycines* VGSC-H2 aligns with Md VGSC regions that form DIII S1-6 and DIV S1-6. Alignment gaps shown as dashes (-), and those involved in splice variants as a plus (+). Methionine (M) residues at start codons are highlighted green. Amino acids mutations involved in knockdown resistance (*kdr*) are shown in black which lead to changes from a leucine to phenylalanine at position 1014 (L1014F) and methionine to threonine at position 918 (M918T) in Md AAB47604.1, respectively.

```
Md_AAB47604.1             M-TED-SDSISEEERSLFRPFTRESLLQIEQRIAEH-EKQKELERKRAA---------EG      48
AG6007485-PA_VGSC-H1_X1   MSIADTDSSFSSEEKSLFRPFTRESLRAIEQRIAEDHAKQKELEEKRAEGEPLPWKRNKN      60
cDNA consensus_VGSC-H1_X2 MSIADTDSSFSSEEKSLFRPFTRESLRAIEQRIAEDHAKQKELEEKRAEGE+++++++++      51


Md_AAB47604.1             EQIRYDDEDEDEGPQPDPTLEQGVPIPVRMQGSFPPELASTPLEDIDPFYSNVLTFVVIS      108
AG6007485-PA_VGSC-H1_X1   KDVRYEDEDEDEGPQPDATLEQGAPLPVRLVGTFPPELASVPLEDIDPYYHNQKTFVVIS      120
cDNA consensus_VGSC-H1_X2 ++VRYEDEDEDEGPQPDATLEQGAPLPVRLVGTFPPELASVPLEDIDPYYHNQKTFVVIS      109

                                                       ........ DI S1.........       .
Md_AAB47604.1             KGKDIFRFSASKAMWLLDPFNPIRRVAIYILVHPLFSLFIITTILTNCILMIMPTTPTVE      168
AG6007485-PA_VGSC-H1_X1   KGKDIFRFSATDGLWALDPFNPIRRVAIYILVHPIFSVTIITTILTNCVFMIMPPTPTIE      180
cDNA consensus_VGSC-H1_X2 KGKDIFRFSATDGLWALDPFNPIRRVAIYILVHPIFSVTIITTILTNCVFMIMPPTPTIE      169

                          .......DI S2.......              .......DI S3.......      ..
Md_AAB47604.1             STEVIFTGIYTFESAVKVMARGFILCPFTYLRDAWNWLDFVVIALAYVTMGIDLGNLAAL      228
AG6007485-PA_VGSC-H1_X1   ASEVIFTGIYTFESAVKVMARGFILEHFTYLRDAWNWLDFIVIALAYVTMGIELGNLAVL      240
cDNA consensus_VGSC-H1_X2 ASEVIFTGIYTFESAVKVMARGFILEHFTYLRDAWNWLDFIVIALAYVTMGIELGNLAVL      229

                          ......DI S4.......                .......DI S5.....
Md_AAB47604.1             RTFRVLRALKTVAIVPGLKTIVGAVIESVKNLRDVIILTMFSLSVFALMGLQIYMGVLTQ      288
AG6007485-PA_VGSC-H1_X1   RTFRVLRALKTVAIVPGLKTIVGAVIESVKNLRDVIILTIFSLSVFALLGLQIYMGVLTQ      300
cDNA consensus_VGSC-H1_X2 RTFRVLRALKTVAIVPGLKTIVGAVIESVKNLRDVIILTIFSLSVFALLGLQIYMGVLTQ      289

Md_AAB47604.1             KCIKRFPLDGSWGNLTDENWFLHNSNSSNWFTEND-GESYPVCGNVSGAGQCGEDYVCLQ      347
AG6007485-PA_VGSC-H1_X1   KCIKYFPLDGSAGNLTNENWFAFMSNKSNWQPNDDEPDEYRLCGNGTGAGQCDEGYMCIQ      360
cDNA consensus_VGSC-H1_X2 KCIKYFPLDGSAGNLTNENWFAFMSNKSNWQPNDDEPDEYRLCGNGTGAGQCDEGYMCIQ      349

                                                                 ......DI S6..
Md_AAB47604.1             GFGPNPNYDYTSFDSFGWAFLSAFRLMTQDFWEDLYQHVLQAAGPWHMLFFIVIIFLGSF      407
AG6007485-PA_VGSC-H1_X1   GFGGNPNYGYTSFDTFAWALLSAFRLMTQDNWEALYQQVLRAAGPWHMFFFIVIIFLGSF      420
cDNA consensus_VGSC-H1_X2 GFGGNPNYGYTSFDTFAWALLSAFRLMTQDNWEALYQQVLRAAGPWHMFFFIVIIFLGSF      409

                          ...DI S6.....
Md_AAB47604.1             YLVNLILAIVAMSYDELQKKAEEEEAAEEEAIREAEEAAAAKAAKLEERANVAAQAAQDA      467
AG6007485-PA_VGSC-H1_X1   YLVNLILAIVAMSYDELQKKAEEEEAAEEEAIREAEQAAKDREVRRQAHEERVAERA---      477
CDNA consensus_VGSC-H1_X2 YLVNLILAIVAMSYDELQKKAEEEEAAEEEAIREAEQAAKDREVRRQAHEERVAERA---      466

Md_AAB47604.1             ADAAAAALHPEMAKSPTYSCISYELFVGGEK----GNDDNNKEKMSIRSVEVESESVSVI      523
AG6007485-PA_VGSC-H1_X1   ERARHVTQHPK--SPSDFSSQSYDNMFGGGQDRGIGNDHHREKNMSLRSMSITSHDKH--      533
cDNA consensus_VGSC-H1_X2 ERARHVTQHPK--SPSDFSSQSYDNMFGGGQDRGIGNDHHREKNMSLRSMSITSHDKH--      522
Md_AAB47604.1             QRQPAPTTAPATKVRKVSTTSLSLPGSPFNLRRGSRSSHKYTIRNGRGRFGIPGSDRKPL      583
AG6007485-PA_VGSC-H1_X1   ----SDTGSVDRQSGKTRKASLSLPGSPFNIRRASRGSHQLSHRNGRPRF--TGADTKPL      587
cDNA consensus_VGSC-H1_X2 ----SDTGSVDRQSGKTRKASLSLPGSPFNIRRASRGSHQLSHRNGRPRF--TGADTKPL      576
```

```
Md_AAB47604.1               VLQTYQDAQQHLPYADDSNAVTPMSEENGAIIVPAYYCNLGSRHSSYTSHQSRISYTSHG      643
AG6007485-PA_VGSC-H1_X1     VLNTLLDAEEHLPYADDSNAVTPMSEENGAIIVPVSYANFGSRHSSYTSHTSRITYTSHA      647
cDNA consensus_VGSC-H1_X2   VLNTLLDAEEHLPYADDSNAVTPMSEENGAIIVPVSYANFGSRHSSYTSHTSRITYTSHA      636


Md_AAB47604.1               DLLGGMAAMGASTMTKESKLRSRNTRNQSIGAATNGGSSTAGGGYPDANHKEQRDYEMGQ      703
AG6007485-PA_VGSC-H1_X1     DLFKP-------PMTKERQLRSRSARNYFNPSD--------------------QRYHRDD      680
cDNA consensus_VGSC-H1_X2   DLFKP-------PMTKERQLRSRSARNYFNPSD--------------------QRYHRDD      669

Md_AAB47604.1               DYTDEA--GKIKHHDNPFIEPVQTQTVVDMKDVMVLNDIIEQAAGRHSRASERG------      755
AG6007485-PA_VGSC-H1_X1     DYDTSSMSKSKQKVDECGYSDSQKHTVVDMRDVMVLNDIIEQAAGRQSRGSEKAVITGGT      740
cDNA consensus_VGSC-H1_X2   DYDTSSMSKSKQKVDECGYSDSQKHTVVDMRDVMVLNDIIEQAAGRQSRGSEKA++++++      723


Md_AAB47604.1               -------------------------EDDDEDGPTFKDIALEYILKGIEIFCVWDCCWVWL      790
AG6007485-PA_VGSC-H1_X1     LVGAWGTVSTVYVFPTDEDAVDGEDEEEENEEPTFREKFQVWLLKFIDTFCVWDCGWPWL      800
cDNA consensus_VGSC-H1_X2   +++++++VSTVYVFPTDEDAVDGEDEEEENEEPTFREKFQVWLLKFIDTFCVWDCGWPWL      776

                                   .........DII S1..........            .........DII S2..
Md_AAB47604.1               KFQEWVSFIVFDPFVELFITLCIVVNTMFMAMDHHDMNPELEKVLKSGNYFFTATFAIEA      850
AG6007485-PA_VGSC-H1_X1     KFQQGLAFIVFDPFVELYITLCIVVNTLFMALDHHEMDPKLDFILNKANVFFSATFGVEA      860
cDNA consensus_VGSC-H1_X2   KFQQGLAFIVFDPFVELYITLCIVVNTLFMALDHHEMDPKLDFILNKANVFFSATFGVEA      836

                            .......       .....DII S3........       .........DI S4.....
Md_AAB47604.1               SMKLMAMSPKYYFQEGWNIFDFIIVALSLLELGLEGVQGLSVLRSFRLLRVFKLAKSWPT      910
AG6007485-PA_VGSC-H1_X1     ALKLMAMSPKYYFQMGWNIFDFIIVILSVVELLSAGYQGLSVLRSFRLLRVFKLAKSWPT      920
cDNA consensus_VGSC-H1_X2   ALKLMAMSPKYYFQMGWNIFDFIIVILSVVELLSAGYQGLSVLRSFRLLRVFKLAKSWPT      896

                            .                ........DII S5.......
Md_AAB47604.1               LNLLISIMGRTMGALGNLTFVLCIIIFIFAVMGMQLFGKNYIDHKDRFKDHELPRWNFTD      970
AG6007485-PA_VGSC-H1_X1     LNLLISIMGRTIGALGNLTFVLCIIIFIFAVMGMQLFGKNYTEKMYLFKDHELPRWNFTD      980
cDNA consensus_VGSC-H1_X2   LNLLISIMGRTIGALGNLTFVLCIIIFIFAVMGMQLFGKNYTEKMYLFKDHELPRWNFTD      956

                                                        ..........DII S6..........
Md_AAB47604.1               FMHSFMIVFRVLCGEWIESMWDCMYVGDVSCIPFFLATVVIGNLVVLNLFLALLLSNFGS      1030
AG6007485-PA_VGSC-H1_X1     FLHSFMIVFRVLCGEWIESMWDCLHVGEPTCIPFFLATVVIGNLVVLNLFLALLLSNFGS      1040
cDNA consensus_VGSC-H1_X2   FLHSFMIVFRVLCGEWIESMWDCLHVGEPTCIPFFLATVVIGNLVVLNLFLALLLSNFGS      1016


Md_AAB47604.1               SSLSAPTADNDTNKIAEAFNRIARFKNWVKRNIADCFKLIRNKLTNQISDQPSEHGDNEL      1090
AG6007485-PA_VGSC-H1_X1     SNLSVPTADSDTNKITEAFERIGRFNKWVKTHILNFLKVLRLKITNQISVQVSGRD-RDI      1099
cDNA consensus_VGSC-H1_X2   SNLSVPTADSDTNKITEAFERIGRFNKWVKTHILNFLKVLRLKITNQISVQVSGRD-RDI      1075


Md_AAB47604.1               ELGHDEIMGDGLIKKGMKGETQLEVAIGDGMEFTIHGDMKNNKPKKSKFMNNTTMIGNSI      1150
AG6007485-PA_VGSC-H1_X1     DLPVDETIVDV-IAPFKDTKEPVEMTIGDGMEFTIPGDVKQKIKKNQ--------VGNSI      1150
cDNA consensus_VGSC-H1_X2   DLPVDETIVDV-IAPFKDTKEPVEMTIGDGMEFTIPGDVKQKIKKNQ--------VGNSI      1126
cDNA consensus_VGSC2        MSVYSSEELLDAGIIYRNKKEQLDVTIGDGMELLIRGEKNKKKKPPTSSS------YNSF        54
AG6007489-PA                MSVYSSEELLDAGIIYRNKKEQLDVTIGDGMELLIRGEKNKKKKPPTSSS------YNSF        54

Md_AAB47604.1               N-HQDNRLEHELNHRGLSIQDDDTASINSYGSHKNRPFKDESHKGSAETIEGEE-KRDVS      1208
AG6007485-PA_VGSC-H1_X1     GNHQGNKVGNDYK---KESFDLD--SLKC-------------------------------      1174
CDNA consensus_VGSC-H1_X2   GNLQGNKVGNDYK---KESFDLD--SLKC-------------------------------      1150
cDNA consensus_VGSC2        GMHQSS---TDENYYLKDKYEYDTRSTKSYGSHEHDPYDSESHRGSKRSLHNAEEKKDPS       111
AG6007489-PA                GMHQSS---TDENYYLKDKYEYDTRSTKSYGSHEHDPYDSESHRGSKRSLHNAEEKKDPS       111


Md_AAB47604.1               KEDLGLDEELD--------EEAEGDEGQLDGDIIIHAQNDDEIIDDYPADCFPDSYYKKF      1260
AG6007485-PA_VGSC-H1_X1     ------------------------------------------------------------      1174
cDNA consensus_VGSC-H1_X2   ------------------------------------------------------------      1150
cDNA consensus_VGSC2        NEDVETNQNILGGHNGIAAEELNGEEYKEQGPV--EMVEDVFEEEEYPEDCFPPNCYKKF       168
AG6007489-PA                NEDVETNQNILGGHNGIAAEELNGEEYKEQGPV--EMVEDVFEEEEYPEDCFPPNCYKKF       169

                                                      .......DIII S1.......
Md_AAB47604.1               PILAGDEDSPFWQGWGNLRLKTFQLIENKYFETAVITMILMSSLALALEDVHLPDRPVMQ      1320
AG6007485-PA_VGSC-H1_X1     ------------------------------------------------------------      1174
cDNA consensus_VGSC-H1_X2   ------------------------------------------------------------      1150
cDNA consensus_VGSC2        PFLAGDDETPFWLGWGQLRLKTFQLIENKYFETAVITMILLSSLALALEDVHLQKRPVLQ       228
AG6007489-PA                PFLAGDDETPFWLGWGQLRLKTFQLIENKYFETAVITMILLSSLALALEDVHLQKRPVLQ       229


                             .........DIII S2.........       .......DIII S3........    .
Md_AAB47604.1               DILYYMDRIFTVIFFLEMLIKWLALGFKVYFTNAWCWLDFVIVMLSLINLVAVWSGLNDI      1380
AG6007485-PA_VGSC-H1_X1     ------------------------------------------------------------      1174
cDNA consensus_VGSC-H1_X2   ------------------------------------------------------------      1150
cDNA consensus_VGSC2        DILYYMDRIFTVIFFLEMLIKWLALGFRNYFTNAWCWLDFIIVMVSLINFVAALLGASGI       288
```

```
AG6007489-PA               DILYYMDRIFTVIFFLEMLIKWLALGFRNYFTNAWCWLDFIIVMLSLINFVAALLGASGI      330


                           .......DIII S4.......                   .......DIII S5.......
Md_AAB47604.1              AVFRSMRTLRALRPLRAVSRWEGMKVVVNALVQAIPSIFNVLLVCLIFWLIFAIMGVQLF     1440
AG6007485-PA_VGSC-H1_X1    ------------------------------------------------------------     1174
CDNA consensus_VGSC-H1_X2  ------------------------------------------------------------     1150
CDNA consensus_VGSC2       QAFKTMRTLRALRPLRAMARMQGMRVVVNALVQAIPSIFNVLLVCLIFWLIFAIMGVQLF      348
AG6007489-PA               QAFKTMRTLRALRPLRAMARMQGMRVCDR                                     359
AG6007488-PA                                    MVLYQVVVNALVQAIPSIFNVLLVCLIFWLIFAIMGVQLF       40


                           ..
Md_AAB47604.1              AGKYFKCKDGNDTVLSHEIIPNRNACKSENYTWENSAMNFDHVGNAYLCLFQVATFKGWI     1500
AG6007485-PA_VGSC-H1_X1    ------------------------------------------------------------     1174
cDNA consensus_VGSC-H1_X2  ------------------------------------------------------------     1150
cDNA consensus_VGSC2       AGKYYKCVDNTGKTLNHEIIPDKNVCLAENYKWENSKMNFDHVGNAYLCLFQVATFNGWM      408
AG6007488-PA               AGKYYKCVDNTGKTLNHEIIPDKNVCLAENYKWENSKMNFDHVGNAYLCLFQVATFNGWM      100

                                                  .........DIII S6..........
Md_AAB47604.1              QIMNDAIDSREV-DKQPIRETNIYMYLYFVFFIIFGSFFTLNLFIGVIIDNFNEQKKKAG     1559
AG6007485-PA_VGSC-H1_X1    ------------------------------------------------------------     1174
cDNA consensus_VGSC-H1_X2  ------------------------------------------------------------     1150
cDNA consensus_VGSC2       EIMRDAVDSRDTHGKQPIREINNYMYFYFVFFIIFGSFFTLNLFIGVIIDNFNEQKKKTG      468
AG6007488-PA               EIMRDAVDSRDTHGKQPIREINNYMYFYFVFFIIFGSFFTLNLFIGVIIDNFNEQKKKTG      160

                                                                      .......DIV S1....
Md_AAB47604.1              GSLEMFMTEDQKKYYNAMKKMGSKKPLKAIPRPRWRPQAIVFEIVTDKKFDIIIMLFIGL     1619
AG6007485-PA_VGSC-H1_X1    ------------------------------------------------------------     1174
cDNA consensus_VGSC-H1_X2  ------------------------------------------------------------     1150
cDNA consensus_VGSC2       ASLEMFMTEDQKKYYNAMKKMSSKKPLKAIPRPRWRPQSIVFQIVTDKKFDMLIMLFIGL      528
AG6007488-PA               ASLEMFMTEDQKKYYNAMKKMSSKKPLKAIPRPRWRPQSIVFQIVTDKKFDMLIMLFIGL      220

                           .......            .........DIV S2.........      ......DIV S3
Md_AAB47604.1              NMFTMTLDRYDASEAYNNVLDKLNGIFVVIFSGECLLKIFALRYHYFKEPWNLFDVVVVI     1679
AG6007485-PA_VGSC-H1_X1    ------------------------------------------------------------     1174
cDNA consensus_VGSC-H1_X2  ------------------------------------------------------------     1150
cDNA consensus_VGSC2       NMLTMTLDHYQQTKLFTDVLERLNQIFIAIFSTECLLKIFALRYYYFKEPWNLFDFVVVI      588
AG6007488-PA               NMLTMTLDHYQQTKLFTDVLERLNQIFIAIFSTECLLKIFALRYYYFKEPWNLFDFVVVI      280


                           ............            ........DIV S4........               .
Md_AAB47604.1              LSILGLVLSDIIEKYFVSPTLLRVVRVAKVGRVLRLVKGAKGIRTLLFALAMSLPALFNI     1739
AG6007485-PA_VGSC-H1_X1    ------------------------------------------------------------     1174
cDNA consensus_VGSC-H1_X2  ------------------------------------------------------------     1150
cDNA consensus_VGSC2       LSLAGLVLSDLISKYFVSPTLLRVVRVAKVGRVLRLVKGAKGIRTLLFALAMSLPALFNI      648
AG6007488-PA               LSLAGLVLSDLISKYFVSPTLLRVVRVAKVGRVLRLVKGAKGIRTLLFALAMSLPALFNI      340

                           ........DIV S5........
Md_AAB47604.1              CLLLFLVMFIFAIFGMSFFMHVKEKSGINAVYNFKTFGQSMILLFQMSTSAGWDGVLDAI     1799
AG6007485-PA_VGSC-H1_X1    ------------------------------------------------------------     1174
cDNA consensus_VGSC-H1_X2  ------------------------------------------------------------     1150
cDNA consensus_VGSC2       CLLLFLVMFIFAIFGMSFFMNVDNHGGLDEDYNFRTFGQSMILLFMLSTSSGWDAVLDGI      708
AG6007488-PA               CLLLFLVMFIFAIFGMSFFMNVDNHGGLDEDYNFRTFGQSMILLFMLSTSSGWDAVLDGI      400

                                                  ..........DIV S6..........
Md_AAB47604.1              INEEDCDPPDNDKGYPGNCGSATVGITFLLSYLVISFLIVINMYIAVILENYSQATEDVQ     1859
AG6007485-PA_VGSC-H1_X1    ------------------------------------------------------------     1174
cDNA consensus_VGSC-H1_X2  ------------------------------------------------------------     1150
cDNA consensus_VGSC2       TNEDDCDKPNLEMGITGSCGSSAVGTAFLLSYLVINFLIVINMYIAVILENYSQATEDVQ      768
AG6007488-PA               TNEDDCDKPNLEMGITGSCGSSAVGTAFLLSYLVINFLIVINMYIAVILENYSQATEDVQ      460


Md_AAB47604.1              EGLTDDDYDMYYEIWQQFDPEGTQYIRYDQLSEFLDVLEPPLQIHKPNKYKIISMDMPIC     1919
AG6007485-PA_VGSC-H1_X1    ------------------------------------------------------------     1174
cDNA consensus_VGSC-H1_X2  ------------------------------------------------------------     1150
cDNA consensus_VGSC2       EGLTDDDYDMYYEIWQHFDPNGTRYIRYDQLSDFLDILEPPLKIHKPNKYKIVSMDIPIC      828
AG6007488-PA               EGLTDDDYDMYYEIWQHFDPNGTRYIRYDQLSDFLDILEPPLKIHKPNKYKIVSMDIPIC      520

Md_AAB47604.1              RGDMMYCVDILDALTKDFFARKGNPIEE-TGEIGEIAARPDTEGYDPVSSTLWRQREEYC     1978
AG6007485-PA_VGSC-H1_X1    ------------------------------------------------------------     1174
cDNA consensus_VGSC-H1_X2  ------------------------------------------------------------     1150
cDNA consensus_VGSC2       KGDLIYCVDILDALTKDFFARKGNPIIETVAEIGEMQTRPEEAGYEPISSSLWRMREVYC      888
AG6007488-PA               KGDLIYCVDILDALTKDFFARKGNPIIETVAEIGEMQTRPEEAGYEPISSSLWRMREVYC      580
```

```
Md_AAB47604.1              AKLIQNAWRRYKNGPPQEGDEGEAAGGEDGAEGGEGEGGSGGGGGDDGGSATGATAAAGA      2038
AG6007485-PA_VGSC-H1_X1    ------------------------------------------------------------      1174
cDNA consensus_VGSC-H1_X2  ------------------------------------------------------------      1150
cDNA consensus_VGSC2       ATIIQSSWRKYAAAAKQQTADDDRSDG---------------------------------       915
AG6007488-PA               ATIIQSSWRKYAAAAKQQTADDDRSDG---------------------------------       607

Md_AAB47604.1              TSPSDPDAGEADGASVGGPLSPGCVSGGSNGRQTAVLVESDGFVTKNGHKVVIHSRSPSI      2098
AG6007485-PA_VGSC-H1_X1    ------------------------------------------------------------      1174
cDNA consensus_VGSC-H1_X2  ------------------------------------------------------------      1150
cDNA consensus_VGSC2       -------------------------AASPDGRETAVLVESDGFVTKNGHKVVIHSRSSSK       951
AG6007488-PA               -------------------------AASPDGRETAVLVESDGFVTKNGHKVVIHSRSSSK       642

Md_AAB47604.1              TSRTADV       2105
AG6007485-PA_VGSC-H1_X1    -------       1174
cDNA consensus_VGSC-H1_X2  -------       1150
cDNA consensus_VGSC2       SSRLADV        958
AG6007488-PA               SSRLADV        649
```

Figure S3. Multiple amino acid (aa) sequence alignment among voltage gated sodium channel heterodimer 1 (VGSC-H1) isoforms among species of aphid (Family Aphididae). The 1220 aa consensus alignment was constructed among translations among *Aphis glycines* (Aglycines), *Acyrthosiphon pisum* (Apisum), *Aphis gossypii* (Agossypii), *Melanaphis sacchari* (Msacchari), *Rhopalosiphum maidis* (Rmaidis), *Myzus persicae* (Mpersicae), *Diuraphis noxia* (Dnoxia), and *Sipha flava* (Sflava), appended to RefSeq accessions where available. The *A. glycines* sequence AG6007485-PA corresponds to the gene model in the AgBt1_v6.0 assembly (Giordano et al. 2020), and MW759885.1 is a representative from among cDNA sequences in this study (GenBank accessions MW759883.1 – MW759893.1; **Figure 1A**). Putative structural annotation of domain I and II (D1 and DII) each comprised of six transmembrane α-helical segments (S1-6) are highlighted grey based on orthology between the *A. glycines* protein model AG6007485-PA and the *Musca domestica* VGSC (GenBank accession AAB47604.1; Williamson et al., 1996; **Figure S1**).

```
                              1 [       .         .         .         .         :         .         .         .         .         1 100
AG6007485-PA_Aglycines         MSIADTDSSFSSEEKSLFRPFTRESLRAIEQRIAEDHAKQKELEEKRAEGEPLPWKRNKNKDVRYEDEDEDEGPQPDATLEQGAPLPVRLVGTFPPELAS
QTJ01840.1_X1_Aglycines        MSIADTDSSFSSEEKSLFRPFTRESLRAIEQRIAEDHAKQKELEEKRAEGEPLPWKRNKNKDVRYEDEDEDEGPQPDATLEQGAPLPVRLVGTFPPELAS
QTJ01838.1_X2_Aglycines        MSIADTDSSFSSEEKSLFRPFTRESLRAIEQRIAEDHAKQKELEEKRAEGE-----------VRYEDEDEDEGPQPDATLEQGAPLPVRLVGTFPPELAS
QTJ01839.1_X3_Aglycines        MSIADTDSSFSSEEKSLFRPFTRESLRAIEQRIAEDHAKQKELEEKRAEGE-----------VRYEDEDEDEGPQPDATLEQGAPLPVRLVGTFPPELAS
MW759885.1_X4_Aglycines        MSIADTDSSFSSEEKSLFRPFTRESLRAIEQRIAEDHAKQKELEEKRAEGE-----------VRYEDEDEDEGPQPDATLEQGAPLPVRLVGTFPPELAS
XP_016660066.1_X1_Apisum       MSIADTDSSFSSEEKSLFRPFTRESLRAIEQRIAEDHAKQKELEEKRAEGEPLPWKRNKNKDVRYEDEDEDEGPQPDATLEQGAPLPVRLVGTFPPELAS
XP_016660067.1_X2_Apisum       MSIADTDSSFSSEEKSLFRPFTRESLRAIEQRIAEDHAKQKELEEKRAEGEPLPWKRNKNKDVRYEDEDEDEGPQPDATLEQGAPLPVRLVGTFPPELAS
XP_029346126.1_X3_Apisum       MSIADTDSSFSSEEKSLFRPFTRESLRAIEQRIAEDHAKQKELEEKRAEGE-----------VRYEDEDEDEGPQPDATLEQGAPLPVRLVGTFPPELAS
XP_029346127.1_X4_Apisum       MSIADTDSSFSSEEKSLFRPFTRESLRAIEQRIAEDHAKQKELEEKRAEGE-----------VRYEDEDEDEGPQPDATLEQGAPLPVRLVGTFPPELAS
XP_027840099.1_X1_Agossypii MSIADTDSSFSSEEKSLFRPFTRESLRAIEQRIAEDHAKQKELEEKRAEGEPLPWKRNKNKDVRYEDEDEDEGPQPDATLEQGAPLPVRLVGTFPPELAS
XP_027840100.1_X2_Agossypii MSIADTDSSFSSEEKSLFRPFTRESLRAIEQRIAEDHAKQKELEEKRAEGE-----------VRYEDEDEDEGPQPDATLEQGAPLPVRLVGTFPPELAS
XP_027840101.1_X3_Agossypii MSIADTDSSFSSEEKSLFRPFTRESLRAIEQRIAEDHAKQKELEEKRAEGE-----------VRYEDEDEDEGPQPDATLEQGAPLPVRLVGTFPPELAS
XP_025206080.1_X1_Msacchari MSIADTDSSFSSEEKSLFRPFTRESLRAIEQRIAEDHAKQKELEEKRAEGEPLPWKRNKNKDVRYEDEDEDEGPQPDATLEQGAPLPVRLVGTFPPELAS
XP_025206081.1_X2_Msacchari MSIADTDSSFSSEEKSLFRPFTRESLRAIEQRIAEDHAKQKELEEKRAEGEPLPWKRNKNKDVRYEDEDEDEGPQPDATLEQGAPLPVRLVGTFPPELAS
XP_025206082.1_X3_Msacchari MSIADTDSSFSSEEKSLFRPFTRESLRAIEQRIAEDHAKQKELEEKRAEGE-----------VRYEDEDEDEGPQPDATLEQGAPLPVRLVGTFPPELAS
XP_025206083.1_X4_Msacchari MSIADTDSSFSSEEKSLFRPFTRESLRAIEQRIAEDHAKQKELEEKRAEGE-----------VRYEDEDEDEGPQPDATLEQGAPLPVRLVGTFPPELAS
XP_026806608.1_X1_Rmaidis      MSIADTDSSFSSEEKSLFRPFTRESLRAIEQRIAEDHAKQKELEEKRAEGEPLPWKRNKNKDVRYEDEDEDEGPQPDATLEQGAPLPVRLVGTFPPELAS
XP_026806610.1_X2_Rmaidis      MSIADTDSSFSSEEKSLFRPFTRESLRAIEQRIAEDHAKQKELEEKRAEGEPLPWKRNKNKDVRYEDEDEDEGPQPDATLEQGAPLPVRLVGTFPPELAS
XP_026806611.1_X3_Rmaidis      MSIADTDSSFSSEEKSLFRPFTRESLRAIEQRIAEDHAKQKELEEKRAEGE-----------VRYEDEDEDEGPQPDATLEQGAPLPVRLVGTFPPELAS
XP_026806612.1_X4_Rmaidis      MSIADTDSSFSSEEKSLFRPFTRESLRAIEQRIAEDHAKQKELEEKRAEGE-----------VRYEDEDEDEGPQPDATLEQGAPLPVRLVGTFPPELAS
XP_022174236.1_X1_Mpersicae MSIADTDSSFSSEEKSLFRPFTRDSLRAIEQRIAEDHAKQKELEEKRAEGEPLPWKRNKNKDVRYEDEDEDEGPQPDATLEQGAPLPVRLVGTFPPELAS
XP_022174237.1_X2_Mpersicae MSIADTDSSFSSEEKSLFRPFTRDSLRAIEQRIAEDHAKQKELEEKRAEGE-----------VRYEDEDEDEGPQPDATLEQGAPLPVRLVGTFPPELAS
XP_022174238.1_X3_Mpersicae MSIADTDSSFSSEEKSLFRPFTRDSLRAIEQRIAEDHAKQKELEEKRAEGE-----------VRYEDEDEDEGPQPDATLEQGAPLPVRLVGTFPPELAS
XP_022174239.1_X4_Mpersicae MSIADTDSSFSSEEKSLFRPFTRDSLRAIEQRIAEDHAKQKELEEKRAEGE-----------VRYEDEDEDEGPQPDATLEQGAPLPVRLVGTFPPELAS
XP_015366149.1_X1_Dnoxia       MSIADTDSSFSSEEKSLFRPFTRDSLRAIEQRIAEDHAKQKELEEKRAEGE-----------VRYEDEDEDEGPQPDATLEQGAPLPVRLVGTFPPELAS
XP_015366150.1_X2_Dnoxia       MSIADTDSSFSSEEKSLFRPFTRDSLRAIEQRIAEDHAKQKELEEKRAEGE-----------VRYEDEDEDEGPQPDATLEQGAPLPVRLVGTFPPELAS
XP_015366151.1_X3_Dnoxia       MSIADTDSSFSSEEKSLFRPFTRDSLRAIEQRIAEDHAKQKELEEKRAEGE-----------VRYEDEDEDEGPQPDATLEQGAPLPVRLVGTFPPELAS
XP_015366152.1_X4_Dnoxia       MSIADTDSSFSSEEKSLFRPFTRDSLRAIEQRIAEDHAKQKELEEKRAEGE-----------VRYEDEDEDEGPQPDATLEQGAPLPVRLVGTFPPELAS
XP_025407763.1_X1_Sflava       MSIADTDSSFSSEEKSLFRPFTRESLRAIEQRIAEEHAKQKELEEKRAEGEPLPWKKNKNKDIRYEDEDEDEGPQPDATLEQGAPLPVRLVGTFPPELAS
XP_025407764.1_X2_Sflava       MSIADTDSSFSSEEKSLFRPFTRESLRAIEQRIAEEHAKQKELEEKRAEGEPLPWKKNKNKDIRYEDEDEDEGPQPDATLEQGAPLPVRLVGTFPPELAS
XP_025407765.1_X3_Sflava       MSIADTDSSFSSEEKSLFRPFTRESLRAIEQRIAEEHAKQKELEEKRAEGEPLPWKKNKNKDIRYEDEDEDEGPQPDATLEQGAPLPVRLVGTFPPELAS
XP_025407766.1_X4_Sflava       MSIADTDSSFSSEEKSLFRPFTRESLRAIEQRIAEEHAKQKELEEKRAEG-----------EIRYEDEDEDEGPQPDATLEQGAPLPVRLVGTFPPELAS
XP_025407767.1_X5_Sflava       MSIADTDSSFSSEEKSLFRPFTRESLRAIEQRIAEEHAKQKELEEKRAEG-----------EIRYEDEDEDEGPQPDATLEQGAPLPVRLVGTFPPELAS
XP_025407768.1_X6_Sflava       MSIADTDSSFSSEEKSLFRPFTRESLRAIEQRIAEEHAKQKELEEKRAEGEPLPWKKNKNKDIRYEDEDEDEGPQPDATLEQGAPLPVRLVGTFPPELAS
```

```
                                                                        ...........DI S1..........          .........DI S2.........
                            101         .         .         .         .         :         .         .         .         .         2 200
AG6007485-PA_Aglycines       VPLEDIDPYYHNQKTFVVISKGKDIFRFSATDGLWALDPFNPIRRVAIYILVHPIFSVTIITTILTNCVFMIMPPTPTIEASEVIFTGIYTFESAVKVMA
QTJ01840.1_X1_Aglycines      VPLEDIDPYYHNQKTFVVISKGKDIFRFSATDGLWALDPFNPIRRVAIYILVHPIFSVTIITTILTNCVFMIMPPTPTIEASEVIFTGIYTFESAVKVMA
QTJ01838.1_X2_Aglycines      VPLEDIDPYYHNQKTFVVISKGKDIFRFSATDGLWALDPFNPIRRVAIYILVHPIFSVTIITTILTNCV--------------VIFTGIYTFESAVKVMA
QTJ01839.1_X3_Aglycines      VPLEDIDPYYHNQKTFVVISKGKDIFRFSATDGLWALDPFNPIRRVAIYILVHPIFSVTIITTILTNCVFMIMPPTPTIEASEVIFTGIYTFESAVKVMA
MW759885.1_X4_Aglycines      VPLEDIDPYYHNQKTFVVISKGKDIFRFSATDGLWALDPFNPIRRVAIYILVHPIFSVTIITTILTNCVFMIMPPTPTIEASEVIFTGIYTFESAVKVMA
XP_016660066.1_X1_Apisum     VPLEDIDPYYHNQKTFVVISKGKDIFRFSATDGLWALDPFNPIRRVAIYILVHPIFSVTIITTILTNCVFMIMPPTPTIEASEVIFTGIYTFESAVKVMA
XP_016660067.1_X2_Apisum     VPLEDIDPYYHNQKTFVVISKGKDIFRFSATDGLWALDPFNPIRRVAIYILVHPIFSVTIITTILTNCVFMIMPPTPTIEASEVIFTGIYTFESAVKVMA
XP_029346126.1_X3_Apisum     VPLEDIDPYYHNQKTFVVISKGKDIFRFSATDGLWALDPFNPIRRVAIYILVHPIFSVTIITTILTNCVFMIMPPTPTIEASEVIFTGIYTFESAVKVMA
XP_029346127.1_X4_Apisum     VPLEDIDPYYHNQKTFVVISKGKDIFRFSATDGLWALDPFNPIRRVAIYILVHPIFSVTIITTILTNCVFMIMPPTPTIEASEVIFTGIYTFESAVKVMA
XP_027840099.1_X1_Agossypii VPLEDIDPYYHNQKTFVVISKGKDIFRFSATDGLWALDPFNPIRRVAIYILVHPIFSVTIITTILTNCVFMIMPPTPTIEASEVIFTGIYTFESAVKVMA
XP_027840100.1_X2_Agossypii VPLEDIDPYYHNQKTFVVISKGKDIFRFSATDGLWALDPFNPIRRVAIYILVHPIFSVTIITTILTNCVFMIMPPTPTIEASEVIFTGIYTFESAVKVMA
XP_027840101.1_X3_Agossypii VPLEDIDPYYHNQKTFVVISKGKDIFRFSATDGLWALDPFNPIRRVAIYILVHPIFSVTIITTILTNCVFMIMPPTPTIEASEVIFTGIYTFESAVKVMA
XP_025206080.1_X1_Msacchari VPLEDIDPYYHNQKTFVVISKGKDIFRFSATDGLWALDPFNPIRRVAIYILVHPIFSVTIITTILTNCVFMIMPPTPTIEASEVIFTGIYTFESAVKVMA
XP_025206081.1_X2_Msacchari VPLEDIDPYYHNQKTFVVISKGKDIFRFSATDGLWALDPFNPIRRVAIYILVHPIFSVTIITTILTNCVFMIMPPTPTIEASEVIFTGIYTFESAVKVMA
XP_025206082.1_X3_Msacchari VPLEDIDPYYHNQKTFVVISKGKDIFRFSATDGLWALDPFNPIRRVAIYILVHPIFSVTIITTILTNCVFMIMPPTPTIEASEVIFTGIYTFESAVKVMA
XP_025206083.1_X4_Msacchari VPLEDIDPYYHNQKTFVVISKGKDIFRFSATDGLWALDPFNPIRRVAIYILVHPIFSVTIITTILTNCVFMIMPPTPTIEASEVIFTGIYTFESAVKVMA
XP_026806608.1_X1_Rmaidis    VPLEDIDPYYHNQKTFVVISKGKDIFRFSATDGLWALDPFNPIRRVAIYILVHPIFSVTIITTILTNCVFMIMPPTPTIEASEVIFTGIYTFESAVKVMA
XP_026806610.1_X2_Rmaidis    VPLEDIDPYYHNQKTFVVISKGKDIFRFSATDGLWALDPFNPIRRVAIYILVHPIFSVTIITTILTNCVFMIMPPTPTIEASEVIFTGIYTFESAVKVMA
XP_026806611.1_X3_Rmaidis    VPLEDIDPYYHNQKTFVVISKGKDIFRFSATDGLWALDPFNPIRRVAIYILVHPIFSVTIITTILTNCVFMIMPPTPTIEASEVIFTGIYTFESAVKVMA
XP_026806612.1_X4_Rmaidis    VPLEDIDPYYHNQKTFVVISKGKDIFRFSATDGLWALDPFNPIRRVAIYILVHPIFSVTIITTILTNCVFMIMPPTPTIEASEVIFTGIYTFESAVKVMA
XP_022174236.1_X1_Mpersicae VPLEDIDPYYHNQKTFVVISKGKDIFRFSATDGLWALDPFNPIRRVAIYILVHPIFSVTIITTILTNCVFMIMPPTPTIEASEVIFTGIYTFESAVKVMA
XP_022174237.1_X2_Mpersicae VPLEDIDPYYHNQKTFVVISKGKDIFRFSATDGLWALDPFNPIRRVAIYILVHPIFSVTIITTILTNCVFMIMPPTPTIEASEVIFTGIYTFESAVKVMA
XP_022174238.1_X3_Mpersicae VPLEDIDPYYHNQKTFVVISKGKDIFRFSATDGLWALDPFNPIRRVAIYILVHPIFSVTIITTILTNCVFMIMPPTPTIEASEVIFTGIYTFESAVKVMA
XP_022174239.1_X4_Mpersicae VPLEDIDPYYHNQKTFVVISKGKDIFRFSATDGLWALDPFNPIRRVAIYILVHPIFSVTIITTILTNCVFMIMPPTPTIEASEVIFTGIYTFESAVKVMA
XP_015366149.1_X1_Dnoxia     VPLEDIDPYYHNQKTFVVISKGKDIFRFSATDGLWALDPFNPIRRVAIYILVHPIFSVTIITTILTNCVFMIMPPTPTIEASEVIFTGIYTFESAVKVMA
XP_015366150.1_X2_Dnoxia     VPLEDIDPYYHNQKTFVVISKGKDIFRFSATDGLWALDPFNPIRRVAIYILVHPIFSVTIITTILTNCVFMIMPPTPTIEASEVIFTGIYTFESAVKVMA
XP_015366151.1_X3_Dnoxia     VPLEDIDPYYHNQKTFVVISKGKDIFRFSATDGLWALDPFNPIRRVAIYILVHPIFSVTIITTILTNCVFMIMPPTPTIEASEVIFTGIYTFESAVKVMA
XP_015366152.1_X4_Dnoxia     VPLEDIDPYYHNQKTFVVISKGKDIFRFSATDGLWALDPFNPIRRVAIYILVHPIFSVTIITTILTNCVFMIMPPTPTIEAS------------------
XP_025407763.1_X1_Sflava     VPLEDIDPYYHNQKTFVVISKGKDIFRFSATDGLWALDPFNPIRRVAIYILVHPIFSVTIITTILTNCVFMIMPPTPTIEASEVIFTGIYTFESAVKVMA
XP_025407764.1_X2_Sflava     VPLEDIDPYYHNQKTFVVISKGKDIFRFSATDGLWALDPFNPIRRVAIYILVHPIFSVTIITTILTNCVFMIMPPTPTIEASEVIFTGIYTFESAVKVMA
XP_025407765.1_X3_Sflava     VPLEDIDPYYHNQKTFVVISKGKDIFRFSATDGLWALDPFNPIRRVAIYILVHPIFSVTIITTILTNCVFMIMPPTPTIEASEVIFTGIYTFESAVKVMA
XP_025407766.1_X4_Sflava     VPLEDIDPYYHNQKTFVVISKGKDIFRFSATDGLWALDPFNPIRRVAIYILVHPIFSVTIITTILTNCVFMIMPPTPTIEASEVIFTGIYTFESAVKVMA
XP_025407767.1_X5_Sflava     VPLEDIDPYYHNQKTFVVISKGKDIFRFSATDGLWALDPFNPIRRVAIYILVHPIFSVTIITTILTNCVFMIMPPTPTIEASEVIFTGIYTFESAVKVMA
XP_025407768.1_X6_Sflava     VPLEDIDPYYHNQKTFVVISKGKDIFRFSATDGLWALDPFNPIRRVAIYILVHPIFSVTIITTILTNCVFMIMPPTPTIEAS------------------
```

```
                                           ........DI S3..........     .........DI S4...........          .........DI S5.........
                                201          .         .         .         .         :         .         .         .         .       3 300
AG6007485-PA_Aglycines          RGFILEHFTYLRDAWNWLDFIVIALAYVTMGIELGNLAVLRTFRVLRALKTVAIVPGLKTIVGAVIESVKNLRDVIILTIFSLSVFALLGLQIYMGVLTQ
QTJ01840.1_X1_Aglycines         RGFILEHFTYLRDAWNWLDFIVIALAYVTMGIELGNLAVLRTFRVLRALKTVAIVPGLKTIVGAVIESVKNLRDVIILTIFSLSVFALLGLQIYMGVLTQ
QTJ01838.1_X2_Aglycines         RGFILEHFTYLRDAWNWLDFIVIALAYVTMGIELGNLAVLRTFRVLRALKTVAIVPGLKTIVGAVIESVKNLRDVIILTIFSLSVFALLGLQIYMGVLTQ
QTJ01839.1_X3_Aglycines         RGFILEHFTYLRDAWNWLDFIVIALAYVTMGIELGNLAVLRTFRVLRALKTVAIVPGLKTIVGAVIESVKNLRDVIILTIFSLSVFALLGLQIYMGVLTQ
MW759885.1_X4_Aglycines         RGFILEHFTYLRDAWNWLDFIVIALAYVTMGIELGNLAVLRTFRVLRALKTVAIVPGLKTIVGAVIESVKNLRDVIILTIFSLSVFALLGLQIYMGVLTQ
XP_016660066.1_X1_Apisum        RGFILEHFTYLRDAWNWLDFIVIALAYVTMGIELGNLAVLRTFRVLRALKTVAIVPGLKTIVGAVIESVKNLRDVIILTIFSLSVFALLGLQIYMGVLTQ
XP_016660067.1_X2_Apisum        RGFILEHFTYLRDAWNWLDFIVIALAYVTMGIELGNLAVLRTFRVLRALKTVAIVPGLKTIVGAVIESVKNLRDVIILTIFSLSVFALLGLQIYMGVLTQ
XP_029346126.1_X3_Apisum        RGFILEHFTYLRDAWNWLDFIVIALAYVTMGIELGNLAVLRTFRVLRALKTVAIVPGLKTIVGAVIESVKNLRDVIILTIFSLSVFALLGLQIYMGVLTQ
XP_029346127.1_X4_Apisum        RGFILEHFTYLRDAWNWLDFIVIALAYVTMGIELGNLAVLRTFRVLRALKTVAIVPGLKTIVGAVIESVKNLRDVIILTIFSLSVFALLGLQIYMGVLTQ
XP_027840099.1_X1_Agossypii     RGFILEHFTYLRDAWNWLDFIVIALAYVTMGIELGNLAVLRTFRVLRALKTVAIVPGLKTIVGAVIESVKNLRDVIILTIFSLSVFALLGLQIYMGVLTQ
XP_027840100.1_X2_Agossypii     RGFILEHFTYLRDAWNWLDFIVIALAYVTMGIELGNLAVLRTFRVLRALKTVAIVPGLKTIVGAVIESVKNLRDVIILTIFSLSVFALLGLQIYMGVLTQ
XP_027840101.1_X3_Agossypii     RGFILEHFTYLRDAWNWLDFIVIALAYVTMGIELGNLAVLRTFRVLRALKTVAIVPGLKTIVGAVIESVKNLRDVIILTIFSLSVFALLGLQIYMGVLTQ
XP_025206080.1_X1_Msacchari     RGFILEHFTYLRDAWNWLDFIVIALAYVTMGIELGNLAVLRTFRVLRALKTVAIVPGLKTIVGAVIESVKNLRDVIILTIFSLSVFALLGLQIYMGVLTQ
XP_025206081.1_X2_Msacchari     RGFILEHFTYLRDAWNWLDFIVIALAYVTMGIELGNLAVLRTFRVLRALKTVAIVPGLKTIVGAVIESVKNLRDVIILTIFSLSVFALLGLQIYMGVLTQ
XP_025206082.1_X3_Msacchari     RGFILEHFTYLRDAWNWLDFIVIALAYVTMGIELGNLAVLRTFRVLRALKTVAIVPGLKTIVGAVIESVKNLRDVIILTIFSLSVFALLGLQIYMGVLTQ
XP_025206083.1_X4_Msacchari     RGFILEHFTYLRDAWNWLDFIVIALAYVTMGIELGNLAVLRTFRVLRALKTVAIVPGLKTIVGAVIESVKNLRDVIILTIFSLSVFALLGLQIYMGVLTQ
XP_026806608.1_X1_Rmaidis       RGFILEHFTYLRDAWNWLDFIVIALAYVTMGIELGNLAVLRTFRVLRALKTVAIVPGLKTIVGAVIESVKNLRDVIILTIFSLSVFALLGLQIYMGVLTQ
XP_026806610.1_X2_Rmaidis       RGFILEHFTYLRDAWNWLDFIVIALAYVTMGIELGNLAVLRTFRVLRALKTVAIVPGLKTIVGAVIESVKNLRDVIILTIFSLSVFALLGLQIYMGVLTQ
XP_026806611.1_X3_Rmaidis       RGFILEHFTYLRDAWNWLDFIVIALAYVTMGIELGNLAVLRTFRVLRALKTVAIVPGLKTIVGAVIESVKNLRDVIILTIFSLSVFALLGLQIYMGVLTQ
XP_026806612.1_X4_Rmaidis       RGFILEHFTYLRDAWNWLDFIVIALAYVTMGIELGNLAVLRTFRVLRALKTVAIVPGLKTIVGAVIESVKNLRDVIILTIFSLSVFALLGLQIYMGVLTQ
XP_022174236.1_X1_Mpersicae     RGFILEHFTYLRDAWNWLDFIVIALAYVTMGIELGNLAVLRTFRVLRALKTVAIVPGLKTIVGAVIESVKNLRDVIILTIFSLSVFALLGLQIYMGVLTQ
XP_022174237.1_X2_Mpersicae     RGFILEHFTYLRDAWNWLDFIVIALAYVTMGIELGNLAVLRTFRVLRALKTVAIVPGLKTIVGAVIESVKNLRDVIILTIFSLSVFALLGLQIYMGVLTQ
XP_022174238.1_X3_Mpersicae     RGFILEHFTYLRDAWNWLDFIVIALAYVTMGIELGNLAVLRTFRVLRALKTVAIVPGLKTIVGAVIESVKNLRDVIILTIFSLSVFALLGLQIYMGVLTQ
XP_022174239.1_X4_Mpersicae     RGFILEHFTYLRDAWNWLDFIVIALAYVTMGIELGNLAVLRTFRVLRALKTVAIVPGLKTIVGAVIESVKNLRDVIILTIFSLSVFALLGLQIYMGVLTQ
XP_015366149.1_X1_Dnoxia        RGFILEHFTYLRDAWNWLDFIVIALAYVTMGIELGNLAVLRTFRVLRALKTVAIVPGLKTIVGAVIESVKNLRDVIILTIFSLSVFALLGLQIYMGVLTQ
XP_015366150.1_X2_Dnoxia        RGFILEHFTYLRDAWNWLDFIVIALAYVTMGIELGNLAVLRTFRVLRALKTVAIVPGLKTIVGAVIESVKNLRDVIILTIFSLSVFALLGLQIYMGVLTQ
XP_015366151.1_X3_Dnoxia        RGFILEHFTYLRDAWNWLDFIVIALAYVTMGIELGNLAVLRTFRVLRALKTVAIVPGLKTIVGAVIESVKNLRDVIILTIFSLSVFALLGLQIYMGVLTQ
XP_015366152.1_X4_Dnoxia        -------------------------DYVTMGIELGNLAVLRTFRVLRALKTVAIVPGLKTIVGAVIESVKNLRDVIILTIFSLSVFALLGLQIYMGVLTQ
XP_025407763.1_X1_Sflava        RGFILEHFTYLRDAWNWLDFIVIALAYVTMGIELGNLAVLRTFRVLRALKTVAIVPGLKTIVGAVIESVKNLRDVIILTIFSLSVFALLGLQIYMGVLTQ
XP_025407764.1_X2_Sflava        RGFILEHFTYLRDAWNWLDFIVIALAYVTMGIELGNLAVLRTFRVLRALKTVAIVPGLKTIVGAVIESVKNLRDVIILTIFSLSVFALLGLQIYMGVLTQ
XP_025407765.1_X3_Sflava        RGFILEHFTYLRDAWNWLDFIVIALAYVTMGIELGNLAVLRTFRVLRALKTVAIVPGLKTIVGAVIESVKNLRDVIILTIFSLSVFALLGLQIYMGVLTQ
XP_025407766.1_X4_Sflava        RGFILEHFTYLRDAWNWLDFIVIALAYVTMGIELGNLAVLRTFRVLRALKTVAIVPGLKTIVGAVIESVKNLRDVIILTIFSLSVFALLGLQIYMGVLTQ
XP_025407767.1_X5_Sflava        RGFILEHFTYLRDAWNWLDFIVIALAYVTMGIELGNLAVLRTFRVLRALKTVAIVPGLKTIVGAVIESVKNLRDVIILTIFSLSVFALLGLQIYMGVLTQ
XP_025407768.1_X6_Sflava        -------------------------DYVTMGIELGNLAVLRTFRVLRALKTVAIVPGLKTIVGAVIESVKNLRDVIILTIFSLSVFALLGLQIYMGVLTQ
```

```
                             301         .         .         .         .         :         .         .         .         .        4 400
AG6007485-PA_Aglycines       KCIKYFPLDGSAGNLTNENWFAFMSNKSNWQPNDDEPDEYRLCGNGTGAGQCDEGYMCIQGFGGNPNYGYTSFDTFAWALLSAFRLMTQDNWEALYQQVL
QTJ01840.1_X1_Aglycines      KCIKYFPLDGSAGNLTNENWFAFMSNKSNWQPNDDEPDEYRLCGNGTGAGQCDEGYMCIQGFGGNPNYGYTSFDTFAWALLSAFRLMTQDNWEALYQQVL
QTJ01838.1_X2_Aglycines      KCIKYFPLDGSAGNLTNENWFAFMSNKSNWQPNDDEPDEYRLCGNGTGAGQCDEGYMCIQGFGGNPNYGYTSFDTFAWALLSAFRLMTQDNWEALYQQVL
QTJ01839.1_X3_Aglycines      KCIKYFPLDGSAGNLTNENWFAFMSNKSNWQPNDDEPDEYRLCGNGTGAGQCDEGYMCIQGFGGNPNYGYTSFDTFAWALLSAFRLMTQDNWEALYQQVL
MW759885.1_X4_Aglycines      KCIKYFPLDGSAGNLTNENWFAFMSNKSNWQPNDDEPDEYRLCGNGTGAGQCDEGYMCIQGFGGNPNYGYTSFDTFAWALLSAFRLMTQDNWEALYQQ--
XP_016660066.1_X1_Apisum     KCIKYFPTDGSAGNLTNENWVAFMSNKSNWQPGKEEPEDYPLCGNGTGAGQCDEGYMCIQGFGMNPNYGYTSFDTFAWALLSAFRLMTQDNWEALYQQVL
XP_016660067.1_X2_Apisum     KCIKYFPTDGSAGNLTNENWVAFMSNKSNWQPGKEEPEDYPLCGNGTGAGQCDEGYMCIQGFGMNPNYGYTSFDTFAWALLSAFRLMTQDNWEALYQQVL
XP_029346126.1_X3_Apisum     KCIKYFPTDGSAGNLTNENWVAFMSNKSNWQPGKEEPEDYPLCGNGTGAGQCDEGYMCIQGFGMNPNYGYTSFDTFAWALLSAFRLMTQDNWEALYQQVL
XP_029346127.1_X4_Apisum     KCIKYFPTDGSAGNLTNENWVAFMSNKSNWQPGKEEPEDYPLCGNGTGAGQCDEGYMCIQGFGMNPNYGYTSFDTFAWALLSAFRLMTQDNWEALYQQVL
XP_027840099.1_X1_Agossypii  KCIKYFPLDGSAGNLTNENWFAFMSNKSNWQPNEDEPDEYRLCGNGTGAGQCDEGYMCIQGFGGNPNYGYTSFDTFAWALLSAFRLMTQDNWEALYQQVL
XP_027840100.1_X2_Agossypii  KCIKYFPLDGSAGNLTNENWFAFMSNKSNWQPNEDEPDEYRLCGNGTGAGQCDEGYMCIQGFGGNPNYGYTSFDTFAWALLSAFRLMTQDNWEALYQQVL
XP_027840101.1_X3_Agossypii  KCIKYFPLDGSAGNLTNENWFAFMSNKSNWQPNEDEPDEYRLCGNGTGAGQCDEGYMCIQGFGGNPNYGYTSFDTFAWALLSAFRLMTQDNWEALYQQVL
XP_025206080.1_X1_Msacchari  KCIKHFPDDGSAGNLTNENWFAFMSNKSNWQPGEDEPDEYPLCGNGTGAGQCKEGYMCIQGFGSNPNYGYTSFDTFAWALLSAFRLMTQDNWEALYQQVL
XP_025206081.1_X2_Msacchari  KCIKHFPDDGSAGNLTNENWFAFMSNKSNWQPGEDEPDEYPLCGNGTGAGQCKEGYMCIQGFGSNPNYGYTSFDTFAWALLSAFRLMTQDNWEALYQQVL
XP_025206082.1_X3_Msacchari  KCIKHFPDDGSAGNLTNENWFAFMSNKSNWQPGEDEPDEYPLCGNGTGAGQCKEGYMCIQGFGSNPNYGYTSFDTFAWALLSAFRLMTQDNWEALYQQVL
XP_025206083.1_X4_Msacchari  KCIKHFPDDGSAGNLTNENWFAFMSNKSNWQPGEDEPDEYPLCGNGTGAGQCKEGYMCIQGFGSNPNYGYTSFDTFAWALLSAFRLMTQDNWEALYQQVL
XP_026806608.1_X1_Rmaidis    KCIKYFPTDGSAGNLTNENWFAFMSNSSNWQPGEEEPDEYPLCGNGTGAGQCKEGYMCIQGFGINPNYGYTSFDTFAWALLSAFRLMTQDNWEALYQQVL
XP_026806610.1_X2_Rmaidis    KCIKYFPTDGSAGNLTNENWFAFMSNSSNWQPGEEEPDEYPLCGNGTGAGQCKEGYMCIQGFGINPNYGYTSFDTFAWALLSAFRLMTQDNWEALYQQVL
XP_026806611.1_X3_Rmaidis    KCIKYFPTDGSAGNLTNENWFAFMSNSSNWQPGEEEPDEYPLCGNGTGAGQCKEGYMCIQGFGINPNYGYTSFDTFAWALLSAFRLMTQDNWEALYQQVL
XP_026806612.1_X4_Rmaidis    KCIKYFPTDGSAGNLTNENWFAFMSNSSNWQPGEEEPDEYPLCGNGTGAGQCKEGYMCIQGFGINPNYGYTSFDTFAWALLSAFRLMTQDNWEALYQQVL
XP_022174236.1_X1_Mpersicae  KCIKYFPLDGSAGNLTNENWVAFMSNKSNWQPGDEEPEDYPLCGNGTGAGQCKEGYMCIQGFGKNPNYGYTSFDTFAWALLSAFRLMTQDNWEALYQQVL
XP_022174237.1_X2_Mpersicae  KCIKYFPLDGSAGNLTNENWVAFMSNKSNWQPGDEEPEDYPLCGNGTGAGQCKEGYMCIQGFGKNPNYGYTSFDTFAWALLSAFRLMTQDNWEALYQQVL
XP_022174238.1_X3_Mpersicae  KCIKYFPLDGSAGNLTNENWVAFMSNKSNWQPGDEEPEDYPLCGNGTGAGQCKEGYMCIQGFGKNPNYGYTSFDTFAWALLSAFRLMTQDNWEALYQQVL
XP_022174239.1_X4_Mpersicae  KCIKYFPLDGSAGNLTNENWVAFMSNKSNWQPGDEEPEDYPLCGNGTGAGQCKEGYMCIQGFGKNPNYGYTSFDTFAWALLSAFRLMTQDNWEALYQQVL
XP_015366149.1_X1_Dnoxia     KCIKHFPTDGSAGNLTNENWFAFMSNSANWQPGEEEPEDYPLCGNGTGAGQCKEGYICIQGFGKNPNYGYTSFDTFAWALLSAFRLMTQDNWEALYQQVL
XP_015366150.1_X2_Dnoxia     KCIKHFPTDGSAGNLTNENWFAFMSNSANWQPGEEEPEDYPLCGNGTGAGQCKEGYICIQGFGKNPNYGYTSFDTFAWALLSAFRLMTQDNWEALYQQVL
XP_015366151.1_X3_Dnoxia     KCIKHFPTDGSAGNLTNENWFAFMSNSANWQPGEEEPEDYPLCGNGTGAGQCKEGYICIQGFGKNPNYGYTSFDTFAWALLSAFRLMTQDNWEALYQQVL
XP_015366152.1_X4_Dnoxia     KCIKHFPTDGSAGNLTNENWFAFMSNSANWQPGEEEPEDYPLCGNGTGAGQCKEGYICIQGFGKNPNYGYTSFDTFAWALLSAFRLMTQDNWEALYQQVL
XP_025407763.1_X1_Sflava     KCIKEFPLDGSAGNLTNENWFAFTSNSSNWQKTE--EEDYTLCGNGTGAGQCQPGYICIQGFGMNPNYGYTSFDTFAWALLSAFRLMTQDNWEALYQQVL
XP_025407764.1_X2_Sflava     KCIKEFPLDGSAGNLTNENWFAFTSNSSNWQKTE--EEDYTLCGNGTGAGQCQPGYICIQGFGMNPNYGYTSFDTFAWALLSAFRLMTQDNWEALYQQVL
XP_025407765.1_X3_Sflava     KCIKEFPLDGSAGNLTNENWFAFTSNSSNWQKTE--EEDYTLCGNGTGAGQCQPGYICIQGFGMNPNYGYTSFDTFAWALLSAFRLMTQDNWEALYQQVL
XP_025407766.1_X4_Sflava     KCIKEFPLDGSAGNLTNENWFAFTSNSSNWQKTE--EEDYTLCGNGTGAGQCQPGYICIQGFGMNPNYGYTSFDTFAWALLSAFRLMTQDNWEALYQQVL
XP_025407767.1_X5_Sflava     KCIKEFPLDGSAGNLTNENWFAFTSNSSNWQKTE--EEDYTLCGNGTGAGQCQPGYICIQGFGMNPNYGYTSFDTFAWALLSAFRLMTQDNWEALYQQVL
XP_025407768.1_X6_Sflava     KCIKEFPLDGSAGNLTNENWFAFTSNSSNWQKTE--EEDYTLCGNGTGAGQCQPGYICIQGFGMNPNYGYTSFDTFAWALLSAFRLMTQDNWEALYQQVL
```

```
                                    ..............DI S6..............
                               401            .         .         .         .         :         .         .         .         .         5 500
AG6007485-PA_Aglycines         RAAGPWHMFFFIVIIFLGSFYLVNLILAIVAMSYDELQKKAEEEEAAEEEAIREAEQAAKDREVRRQAHEERVAERAERARHVTQHPKSPSDFSSQSYDN
QTJ01840.1_X1_Aglycines        RAAGPWHMFFFIVIIFLGSFYLVNLILAIVAMSYDELQKKAEEEEAAEEEAIREAEQAAKDREVRRQAHEERVAERAERARHVTQHPKSPSDFSSQSYDN
QTJ01838.1_X2_Aglycines        RAAGPWHMFFFIVIIFLGSFYLVNLILAIVAMSYDELQKKAEEEEAAEEEAIREAEQAAKDREVRRQAHEERVAERAERARHVTQHPKSPSDFSSQSYDN
QTJ01839.1_X3_Aglycines        RAAGPWHMFFFIVIIFLGSFYLVNLILAIVAMSYDELQKKAEEEEAAEEEAIREAEQAAKDREVRRQAHEERVAERAERARHVTQHPKSPSDFSSQSYDN
MW759885.1_X4_Aglycines        ----------FIVIIFLGSFYLVNLILAIVAMSYDELQKKAEEEEAAEEEAIREAEQAAKDREVRRQAHEERVAERAERARHVTQHPKSPSDFSSQSYDN
XP_016660066.1_X1_Apisum       RAAGPWHMFFFIVIIFLGSFYLVNLILAIVAMSYDELQKKAEEEEAAEEEAIREAEQAAKDREVRRQAHEERVAERAERARHVTQHPKSPSDFSCQSYDN
XP_016660067.1_X2_Apisum       RAAGPWHMFFFIVIIFLGSFYLVNLILAIVAMSYDELQKKAEEEEAAEEEAIREAEQAAKDREVRRQAHEERVAERAERARHVTQHPKSPSDFSCQSYDN
XP_029346126.1_X3_Apisum       RAAGPWHMFFFIVIIFLGSFYLVNLILAIVAMSYDELQKKAEEEEAAEEEAIREAEQAAKDREVRRQAHEERVAERAERARHVTQHPKSPSDFSCQSYDN
XP_029346127.1_X4_Apisum       RAAGPWHMFFFIVIIFLGSFYLVNLILAIVAMSYDELQKKAEEEEAAEEEAIREAEQAAKDREVRRQAHEERVAERAERARHVTQHPKSPSDFSCQSYDN
XP_027840099.1_X1_Agossypii    RAAGPWHMFFFIVIIFLGSFYLVNLILAIVAMSYDELQKKAEEEEAAEEEAIREAEQAAKDREVRRQAHEERVAERAERARHVTQHPKSPSDFSSQSYDN
XP_027840100.1_X2_Agossypii    RAAGPWHMFFFIVIIFLGSFYLVNLILAIVAMSYDELQKKAEEEEAAEEEAIREAEQAAKDREVRRQAHEERVAERAERARHVTQHPKSPSDFSSQSYDN
XP_027840101.1_X3_Agossypii    RAAGPWHMFFFIVIIFLGSFYLVNLILAIVAMSYDELQKKAEEEEAAEEEAIREAEQAAKDREVRRQAHEERVAERAERARHVTQHPKSPSDFSSQSYDN
XP_025206080.1_X1_Msacchari    RAAGPWHMFFFIVIIFLGSFYLVNLILAIVAMSYDELQKKAEEEEAAEEEAIREAEQAAKDREVRRQAHEERVAERAERARHVTQHPKSPSDFSCQSYDN
XP_025206081.1_X2_Msacchari    RAAGPWHMFFFIVIIFLGSFYLVNLILAIVAMSYDELQKKAEEEEAAEEEAIREAEQAAKDREVRRQAHEERVAERAERARHVTQHPKSPSDFSCQSYDN
XP_025206082.1_X3_Msacchari    RAAGPWHMFFFIVIIFLGSFYLVNLILAIVAMSYDELQKKAEEEEAAEEEAIREAEQAAKDREVRRQAHEERVAERAERARHVTQHPKSPSDFSCQSYDN
XP_025206083.1_X4_Msacchari    RAAGPWHMFFFIVIIFLGSFYLVNLILAIVAMSYDELQKKAEEEEAAEEEAIREAEQAAKDREVRRQAHEERVAERAERARHVTQHPKSPSDFSCQSYDN
XP_026806608.1_X1_Rmaidis      RAAGPWHMFFFIVIIFLGSFYLVNLILAIVAMSYDELQKKAEEEEAAEEEAIREAEQAAKDREVRRQAHEERVAERAERARHVTQHPKSPSDFSCQSYDN
XP_026806610.1_X2_Rmaidis      RAAGPWHMFFFIVIIFLGSFYLVNLILAIVAMSYDELQKKAEEEEAAEEEAIREAEQAAKDREVRRQAHEERVAERAERARHVTQHPKSPSDFSCQSYDN
XP_026806611.1_X3_Rmaidis      RAAGPWHMFFFIVIIFLGSFYLVNLILAIVAMSYDELQKKAEEEEAAEEEAIREAEQAAKDREVRRQAHEERVAERAERARHVTQHPKSPSDFSCQSYDN
XP_026806612.1_X4_Rmaidis      RAAGPWHMFFFIVIIFLGSFYLVNLILAIVAMSYDELQKKAEEEEAAEEEAIREAEQAAKDREVRRQAHEERVAERAERARHVTQHPKSPSDFSCQSYDN
XP_022174236.1_X1_Mpersicae    RAAGPWHMFFFIVIIFLGSFYLVNLILAIVAMSYDELQKKAEEEEAAEEEAIREAEQAAKDREVRRQAHEERVAERAERARHVTQHPKSPSDFSCQSYDN
XP_022174237.1_X2_Mpersicae    RAAGPWHMFFFIVIIFLGSFYLVNLILAIVAMSYDELQKKAEEEEAAEEEAIREAEQAAKDREVRRQAHEERVAERAERARHVTQHPKSPSDFSCQSYDN
XP_022174238.1_X3_Mpersicae    RAAGPWHMFFFIVIIFLGSFYLVNLILAIVAMSYDELQKKAEEEEAAEEEAIREAEQAAKDREVRRQAHEERVAERAERARHVTQHPKSPSDFSCQSYDN
XP_022174239.1_X4_Mpersicae    RAAGPWHMFFFIVIIFLGSFYLVNLILAIVAMSYDELQKKAEEEEAAEEEAIREAEQAAKDREVRRQAHEERVAERAERARHVTQHPKSPSDFSCQSYDN
XP_015366149.1_X1_Dnoxia       RAAGPWHMFFFIVIIFLGSFYLVNLILAIVAMSYDELQKKAEEEEAAEEEAIREAEQAAKDREVRRQAHEERVAERAERARNVTQHPKSPSDFSCQSYDN
XP_015366150.1_X2_Dnoxia       RAAGPWHMFFFIVIIFLGSFYLVNLILAIVAMSYDELQKKAEEEEAAEEEAIREAEQAAKDREVRRQAHEERVAERAERARNVTQHPKSPSDFSCQSYDN
XP_015366151.1_X3_Dnoxia       RAAGPWHMFFFIVIIFLGSFYLVNLILAIVAMSYDELQKKAEEEEAAEEEAIREAEQAAKDREVRRQAHEERVAERAERARNVTQHPKSPSDFSCQSYDN
XP_015366152.1_X4_Dnoxia       RAAGPWHMFFFIVIIFLGSFYLVNLILAIVAMSYDELQKKAEEEEAAEEEAIREAEQAAKDREVRRQAHEERVAERAERARNVTQHPKSPSDFSCQSYDN
XP_025407763.1_X1_Sflava       RAAGPWHMFFFIVIIFLGSFYLVNLILAIVAMSYDELQKKAEEEEAAEEEAIREAEQAAKDREVRRQAHEERVAERAERARHVTQHPKSPSDFSCQSYDN
XP_025407764.1_X2_Sflava       RAAGPWHMFFFIVIIFLGSFYLVNLILAIVAMSYDELQKKAEEEEAAEEEAIREAEQAAKDREVRRQAHEERVAERAERARHVTQHPKSPSDFSCQSYDN
XP_025407765.1_X3_Sflava       RAAGPWHMFFFIVIIFLGSFYLVNLILAIVAMSYDELQKKAEEEEAAEEEAIREAEQAAKDREVRRQAHEERVAERAERARHVTQHPKSPSDFSCQSYDN
XP_025407766.1_X4_Sflava       RAAGPWHMFFFIVIIFLGSFYLVNLILAIVAMSYDELQKKAEEEEAAEEEAIREAEQAAKDREVRRQAHEERVAERAERARHVTQHPKSPSDFSCQSYDN
XP_025407767.1_X5_Sflava       RAAGPWHMFFFIVIIFLGSFYLVNLILAIVAMSYDELQKKAEEEEAAEEEAIREAEQAAKDREVRRQAHEERVAERAERARHVTQHPKSPSDFSCQSYDN
XP_025407768.1_X6_Sflava       RAAGPWHMFFFIVIIFLGSFYLVNLILAIVAMSYDELQKKAEEEEAAEEEAIREAEQAAKDREVRRQAHEERVAERAERARHVTQHPKSPSDFSCQSYDN
```

```
                               501          .         .         .         .         :         .         .         .         .     6 600
AG6007485-PA_Aglycines       MFGGGQDRGIGNDHHREKNMSLRSMSITSHDKHSDTGSVDRQSGKTRKASLSLPGSPFNIRRASRGSHQLSHRNGRPRFTGADTKPLVLNTLLDAEEHLP
QTJ01840.1_X1_Aglycines      MFGGGQDRGIGNDHHREKNMSLRSMSITSHDKHSDTGSVDRQSGKTRKASLSLPGSPFNIRRASRGSHQLSHRNGRPRFTGADTKPLVLNTLLDAEEHLP
QTJ01838.1_X2_Aglycines      MFGGGQDRGIGNDHHREKNMSLRSMSITSHDKHSDTGSVDRQSGKTRKASLSLPGSPFNIRRASRGSHQLSHRNGRPRFTGADTKPLVLNTLLDAEEHLP
QTJ01839.1_X3_Aglycines      MFGGGQDRGIGNDHHREKNMSLRSMSITSHDKHSDTGSVDRQSGKTRKASLSLPGSPFNIRRASRGSHQLSHRNGRPRFTGADTKPLVLNTLLDAEEHLP
MW759885.1_X4_Aglycines      MFGGGQDRGIGNDHHREKNMSLRSMSITSHDKHSDTGSVDRQSGKTRKASLSLPGSPFNIRRASRGSHQLSHRNGRPRFTGADTKPLVLNTLLDAEEHLP
XP_016660066.1_X1_Apisum     MFTSGQDRGMGNDHHREK-MSLRSVSITSHDKHSDTGSVDRQSGKTRKASLSLPGSPFNIRRASRGSHQLSHRNGRPRFTGADTKPLVLNTLLDAEEHLP
XP_016660067.1_X2_Apisum     MFTSGQDRGMGNDHHREK-MSLRSVSITSHDKHSDTGSVDRQSGKTRKASLSLPGSPFNIRRASRGSHQLSHRNGRPRFTGADTKPLVLNTLLDAEEHLP
XP_029346126.1_X3_Apisum     MFTSGQDRGMGNDHHREK-MSLRSVSITSHDKHSDTGSVDRQSGKTRKASLSLPGSPFNIRRASRGSHQLSHRNGRPRFTGADTKPLVLNTLLDAEEHLP
XP_029346127.1_X4_Apisum     MFTSGQDRGMGNDHHREK-MSLRSVSITSHDKHSDTGSVDRQSGKTRKASLSLPGSPFNIRRASRGSHQLSHRNGRPRFTGADTKPLVLNTLLDAEEHLP
XP_027840099.1_X1_Agossypii MFGGGQDRGIGNDHHREKNMSLRSMSITSHDKHSDTGSVDRQSGKTRKASLSLPGSPFNIRRASRGSHQLSHRNGRPRFTGADTKPLVLNTLLDAEEHLP
XP_027840100.1_X2_Agossypii MFGGGQDRGIGNDHHREKNMSLRSMSITSHDKHSDTGSVDRQSGKTRKASLSLPGSPFNIRRASRGSHQLSHRNGRPRFTGADTKPLVLNTLLDAEEHLP
XP_027840101.1_X3_Agossypii MFGGGQDRGIGNDHHREKNMSLRSMSITSHDKHSDTGSVDRQSGKTRKASLSLPGSPFNIRRASRGSHQLSHRNGRPRFTGADTKPLVLNTLLDAEEHLP
XP_025206080.1_X1_Msacchari MFAGGQDRGIGNDHHREK-MSLRSVSITSHDKHSDTGSVDRQSGKTRKASLSLPGSPFNIRRASRGSHQLSHRNGRPRFTGADTKPLVLNTLLDAEEHLP
XP_025206081.1_X2_Msacchari MFAGGQDRGIGNDHHREK-MSLRSVSITSHDKHSDTGSVDRQSGKTRKASLSLPGSPFNIRRASRGSHQLSHRNGRPRFTGADTKPLVLNTLLDAEEHLP
XP_025206082.1_X3_Msacchari MFAGGQDRGIGNDHHREK-MSLRSVSITSHDKHSDTGSVDRQSGKTRKASLSLPGSPFNIRRASRGSHQLSHRNGRPRFTGADTKPLVLNTLLDAEEHLP
XP_025206083.1_X4_Msacchari MFAGGQDRGIGNDHHREK-MSLRSVSITSHDKHSDTGSVDRQSGKTRKASLSLPGSPFNIRRASRGSHQLSHRNGRPRFTGADTKPLVLNTLLDAEEHLP
XP_026806608.1_X1_Rmaidis    MFADGQDRGIGNDHHREK-MSLRSVSITSHDKHSDTGSVDRQSGKTRKASLSLPGSPFNIRRASRGSHQLSHRNGRPRFTGADTKPLVLNTLLDAEEHLP
XP_026806610.1_X2_Rmaidis    MFADGQDRGIGNDHHREK-MSLRSVSITSHDKHSDTGSVDRQSGKTRKASLSLPGSPFNIRRASRGSHQLSHRNGRPRFTGADTKPLVLNTLLDAEEHLP
XP_026806611.1_X3_Rmaidis    MFADGQDRGIGNDHHREK-MSLRSVSITSHDKHSDTGSVDRQSGKTRKASLSLPGSPFNIRRASRGSHQLSHRNGRPRFTGADTKPLVLNTLLDAEEHLP
XP_026806612.1_X4_Rmaidis    MFADGQDRGIGNDHHREK-MSLRSVSITSHDKHSDTGSVDRQSGKTRKASLSLPGSPFNIRRASRGSHQLSHRNGRPRFTGADTKPLVLNTLLDAEEHLP
XP_022174236.1_X1_Mpersicae MFASGQDRGMGNDHHREK-MSLRSVSITSHDKHSDTGSVDRQSGKTRKASLSLPGSPFNIRRASRGSHQLSHRNGRPRFTGADTKPLVLNTLLDAEEHLP
XP_022174237.1_X2_Mpersicae MFASGQDRGMGNDHHREK-MSLRSVSITSHDKHSDTGSVDRQSGKTRKASLSLPGSPFNIRRASRGSHQLSHRNGRPRFTGADTKPLVLNTLLDAEEHLP
XP_022174238.1_X3_Mpersicae MFASGQDRGMGNDHHREK-MSLRSVSITSHDKHSDTGSVDRQSGKTRKASLSLPGSPFNIRRASRGSHQLSHRNGRPRFTGADTKPLVLNTLLDAEEHLP
XP_022174239.1_X4_Mpersicae MFASGQDRGMGNDHHREK-MSLRSVSITSHDKHSDTGSVDRQSGKTRKASLSLPGSPFNIRRASRGSHQLSHRNGRPRFTGADTKPLVLNTLLDAEEHLP
XP_015366149.1_X1_Dnoxia     MFTSGQDRGIGNDHHREK-MSLRSVSITSHDKHSDTGSVDRQNGKTRKASLSLPGSPFNIRRASRGSHQLSHRNGRPRFTGSDTKPLVLNTLLDAEEHLP
XP_015366150.1_X2_Dnoxia     MFTSGQDRGIGNDHHREK-MSLRSVSITSHDKHSDTGSVDRQNGKTRKASLSLPGSPFNIRRASRGSHQLSHRNGRPRFTGSDTKPLVLNTLLDAEEHLP
XP_015366151.1_X3_Dnoxia     MFTSGQDRGIGNDHHREK-MSLRSVSITSHDKHSDTGSVDRQNGKTRKASLSLPGSPFNIRRASRGSHQLSHRNGRPRFTGSDTKPLVLNTLLDAEEHLP
XP_015366152.1_X4_Dnoxia     MFTSGQDRGIGNDHHREK-MSLRSVSITSHDKHSDTGSVDRQNGKTRKASLSLPGSPFNIRRASRGSHQLSHRNGRPRFTGSDTKPLVLNTLLDAEEHLP
XP_025407763.1_X1_Sflava     MFA-GQDRGIGDDNHREK-MSLRSASMTSHDKHSDTASVDRQNGKTRKASLSLPGSPFNIRRASRGSHQLSHRNGRPRFTGADTKPLVLNTLLDAEEHLP
XP_025407764.1_X2_Sflava     MFA-GQDRGIGDDNHREK-MSLRSASMTSHDKHSDTASVDRQNGKTRKASLSLPGSPFNIRRASRGSHQLSHRNGRPRFTGADTKPLVLNTLLDAEEHLP
XP_025407765.1_X3_Sflava     MFA-GQDRGIGDDNHREK-MSLRSASMTSHDKHSDTASVDRQNGKTRKASLSLPGSPFNIRRASRGSHQLSHRNGRPRFTGADTKPLVLNTLLDAEEHLP
XP_025407766.1_X4_Sflava     MFA-GQDRGIGDDNHREK-MSLRSASMTSHDKHSDTASVDRQNGKTRKASLSLPGSPFNIRRASRGSHQLSHRNGRPRFTGADTKPLVLNTLLDAEEHLP
XP_025407767.1_X5_Sflava     MFA-GQDRGIGDDNHREK-MSLRSASMTSHDKHSDTASVDRQNGKTRKASLSLPGSPFNIRRASRGSHQLSHRNGRPRFTGADTKPLVLNTLLDAEEHLP
XP_025407768.1_X6_Sflava     MFA-GQDRGIGDDNHREK-MSLRSASMTSHDKHSDTASVDRQNGKTRKASLSLPGSPFNIRRASRGSHQLSHRNGRPRFTGADTKPLVLNTLLDAEEHLP
```

```
                           601         .         .         .         .         :         .         .         .         .        7 700
AG6007485_X1_Aglycines       YADDSNAVTPMSEENGAIIVPVSYANFGSRHSSYTSHTSRITYTSHADLFKPPMTKERQLRSRSARNYFNPSDQRYHRDDDYDTSSMSKSKQKVDECGYS
QTJ01840.1_X1_Aglycines      YADDSNAVTPMSEENGAIIVPVSYANFGSRHSSYTSHTSRITYTSHADLFKPPMTKERQLRSRSARNYFNPSDQRYHRDDDYDTSSMSKSKQKVDECGYS
QTJ01838.1_X2_Aglycines      YADDSNAVTPMSEENGAIIVPVSYANFGSRHSSYTSHTSRITYTSHADLFKPPMTKERQLRSRSARNYFNPSDQRYHRDDDYDTSSMSKSKQKVDECGYS
QTJ01839.1_X3_Aglycines      YADDSNAVTPMSEENGAIIVPVSYANFGSRHSSYTSHTSRITYTSHADLFKPPMTKERQLRSRSARNYFNPSDQRYHRDDDYDTSSMSKSKQKVDECGYS
MW759885.1_X4_Aglycines      YADDSNAVTPMSEENGAIIVPVSYANFGSRHSSYTSHTSRITYTSHADLFKPPMTKERQLRSRSARNYFNPSDQRYHRDDDYDTSSMSKSKQKVDECGYS
XP_016660066.1_X1_Apisum     YADDSNAVTPMSEENGAIIVPVSYANFGSRHSSYTSHTSRITYTSHADLFKPPMTKERQLRSRSARNYFNPSDQRYNRDDDYDSSSMSKSKQQVDECGYN
XP_016660067.1_X2_Apisum     YADDSNAVTPMSEENGAIIVPVSYANFGSRHSSYTSHTSRITYTSHADLFKPPMTKERQLRSRSARNYFNPSDQRYNRDDDYDSSSMSKSKQQVDECGYN
XP_029346126.1_X3_Apisum     YADDSNAVTPMSEENGAIIVPVSYANFGSRHSSYTSHTSRITYTSHADLFKPPMTKERQLRSRSARNYFNPSDQRYNRDDDYDSSSMSKSKQQVDECGYN
XP_029346127.1_X4_Apisum     YADDSNAVTPMSEENGAIIVPVSYANFGSRHSSYTSHTSRITYTSHADLFKPPMTKERQLRSRSARNYFNPSDQRYNRDDDYDSSSMSKSKQQVDECGYN
XP_027840099.1_X1_Agossypii  YADDSNAVTPMSEENGAIIVPVSYANFGSRHSSYTSHTSRITYTSHADLFKPPMTKERQLRSRSARNYFNPSDQRYHRDDDYDTSSMSKSKQKVDECGYS
XP_027840100.1_X2_Agossypii  YADDSNAVTPMSEENGAIIVPVSYANFGSRHSSYTSHTSRITYTSHADLFKPPMTKERQLRSRSARNYFNPSDQRYHRDDDYDTSSMSKSKQKVDECGYS
XP_027840101.1_X3_Agossypii  YADDSNAVTPMSEENGAIIVPVSYANFGSRHSSYTSHTSRITYTSHADLFKPPMTKERQLRSRSARNYFNPSDQRYHRDDDYDTSSMSKSKQKVDECGYS
XP_025206080.1_X1_Msacchari  YADDSNAVTPMSEENGAIIVPVSYANFGSRHSSYTSHTSRITYTSHADLFKPPMTKERQLRSRSARNYFNSSDQRYHRDDDYDTSSMSKSKQKVDECSYN
XP_025206081.1_X2_Msacchari  YADDSNAVTPMSEENGAIIVPVSYANFGSRHSSYTSHTSRITYTSHADLFKPPMTKERQLRSRSARNYFNSSDQRYHRDDDYDTSSMSKSKQKVDECSYN
XP_025206082.1_X3_Msacchari  YADDSNAVTPMSEENGAIIVPVSYANFGSRHSSYTSHTSRITYTSHADLFKPPMTKERQLRSRSARNYFNSSDQRYHRDDDYDTSSMSKSKQKVDECSYN
XP_025206083.1_X4_Msacchari  YADDSNAVTPMSEENGAIIVPVSYANFGSRHSSYTSHTSRITYTSHADLFKPPMTKERQLRSRSARNYFNSSDQRYHRDDDYDTSSMSKSKQKVDECSYN
XP_026806608.1_X1_Rmaidis    YADDSNAVTPMSEENGAIIVPVSYANFGSRHSSYTSHTSRITYTSHADLFKPPMTKERQLRSRSARNYFNPSDQRYHRDDDYDSSSMSKSKQKVDECGYN
XP_026806610.1_X2_Rmaidis    YADDSNAVTPMSEENGAIIVPVSYANFGSRHSSYTSHTSRITYTSHADLFKPPMTKERQLRSRSARNYFNPSDQRYHRDDDYDSSSMSKSKQKVDECGYN
XP_026806611.1_X3_Rmaidis    YADDSNAVTPMSEENGAIIVPVSYANFGSRHSSYTSHTSRITYTSHADLFKPPMTKERQLRSRSARNYFNPSDQRYHRDDDYDSSSMSKSKQKVDECGYN
XP_026806612.1_X4_Rmaidis    YADDSNAVTPMSEENGAIIVPVSYANFGSRHSSYTSHTSRITYTSHADLFKPPMTKERQLRSRSARNYFNPSDQRYHRDDDYDSSSMSKSKQKVDECGYN
XP_022174236.1_X1_Mpersicae  YADDSNAVTPMSEENGAIIVPVSYANFGSRHSSYTSHTSRITYTSHADLFKPPMTKERQLRSRSARNYFNPSDQRYNRDDDYDSSSMSKSKQQVDECGYN
XP_022174237.1_X2_Mpersicae  YADDSNAVTPMSEENGAIIVPVSYANFGSRHSSYTSHTSRITYTSHADLFKPPMTKERQLRSRSARNYFNPSDQRYNRDDDYDSSSMSKSKQQVDECGYN
XP_022174238.1_X3_Mpersicae  YADDSNAVTPMSEENGAIIVPVSYANFGSRHSSYTSHTSRITYTSHADLFKPPMTKERQLRSRSARNYFNPSDQRYNRDDDYDSSSMSKSKQQVDECGYN
XP_022174239.1_X4_Mpersicae  YADDSNAVTPMSEENGAIIVPVSYANFGSRHSSYTSHTSRITYTSHADLFKPPMTKERQLRSRSARNYFNPSDQRYNRDDDYDSSSMSKSKQQVDECGYN
XP_015366149.1_X1_Dnoxia     YADDSNAVTPMSEENGAIIVPVSYANFGSRHSSYTSHTSRITYTSHADLFKPPMTKERQLRSRSARNYFNPSDQRYNRDEDYDSSSMSKSKQQVDECGYN
XP_015366150.1_X2_Dnoxia     YADDSNAVTPMSEENGAIIVPVSYANFGSRHSSYTSHTSRITYTSHADLFKPPMTKERQLRSRSARNYFNPSDQRYNRDEDYDSSSMSKSKQQVDECGYN
XP_015366151.1_X3_Dnoxia     YADDSNAVTPMSEENGAIIVPVSYANFGSRHSSYTSHTSRITYTSHADLFKPPMTKERQLRSRSARNYFNPSDQRYNRDEDYDSSSMSKSKQQVDECGYN
XP_015366152.1_X4_Dnoxia     YADDSNAVTPMSEENGAIIVPVSYANFGSRHSSYTSHTSRITYTSHADLFKPPMTKERQLRSRSARNYFNPSDQRYNRDEDYDSSSMSKSKQQVDECGYN
XP_025407763.1_X1_Sflava     YADDSNAVTPMSEENGAIIVPVSYANFGSRHSSYTSHTSRITYTSHADLFKPPMTKERQLRSRSARNYYNPSEQRYYQDDDFDSSSMSKSKQQVDECGYN
XP_025407764.1_X2_Sflava     YADDSNAVTPMSEENGAIIVPVSYANFGSRHSSYTSHTSRITYTSHADLFKPPMTKERQLRSRSARNYYNPSEQRYYQDDDFDSSSMSKSKQQVDECGYN
XP_025407765.1_X3_Sflava     YADDSNAVTPMSEENGAIIVPVSYANFGSRHSSYTSHTSRITYTSHADLFKPPMTKERQLRSRSARNYYNPSEQRYYQDDDFDSSSMSKSKQQVDECGYN
XP_025407766.1_X4_Sflava     YADDSNAVTPMSEENGAIIVPVSYANFGSRHSSYTSHTSRITYTSHADLFKPPMTKERQLRSRSARNYYNPSEQRYYQDDDFDSSSMSKSKQQVDECGYN
XP_025407767.1_X5_Sflava     YADDSNAVTPMSEENGAIIVPVSYANFGSRHSSYTSHTSRITYTSHADLFKPPMTKERQLRSRSARNYYNPSEQRYYQDDDFDSSSMSKSKQQVDECGYN
XP_025407768.1_X6_Sflava     YADDSNAVTPMSEENGAIIVPVSYANFGSRHSSYTSHTSRITYTSHADLFKPPMTKERQLRSRSARNYYNPSEQRYYQDDDFDSSSMSKSKQQVDECGYN
```

```
                        701        .         .         .         .         :         .         .         .         .       8 800
AG6007485-PA_Aglycines      DSQKHTVVDMRDVMVLNDIIEQAAGRQSRGSEKAVITGGTLVGAWGTVSTVYVFPTDEDAVDGEDEEEENEEPTFREKFQVWLLKFIDTFCVWDCGWPWL
QTJ01840.1_X1_Aglycines     DSQKHTVVDMRDVMVLNDIIEQAAGRQSRGSEKAVITGGTLVGAWGTVSTVYVFPTDEDAVDGEDEEEENEEPTFREKFQVWLLKFIDTFCVWDCGWPWL
QTJ01838.1_X2_Aglycines     DSQKHTVVDMRDVMVLNDIIEQAAGRQSRGSEKAVITGGTLVGAWGTVSTVYVFPTDEDAVDGEDEEEENEEPTFREKFQVWLLKFIDTFCVWDCGWPWL
QTJ01839.1_X3_Aglycines     DSQKHTVVDMRDVMVLNDIIEQAAGRQSRGSEKAVITGGTLVGAWGTVSTVYVFPTDEDAVDGEDEEEENEEPTFREKFQVWLLKFIDTFCVWDCGWPWL
MW759885.1_X4_Aglycines     DSQKHTVVDMRDVMVLNDIIEQAAGRQSRGSEKA-------------VSTVYVFPTDEDAVDGEDEEEENEEPTFREKFQVWLLKFIDTFCVWDCGWPWL
XP_016660066.1_X1_Apisum    DSQKHTVVDMRDVMVLNDIIEQAAGRQSRGSEKA-------------VSTVYVFPTDEDAVDGEDEEEDEEPTFREKFQVWLLKFIDTFCVWDCGWPWL
XP_016660067.1_X2_Apisum    DSQKHTVVDMRDVMVLNDIIEQAAGRQSRGSEKA----------------------DEDAVDGEDEEEDEEPTFREKFQVWLLKFIDTFCVWDCGWPWL
XP_029346126.1_X3_Apisum    DSQKHTVVDMRDVMVLNDIIEQAAGRQSRGSEKA-------------VSTVYVFPTDEDAVDGEDEEEDEEPTFREKFQVWLLKFIDTFCVWDCGWPWL
XP_029346127.1_X4_Apisum    DSQKHTVVDMRDVMVLNDIIEQAAGRQSRGSEKA----------------------DEDAVDGEDEEEDEEPTFREKFQVWLLKFIDTFCVWDCGWPWL
XP_027840099.1_X1_Agossypii DSQKHTVVDMRDVMVLNDIIEQAAGRQSRGSEKA-------------VSTVYVFPTDEDAVDGEDEEEENEEPTFREKFQVWLLKFIDTFCVWDCGWPWL
XP_027840100.1_X2_Agossypii DSQKHTVVDMRDVMVLNDIIEQAAGRQSRGSEKA-------------VSTVYVFPTDEDAVDGEDEEEENEEPTFREKFQVWLLKFIDTFCVWDCGWPWL
XP_027840101.1_X3_Agossypii DSQKHTVVDMRDVMVLNDIIEQAAGRQSRGSEKA----------------------DEDAVDGEDEEEENEEPTFREKFQVWLLKFIDTFCVWDCGWPWL
XP_025206080.1_X1_Msacchari DSQKHTVVDMRDVMVLNDIIEQAAGRQSRGSEKA-------------VSTVYVFPTDEDAVDGEDEEEEDEEPTFREKFQVWLLKFIDTFCVWDCGWPWL
XP_025206081.1_X2_Msacchari DSQKHTVVDMRDVMVLNDIIEQAAGRQSRGSEKA----------------------DEDAVDGEDEEEEDEEPTFREKFQVWLLKFIDTFCVWDCGWPWL
XP_025206082.1_X3_Msacchari DSQKHTVVDMRDVMVLNDIIEQAAGRQSRGSEKA-------------VSTVYVFPTDEDAVDGEDEEEEDEEPTFREKFQVWLLKFIDTFCVWDCGWPWL
XP_025206083.1_X4_Msacchari DSQKHTVVDMRDVMVLNDIIEQAAGRQSRGSEKA----------------------DEDAVDGEDEEEEDEEPTFREKFQVWLLKFIDTFCVWDCGWPWL
XP_026806608.1_X1_Rmaidis   DSQKHTVVDMRDVMVLNDIIEQAAGRQSRGSEKA-------------VSTVYVFPTDEDAVDGEDEEEEDEEPTFREKFQVWLLKFIDTFCVWDCGWPWL
XP_026806610.1_X2_Rmaidis   DSQKHTVVDMRDVMVLNDIIEQAAGRQSRGSEKA----------------------DEDAVDGEDEEEEDEEPTFREKFQVWLLKFIDTFCVWDCGWPWL
XP_026806611.1_X3_Rmaidis   DSQKHTVVDMRDVMVLNDIIEQAAGRQSRGSEKA-------------VSTVYVFPTDEDAVDGEDEEEEDEEPTFREKFQVWLLKFIDTFCVWDCGWPWL
XP_026806612.1_X4_Rmaidis   DSQKHTVVDMRDVMVLNDIIEQAAGRQSRGSEKA----------------------DEDAVDGEDEEEEDEEPTFREKFQVWLLKFIDTFCVWDCGWPWL
XP_022174236.1_X1_Mpersicae DSQKHTVVDMRDVMVLNDIIEQAAGRQSRGSEKA-------------VSTVYVFPTDEDAVDGEDEGEEDEEPTFREKFQVWLLKFIDTFCVWDCGWPWL
XP_022174237.1_X2_Mpersicae DSQKHTVVDMRDVMVLNDIIEQAAGRQSRGSEKA-------------VSTVYVFPTDEDAVDGEDEGEEDEEPTFREKFQVWLLKFIDTFCVWDCGWPWL
XP_022174238.1_X3_Mpersicae DSQKHTVVDMRDVMVLNDIIEQAAGRQSRGSEKA-------------VSTVYVFPTDEDAVDGEDEGEEDEEPTFREKFQVWLLKFIDTFCVWDCGWPWL
XP_022174239.1_X4_Mpersicae DSQKHTVVDMRDVMVLNDIIEQAAGRQSRGSEKA----------------------DEDAVDGEDEGEEDEEPTFREKFQVWLLKFIDTFCVWDCGWPWL
XP_015366149.1_X1_Dnoxia    XSQKHTVVDMRDVMVLNDIIEQAAGRQSRGSEKA-------------VSTVYVFPTDEDAVDGEDEEEEEEEPTFREKFQVWLLKFIDTFCVWDCGWPWL
XP_015366150.1_X2_Dnoxia    XSQKHTVVDMRDVMVLNDIIEQAAGRQSRGSEKA-------------VSTVYVFPTDEDAVDGEDEEEEEEEPTFREKFQVWLLKFIDTFCVWDCGWPWL
XP_015366151.1_X3_Dnoxia    XSQKHTVVDMRDVMVLNDIIEQAAGRQSRGSEKA----------------------DEDAVDGEDEEEEEEEPTFREKFQVWLLKFIDTFCVWDCGWPWL
XP_015366152.1_X4_Dnoxia    XSQKHTVVDMRDVMVLNDIIEQAAGRQSRGSEKA-------------VSTVYVFPTDEDAVDGEDEEEEEEEPTFREKFQVWLLKFIDTFCVWDCGWPWL
XP_025407763.1_X1_Sflava    DSQKHTVVDMRDVMVLNDIIEQAAGRHSRGSDKA-------------VSTVYVFPTDEEAIDGEGEDDEDDEPTFREKFQVWLLKFIDTFCVWDCGWPWL
XP_025407764.1_X2_Sflava    DSQKHTVVDMRDVMVLNDIIEQAAGRHSRGSDKA----------------------DEEAIDGEGEDDEDDEPTFREKFQVWLLKFIDTFCVWDCGWPWL
XP_025407765.1_X3_Sflava    DSQKHTVVDMRDVMVLNDIIEQAAGRHSRGSDKA-------------VSTVYVFPTDEEAIDGEGEDDEDDEPTFREKFQVWLLKFIDTFCVWDCGWPWL
XP_025407766.1_X4_Sflava    DSQKHTVVDMRDVMVLNDIIEQAAGRHSRGSDKA-------------VSTVYVFPTDEEAIDGEGEDDEDDEPTFREKFQVWLLKFIDTFCVWDCGWPWL
XP_025407767.1_X5_Sflava    DSQKHTVVDMRDVMVLNDIIEQAAGRHSRGSDKA----------------------DEEAIDGEGEDDEDDEPTFREKFQVWLLKFIDTFCVWDCGWPWL
XP_025407768.1_X6_Sflava    DSQKHTVVDMRDVMVLNDIIEQAAGRHSRGSDKA-------------VSTVYVFPTDEEAIDGEGEDDEDDEPTFREKFQVWLLKFIDTFCVWDCGWPWL
```

```
                                   ..........DiI S1.............          ..........DI S2.............       .......DII S3.........
                         801       .         .         .         .         :         .         .         .         .         9 900
AG6007485-PA_Aglycines     KFQQGLAFIVFDPFVELYITLCIVVNTLFMALDHHEMDPKLDFILNKANVFFSATFGVEAALKLMAMSPKYYFQMGWNIFDFIIVILSVVELLSAGYQGL
QTJ01840.1_X1_Aglycines    KFQQGLAFIVFDPFVELYITLCIVVNTLFMALDHHEMDPKLDFILNKANVFFSATFGVEAALKLMAMSPKYYFQMGWNIFDFIIVILSVVELLSAGYQGL
QTJ01838.1_X2_Aglycines    KFQQGLAFIVFDPFVELYITLCIVVNTLFMALDHHEMDPKLDFILNKANVFFSATFGVEAALKLMAMSPKYYFQMGWNIFDFIIVILSVVELLSAGYQGL
QTJ01839.1_X3_Aglycines    KFQQGLAFIVFDPFVELYITLCIVVNTLFMALDHHEMDPKLDFILNKANVFFSATFGVEAALKLMAMSPKYYFQMGWNIFDFIIVILSVVELLSAGYQGL
MW759885.1_X4_Aglycines    KFQQGLAFIVFDPFVELYITLCIVVNTLFMALDHHEMDPKLDFILNKANVFFSATFGVEAALKLMAMSPKYYFQMGWNIFDFIIVILSVVELLSAGYQGL
XP_016660066.1_X1_Apisum   KFQQGLAFIVFDPFVELYITLCIVVNTLFMALDHHEMDPKLDFVLNKANVFFSATFGVEAALKLMAMSPKYYFQMGWNIFDFIIVILSVVELLSAGYQGL
XP_016660067.1_X2_Apisum   KFQQGLAFIVFDPFVELYITLCIVVNTLFMALDHHEMDPKLDFVLNKANVFFSATFGVEAALKLMAMSPKYYFQMGWNIFDFIIVILSVVELLSAGYQGL
XP_029346126.1_X3_Apisum   KFQQGLAFIVFDPFVELYITLCIVVNTLFMALDHHEMDPKLDFVLNKANVFFSATFGVEAALKLMAMSPKYYFQMGWNIFDFIIVILSVVELLSAGYQGL
XP_029346127.1_X4_Apisum   KFQQGLAFIVFDPFVELYITLCIVVNTLFMALDHHEMDPKLDFVLNKANVFFSATFGVEAALKLMAMSPKYYFQMGWNIFDFIIVILSVVELLSAGYQGL
XP_027840099.1_X1_Agossypii KFQQGLAFIVFDPFVELYITLCIVVNTLFMALDHHEMDPKLDFILNKANVFFSATFGVEAALKLMAMSPKYYFQMGWNIFDFIIVILSVVELLSAGYQGL
XP_027840100.1_X2_Agossypii KFQQGLAFIVFDPFVELYITLCIVVNTLFMALDHHEMDPKLDFILNKANVFFSATFGVEAALKLMAMSPKYYFQMGWNIFDFIIVILSVVELLSAGYQGL
XP_027840101.1_X3_Agossypii KFQQGLAFIVFDPFVELYITLCIVVNTLFMALDHHEMDPKLDFILNKANVFFSATFGVEAALKLMAMSPKYYFQMGWNIFDFIIVILSVVELLSAGYQGL
XP_025206080.1_X1_Msacchari KFQQGLAFIVFDPFVELYITLCIVVNTLFMALDHHEMDPKLDFILNKANVFFSATFGVEAALKLMAMSPKYYFQMGWNIFDFIIVILSVVELLSAGYQGL
XP_025206081.1_X2_Msacchari KFQQGLAFIVFDPFVELYITLCIVVNTLFMALDHHEMDPKLDFILNKANVFFSATFGVEAALKLMAMSPKYYFQMGWNIFDFIIVILSVVELLSAGYQGL
XP_025206082.1_X3_Msacchari KFQQGLAFIVFDPFVELYITLCIVVNTLFMALDHHEMDPKLDFILNKANVFFSATFGVEAALKLMAMSPKYYFQMGWNIFDFIIVILSVVELLSAGYQGL
XP_025206083.1_X4_Msacchari KFQQGLAFIVFDPFVELYITLCIVVNTLFMALDHHEMDPKLDFILNKANVFFSATFGVEAALKLMAMSPKYYFQMGWNIFDFIIVILSVVELLSAGYQGL
XP_026806608.1_X1_Rmaidis  KFQQGLAFIVFDPFVELYITLCIVVNTLFMALDHHEMDPKLDFILNKANVFFSATFGVEAALKLMAMSPKYYFQMGWNIFDFIIVILSVVELLSAGYQGL
XP_026806610.1_X2_Rmaidis  KFQQGLAFIVFDPFVELYITLCIVVNTLFMALDHHEMDPKLDFILNKANVFFSATFGVEAALKLMAMSPKYYFQMGWNIFDFIIVILSVVELLSAGYQGL
XP_026806611.1_X3_Rmaidis  KFQQGLAFIVFDPFVELYITLCIVVNTLFMALDHHEMDPKLDFILNKANVFFSATFGVEAALKLMAMSPKYYFQMGWNIFDFIIVILSVVELLSAGYQGL
XP_026806612.1_X4_Rmaidis  KFQQGLAFIVFDPFVELYITLCIVVNTLFMALDHHEMDPKLDFILNKANVFFSATFGVEAALKLMAMSPKYYFQMGWNIFDFIIVILSVVELLSAGYQGL
XP_022174236.1_X1_Mpersicae KFQQGLAFIVFDPFVELYITLCIVVNTLFMALDHHEMDPKLDFILNKANVFFSATFGVEAALKLMAMSPKYYFQMGWNIFDFIIVILSVVELLSAGYQGL
XP_022174237.1_X2_Mpersicae KFQQGLAFIVFDPFVELYITLCIVVNTLFMALDHHEMDPKLDFILNKANVFFSATFGVEAALKLMAMSPKYYFQMGWNIFDFIIVILSVVELLSAGYQGL
XP_022174238.1_X3_Mpersicae KFQQGLAFIVFDPFVELYITLCIVVNTLFMALDHHEMDPKLDFILNKANVFFSATFGVEAALKLMAMSPKYYFQMGWNIFDFIIVILSVVELLSAGYQGL
XP_022174239.1_X4_Mpersicae KFQQGLAFIVFDPFVELYITLCIVVNTLFMALDHHEMDPKLDFILNKANVFFSATFGVEAALKLMAMSPKYYFQMGWNIFDFIIVILSVVELLSAGYQGL
XP_015366149.1_X1_Dnoxia   KFQQGLAFIVFDPFVELYITLCIVVNTLFMALDHHEMDPKLDFILNKANVFFSATFGVEAALKLMAMSPKYYFQMGWNIFDFIIVILSVVELLSAGYQGL
XP_015366150.1_X2_Dnoxia   KFQQGLAFIVFDPFVELYITLCIVVNTLFMALDHHEMDPKLDFILNKANVFFSATFGVEAALKLMAMSPKYYFQMGWNIFDFIIVILSVVELLSAGYQGL
XP_015366151.1_X3_Dnoxia   KFQQGLAFIVFDPFVELYITLCIVVNTLFMALDHHEMDPKLDFILNKANVFFSATFGVEAALKLMAMSPKYYFQMGWNIFDFIIVILSVVELLSAGYQGL
XP_015366152.1_X4_Dnoxia   KFQQGLAFIVFDPFVELYITLCIVVNTLFMALDHHEMDPKLDFILNKANVFFSATFGVEAALKLMAMSPKYYFQMGWNIFDFIIVILSVVELLSAGYQGL
XP_025407763.1_X1_Sflava   KFQQGLAFIVFDPFVELYITLCIVVNTLFMALDHHEMNPKLDFVLNKANVFFSATFGVEAALKLMAMSPKYYFQMGWNIFDFIIVILSVVELLSAGYQGL
XP_025407764.1_X2_Sflava   KFQQGLAFIVFDPFVELYITLCIVVNTLFMALDHHEMNPKLDFVLNKANVFFSATFGVEAALKLMAMSPKYYFQMGWNIFDFIIVILSVVELLSAGYQGL
XP_025407765.1_X3_Sflava   KFQQGLAFIVFDPFVELYITLCIVVNTLFMALDHHEMNPKLDFVLNKANVFFSATFGVEAALKLMAMSPKYYFQMGWNIFDFIIVILSVVELLSAGYQGL
XP_025407766.1_X4_Sflava   KFQQGLAFIVFDPFVELYITLCIVVNTLFMALDHHEMNPKLDFVLNKANVFFSATFGVEAALKLMAMSPKYYFQMGWNIFDFIIVILSVVELLSAGYQGL
XP_025407767.1_X5_Sflava   KFQQGLAFIVFDPFVELYITLCIVVNTLFMALDHHEMNPKLDFVLNKANVFFSATFGVEAALKLMAMSPKYYFQMGWNIFDFIIVILSVVELLSAGYQGL
XP_025407768.1_X6_Sflava   KFQQGLAFIVFDPFVELYITLCIVVNTLFMALDHHEMNPKLDFVLNKANVFFSATFGVEAALKLMAMSPKYYFQMGWNIFDFIIVILSVVELLSAGYQGL
```

```
                                .........DII S4.........                 ........DII S5...........
                          901          .         .         .         .         :         .         .         .         .         0 1000
AG6007485-PA_Aglycines      SVLRSFRLLRVFKLAKSWPTLNLLISIMGRTIGALGNLTFVLCIIIFIFAVMGMQLFGKNYTEKMYLFKDHELPRWNFTDFLHSFMIVFRVLCGEWIESM
QTJ01840.1_X1_Aglycines     SVLRSFRLLRVFKLAKSWPTLNLLISIMGRTIGALGNLTFVLCIIIFIFAVMGMQLFGKNYTEKMYLFKDHELPRWNFTDFLHSFMIVFRVLCGEWIESM
QTJ01838.1_X2_Aglycines     SVLRSFRLLRVFKLAKSWPTLNLLISIMGRTIGALGNLTFVLCIIIFIFAVMGMQLFGKNYTEKMYLFKDHELPRWNFTDFLHSFMIVFRVLCGEWIESM
QTJ01839.1_X3_Aglycines     SVLRSFRLLRVFKLAKSWPTLNLLISIMGRTIGALGNLTFVLCIIIFIFAVMGMQLFGKNYTEKMYLFKDHELPRWNFTDFLHSFMIVFRVLCGEWIESM
MW759885.1_X4_Aglycines     SVLRSFRLLRVFKLAKSWPTLNLLISIMGRTIGALGNLTFVLCIIIFIFAVMGMQLFGKNYTEKMYLFKDHELPRWNFTDFLHSFMIVFRVLCGEWIESM
XP_016660066.1_X1_Apisum    SVLRSFRLLRVFKLAKSWPTLNLLISIMGRTIGALGNLTFVLCIIIFIFAVMGMQLFGKNYTEKMYLFKDHELPRWNFTDFLHSFMIVFRVLCGEWIESM
XP_016660067.1_X2_Apisum    SVLRSFRLLRVFKLAKSWPTLNLLISIMGRTIGALGNLTFVLCIIIFIFAVMGMQLFGKNYTEKMYLFKDHELPRWNFTDFLHSFMIVFRVLCGEWIESM
XP_029346126.1_X3_Apisum    SVLRSFRLLRVFKLAKSWPTLNLLISIMGRTIGALGNLTFVLCIIIFIFAVMGMQLFGKNYTEKMYLFKDHELPRWNFTDFLHSFMIVFRVLCGEWIESM
XP_029346127.1_X4_Apisum    SVLRSFRLLRVFKLAKSWPTLNLLISIMGRTIGALGNLTFVLCIIIFIFAVMGMQLFGKNYTEKMYLFKDHELPRWNFTDFLHSFMIVFRVLCGEWIESM
XP_027840099.1_X1_Agossypii SVLRSFRLLRVFKLAKSWPTLNLLISIMGRTIGAMGNLTFVLCIIIFIFAVMGMQLFGKNYTEKMYLFKDHELPRWNFTDFLHSFMIVFRVLCGEWIESM
XP_027840100.1_X2_Agossypii SVLRSFRLLRVFKLAKSWPTLNLLISIMGRTIGAMGNLTFVLCIIIFIFAVMGMQLFGKNYTEKMYLFKDHELPRWNFTDFLHSFMIVFRVLCGEWIESM
XP_027840101.1_X3_Agossypii SVLRSFRLLRVFKLAKSWPTLNLLISIMGRTIGAMGNLTFVLCIIIFIFAVMGMQLFGKNYTEKMYLFKDHELPRWNFTDFLHSFMIVFRVLCGEWIESM
XP_025206080.1_X1_Msacchari SVLRSFRLLRVFKLAKSWPTLNLLISIMGRTIGALGNLTFVLCIIIFIFAVMGMQLFGKNYTEKVYLFKDHELPRWNFTDFLHSFMIVFRVLCGEWIESM
XP_025206081.1_X2_Msacchari SVLRSFRLLRVFKLAKSWPTLNLLISIMGRTIGALGNLTFVLCIIIFIFAVMGMQLFGKNYTEKVYLFKDHELPRWNFTDFLHSFMIVFRVLCGEWIESM
XP_025206082.1_X3_Msacchari SVLRSFRLLRVFKLAKSWPTLNLLISIMGRTIGALGNLTFVLCIIIFIFAVMGMQLFGKNYTEKVYLFKDHELPRWNFTDFLHSFMIVFRVLCGEWIESM
XP_025206083.1_X4_Msacchari SVLRSFRLLRVFKLAKSWPTLNLLISIMGRTIGALGNLTFVLCIIIFIFAVMGMQLFGKNYTEKVYLFKDHELPRWNFTDFLHSFMIVFRVLCGEWIESM
XP_026806608.1_X1_Rmaidis   SVLRSFRLLRVFKLAKSWPTLNLLISIMGRTIGALGNLTFVLCIIIFIFAVMGMQLFGKNYTEKMYLFKDHELPRWNFTDFLHSFMIVFRVLCGEWIESM
XP_026806610.1_X2_Rmaidis   SVLRSFRLLRVFKLAKSWPTLNLLISIMGRTIGALGNLTFVLCIIIFIFAVMGMQLFGKNYTEKMYLFKDHELPRWNFTDFLHSFMIVFRVLCGEWIESM
XP_026806611.1_X3_Rmaidis   SVLRSFRLLRVFKLAKSWPTLNLLISIMGRTIGALGNLTFVLCIIIFIFAVMGMQLFGKNYTEKMYLFKDHELPRWNFTDFLHSFMIVFRVLCGEWIESM
XP_026806612.1_X4_Rmaidis   SVLRSFRLLRVFKLAKSWPTLNLLISIMGRTIGALGNLTFVLCIIIFIFAVMGMQLFGKNYTEKMYLFKDHELPRWNFTDFLHSFMIVFRVLCGEWIESM
XP_022174236.1_X1_Mpersicae SVLRSFRLLRVFKLAKSWPTLNLLISIKGRTIGALGNLTFVLCIIIFIFAVMGMQLFGKNYTEKMYMFKDHELPRWNFTDFLHSFMIVFRVLCGEWIESM
XP_022174237.1_X2_Mpersicae SVLRSFRLLRVFKLAKSWPTLNLLISIKGRTIGALGNLTFVLCIIIFIFAVMGMQLFGKNYTEKMYMFKDHELPRWNFTDFLHSFMIVFRVLCGEWIESM
XP_022174238.1_X3_Mpersicae SVLRSFRLLRVFKLAKSWPTLNLLISIKGRTIGALGNLTFVLCIIIFIFAVMGMQLFGKNYTEKMYMFKDHELPRWNFTDFLHSFMIVFRVLCGEWIESM
XP_022174239.1_X4_Mpersicae SVLRSFRLLRVFKLAKSWPTLNLLISIKGRTIGALGNLTFVLCIIIFIFAVMGMQLFGKNYTEKMYMFKDHELPRWNFTDFLHSFMIVFRVLCGEWIESM
XP_015366149.1_X1_Dnoxia    SVLRSFRLLRVFKLAKSWPTLNLLISIMGRTIGALGNLTFVLCIIIFIFAVMGMQLFGKNYTEKMYLFKDHELPRWNFTDFLHSFMIVFRVLCGEWIESM
XP_015366150.1_X2_Dnoxia    SVLRSFRLLRVFKLAKSWPTLNLLISIMGRTIGALGNLTFVLCIIIFIFAVMGMQLFGKNYTEKMYLFKDHELPRWNFTDFLHSFMIVFRVLCGEWIESM
XP_015366151.1_X3_Dnoxia    SVLRSFRLLRVFKLAKSWPTLNLLISIMGRTIGALGNLTFVLCIIIFIFAVMGMQLFGKNYTEKMYLFKDHELPRWNFTDFLHSFMIVFRVLCGEWIESM
XP_015366152.1_X4_Dnoxia    SVLRSFRLLRVFKLAKSWPTLNLLISIMGRTIGALGNLTFVLCIIIFIFAVMGMQLFGKNYTEKMYLFKDHELPRWNFTDFLHSFMIVFRVLCGEWIESM
XP_025407763.1_X1_Sflava    SVLRSFRLLRVFKLAKSWPTLNLLISIMGRTIGALGNLTFVLCIIIFIFAVMGMQLFGKSYTEKVHLFKDQELPRWNFTDFLHSFMIVFRVLCGEWIESM
XP_025407764.1_X2_Sflava    SVLRSFRLLRVFKLAKSWPTLNLLISIMGRTIGALGNLTFVLCIIIFIFAVMGMQLFGKSYTEKVHLFKDQELPRWNFTDFLHSFMIVFRVLCGEWIESM
XP_025407765.1_X3_Sflava    SVLRSFRLLRVFKLAKSWPTLNLLISIMGRTIGALGNLTFVLCIIIFIFAVMGMQLFGKSYTEKVHLFKDQELPRWNFTDFLHSFMIVFRVLCGEWIESM
XP_025407766.1_X4_Sflava    SVLRSFRLLRVFKLAKSWPTLNLLISIMGRTIGALGNLTFVLCIIIFIFAVMGMQLFGKSYTEKVHLFKDQELPRWNFTDFLHSFMIVFRVLCGEWIESM
XP_025407767.1_X5_Sflava    SVLRSFRLLRVFKLAKSWPTLNLLISIMGRTIGALGNLTFVLCIIIFIFAVMGMQLFGKSYTEKVHLFKDQELPRWNFTDFLHSFMIVFRVLCGEWIESM
XP_025407768.1_X6_Sflava    SVLRSFRLLRVFKLAKSWPTLNLLISIMGRTIGALGNLTFVLCIIIFIFAVMGMQLFGKSYTEKVHLFKDQELPRWNFTDFLHSFMIVFRVLCGEWIESM
```

```
                                                  ............DII S6.............
                                     1001          .          .          .          .          :          .          .          .          .          1 1100
AG6007485-PA_Aglycines               WDCLHVGEPTCIPFFLATVVIGNLV-VLNLFLALLLSNFGSSNLSVPTADSDTNKITEAFERIGRFNKWVKTHILNFLKVLRLKITNQISVQVS------
QTJ01840.1_X1_Aglycines              WDCLHVGEPTCIPFFLATVVIGNLV-VLNLFLALLLSNFGSSNLSVPTADSDTNKITEAFERIGRFNKWVKTHILNFLKVLRLKITNQISVQVS------
QTJ01838.1_X2_Aglycines              WDCLHVGEPTCIPFFLATVVIGNLVVVLNLFLALLLSNFGSSNLSVPTADSDTNKITEAFERIGRFNKWVKTHILNFLKVLRLKITNQISVQVS------
QTJ01839.1_X3_Aglycines              WDCLHVGEPTCIPFFLATVVIGNLV-VLNLFLALLLSNFGSSNLSVPTADSDTNKITEAFERIGRFNKWVKTHILNFLKVLRLKITNQISVQVS------
MW759885.1_X4_Aglycines              WDCLHVGEPTCIPFFLATVVIGNLV-VLNLFLALLLSNFGSSNLSVPTADSDTNKITEAFERIGRFNKWVKTHILNFLKVLRLKITNQISVQVS------
XP_016660066.1_X1_Apisum             WDCLHVGEPTCIPFFLATVVIGNLV-VLNLFLALLLSNFGSSNLSVPTADSDTNKITEAFERISRFNKWVKTRIMDFLKALRAKLTNQISVQAS------
XP_016660067.1_X2_Apisum             WDCLHVGEPTCIPFFLATVVIGNLV-VLNLFLALLLSNFGSSNLSVPTADSDTNKITEAFERISRFNKWVKTRIMDFLKALRAKLTNQISVQAS------
XP_029346126.1_X3_Apisum             WDCLHVGEPTCIPFFLATVVIGNLV-VLNLFLALLLSNFGSSNLSVPTADSDTNKITEAFERISRFNKWVKTRIMDFLKALRAKLTNQISVQAS------
XP_029346127.1_X4_Apisum             WDCLHVGEPTCIPFFLATVVIGNLV-VLNLFLALLLSNFGSSNLSVPTADSDTNKITEAFERISRFNKWVKTRIMDFLKALRAKLTNQISVQAS------
XP_027840099.1_X1_Agossypii          WDCLHVGEPTCIPFFLATVVIGNIV-VLNLYLALLLSNFGSSNLSVPTADSDTNKITEAFERIGRFNKWVKTHILNFLKALRLKITNQISVQTS------
XP_027840100.1_X2_Agossypii          WDCLHVGEPTCIPFFLATVVIGNIV-VLNLYLALLLSNFGSSNLSVPTADSDTNKITEAFERIGRFNKWVKTHILNFLKALRLKITNQISVQTS------
XP_027840101.1_X3_Agossypii          WDCLHVGEPTCIPFFLATVVIGNIV-VLNLYLALLLSNFGSSNLSVPTADSDTNKITEAFERIGRFNKWVKTHILNFLKALRLKITNQISVQTS------
XP_025206080.1_X1_Msacchari          WDCLHVGEPTCIPFFLATVVIGNLV-VLNLFLALLLSNFGSSNLSVPTADSDTNKITEAFERIGRFNKWVKKHIMDFLKALRAKLTNQISVQVS------
XP_025206081.1_X2_Msacchari          WDCLHVGEPTCIPFFLATVVIGNLV-VLNLFLALLLSNFGSSNLSVPTADSDTNKITEAFERIGRFNKWVKKHIMDFLKALRAKLTNQISVQVS------
XP_025206082.1_X3_Msacchari          WDCLHVGEPTCIPFFLATVVIGNLV-VLNLFLALLLSNFGSSNLSVPTADSDTNKITEAFERIGRFNKWVKKHIMDFLKALRAKLTNQISVQVS------
XP_025206083.1_X4_Msacchari          WDCLHVGEPTCIPFFLATVVIGNLV-VLNLFLALLLSNFGSSNLSVPTADSDTNKITEAFERIGRFNKWVKKHIMDFLKALRAKLTNQISVQVS------
XP_026806608.1_X1_Rmaidis            WDCLHVGEPTCIPFFLATVVIGNLV-VLNLFLALLLSNFGSSNLSVPTADSDTNKITEAFERIGRFNKWMKMHIMNFLKALRAKITNQISVQAS------
XP_026806610.1_X2_Rmaidis            WDCLHVGEPTCIPFFLATVVIGNLV-VLNLFLALLLSNFGSSNLSVPTADSDTNKITEAFERIGRFNKWMKMHIMNFLKALRAKITNQISVQAS------
XP_026806611.1_X3_Rmaidis            WDCLHVGEPTCIPFFLATVVIGNLV-VLNLFLALLLSNFGSSNLSVPTADSDTNKITEAFERIGRFNKWMKMHIMNFLKALRAKITNQISVQAS------
XP_026806612.1_X4_Rmaidis            WDCLHVGEPTCIPFFLATVVIGNLV-VLNLFLALLLSNFGSSNLSVPTADSDTNKITEAFERIGRFNKWMKMHIMNFLKALRAKITNQISVQAS------
XP_022174236.1_X1_Mpersicae          WDCLHVGEPTCIPFFLATVVIGNFV-VLNLFLALLLSNFGSSNLSVPTADSDTNKITEAFERIGRFNKWVKTRIMDFLKAIRAKLTNQISVQAS------
XP_022174237.1_X2_Mpersicae          WDCLHVGEPTCIPFFLATVVIGNFV-VLNLFLALLLSNFGSSNLSVPTADSDTNKITEAFERIGRFNKWVKTRIMDFLKAIRAKLTNQISVQAS------
XP_022174238.1_X3_Mpersicae          WDCLHVGEPTCIPFFLATVVIGNFV-VLNLFLALLLSNFGSSNLSVPTADSDTNKITEAFERIGRFNKWVKTRIMDFLKAIRAKLTNQISVQAS------
XP_022174239.1_X4_Mpersicae          WDCLHVGEPTCIPFFLATVVIGNFV-VLNLFLALLLSNFGSSNLSVPTADSDTNKITEAFERIGRFNKWVKTRIMDFLKAIRAKLTNQISVQAS------
XP_015366149.1_X1_Dnoxia             WDCLHVGEPTCIPFFLATVVIGNLV-VLNLFLALLLSNFGSSNLSVPTADSDTNKITEAFERIGRFNKWVKTRIMDFLKAIRAKLTNQISVQAS------
XP_015366150.1_X2_Dnoxia             WDCLHVGEPTCIPFFLATVVIGNLV-VLNLFLALLLSNFGSSNLSVPTADSDTNKITEAFERIGRFNKWVKTRIMDFLKAIRAKLTNQISVQAS------
XP_015366151.1_X3_Dnoxia             WDCLHVGEPTCIPFFLATVVIGNLV-VLNLFLALLLSNFGSSNLSVPTADSDTNKITEAFERIGRFNKWVKTRIMDFLKAIRAKLTNQISVQAS------
XP_015366152.1_X4_Dnoxia             WDCLHVGEPTCIPFFLATVVIGNLV-VLNLFLALLLSNFGSSNLSVPTADSDTNKITEAFERIGRFNKWVKTRIMDFLKAIRAKLTNQISVQAS------
XP_025407763.1_X1_Sflava             WDCLLVAESTCIPFFLATVVIGNLV-VLNLFLALLLSNFGSSNLSVPTADSDTNKITEAFERIGRFNKWVKTRIMDFLKALRAKLTNQISVQASGEACLNY
XP_025407764.1_X2_Sflava             WDCLLVAESTCIPFFLATVVIGNLV-VLNLFLALLLSNFGSSNLSVPTADSDTNKITEAFERIGRFNKWVKTRIMDFLKALRAKLTNQISVQASGEACLNY
XP_025407765.1_X3_Sflava             WDCLLVAESTCIPFFLATVVIGNLV-VLNLFLALLLSNFGSSNLSVPTADSDTNKITEAFERIGRFNKWVKTRIMDFLKALRAKLTNQISVQAS------
XP_025407766.1_X4_Sflava             WDCLLVAESTCIPFFLATVVIGNLV-VLNLFLALLLSNFGSSNLSVPTADSDTNKITEAFERIGRFNKWVKTRIMDFLKALRAKLTNQISVQAS------
XP_025407767.1_X5_Sflava             WDCLLVAESTCIPFFLATVVIGNLV-VLNLFLALLLSNFGSSNLSVPTADSDTNKITEAFERIGRFNKWVKTRIMDFLKALRAKLTNQISVQAS------
XP_025407768.1_X6_Sflava             WDCLLVAESTCIPFFLATVVIGNLV-VLNLFLALLLSNFGSSNLSVPTADSDTNKITEAFERIGRFNKWVKTRIMDFLKALRAKLTNQISVQAS------
```

```
                          1101        .         .         .         .         :         .         .         .                 ] 1200
AG6007485-PA_Aglycines      -----GRDRDIDLPVDETIVDVIAPFKDTKEPVEMTIGDGMEFTIPGDVKQKIKKNQVGNSIGNHQGNKVGNDYKKESFDLDSLKC--------------
QTJ01840.1_X1_Aglycines     -----GRDRDIDLPVDETIVDVIAPFKDTKEPVEMTIGDGMEFTIPGDVKQKIKKNQVGNSIGNLQGNKVGNDYKKESFDLDSLKC--------------
QTJ01838.1_X2_Aglycines     -----GRDRDIDLPVDETIVDVIAPFKDTKEPVEMTIGDGMEFTIPGDVKQKIKKNQVGNSIGNLQGNKVGNDYKKESFDLDSLKC--------------
QTJ01839.1_X3_Aglycines     -----GRDRDIDLPVDETIVDVIAPFKDTKEPVEMTIGDGMEFTIPGDVKQKIKKNQVGNSIGNLQGNKVGNDYKKESFDLDSLKCTKEQKKTLDSITKK
MW759885.1_X4_Aglycines     -----GRDRDIDLPVDETIVDVIAPFKDTKEPVEMTIGDGMEFTIPGDVKQKIKKNQVGNSIGNLQGNKVGNDYKKESFDLDSLKC--------------
XP_016660066.1_X1_Apisum    -----GRDRDIDLPADDTIVDVIAPFKDTKEPVEMTIGDGMEFTIPGDVKQKIKKNQVGNSIGNHQGNKVGNDHKKESFDLDSLKC--------------
XP_016660067.1_X2_Apisum    -----GRDRDIDLPADDTIVDVIAPFKDTKEPVEMTIGDGMEFTIPGDVKQKIKKNQVGNSIGNHQGNKVGNDHKKESFDLDSLKC--------------
XP_029346126.1_X3_Apisum    -----GRDRDIDLPADDTIVDVIAPFKDTKEPVEMTIGDGMEFTIPGDVKQKIKKNQVGNSIGNHQGNKVGNDHKKESFDLDSLK---------------
XP_029346127.1_X4_Apisum    -----GRDRDIDLPADDTIVDVIAPFKDTKEPVEMTIGDGMEFTIPGDVKQKIKKNQVGNSIGNHQGNKVGNDHKKESFDLDSLK---------------
XP_027840099.1_X1_Agossypii -----GRDRDIDLPVDETIVDVIAPFKDTKEPVEMTIGDGMEFTIPGDVKQKIKKNQVGNSIGNHQGNKVGNDYKKESFDLDSLKC--------------
XP_027840100.1_X2_Agossypii -----GRDRDIDLPVDETIVDVIAPFKDTKEPVEMTIGDGMEFTIPGDVKQKIKKNQVGNSIGNHQGNKVGNDYKKESFDLDSLK---------------
XP_027840101.1_X3_Agossypii -----GRDRDIDLPVDETIVDVIAPFKDTKEPVEMTIGDGMEFTIPGDVKQKIKKNQVGNSIGNHQGNKVGNDYKKESFDLDSLK---------------
XP_025206080.1_X1_Msacchari -----GRGKDIDLPVDETIVDVIAPFKDTKEPVEMTIGDGMEFTIPGDVKQKIKKNQVGNSIGNHQGNKVGNDYKKESFDLDSLKC--------------
XP_025206081.1_X2_Msacchari -----GRGKDIDLPVDETIVDVIAPFKDTKEPVEMTIGDGMEFTIPGDVKQKIKKNQVGNSIGNHQGNKVGNDYKKESFDLDSLKC--------------
XP_025206082.1_X3_Msacchari -----GRGKDIDLPVDETIVDVIAPFKDTKEPVEMTIGDGMEFTIPGDVKQKIKKNQVGNSIGNHQGNKVGNDYKKESFDLDSLK---------------
XP_025206083.1_X4_Msacchari -----GRGKDIDLPVDETIVDVIAPFKDTKEPVEMTIGDGMEFTIPGDVKQKIKKNQVGNSIGNHQGNKVGNDYKKESFDLDSLK---------------
XP_026806608.1_X1_Rmaidis   -----GRDRDIDLPVDETIVDVIAPFKDTKEPVEMTIGDGMEFTIPGDVKQKIKKNQVGNSIGNHQGNKVGNDYKKESFDLDSLKC--------------
XP_026806610.1_X2_Rmaidis   -----GRDRDIDLPVDETIVDVIAPFKDTKEPVEMTIGDGMEFTIPGDVKQKIKKNQVGNSIGNHQGNKVGNDYKKESFDLDSLKC--------------
XP_026806611.1_X3_Rmaidis   -----GRDRDIDLPVDETIVDVIAPFKDTKEPVEMTIGDGMEFTIPGDVKQKIKKNQVGNSIGNHQGNKVGNDYKKESFDLDSLK---------------
XP_026806612.1_X4_Rmaidis   -----GRDRDIDLPVDETIVDVIAPFKDTKEPVEMTIGDGMEFTIPGDVKQKIKKNQVGNSIGNHQGNKVGNDYKKESFDLDSLK---------------
XP_022174236.1_X1_Mpersicae -----GRDRDIDLPVDETIVDVIAPFKDTKEPVEMTIGDGMEFTIPGDVKQKTKKNQVGNSIGNHQGNKVGNDYKKESFDLDSLKC--------------
XP_022174237.1_X2_Mpersicae -----GRDRDIDLPVDETIVDVIAPFKDTKEPVEMTIGDGMEFTIPGDVKQKTKKNQVGNSIGNHQGNKVGNDYKKESFDLDSLKC--------------
XP_022174238.1_X3_Mpersicae -----GRDRDIDLPVDETIVDVIAPFKDTKEPVEMTIGDGMEFTIPGDVKQKTKKNQVGNSIGNHQGNKVGNDYKKESFDLDSLK---------------
XP_022174239.1_X4_Mpersicae -----GRDRDIDLPVDETIVDVIAPFKDTKEPVEMTIGDGMEFTIPGDVKQKTKKNQVGNSIGNHQGNKVGNDYKKESFDLDSLK---------------
XP_015366149.1_X1_Dnoxia    -----GRDRDIDLPVDETIIDVIAPFKDTKEPVEMTIGDGMEFTIPGDVKQKTKRNQVGNSIGNHQGNKVGNDYKKESFDLDNLKC--------------
XP_015366150.1_X2_Dnoxia    -----GRDRDIDLPVDETIIDVIAPFKDTKEPVEMTIGDGMEFTIPGDVKQKTKRNQVGNSIGNHQGNKVGNDYKKESFDLDNLK---------------
XP_015366151.1_X3_Dnoxia    -----GRDRDIDLPVDETIIDVIAPFKDTKEPVEMTIGDGMEFTIPGDVKQKTKRNQVGNSIGNHQGNKVGNDYKKESFDLDNLKC--------------
XP_015366152.1_X4_Dnoxia    -----GRDRDIDLPVDETIIDVIAPFKDTKEPVEMTIGDGMEFTIPGDVKQKTKRNQVGNSIGNHQGNKVGNDYKKESFDLDNLKC--------------
XP_025407763.1_X1_Sflava    YSWDQGRDRDIDLPADEVIVDVITPFKDSKEPVEMTIGDGMEFTIPGDVKQKIKRNQVANSIGNHQGNKVGNDYKKESFDLDSLKC--------------
XP_025407764.1_X2_Sflava    YSWDQGRDRDIDLPADEVIVDVITPFKDSKEPVEMTIGDGMEFTIPGDVKQKIKRNQVANSIGNHQGNKVGNDYKKESFDLDSLKC--------------
XP_025407765.1_X3_Sflava    -----GRDRDIDLPADEVIVDVITPFKDSKEPVEMTIGDGMEFTIPGDVKQKIKRNQVANSIGNHQGNKVGNDYKKESFDLDSLKC--------------
XP_025407766.1_X4_Sflava    -----GRDRDIDLPADEVIVDVITPFKDSKEPVEMTIGDGMEFTIPGDVKQKIKRNQVANSIGNHQGNKVGNDYKKESFDLDSLK---------------
XP_025407767.1_X5_Sflava    -----GRDRDIDLPADEVIVDVITPFKDSKEPVEMTIGDGMEFTIPGDVKQKIKRNQVANSIGNHQGNKVGNDYKKESFDLDSLK---------------
XP_025407768.1_X6_Sflava    -----GRDRDIDLPADEVIVDVITPFKDSKEPVEMTIGDGMEFTIPGDVKQKIKRNQVANSIGNHQGNKVGNDYKKESFDLDSLKC--------------
```

```
                           1201          .      ] 1219
AG6007485-PA_Aglycines      -------------------
QTJ01840.1_X1_Aglycines     -------------------
QTJ01838.1_X2_Aglycines     -------------------
QTJ01839.1_X3_Aglycines     RTKFCNDAFLLENNQLNSS
MW759885.1_X4_Aglycines     -------------------
XP_016660066.1_X1_Apisum    -------------------
XP_016660067.1_X2_Apisum    -------------------
XP_029346126.1_X3_Apisum    -------------------
XP_029346127.1_X4_Apisum    -------------------
XP_027840099.1_X1_Agossypii -------------------
XP_027840100.1_X2_Agossypii -------------------
XP_027840101.1_X3_Agossypii -------------------
XP_025206080.1_X1_Msacchari -------------------
XP_025206081.1_X2_Msacchari -------------------
XP_025206082.1_X3_Msacchari -------------------
XP_025206083.1_X4_Msacchari -------------------
XP_026806608.1_X1_Rmaidis   -------------------
XP_026806610.1_X2_Rmaidis   -------------------
XP_026806611.1_X3_Rmaidis   -------------------
XP_026806612.1_X4_Rmaidis   -------------------
XP_022174236.1_X1_Mpersicae -------------------
XP_022174237.1_X2_Mpersicae -------------------
XP_022174238.1_X3_Mpersicae -------------------
XP_022174239.1_X4_Mpersicae -------------------
XP_015366149.1_X1_Dnoxia    -------------------
XP_015366150.1_X2_Dnoxia    -------------------
XP_015366151.1_X3_Dnoxia    -------------------
XP_015366152.1_X4_Dnoxia    -------------------
XP_025407763.1_X1_Sflava    -------------------
XP_025407764.1_X2_Sflava    -------------------
XP_025407765.1_X3_Sflava    -------------------
XP_025407766.1_X4_Sflava    -------------------
XP_025407767.1_X5_Sflava    -------------------
XP_025407768.1_X6_Sflava    -------------------
```

Figure S4: Prediction of *Aphis glycines* genotypes from Sanger trace data (electropherograms) derived from voltage gated sodium channel (*vgsc*) cDNA amplicon sequencing (GenBank accessions MW759883.1–MW759893.1). **A)** Putative cytosine (C) and thymidine (T) transition mutation causing a predicted leucine (L) to a phenylalanine change at amino acid position 1014 of the *A. glycines* gene mode AG6007485-RA (L1014F), and was orthologous to the L1014F knockdown resistance (*kdr*) mutation in *Musca domestica* (Williamson et al., 1996). Sequenced individuals from susceptible biotype 1 and 3 colonies were homozygous for the wildtype allele encoding a leucine, whereas sequence data from resistant colonies were either homozygous for alleles encoding a phenylalanine or showed co-occurring C and T peaks that were interpreted as representing heterozygotes.

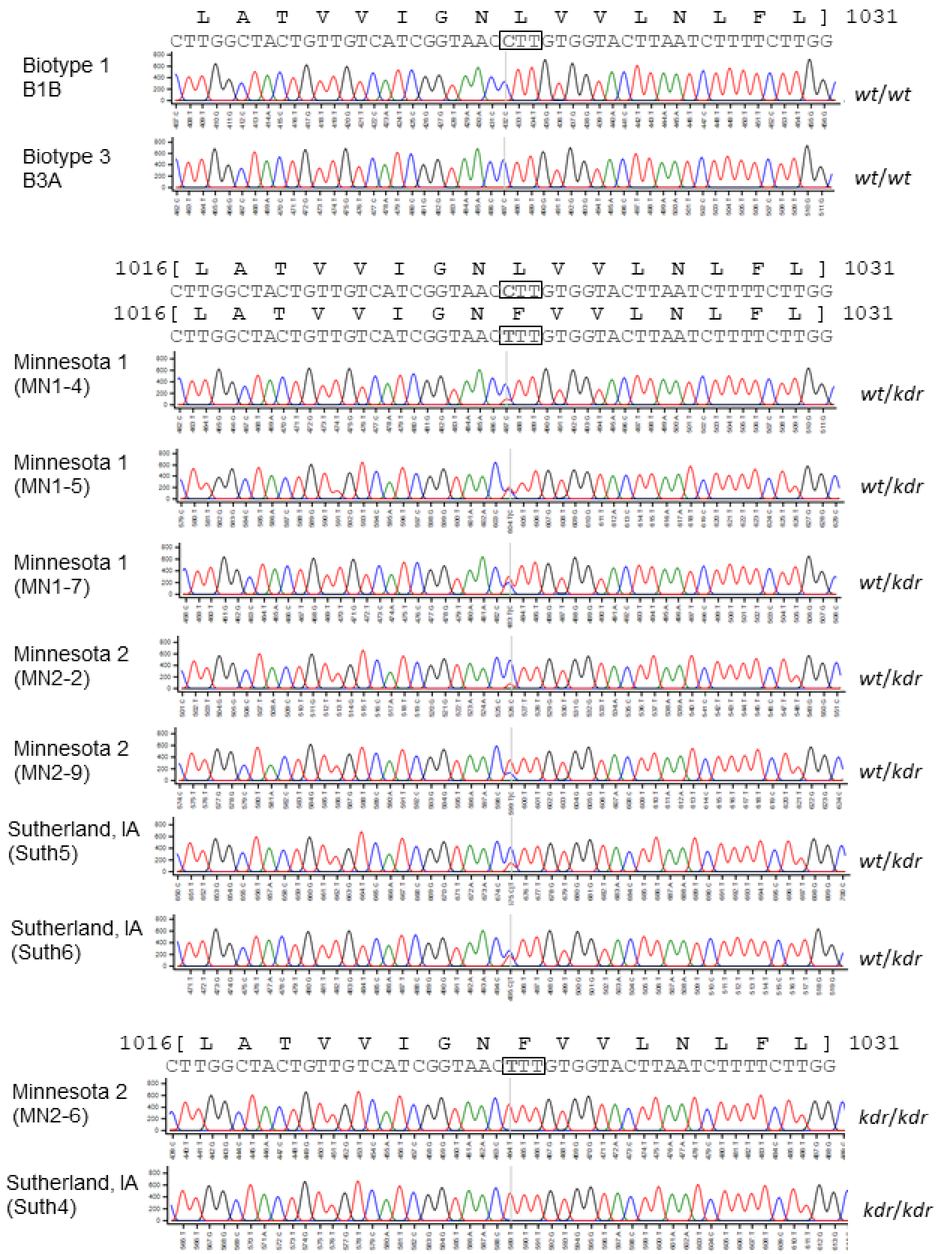

B) Putative guanine (G) to adenosine (A) nucleotide transition mutation causing a predicted methionine (M) to an isoleucine (I) change at amino acid position 918 of the *A. glycines* vgsc protein (M918I), and is orthologous to the position of a M918T mutation in *Musca domestica* (Williamson et al., 1996). Sequenced individuals from susceptible biotype 1 and 3 colonies as well as one sample from resistant colonies MN2 and Sutherland were homozygous for the wildtype allele encoding a M. Sequence data from resistant colonies indicated a single read from a homozygote for the allele encoding an I (single A nucleotide peak), whereas all other reads from resistant colonies had co-occurring G and A peaks that were interpreted as representing heterozygotes.

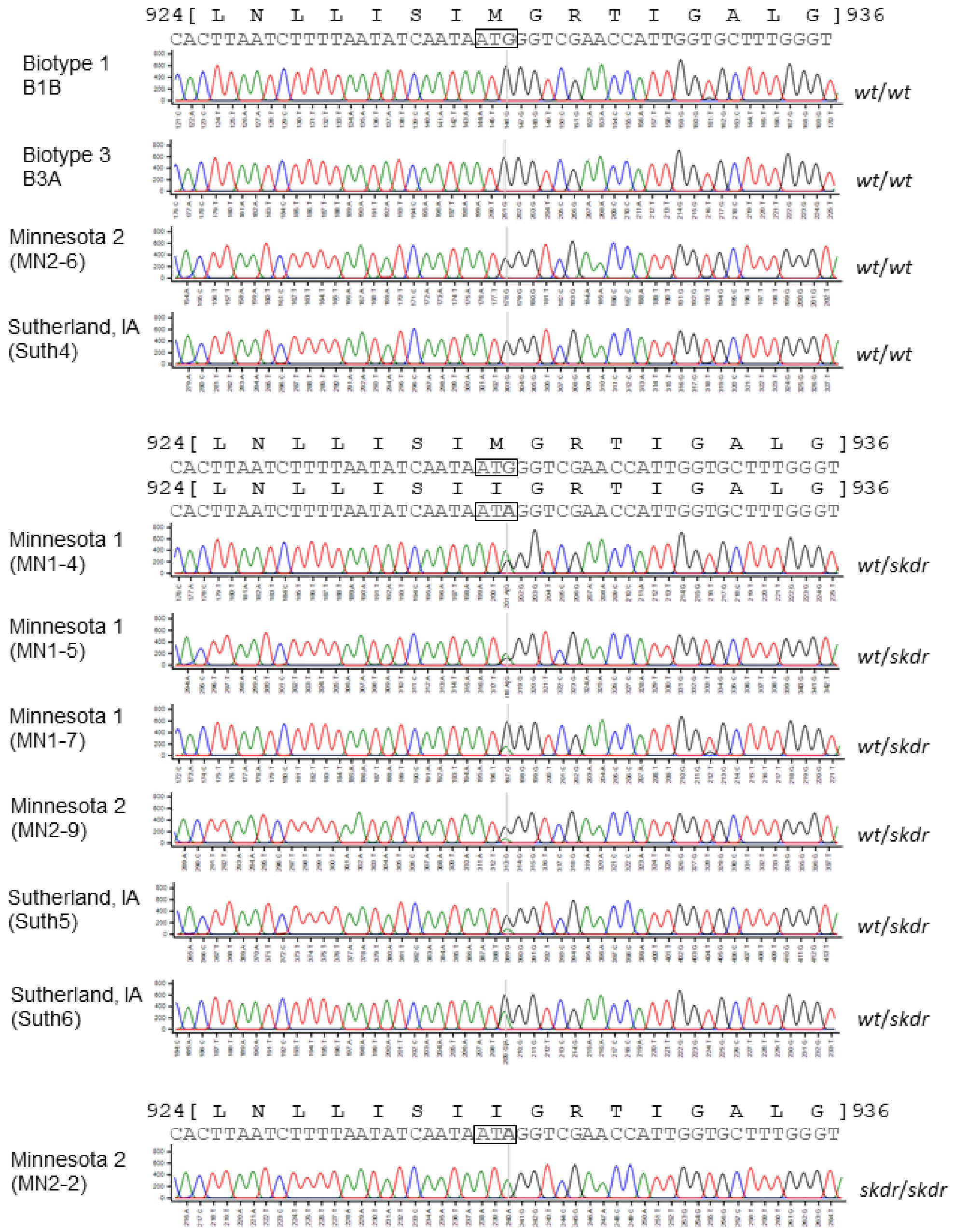

Figure S5: Multiple nucleic acid sequence alignment among *Aphis glycines para*-type voltage gated sodium channel heterodimer 2 (*vgsc-h2*) among isoforms X1, X2, and X3 defined in sequence accession MT379844.1 (Paula et al. 2021), and newly defined isoform X4 from pyrethroid (lambda-cyhalothrin) resistant (RES; GenBank accessions MW759898.1 – MW759904.1) or susceptible (SUS; accessions MW759894.1 – MW759897.1) based on results of leaf-dip bioassay (IRAC, 2019) in this study. Locations of putative splice variation in the cDNA consensus among isoforms are indicated as a plus (+). Partial cDNA sequences gaps corresponding to missing nucleotides are indicated as a dash (-). Coding sequence translations are overlay the nucleotide alignment with structural domain III and IV (D1II and DIV) each comprised of six transmembrane α-helical segments (S1-6) shown as predicted by orthology to the *Musca domestica* VGSC (GenBank accession AAB47604.1; Williamson et al. 1996; **Figure S1**), are overlayed and highlighted in grey. Methionine start (ATG) and stop codons (TAA) are shown accordingly. Predicted point mutations are enclosed in boxes, and those creating putative nonsynonymous (amino acid changing) mutations are indicated with orthologous position in the *M. domestica* VGSC protein indicated (e.g. G1219S, V1424G, and L1430V). Alignment with *M. domestica* VGSC provided in supplementary data (**Figure S2**).

```
                               M  E  H  P  K  I  T  F  G  Q  K  F  I  D  Y  L  G  P  K  N  Y  N  L  M  P  L  N  I  K  K  D  V  F  F  T  D  E  E  L  L   40
                            1 [         .         .         .         .         :         .         .         .         .         1         .         . 120
 1 MT379844.1_X1              ATGGAACATCCTAAAATTACGTTTGGTCAAAAATTCATTGATTACCTAGGTCCAAAAAATTATAATTTAATGCCATTAAATATAAAAAAGGATGTATTCTTTACAGATGAGGAACTCCTG
 2 MT379844.1_X2              ATGGAACATCCTAAAATTACGTTTGGTCAAAAATTCATTGATTACCTAGGTCCAAAAAATTATAATTTAATGCCATTAAATATAAAAAAGGATGTATTCTTTACAGATGAGGAACTCCTG
                                                                                                                  M  D  S  S  S  D  Y  N  V  E  E  L  L
 3 MT379844.1_X3              ---------------------------------------------------------------------------------ATGGACTCCAGTTCGGATTACAATGTAGAGGAACTCCTG
                                                                                                                           M  S  V  Y  S  S  E  E  L  L
 4 MW759895.1_X4_B1B-34       ------------------------------------------------------------------------------------------ATGAGTGTGTATAGTAGTGAGGAACTCCTG SUS
 5 MW759894.1_ND_B1A-34       ------------------------------------------------------------------------------------------------------------------------ SUS
 6 MW759896.1_ND_B3B-34       ------------------------------------------------------------------------------------------------------------------------ SUS
 7 MW759897.1_ND_B4B-34       ------------------------------------------------------------------------------------------------------------------------ SUS
 8 MW759898.1_X4_MN1-4-34     ------------------------------------------------------------------------------------------ATGAGTGTGTATAGTAGTGAGGAACTCCTG RES
 9 MW759899.1_X4_MN1-5-34     ------------------------------------------------------------------------------------------ATGAGTGTGTATAGTAGTGAGGAACTCCTG RES
10 MW759900.1_ND_MN1-7-34     ------------------------------------------------------------------------------------------------------------------------ RES
11 MW759901.1_ND_MN2-2-34     ------------------------------------------------------------------------------------------------------------------------ RES
12 MW759902.1_ND_MN2-9-34     ------------------------------------------------------------------------------------------------------------------------ RES
13 MW759903.1_nd_Suth5-34     ------------------------------------------------------------------------------------------------------------------------ RES
14 MW759904.1_X4_Suth6-34     ------------------------------------------------------------------------------------------ATGAGTGTGTATAGTAGTGAGGAACTCCTG RES


                               D  A  G  I  I  Y  R  N  K  K  E  Q  L  D  V  T  I  G  D  G  M  E  L  L  I  R  G  E  K  N  K  K  K  K  P  P  T  S  S  S   80
                          121           .         .         :         .         .         .         .         2         .         .         .         . 240
 1 MT379844.1_X1              GATGCTGGAATTATTTATCGAAATAAAAAAGAACAACTGGATGTGACTATAGGTGATGGAATGGAGTTACTTATACGTGGAGAAAAAAATAAGAAAAAAAAGCCACCAACGAGCAGTTCG
 2 MT379844.1_X2              GATGCTGGAATTATTTATCGAAATAAAAAAGAACAACTGGATGTGACTATAGGTGATGGAATGGAGTTACTTATACGTGGAGAAAAAAATAAGAAAAAAAAGCCACCAACGAGCAGTTCG
 3 MT379844.1_X3              GATGCTGGAATTATTTATCGAAATAAAAAAGAACAACTGGATGTGACTATAGGTGATGGAATGGAGTTACTTATACGTGGAGAAAAAAATAAGAAAAAAAAGCCACCAACGAGCAGTTCG
 4 MW759894.1_ND_B1A-34       ----------------------------------------------------GTGATGGAATGGAGTTACTTATACGTGGAGAAAAAAATAAGAAAAAAAAGCCACCAACGAGCAGTTCG SUS
 5 MW759895.1_X4_B1B-34       GATGCTGGAATTATTTATCGAAATAAAAAAGAACAACTGGATGGGACTATAGGTGATGGAATGGAGTTACTTATACGTGGAGAAAAAAATAAGAAAAAAAAGCCACCAACGAGCAGTTCG SUS
 6 MW759896.1_ND_B3B-34       -------------------------------------------------TAGGTGATGGAATGGAGTTACTTATACGTGGAGAAAAAAATAAGAAAAAAAAGCCACCAACGAGCAGTTCG SUS
 7 MW759897.1_ND_B4B-34       ---------------------------------------------------------------------------------GAAAAAAATAAGAAAAAAAAGCCACCAACGAGCAGTTCG SUS
 8 MW759898.1_X4_MN1-4-34     GATGCTGGAATTATTTATCGAAATAAAAAAGAACAACTGGATGTGACTATAGGTGATGGAATGGAGTTACTTATACGTGGAGAAAAAAATAAGAAAAAAAAGCCACCAACGAGCAGTTCG RES
 9 MW759899.1_X4_MN1-5-34     GATGCTGGAATTATTTATCGAAATAAAAAAGAACAACTGGATGTGACTATAGGTGATGGAATGGAGTTACTTATACGTGGAGAAAAAAATAAGAAAAAAAAGCCACCAACGAGCAGTTCG RES
10 MW759900.1_ND_MN1-7-34     --------------------------------ACAACTGGATGTGACTATAGGTGATGGAATGGAGTTACTTATACGTGGAGAAAAAAATAAGAAAAAAAAGCCACCAACGAGCAGTTCG RES
11 MW759901.1_ND_MN2-2-34     ---------------------------------------GATGTGACTATAGGTGATGGAATGGAGTTACTTATACGTGGAGAAAAAAATAAGAAAAAAAAGCCACCAACGAGCAGTTCG RES
12 MW759902.1_ND_MN2-9-34     ------------------------------------------------------------------------------GGAGAAAAAAATAAGAAAAAAAAGCCACCAACGAGCAGTTCG RES
13 MW759903.1_nd_Suth5-34     ---------------------------------------GATGTGACTATAAGTGATGGAATGGAGTTACTTATACGTGGAGAAAAAAATAAGAAAAAAAAGCCACCAACGAGCAGTTCG RES
14 MW759904.1_X4_Suth6-34     GATGCTGGAATTATTTATCGAAATAAAAAAGAACAACTGGATGTGACTATAGGTGATGGAATGGAGTTACTTATACGTGGAGAAAAAAATAAGAAAAAAAAGCCACCAACGAGCAGTTCG RES
```

```
                                  Y  N  S  F  G  M  H  Q  S  S  T  D  E  N  Y  Y  L  K  D  K  Y  E  Y  D  T  R  S  T  K  S  Y  G  S  H  E  H  D  P  Y  D  120
                         241              :           .           .           .           .           3           .           .           .           :           .  360
 1 MT379844.1_X1           TATAATTCGTTTGGAATGCACCAAAGCAGTACGGACGAGAACTATTACCTAAAGGATAAATATGAATATGACACTAGAAGTACTAAATCTTACGGAAGTCATGAGCACGATCCTTACGAC
 2 MT379844.1_X2           TATAATTCGTTTGGAATGCACCAAAGCAGTACGGACGAGAACTATTACCTAAAGGATAAATATGAATATGACACTAGAAGTACTAAATCTTACGGAAGTCATGAGCACGATCCTTACGAC
 3 MT379844.1_X3           TATAATTCGTTTGGAATGCACCAAAGCAGTACGGACGAGAACTATTACCTAAAGGATAAATATGAATATGACACTAGAAGTACTAAATCTTACGGAAGTCATGAGCACGATCCTTACGAC
 4 MW759894.1_ND_B1A-34    TATAATTCGTTTGGAATGCACCAAAGCAGTACGGACGAGAACTATTACCTAAAGGATAAATATGAATATGACACTAGAAGTACTAAATCTTACGGAAGTCATGAGCACGATCCTTACGAC SUS
 5 MW759895.1_X4_B1B-34    TATAATTCGTTTGGAATGCACCAAAGCAGTACGGACGAGAACTATTACCTAAAGGATAAATATGAATATGACACTAGAAGTACTAAATCTTACGGAAGTCATGAGCACGATCCTTACGAC SUS
 6 MW759896.1_ND_B3B-34    TATAATTCGTTTGGAATGCACCAAAGCAGTACGGACGAGAACTATTACCTAAAGGATAAATATGAATATGACACTAGAAGTACTAAATCTTACGGAAGTCATGAGCACGATCCTTACGAC SUS
 7 MW759897.1_ND_B4B-34    TATAATTCGTTTGGAATGCACCAAAGCAGTACGGACGAGAACTATTACCTAAAGGATAAATATGAATATGACACTAGAAGTACTAAATCTTACGGAAGTCATGAGCACGATCCTTACGAC SUS
 8 MW759898.1_X4_MN1-4-34  TATAATTCGTTTGGAATGCACCAAAGCAGTACGGACGAGAACTATTACCTAAAGGATAAATATGAATATGACACTAGAAGTACTAAATCTTACGGAAGTCATGAGCACGATCCTTACGAC RES
 9 MW759899.1_X4_MN1-5-34  TATAATTCGTTTGGAATGCACCAAAGCAGTACGGACGAGAACTATTACCTAAAGGATAAATATGAATATGACACTAGAAGTACTAAATCTTACGGAAGTCATGAGCACGATCCTTACGAC RES
10 MW759900.1_ND_MN1-7-34  TATAATTCGTTTGGAATGCACCAAAGCAGTACGGACGAGAACTATTACCTAAAGGATAAATATGAATATGACACTAGAAGTACTAAATCTTACGGAAGTCATGAGCACGATCCTTACGAC RES
11 MW759901.1_ND_MN2-2-34  TATAATTCGTTTGGAATGCACCAAAGCAGTACGGACGAGAACTATTACCTAAAGGATAAATATGAATATGACACTAGAAGTACTAAATCTTACGGAAGTCATGAGCACGATCCTTACGAC RES
12 MW759902.1_ND_MN2-9-34  TATAATTCGTTTGGAATGCACCAAAGCAGTACGGACGAGAACTATTACCTAAAGGATAAATATGAATATGACACTAGAAGTACTAAATCTTACGGAAGTCATGAGCACGATCCTTACGAC RES
13 MW759903.1_nd_Suth5-34  TATAATTCGTTTGGAATGCACCAAAGCAGTACGGACGAGAACTATTACCTAAAGGATAAATATGAATATGACACTAGAAGTACTAAATCTTACGGAAGTCATGAGCACGATCCTTACGAC RES
14 MW759904.1_X4_Suth6-34  TATAATTCGTTTGGAATGCACCAAAGCAGTACGGACGAGAACTATTACCTAAAGGATAAATATGAATATGACACTAGAAGTACTAAATCTTACGGAAGTCATGAGCACGATCCTTACGAC RES

                                  S  E  S  H  R  G  S  K  R  S  L  H  N  A  E  E  K  K  D  P  S  N  E  D  V  E  T  N  Q  N  I  L  G  S  G  H  N  G  I  A  A  160
                         361              .           .           .           4           .           .           .           .           :      G1219S          .  480
 1 MT379844.1_X1           AGTGAGAGCCATAGAGGAAGTAAAAGGAGCTTACATAATGCTGAAGAAAAAAAAGATCCTAGCAATGAAGACGTCGAAACCAATCAAAAC++++++AGTGGTCATAATGGGATTGCTGCA
 2 MT379844.1_X2           AGTGAGAGCCATAGAGGAAGTAAAAGGAGCTTACATAATGCTGAAGAAAAAAAAGATCCTAGCAATGAAGACGTCGAAACCAATCAAAAC++++++AGTGGTCATAATGGGATTGCTGCA
 3 MT379844.1_X3           AGTGAGAGCCATAGAGGAAGTAAAAGGAGCTTACATAATGCTGAAGAAAAAAAAGATCCTAGCAATGAAGACGTCGAAACCAATCAAAAC++++++AGTGGTCATAATGGGATTGCTGCA
 4 MW759894.1_ND_B1A-34    AGTGAGAGCCATAGAGGAAGTAAAAGGAGCTTACATAATGCTGAAGAAAAAAAAGATCCTAGCAATGAAGACGTCGAAACCAATCAAAACATTTTAGGTGGTCATAATGGGATTGCTGCA SUS
 5 MW759895.1_X4_B1B-34    AGTGAGAGCCATAGAGGAAGTAAAAGGAGCTTACATAATGCTGAAGAAAAAAAAGATCCTAGCAATGAAGACGTCGAAACCAATCAAAACATTTTAGGTGGTCATAATGGGATTGCTGCA SUS
 6 MW759896.1_ND_B3B-34    AGTGAGAGCCATAGAGGAAGTAAAAGGAGCTTACATAATGCTGAAGAAAAAAAAGATCCTAGCAATGAAGACGTCGAAACCAATCAAAACATTTTAGGTGGTCATAATGGGATTGCTGCA SUS
 7 MW759897.1_ND_B4B-34    AGTGAGAGCCATAGAGGAAGTAAAAGGAGCTTACATAATGCTGAAGAAAAAAAAGATCCTAGCAATGAAGACGTCGAAACCAATCAAAACATTTTAGGTGGTCATAATGGGATTGCTGCA SUS
 8 MW759898.1_X4_MN1-4-34  AGTGAGAGCCATAGAGGAAGTAAAAGGAGCTTACATAATGCTGAAGAAAAAAAAGATCCTAGCAATGAAGACGTCGAAACCAATCAAAACATTTTAGGTGGTCATAATGGGATTGCTGCA RES
 9 MW759899.1_X4_MN1-5-34  AGTGAGAGCCATAGAGGAAGTAAAAGGAGCTTACATAATGCTGAAGAAAAAAAAGATCCTAGCAATGAAGACGTCGAAACCAATCAAAACATTTTAGGTGGTCATAATGGGATTGCTGCA RES
10 MW759900.1_ND_MN1-7-34  AGTGAGAGCCATAGAGGAAGTAAAAGGAGCTTACATAATGCTGAAGAAAAAAAAGATCCTAGCAATGAAGACGTCGAAACCAATCAAAACATTTTAGGTGGTCATAATGGGATTGCTGCA RES
11 MW759901.1_ND_MN2-2-34  AGTGAGAGCCATAGAGGAAGTAAAAGGAGCTTACATAATGCTGAAGAAAAAAAAGATCCTAGCAATGAAGACGTCGAAACCAATCAAAACATTTTAGGTGGTCATAATGGGATTGCTGCA RES
12 MW759902.1_ND_MN2-9-34  AGTGAGAGCCATAGAGGAAGTAAAAGGAGCTTACATAATGCTGAAGAAAAAAAAGATCCTAGCAATGAAGACGTCGAAACCAATCAAAACATTTTAGGTGGTCATAATGGGATTGCTGCA RES
13 MW759903.1_nd_Suth5-34  AGTGAGAGCCATAGAGGAAGTAAAAGGAGCTTACATAATGCTGAAGAAAAAAAAGATCCTAGCAATGAAGACGTCGAAACCAATCAAAACATTTTAGGTGGTCATAATGGGATTGCTGCA RES
14 MW759904.1_X4_Suth6-34  AGTGAGAGCCATAGAGGAAGTAAAAGGAGCTTACATAATGCTGAAGAAAAAAAAGATCCTAGCAATGAAGACGTCGAAACCAATCAAAACATTTTAGGTGGTCATAATGGGATTGCTGCA RES

                                  E  E  L  N  G  E  E  Y  K  E  Q  G  P  V  E  M  V  E  D  V  F  E  E  E  E  Y  P  E  D  C  F  P  P  N  C  Y  K  K  F  P  200
                         481              .           5           .           .           .           .           :           .           .           .           .           6 600
 1 MT379844.1_X1           GAAGAACTTAATGGTGAAGAATACAAAGAACAAGGTCCTGTTGAAATGGTAGAAGACGTATTTGAGGAAGAAGAATATCCCGAAGACTGTTTTCCGCCGAACTGTTATAAAAAATTTCCA
 2 MT379844.1_X2           GAAGAACTTAATGGTGAAGAATACAAAGAACAAGGTCCTGTTGAAATGGTAGAAGACGTATTTGAGGAAGAAGAATATCCCGAAGACTGTTTTCCGCCGAACTGTTATAAAAAATTTCCA
 3 MT379844.1_X3           GAAGAACTTAATGGTGAAGAATACAAAGAACAAGGTCCTGTTGAAATGGTAGAAGACGTATTTGAGGAAGAAGAATATCCCGAAGACTGTTTTCCGCCGAACTGTTATAAAAAATTTCCA
 4 MW759894.1_ND_B1A-34    GAAGAACTTAATGGGGAAGAATACAAAGAACAAGGTCCTGTTGAAATGGTAGAAGACGTATTTGAGGAAGAAGAATATCCCGAAGACTGTTTTCCGCCGAACTGTTATAAAAAATTTCCA SUS
 5 MW759895.1_X4_B1B-34    GAAGAACTTAATGGTGAAGAATACAAAGAACAAGGTCCTGTTGAAATGGTAGAAGACGTATTTGAGGAAGAAGAATATCCCGAAGACTGTTTTCCGCCGAACTGTTATAAAAAATTTCCA SUS
 6 MW759896.1_ND_B3B-34    GAAGAACTTAATGGTGAAGAATACAAAGAACAAGGTCCTGTTGAAATGGTAGAAGACGTATTTGAGGAAGAAGAATATCCCGAAGACTGTTTTCCGCCGAACTGTTATAAAAAATTTCCA SUS
 7 MW759897.1_ND_B4B-34    GAAGAACTTAATGGTGAAGAATACAAAGAACAAGGTCCTGTTGAAATGGTAGAAGACGTATTTGAGGAAGAAGAATATCCCGAAGACTGTTTTCCGCCGAACTGTTATAAAAAATTTCCA SUS
 8 MW759898.1_X4_MN1-4-34  GAAGAACTTAATGGTGAAGAATACAAAGAACAAGGTCCTGTTGAAATGGTAGAAGACGTATTTGAGGAAGAAGAATATCCCGAAGACTGTTTTCCGCCGAACTGTTATAAAAAATTTCCA RES
 9 MW759899.1_X4_MN1-5-34  GAAGAACTTAATGGTGAAGAATACAAAGAACAAGGTCCTGTTGAAATGGTAGAAGACGTATTTGAGGAAGAAGAATATCCCGAAGACTGTTTTCCGCCGAACTGTTATAAAAAATTTCCA RES
10 MW759900.1_ND_MN1-7-34  GAAGAACTTAATGGTGAAGAATACAAAGAACAAGGTCCTGTTGAAATGGTAGAAGACGTATTTGAGGAAGAAGAATATCCCGAAGACTGTTTTCCGCCGAACTGTTATAAAAAATTTCCA RES
11 MW759901.1_ND_MN2-2-34  GAAGAACTTAATGGTGAAGAATACAAAGAACAAGGTCCTGTTGAAATGGTAGAAGACGTATTTGAGGAAGAAGAATATCCCGAAGACTGTTTTCCGCCGAACTGTTATAAAAAATTTCCA RES
12 MW759902.1_ND_MN2-9-34  GAAGAACTTAATGGTGAAGAATACAAAGAACAAGGTCCTGTTGAAATGGTAGAAGACGTATTTGAGGAAGAAGAATATCCCGAAGACTGTTTTCCGCCGAACTGTTATAAAAAATTTCCA RES
13 MW759903.1_nd_Suth5-34  GAAGAACTTAATGGTGAAGAATACAAAGAACAAGGTCCTGTTGAAATGGTAGAAGACGTATTTGAGGAAGAAGAATATCCCGAAGACTGTTTTCCGCCGAACTGTTATAAAAAATTTCCA RES
14 MW759904.1_X4_Suth6-34  GAAGAACTTAATGGTGAAGAATACAAAGAACAAGGTCCTGTTGAAATGGTAGAAGACGTATTTGAGGAAGAAGAATATCCCGAAGACTGTTTTCCGCCGAACTGTTATAAAAAATTTCCA RES
```

```
                                                                                                ......................DIII S1....... ...........>
                            F  L  A  G  D  D  E  T  P  F  W  L  G  W  G  Q  L  R  L  K  T  F  Q  L  I  E  N  K  Y  F  E  T  A  V  I  T  M  I  L  L  240
                       601          .         .         .         .         :         .         .         .         .         7         .         . 720
 1 MT379844.1_X1             TTCCTGGCTGGCGATGATGAAACGCCATTTTGGTTAGGGTGGGGACAGCTCAGACTGAAAACATTTCAACTTATAGAAAACAAATATTTTGAAACTGCTGTGATTACTATGATTTTGCTT
 2 MT379844.1_X2             TTCCTGGCTGGCGATGATGAAACGCCATTTTGGTTAGGGTGGGGACAGCTCAGACTGAAAACATTTCAACTTATAGAAAACAAATATTTTGAAACTGCTGTGATTACTATGATTTTGCTT
 3 MT379844.1_X3             TTCCTGGCTGGCGATGATGAAACGCCATTTTGGTTAGGGTGGGGACAGCTCAGACTGAAAACATTTCAACTTATAGAAAACAAATATTTTGAAACTGCTGTGATTACTATGATTTTGCTT
 4 MW759894.1_ND_B1A-34      TTCCTGGCTGGCGATGATGAAACGCCATTTTGGTTAGGGTGGGGACAGCTCAGACTGAAAACATTTCAACTTATAGAAAACAAATATTTTGAAACTGCTGTGATTACTATGATTTTGCTT SUS
 5 MW759895.1_X4_B1B-34      TTCCTGGCTGGCGATGATGAAACGCCATTTTGGTTAGGGTGGGGACAGCTCAGACTGAAAACATTTCAACTTATAGAAAACAAATATTTTGAAACTGCTGTGATTACTATGATTTTGCTT SUS
 6 MW759896.1_ND_B3B-34      TTCCTGGCTGGCGATGATGAAACGCCATTTTGGTTAGGGTGGGGACAGCTCAGACTGAAAACATTTCAACTTATAGAAAACAAATATTTTGAAACTGCTGTGATTACTATGATTTTGCTT SUS
 7 MW759897.1_ND_B4B-34      TTCCTGGCTGGCGATGATGAAACGCCATTTTGGTTAGGGTGGGGACAGCTCAGACTGAAAACATTTCAACTTATAGAAAACAAATATTTTGAAACTGCTGTGATTACTATGATTTTGCTT SUS
 8 MW759898.1_X4_MN1-4-34    TTCCTGGCTGGCGATGATGAAACGCCATTTTGGTTAGGGTGGGGACAGCTCAGACTGAAAACATTTCAACTTATAGAAAACAAATATTTTGAAACTGCTGTGATTACTATGATTTTGCTT RES
 9 MW759899.1_X4_MN1-5-34    TTCCTGGCTGGCGATGATGAAACGCCATTTTGGTTAGGGTGGGGACAGCTCAGACTGAAAACATTTCAACTTATAGAAAACAAATATTTTGAAACTGCTGTGATTACTATGATTTTGCTT RES
10 MW759900.1_ND_MN1-7-34    TTCCTGGCTGGCGATGATGAAACGCCATTTTGGTTAGGGTGGGGACAGCTCAGACTGAAAACATTTCAACTTATAGAAAACAAATATTTTGAAACTGCTGTGATTACTATGATTTTGCTT RES
11 MW759901.1_ND_MN2-2-34    TTCCTGGCTGGCGATGATGAAACGCCATTTTGGTTAGGGTGGGGACAGCTCAGACTGAAAACATTTCAACTTATAGAAAACAAATATTTTGAAACTGCTGTGATTACTATGATTTTGCTT RES
12 MW759902.1_ND_MN2-9-34    TTCCTGGCTGGCGATGATGAAACGCCATTTTGGTTAGGGTGGGGACAGCTCAGACTGAAAACATTTCAACTTATAGAAAACAAATATTTTGAAACTGCTGTGATTACTATGATTTTGCTT RES
13 MW759903.1_nd_Suth5-34    TTCCTGGCTGGCGATGATGAAACGCCATTTTGGTTAGGGTGGGGACAGCTCAGACTGAAAACATTTCAACTTATAGAAAACAAATATTTTGAAACTGCTGTGATTACTATGATTTTGCTT RES
14 MW759904.1_X4_Suth6-34    TTCCTGGCTGGCGATGATGAAACGCCATTTTGGTTAGGGTGGGGACAGCTCAGACTGAAAACATTTCAACTTATAGAAAACAAATATTTTGAAACTGCTGTGATTACTATGATTTTGCTT RES

                           <..DIII S1..                                                ........................DIII S2........................>
                            S  S  L  A  L  A  L  E  D  V  H  L  Q  K  R  P  V  L  Q  D  I  L  Y  Y  M  D  R  I  F  T  V  I  F  F  L  E  M  L  I  K  280
                       721          .         .         :         .         .         .         .         8         .         .         .         . 840
 1 MT379844.1_X1             AGCAGTTTGGCGCTGGCGCTAGAAGATGTACACCTCCAGAAACGACCGGTTTTACAAGACATTTTGTATTACATGGATCGAATATTCACTGTTATATTCTTTTTGGAGATGTTGATCAAG
 2 MT379844.1_X2             AGCAGTTTGGCGCTGGCGCTAGAAGATGTACACCTCCAGAAACGACCGGTTTTACAAGACATTTTGTATTACATGGATCGAATATTCACTGTTATATTCTTTTTGGAGATGTTGATCAAG
 3 MT379844.1_X3             AGCAGTTTGGCGCTGGCGCTAGAAGATGTACACCTCCAGAAACGACCGGTTTTACAAGACATTTTGTATTACATGGATCGAATATTCACTGTTATATTCTTTTTGGAGATGTTGATCAAG
 4 MW759894.1_ND_B1A-34      AGCAGTTTGGCGCTGGCGCTAGAAGATGTACACCTCCAGAAACGACCGGTTTTACAAGACATTTTGTATTACATGGATCGAATATTCACTGTTATATTCTTTTTGGAGATGTTGATCAAG SUS
 5 MW759895.1_X4_B1B-34      AGCAGTTTGGCGCTGGCGCTAGAAGATGTACACCTCCAGAAACGACCGGTTTTACAAGACATTTTGTATTACATGGATCGAATATTCACTGTTATATTCTTTTTGGAGATGTTGATCAAG SUS
 6 MW759896.1_ND_B3B-34      AGCAGTTTGGCGCTGGCGCTAGAAGATGTACACCTCCAGAAACGACCGGTTTTACAAGACATTTTGTATTACATGGATCGAATATTCACTGTTATATTCTTTTTGGAGATGTTGATCAAG SUS
 7 MW759897.1_ND_B4B-34      AGCAGTTTGGCGCTGGCGCTAGAAGATGTACACCTCCAGAAACGACCGGTTTTACAAGACATTTTGTATTACATGGATCGAATATTCACTGTTATATTCTTTTTGGAGATGTTGATCAAG SUS
 8 MW759898.1_X4_MN1-4-34    AGCAGTTTGGCGCTGGCGCTAGAAGATGTACACCTCCAGAAACGACCGGTTTTACAAGACATTTTGTATTACATGGATCGAATATTCACTGTTATATTCTTTTTGGAGATGTTGATCAAG RES
 9 MW759899.1_X4_MN1-5-34    AGCAGTTTGGCGCTGGCGCTAGAAGATGTACACCTCCAGAAACGACCGGTTTTACAAGACATTTTGTATTACATGGATCGAATATTCACTGTTATATTCTTTTTGGAGATGTTGATCAAG RES
10 MW759900.1_ND_MN1-7-34    AGCAGTTTGGCGCTGGCGCTAGAAGATGTACACCTCCAGAAACGACCGGTTTTACAAGACATTTTGTATTACATGGATCGAATATTCACTGTTATATTCTTTTTGGAGATGTTGATCAAG RES
11 MW759901.1_ND_MN2-2-34    AGCAGTTTGGCGCTGGCGCTAGAAGATGTACACCTCCAGAAACGACCGGTTTTACAAGACATTTTGTATTACATGGATCGAATATTCACTGTTATATTCTTTTTGGAGATGTTGATCAAG RES
12 MW759902.1_ND_MN2-9-34    AGCAGTTTGGCGCTGGCGCTAGAAGATGTACACCTCCAGAAACGACCGGTTTTACAAGACATTTTGTATTACATGGATCGAATATTCACTGTTATATTCTTTTTGGAGATGTTGATCAAG RES
13 MW759903.1_nd_Suth5-34    AGCAGTTTGGCGCTGGCGCTAGAAGATGTACACCTCCAGAAACGACCGGTTTTACAAGACATTTTGTATTACATGGATCGAATATTCACTGTTATATTCTTTTTGGAGATGTTGATCAAG RES
14 MW759904.1_X4_Suth6-34    AGCAGTTTGGCGCTGGCGCTAGAAGATGTACACCTCCAGAAACGACCGGTTTTACAAGACATTTTGTATTACATGGATCGAATATTCACTGTTATATTCTTTTTGGAGATGTTGATCAAG RES

                           <...DIII S2....                     ..............DIII S3.........>
                            W  L  A  L  G  F  R  N  Y  F  T  N  A  W  C  W  L  D  F  I  I  V  M  L  S  L  V  N  L  A  A  I  W  F  G  A  A  D  I  P  320
                       841          :         .         .         .         .         9         .         .         .         .         :         . 960
 1 MT379844.1_X1             TGGTTGGCATTGGGTTTTCGCAATTATTTTACAAATGCCTGGTGTTGGCTGGACTTCATAATTGTTATGTTGTCATTAGTTAACTTGGCAGCGATATGGTTTGGGGCGGCAGATATACCA
 2 MT379844.1_X2             TGGTTGGCATTGGGTTTTCGCAATTATTTTACAAATGCCTGGTGTTGGCTGGACTTCATAATTGTTATG+++++++++++++++++++++++++++++++++++++++++++++++++++
 3 MT379844.1_X3             TGGTTGGCATTGGGTTTTCGCAATTATTTTACAAATGCCTGGTGTTGGCTGGACTTCATAATTGTTATGTTGTCATTAGTTAACTTGGCAGCGATATGGTTTGGGGCGGCAGATATACCA
 4 MW759894.1_ND_B1A-34      TGGTTGGCATTGGGTTTTCGCAATTATTTTACAAATGCCTGGTGTTGGCTGGACTTCATAATTGTTATG+++++++++++++++++++++++++++++++++++++++++++++++++++ SUS
 5 MW759895.1_X4_B1B-34      TGGTTGGCATTGGGTTTTCGCAATTATTTTACAAATGCCTGGTGTTGGCTGGACTTCATAATTGTTATG+++++++++++++++++++++++++++++++++++++++++++++++++++ SUS
 6 MW759896.1_ND_B3B-34      TGGTTGGCATTGGGTTTTCGCAATTATTTTACAAATGCCTGGTGTTGGCTGGACTTCATAATTGTTATG+++++++++++++++++++++++++++++++++++++++++++++++++++ SUS
 7 MW759897.1_ND_B4B-34      TGGTTGGCATTGGGTTTTCGCAATTATTTTACAAATGCCTGGTGTTGGCTGGACTTCATAATTGTTATG+++++++++++++++++++++++++++++++++++++++++++++++++++ SUS
 8 MW759898.1_X4_MN1-4-34    TGGTTGGCATTGGGTTTTCGCAATTATTTTACAAATGCCTGGTGTTGGCTGGACTTCATAATTGTTATG+++++++++++++++++++++++++++++++++++++++++++++++++++ RES
 9 MW759899.1_X4_MN1-5-34    TGGTTGGCATTGGGTTTTCGCAATTATTTTACAAATGCCTGGTGTTGGCTGGACTTCATAATTGTTATG+++++++++++++++++++++++++++++++++++++++++++++++++++ RES
10 MW759900.1_ND_MN1-7-34    TGGTTGGCATTGGGTTTTCGCAATTATTTTACAAATGCCTGGTGTTGGCTGGACTTCATAATTGTTATG+++++++++++++++++++++++++++++++++++++++++++++++++++ RES
11 MW759901.1_ND_MN2-2-34    TGGTTGGCATTGGGTTTTCGCAATTATTTTACAAATGCCTGGTGTTGGCTGGACTTCATAATTGTTATG+++++++++++++++++++++++++++++++++++++++++++++++++++ RES
12 MW759902.1_ND_MN2-9-34    TGGTTGGCATTGGGTTTTCGCAATTATTTTACAAATGCCTGGTGTTGGCTGGACTTCATAATTGTTATG+++++++++++++++++++++++++++++++++++++++++++++++++++ RES
13 MW759903.1_nd_Suth5-34    TGGTTGGCATTGGGTTTTCGCAATTATTTTACAAATGCCTGGTGTTGGCTGGACTTCATAATTGTTATG+++++++++++++++++++++++++++++++++++++++++++++++++++ RES
14 MW759904.1_X4_Suth6-34    TGGTTGGCATTGGGTTTTCGCAATTATTTTACAAATGCCTGGTGTTGGCTGGACTTCATAATTGTTATG+++++++++++++++++++++++++++++++++++++++++++++++++++ RES
```

```
                                                                              <...........DIII S3.............        ...
                     A  F  R  S  M  R  T  L  R  A  L  R  P  L  R  A  V  S  R  W  E  G  M  R  V  S  L  I  N  F  V  A  A  L  L  G  A  S  G  I  360
                 961          .          .          0          .          .          .          .          :          .          .          . 1080
 1 MT379844.1_X1            GCGTTCAGGTCAATGAGAACTCTTCGGGCCTTAAGACCCCTGAGAGCGGTATCTCGTTGGGAGGGTATGAGAGTTTCTCTGATAAATTTTGTTGCTGCCTTGTTGGGAGCCAGTGGAATC
 2 MT379844.1_X2            ++++++++++++++++++++++++++++++++++++++++++++++++++++++++++++++++++++++++GTTTCTCTGATAAATTTTGTTGCTGCCTTGTTGGGAGCCAGTGGAATC
 3 MT379844.1_X3            GCGTTCAGGTCAATGAGAACTCTTCGGGCCTTAAGACCCCTGAGAGCGGTATCTCGTTGGGAGGGTATGAGAGTTTCTCTGATAAATTTTGTTGCTGCCTTGTTGGGAGCCAGTGGAATC
 4 MW759894.1_ND_B1A-34     ++++++++++++++++++++++++++++++++++++++++++++++++++++++++++++++++++++++++GTTTCTCTGATAAATTTTGTTGCTGCCTTGTTGGGAGCCAGTGGAATC SUS
 5 MW759895.1_X4_B1B-34     ++++++++++++++++++++++++++++++++++++++++++++++++++++++++++++++++++++++++GTTTCTCTGATAAATTTTGTTGCTGCCTTGTTGGGAGCCAGTGGAATC SUS
 6 MW759896.1_ND_B3B-34     ++++++++++++++++++++++++++++++++++++++++++++++++++++++++++++++++++++++++GTTTCTCTGATAAATTTTGTTGCTGCCTTGTTGGGAGCCAGTGGAATC SUS
 7 MW759897.1_ND_B4B-34     ++++++++++++++++++++++++++++++++++++++++++++++++++++++++++++++++++++++++GTTTCTCTGATAAATTTTGTTGCTGCCTTGTTGGGAGCCAGTGGAATC SUS
 8 MW759898.1_X4_MN1-4-34   ++++++++++++++++++++++++++++++++++++++++++++++++++++++++++++++++++++++++GTTTCTCTGATAAATTTTGTTGCTGCCTTGTTGGGAGCCAGTGGAATC RES
 9 MW759899.1_X4_MN1-5-34   ++++++++++++++++++++++++++++++++++++++++++++++++++++++++++++++++++++++++GTTTCTCTGATAAATTTTGTTGCTGCCTTGTTGGGAGCCAGTGGAATC RES
10 MW759900.1_ND_MN1-7-34   ++++++++++++++++++++++++++++++++++++++++++++++++++++++++++++++++++++++++GTGTCTCTGATAAATTTTGTTGCTGCCTTGTTGGGAGCCAGTGGAATC RES
11 MW759901.1_ND_MN2-2-34   ++++++++++++++++++++++++++++++++++++++++++++++++++++++++++++++++++++++++GTTTCTCTGATAAATTTTGTTGCTGCCTTGTTGGGAGCCAGTGGAATC RES
12 MW759902.1_ND_MN2-9-34   ++++++++++++++++++++++++++++++++++++++++++++++++++++++++++++++++++++++++GTTTCTCTGATAAATTTTGTTGCTGCCTTGTTGGGAGCCAGTGGAATC RES
13 MW759903.1_nd_Suth5-34   ++++++++++++++++++++++++++++++++++++++++++++++++++++++++++++++++++++++++GTTTCTCTGATAAATTTTGTTGCTGCCTTGTTGGGAGCCAGTGGAATC RES
14 MW759904.1_X4_Suth6-34   ++++++++++++++++++++++++++++++++++++++++++++++++++++++++++++++++++++++++GTTTCTCTGATAAATTTTGTTGCTGCCTTGTTGGGAGCCAGTGGAATC RES

                         <...........................DIII S4.......................
                     Q  A  F  K  T  M  R  T  L  R  A  L  R  P  L  R  A  M  A  R  M  Q  G  M  R  V  V  V  N  A  L  V  Q  A  I  P  S  I  F  N  400
                1081          .          1          .          .          .          :          .          .          .          .          2 1200
 1 MT379844.1_X1            CAAGCTTTCAAAACTATGCGAACTCTAAGAGCTTTGCGGCCACTGCGAGCTATGGCTAGAATGCAGGGTATGAGGGTGGTGGTTAACGCGTTAGTACAGGCCATTCCTAGTATTTTCAAC
 2 MT379844.1_X2            CAAGCTTTCAAAACTATGCGAACTCTAAGAGCTTTGCGGCCACTGCGAGCTATGGCTAGAATGCAGGGTATGAGGGTGGTGGTTAACGCGTTAGTACAGGCCATTCCTAGTATTTTCAAC
 3 MT379844.1_X3            CAAGCTTTCAAAACTATGCGAACTCTAAGAGCTTTGCGGCCACTGCGAGCTATGGCTAGAATGCAGGGTATGAGGGTGGTGGTTAACGCGTTAGTACAGGCCATTCCTAGTATTTTCAAC
 4 MW759894.1_ND_B1A-34     CAAGCTTTCAAAACTATGCGAACTCTAAGAGCTTTGCGGCCACTGCGAGCTATGGCTAGAATGCAGGGTATGAGGGTGGTGGTTAACGCGTTAGTACAGGCCATTCCTAGTATTTTCAAC SUS
 5 MW759895.1_X4_B1B-34     CAAGCTTTCAAAACTATGCGAACTCTAAGAGCTTTGCGGCCACTGCGAGCTATGGCTAGAATGCAGGGTATGAGGGTGGTGGTTAACGCGTTAGTACAGGCCATTCCTAGTATTTTCAAC SUS
 6 MW759896.1_ND_B3B-34     CAAGCTTTCAAAACTATGCGAACTCTAAGAGCTTTGCGGCCACTGCGAGCTATGGCTAGAATGCAGGGTATGAGGGTGGTGGTTAACGCGTTAGTACAGGCCATTCCTAGTATTTTCAAC SUS
 7 MW759897.1_ND_B4B-34     CAAGCTTTCAAAACTATGCGAACTCTAAGAGCTTTGCGGCCACTGCGAGCTATGGCTAGAATGCAGGGTATGAGGGTGGTGGTTAACGCGTTAGTACAGGCCATTCCTAGTATTTTCAAC SUS
 8 MW759898.1_X4_MN1-4-34   CAAGCTTTCAAAACTATGCGAACTCTAAGAGCTTTGCGGCCACTGCGAGCTATGGCTAGAATGCAGGGTATGAGGGTGGTGGTTAACGCGTTAGTACAGGCCATTCCTAGTATTTTCAAC RES
 9 MW759899.1_X4_MN1-5-34   CAAGCTTTCAAAACTATGCGAACTCTAAGAGCTTTGCGGCCACTGCGAGCTATGGCTAGAATGCAGGGTATGAGGGTGGTGGTTAACGCGTTAGTACAGGCCATTCCTAGTATTTTCAAC RES
10 MW759900.1_ND_MN1-7-34   CAAGCTTTCAAAACTATGCGAACTCTAAGAGCTTTGCGACCACTGCGAGCTATGGCTAGAATGCAGGGTATGAGGGTGGTGGTTAACGCGTTAGTACAGGCCATTCCTAGTATTTTCAAC RES
11 MW759901.1_ND_MN2-2-34   CAAGCTTTCAAAACTATGCGAACTCTAAGAGCTTTGCGGCCACTGCGAGCTATGGCTAGAATGCAGGGTATGAGGGTGGTGGTTAACGCGTTAGTACAGGCCATTCCTAGTATTTTCAAC RES
12 MW759902.1_ND_MN2-9-34   CAAGCTTTCAAAACTATGCGAACTCTAAGAGCTTTGCGGCCACTGCGAGCTATGGCTAGAATGCAGGGTATGAGGGTGGTGGTTAACGCGTTAGTACAGGCCATTCCTAGTATTTTCAAC RES
13 MW759903.1_nd_Suth5-34   CAAGCTTTCAAAACTATGCGAACTCTAAGAGCTTTGCGGCCACTGCGAGCTATGGCTAGAATGCAGGGTATGAGGGTGGTGGTTAACGCGTTAGTACAGGCCATTCCTAGTATTTTCAAC RES
14 MW759904.1_X4_Suth6-34   CAAGCTTTCAAAACTATGCGAACTCTAAGAGCTTTGCGGCCACTGCGAGCTATGGCTAGAATGCAGGGTATGAGGGTGGTGGTTAACGCGTTAGTACAGGCCATTCCTAGTATTTTCAAC RES

                         ............................DIII S5.........................
                     V  L  L  V  C  L  I  F  W  L  I  F  A  I  M  G  V  Q  L  F  A  G  K  Y  Y  K  C  V  D  N  T  G  K  T  L  N  H  E  I  I  440
                1201          .V1424S   .  L14309V .          .          :          .          .          .          .          3          . 1320
 1 MT379844.1_X1            GTGTTACTGGTGTGTCTGATATTTTGGTTAATTTTTGCTATAATGGGTGTACAACTTTTCGCCGGAAAATATTACAAGTGTGTTGATAATACTGGGAAAACATTGAACCACGAAATTATA
 2 MT379844.1_X2            GTGTTACTGGTGTGTCTGATATTTTGGTTAATTTTTGCTATAATGGGTGTACAACTTTTCGCCGGAAAATATTACAAGTGTGTTGATAATACTGGGAAAACATTGAACCACGAAATTATA
 3 MT379844.1_X3            GTGTTACTGGTGTGTCTGATATTTTGGTTAATTTTTGCTATAATGGGTGTACAACTTTTCGCCGGAAAATATTACAAGTGTGTTGATAATACTGGGAAAACATTGAACCACGAAATTATA
 4 MW759894.1_ND_B1A-34     GTGTTACTGGTGTGTCTGATATTTTGGTTAATTTTTGCTATAATGGGTGTACAACTTTTCGCCGGAAAATATTACAAGTGTGTTGATAATACTGGGAAAACATTGAACCACGAAATTATA SUS
 5 MW759895.1_X4_B1B-34     GTGTTACTGGTGTGTCTGATATTTTGGTTAATTTTTGCTATAATGGGTGTACAACTTTTCGCCGGAAAATATTACAAGTGTGTTGATAATACTGGGAAAACATTGAACCACGAAATTATA SUS
 6 MW759896.1_ND_B3B-34     GTGTTACTGGTGTGTCTGATATTTTGGTTAATTTTTGCTATAATGGGTGTACAACTTTTCGCCGGAAAATATTACAAGTGTGTTGATAATACTGGGAAAACATTGAACCACGAAATTATA SUS
 7 MW759897.1_ND_B4B-34     GTGTTACTGGTGTGTCTGATATTTTGGGTAATTTTTGCTATAATGGGTGTACAACTTTTCGCCGGAAAATATTACAAGTGTGTTGATAATACTGGGAAAACATTGAACCACGAAATTATA SUS
 8 MW759898.1_X4_MN1-4-34   GTGTTACTGGTGTGTCTGATATTTTGGTTAATTTTTGCTATAATGGGTGTACAACTTTTCGCCGGAAAATATTACAAGTGTGTTGATAATACTGGGAAAACATTGAACCACGAAATTATA RES
 9 MW759899.1_X4_MN1-5-34   GTGTTACTGGTGTGTCTGATATTTTGGTTAATTTTTGCTATAATGGGTGTACAACTTTTCGCCGGAAAATATTACAAGTGTGTTGATAATACTGGGAAAACATTGAACCACGAAATTATA RES
10 MW759900.1_ND_MN1-7-34   GTGTTACTGGTGTGTCTGATATTTTGGTTAATTTTTGCTATAATGGGTGTACAACTTTTCGCCGGAAAATATTACAAGTGTGTTGATAATACTGGGAAAACATTGAACCACGAAATTATA RES
11 MW759901.1_ND_MN2-2-34   GTGTTACTGGGGTGTCTGATATTTTGGTTAATTTTTGCTATAATGGGTGTACAACTTTTCGCCGGAAAATATTACAAGTGTGTTGATAATACTGGGAAAACATTGAACCACGAAATTATA RES
12 MW759902.1_ND_MN2-9-34   GTGTTACTGGGGTGTCTGATATTTTGGGTAATTTTTGCTATAATGGGTGTACAACTTTTCGCCGGAAAATATTACAAGTGTGTTGATAATACTGGGAAAACATTGAACCACGAAATTATA RES
13 MW759903.1_nd_Suth5-34   GTGTTACTGGTGTGTCTGATATTTTGGTTAATTTTTGCTATAATGGGTGTACAACTTTTCGCCGGAAAATATTACAAGTGTGTTGATAATACTGGGAAAACATTGAACCACGAAATTATT RES
14 MW759904.1_X4_Suth6-34   GTGTTACTGGTGTGTCTGATATTTTGGTTAATTTTTGCTATAATGGGTGTACAACTTTTCGCCGGAAAATATTACAAGTGTGTTGATAATACTGGGAAAACATTGAACCACGAAATTATA RES
```

```
                            P  D  K  N  V  C  L  A  E  N  Y  K  W  E  N  S  K  M  N  F  D  H  V  G  N  A  Y  L  C  L  F  Q  V  A  T  F  N  G  W  M   480
                     1321          .         .         :         .         .         .         .         4         .         .         .         . 1440
 1 MT379844.1_X1             CCAGATAAAAACGTCTGTTTAGCTGAGAACTATAAATGGAAAACTCTAAAATGAACTTTGATCACGTGGGAAACGCGTATCTTTGCCTTTTCCAAGTAGCTACTTTTAATGGATGGATG
 2 MT379844.1_X2             CCAGATAAAAACGTCTGTTTAGCTGAGAACTATAAATGGAAAACTCTAAAATGAACTTTGATCACGTGGGAAACGCGTATCTTTGCCTTTTCCAAGTAGCTACTTTTAATGGATGGATG
 3 MT379844.1_X3             CCAGATAAAAACGTCTGTTTAGCTGAGAACTATAAATGGAAAACTCTAAAATGAACTTTGATCACGTGGGAAACGCGTATCTTTGCCTTTTCCAAGTAGCTACTTTTAATGGATGGATG
 4 MW759894.1_ND_B1A-34      CCAGATAAAAACGTCTGTTTAGCTGAAAACTATAAATGGAAAACTCTAAAATGAACTTTGATCACGTGGGAAACGCGTATCTTTGCCTTTTCCAAGTAGCTACTTTTAATGGATGGATG SUS
 5 MW759895.1_X4_B1B-34      CCAGATAAAAACGTCTGTTTAGCTGAAAACTATAAATGGAAAACTCTAAAATGAACTTCGATCACGTGGGAAACGCGTATCTTTGCCTTTTCCAAGTAGCTACTTTTAATGGATGGATG SUS
 6 MW759896.1_ND_B3B-34      CCAGATAAAAACGTCTGTTTAGCTGAAAACTATAAATGGAAAACTCTAAAATGAACTTCGATCACGTGGGAAACGCGTATCTTTGCCTTTTCCAAGTAGCTACTTTTAATGGATGGATG SUS
 7 MW759897.1_ND_B4B-34      CCAGATAAAAACGTCTGTTTAGCTGAAAACTATAAATGGAAAACTCTAAAATGAACTTCGATCACGTGGGAAACGCGTATCTTTGCCTTTTCCAAGTAGCTACTTTTAATGGATGGATG SUS
 8 MW759898.1_X4_MN1-4-34    CCAGATAAAAACGTCTGTTTAGCTGAAAACTATAAATGGAAAACTCTAAAATGAACTTCGATCACGTGGGAAACGCGTATCTTTGCCTTTTCCAAGTAGCTACTTTTAATGGATGGATG RES
 9 MW759899.1_X4_MN1-5-34    CCAGATAAAAACGTCTGTTTAGCTGAAAACTATAAATGGAAAACTCTAAAATGAACTTCGATCACGTGGGAAACGCGTATCTTTGCCTTTTCCAAGTAGCTACTTTTAATGGATGGATG RES
10 MW759900.1_ND_MN1-7-34    CCAGATAAAAACGTCTGTTTAGCTGAAAACTATAAATGGAAAACTCTAAAATGAACTTCGATCACGTGGGAAACGCGTATCTTTGCCTTTTCCAAGTAGCTACTTTTAATGGATGGATG RES
11 MW759901.1_ND_MN2-2-34    CCAGATAAAAACGTCTGTTTAGCTGAAAACTATAAATGGAAAACTCTAAAATGAACTTCGATCACGTGGGAAACGCGTATCTTTGCCTTTTCCAAGTAGCTACTTTTAATGGATGGATG RES
12 MW759902.1_ND_MN2-9-34    CCAGATAAAAACGTCTGTTTAGCTGAAAACTATAAATGGAAAACTCTAAAATGAACTTCGATCACGTGGGAAACGCGTATCTTTGCCTTTTCCAAGTAGCTACTTTTAATGGATGGATG RES
13 MW759903.1_nd_Suth5-34    CCAGATAAAAACGTCTGTTTAGCTGAAAACTATAAATGGAAAACTCTAAAATGAACTTCGATCACGTGGGAAACGCGTATCTTTGCCTTTTCCAAGTAGCTACTTTTAATGGATGGATG RES
14 MW759904.1_X4_Suth6-34    CCAGATAAAAACGTCTGTTTAGCTGAAAACTATAAATGGAAAACTCTAAAATGAACTTCGATCACGTGGGAAACGCGTATCTTTGCCTTTTCCAAGTAGCTACTTTTAATGGATGGATG RES

                                                                                                    .......................DIII S6..................>
                            E  I  M  R  D  A  V  D  S  R  D  T  H  G  K  Q  P  I  R  E  I  N  N  Y  M  Y  F  Y  F  V  F  F  I  I  F  G  S  F  F  T   520
                     1441          :         .         .         .         .         5         .         .         .         .         :         . 1560
 1 MT379844.1_X1             GAAATAATGAGGGACGCAGTAGATTCAAGAGACACGCACGGGAAACAGCCAATTCGTGAAATCAATAATTATATGTATTTTTATTTTGTTTTTTTTATTATTTTTGGTTCATTTTTTACT
 2 MT379844.1_X2             GAAATAATGAGGGACGCAGTAGATTCAAGAGACACGCACGGGAAACAGCCAATTCGTGAAATCAATAATTATATGTATTTTTATTTTGTTTTTTTTATTATTTTTGGTTCATTTTTTACT
 3 MT379844.1_X3             GAAATAATGAGGGACGCAGTAGATTCAAGAGACACGCACGGGAAACAGCCAATTCGTGAAATCAATAATTATATGTATTTTTATTTTGTTTTTTTTATTATTTTTGGTTCATTTTTTACT
 4 MW759894.1_ND_B1A-34      GAAATAATGAGGGACGCAGTAGATTCAAGAGACACGCACGGGAAACAGCCAATTCGTGAAATCAATAATTATATGTATTTTTATTTTGTTTTTTTTATTATTTTTGGTTCATTTTTTACT SUS
 5 MW759895.1_X4_B1B-34      GAAATAATGAGGGACGCAGTAGATTCAAGAGACACGCACGGGAAACAGCCAATTCGTGAAATCAATAATTATATGTATTTTTATTTTGTTTTTTTTATTATTTTTGGTTCATTTTTTACT SUS
 6 MW759896.1_ND_B3B-34      GAAATAATGAGGGACGCAGTAGATTCAAGAGACACGCACGGGAAACAGCCAATTCGTGAAATCAATAATTATATGTATTTTTATTTTGTTTTTTTTATTATTTTTGGTTCATTTTTTACT SUS
 7 MW759897.1_ND_B4B-34      GAAATAATGAGGGACGCAGTAGATTCAAGAGACACGCACGGGAAACAGCCAATTCGTGAAATCAATAATTATATGTATTTTTATTTTGTTTTTTTTATTATTTTTGGTTCATTTTTTACT SUS
 8 MW759898.1_X4_MN1-4-34    GAAATAATGAGGGACGCAGTAGATTCAAGAGACACGCACGGGAAACAGCCAATTCGTGAAATCAATAATTATATGTATTTTTATTTTGTTTTTTTTATTATTTTTGGTTCATTTTTTACT RES
 9 MW759899.1_X4_MN1-5-34    GAAATAATGAGGGACGCAGTAGATTCAAGAGACACGCACGGGAAACAGCCAATTCGTGAAATCAATAATTATATGTATTTTTATTTTGTTTTTTTTATTATTTTTGGTTCATTTTTTACT RES
10 MW759900.1_ND_MN1-7-34    GAAATAATGAGGGACGCAGTAGATTCAAGAGACACGCACGGGAAACAGCCAATTCGTGAAATCAATAATTATATGTATTTTTATTTTGTTTTTTTTATTATTTTTGGTTCATTTTTTACT RES
11 MW759901.1_ND_MN2-2-34    GAAATAATGAGGGACGCAGTAGATTCAAGAGACACGCACGGGAAACAGCCAATTCGTGAAATCAATAATTATATGTATTTTTATTTTGTTTTTTTTATTATTTTTGGTTCATTTTTTACT RES
12 MW759902.1_ND_MN2-9-34    GAAATAATGAGGGACGCAGTAGATTCAAGAGACACGCACGGGAAACAGCCAATTCGTGAAATCAATAATTATATGTATTTTTATTTTGTTTTTTTTATTATTTTTGGTTCATTTTTTACT RES
13 MW759903.1_nd_Suth5-34    GAAATAATGAGGGACGCAGTAGATTCAAGAGACACGCACGGGAAACAGCCAATTCGTGAAATCAATAATTATATGTATTTTTATTTTGTTTTTTTTATTATTTTTGGTTCATTTTTTACT RES
14 MW759904.1_X4_Suth6-34    GAAATAATGAGGGACGCAGTAGATTCAAGAGACACGCACGGGAAACAGCCAATTCGTGAAATCAATAATTATATGTATTTTTATTTTGTTTTTTTTATTATTTTTGGTTCATTTTTTACT RES

                          <.........DIII S6..........
                            L  N  L  F  I  G  V  I  I  D  N  F  N  E  Q  K  K  K  T  G  A  S  L  E  M  F  M  T  E  D  Q  K  K  Y  Y  N  A  M  K  K   560
                     1561          .         .         .         6         .         .         .         .         :         .         .         . 1680
 1 MT379844.1_X1             CTTAATTTATTCATTGGTGTGATCATTGACAATTTCAACGAACAGAAGAAAAAAACA+++GCTTCTCTTGAAATGTTTATGACGGAGGACCAAAAGAAATATTACAATGCTATGAAAAAA
 2 MT379844.1_X2             CTTAATTTATTCATTGGTGTGATCATTGACAATTTCAACGAACAGAAGAAAAAAACAGGTGCTTCTCTTGAAATGTTTATGACGGAGGACCAAAAGAAATATTACAATGCTATGAAAAAA
 3 MT379844.1_X3             CTTAATTTATTCATTGGTGTGATCATTGACAATTTCAACGAACAGAAGAAAAAAACAGGTGCTTCTCTTGAAATGTTTATGACGGAGGACCAAAAGAAATATTACAATGCTATGAAAAAA
 4 MW759894.1_ND_B1A-34      CTTAATTTATTCATTGGTGTGATCATTGACAATTTCAACGAACAGAAGAAAAAAACAGGTGCTTCTCTTGAAATGTTTATGACGGAGGACCAAAAGAAATATTACAATGCTATGAAAAAA SUS
 5 MW759895.1_X4_B1B-34      CTTAATTTATTCATTGGTGTGATCATTGACAATTTCAACGAACAGAAGAAAAAAACAGGTGCTTCTCTTGAAATGTTTATGACGGAGGACCAAAAGAAATATTACAATGCTATGAAAAAA SUS
 6 MW759896.1_ND_B3B-34      CTTAATTTATTCATTGGTGTGATCATTGACAATTTCAACGAACAGAAGAAAAAAACAGGTGCTTCTCTTGAAATGTTTATGACGGAGGACCAAAAGAAATATTACAATGCTATGAAAAAA SUS
 7 MW759897.1_ND_B4B-34      CTTAATTTATTCATTGGTGTGATCATTGACAATTTCAACGAACAGAAGAAAAAAACAGGTGCTTCTCTTGAAATGTTTATGACGGAGGACCAAAAGAAATATTACAATGCTATGAAAAAA SUS
 8 MW759898.1_X4_MN1-4-34    CTTAATTTATTCATTGGTGTGATCATTGACAATTTCAACGAACAGAAGAAAAAAACAGGTGCTTCTCTTGAAATGTTTATGACGGAGGACCAAAAGAAATATTACAATGCTATGAAAAAA RES
 9 MW759899.1_X4_MN1-5-34    CTTAATTTATTCATTGGTGTGATCATTGACAATTTCAACGAACAGAAGAAAAAAACAGGTGCTTCTCTTGAAATGTTTATGACGGAGGACCAAAAGAAATATTACAATGCTATGAAAAAA RES
10 MW759900.1_ND_MN1-7-34    CTTAATTTATTCATTGGTGTGATCATTGACAATTTCAACGAACAGAAGAAAAAAACAGGTGCTTCTCTTGAAATGTTTATGACGGAGGACCAAAAGAAATATTACAATGCTATGAAAAAA RES
11 MW759901.1_ND_MN2-2-34    CTTAATTTATTCATTGGTGTGATCATTGACAATTTCAACGAACAGAAGAAAAAAACAGGTGCTTCTCTTGAAATGTTTATGACGGAGGACCAAAAGAAATATTACAATGCTATGAAAAAA RES
12 MW759902.1_ND_MN2-9-34    CTTAATTTATTCATTGGTGTGATCATTGACAATTTCAACGAACAGAAGAAAAAAACAGGTGCTTCTCTTGAAATGTTTATGACGGAGGACCAAAAGAAATATTACAATGCTATGAAAAAA RES
13 MW759903.1_nd_Suth5-34    CTTAATTTATTCATTGGTGTGATCATTGACAATTTCAACGAACAGAAGAAAAAAACAGGTGCTTCTCTTGAAATGTTTATGACGGAGGACCAAAAGAAATATTACAATGCTATGAAAAAA RES
14 MW759904.1_X4_Suth6-34    CTTAATTTATTCATTGGTGTGATCATTGACAATTTCAACGAACAGAAGAAAAAAACAGGTGCTTCTCTTGAAATGTTTATGACGGAGGACCAAAAGAAATATTACAATGCTATGAAAAAA RES
```

```
                                                                                 <...........................DIV S1.................
                            M  S  S  K  K  P  L  K  A  I  P  R  P  R  W  R  P  Q  S  I  V  F  Q  I  V  T  D  K  K  F  D  M  L  I  M  L  F  I  G  L   600
                     1681         .        7        .        .        .        .        :        .        .        .        .        8 1800
 1 MT379844.1_X1          ATGAGTTCCAAAAAACCGCTTAAAGCAATTCCAAGACCAAGGTGGCGGCCACAATCTATTGTCTTCCAAATCGTAACAGACAAAAAGTTTGACATGCTAATCATGTTGTTCATCGGATTA
 2 MT379844.1_X2          ATGAGTTCCAAAAAACCGCTTAAAGCAATTCCAAGACCAAGGTGGCGGCCACAATCTATTGTCTTCCAAATCGTAACAGACAAAAAGTTTGACATGCTAATCATGTTGTTCATCGGATTA
 3 MT379844.1_X3          ATGAGTTCCAAAAAACCGCTTAAAGCAATTCCAAGACCAAGGTGGCGGCCACAATCTATTGTCTTCCAAATCGTAACAGACAAAAAGTTTGACATGCTAATCATGTTGTTCATCGGATTA
 4 MW759894.1_ND_B1A-34   ATGAGTTCCAAAAAACCGCTTAAAGCAATTCCAAGACCAAGGTGGCGGCCACAATCTATTGTCTTCCAAATCGTAACAGACAAAAAGTTTGACATGCTAATCATGTTGTTCATCGGATTA SUS
 5 MW759895.1_X4_B1B-34   ATGAGTTCCAAAAAACCGCTTAAAGCAATTCCAAGACCAAGGTGGCGGCCACAATCTATTGTCTTCCAAATCGTAACAGACAAAAAGTTTGACATGCTAATCATGTTGTTCATCGGATTA SUS
 6 MW759896.1_ND_B3B-34   ATGAGTTCCAAAAAACCGCTTAAAGCAATTCCAAGACCAAGGTGGCGGCCACAATCTATTGTCTTCCAAATCGTAACAGACAAAAAGTTTGACATGCTAATCATGTTGTTCATCGGATTA SUS
 7 MW759897.1_ND_B4B-34   ATGAGTTCCAAAAAACCGCTTAAAGCAATTCCAAGACCAAGGTGGCGGCCACAATCTATTGTCTTCCAAATCGTAACAGACAAAAAGTTTGACATGCTAATCATGTTGTTCATCGGATTA SUS
 8 MW759898.1_X4_MN1-4-34 ATGAGTTCCAAAAAACCGCTTAAAGCAATTCCAAGACCAAGGTGGCGGCCACAATCTATTGTCTTCCAAATCGTAACAGACAAAAAGTTTGACATGCTAATCATGTTGTTCATCGGATTA RES
 9 MW759899.1_X4_MN1-5-34 ATGAGTTCCAAAAAACCGCTTAAAGCAATTCCAAGACCAAGGTGGCGGCCACAATCTATTGTCTTCCAAATCGTAACAGACAAAAAGTTTGACATGCTAATCATGTTGTTCATCGGATTA RES
10 MW759900.1_ND_MN1-7-34 ATGAGTTCCAAAAAACCGCTTAAAGCAATTCCAAGACCAAGGTGGCGGCCACAATCTATTGTCTTCCAAATCGTAACAGACAAAAAGTTTGACATGCTAATCATGTTGTTCATCGGATTA RES
11 MW759901.1_ND_MN2-2-34 ATGAGTTCCAAAAAACCGCTTAAAGCAATTCCAAGACCAAGGTGGCGGCCACAATCTATTGTCTTCCAAATCGTAACAGACAAAAAGTTTGACATGCTAATCATGTTGTTCATCGGATTA RES
12 MW759902.1_ND_MN2-9-34 ATGAGTTCCAAAAAACCGCTTAAAGCAATTCCAAGACCAAGGTGGCGGCCACAATCTATTGTCTTCCAAATCGTAACAGACAAAAAGTTTGACATGCTAATCATGTTGTTCATCGGATTA RES
13 MW759903.1_nd_Suth5-34 ATGAGTTCCAAAAAACCGCTTAAAGCAATTCCAAGACCAAGGTGGCGGCCACAATCTATTGTCTTCCAAATCGTAACAGACAAAAAGTTTGACATGCTAATCATGTTGTTCATCGGATTA RES
14 MW759904.1_X4_Suth6-34 ATGAGTTCCAAAAAACCGCTTAAAGCAATTCCAAGACCAAGGTGGCGGCCACAATCTATTGTCTTCCAAATCGTAACAGACAAAAAGTTTGACATGCTAATCATGTTGTTCATCGGATTA RES

                          <.......DIV S1.......                       ..............................DIV S2...........................>
                            N  M  L  T  M  T  L  D  H  Y  Q  Q  T  K  L  F  T  D  V  L  E  R  L  N  Q  I  F  I  A  I  F  S  T  E  C  L  L  K  I  F   640
                     1801         .        .        .        .        :        .        .        .        .        9        .        . 1920
 1 MT379844.1_X1          AACATGTTAACAATGACACTTGACCATTACCAACAAACCAAACTTTTTACAGATGTATTAGAACGTCTTAACCAAATATTTATCGCCATATTTTCAACCGAATGTCTACTTAAAATATTC
 2 MT379844.1_X2          AACATGTTAACAATGACACTTGACCATTACCAACAAACCAAACTTTTTACAGATGTATTAGAACGTCTTAACCAAATATTTATCGCCATATTTTCAACCGAATGTCTACTTAAAATATTC
 3 MT379844.1_X3          AACATGTTAACAATGACACTTGACCATTACCAACAAACCAAACTTTTTACAGATGTATTAGAACGTCTTAACCAAATATTTATCGCCATATTTTCAACCGAATGTCTACTTAAAATATTC
 4 MW759894.1_ND_B1A-34   AACATGTTAACAATGACACTTGACCATTACCAACAAACCAAACTTTTTACAGATGTATTAGAACGTCTTAACCAAATATTTATCGCCATATTTTCAACCGAATGTCTACTTAAAATATTC SUS
 5 MW759895.1_X4_B1B-34   AACATGTTAACAATGACACTTGACCATTACCAACAAACCAAACTTTTTACAGATGTATTAGAACGTCTTAACCAAATATTTATCGCCATATTTTCAACCGAATGTCTACTTAAAATATTC SUS
 6 MW759896.1_ND_B3B-34   AACATGTTAACAATGACACTTGACCATTACCAACAAACCAAACTTTTTACAGATGTATTAGAACGTCTTAACCAAATATTTATCGCCATATTTTCAACCGAATGTCTACTTAAAATATTC SUS
 7 MW759897.1_ND_B4B-34   AACATGTTAACAATGACACTTGACCATTACCAACAAACCAAACTTTTTACAGATGTATTAGAACGTCTTAACCAAATATTTATCGCCATATTTTCAACCGAATGTCTACTTAAAATATTC SUS
 8 MW759898.1_X4_MN1-4-34 AACATGTTAACAATGACACTTGACCATTACCAACAAACCAAACTTTTTACAGATGTATTAGAACGTCTTAACCAAATATTTATCGCCATATTTTCAACCGAATGTCTACTTAAAATATTC RES
 9 MW759899.1_X4_MN1-5-34 AACATGTTAACAATGACACTTGACCATTACCAACAAACCAAACTTTTTACAGATGTATTAGAACGTCTTAACCAAATATTTATCGCCATATTTTCAACCGAATGTCTACTTAAAATATTC RES
10 MW759900.1_ND_MN1-7-34 AACATGTTAACAATGACACTTGACCATTACCAACAAACCAAACTTTTTACAGATGTATTAGAACGTCTTAACCAAATATTTATCGCCATATTTTCAACCGAATGTCTACTTAAAATATTC RES
11 MW759901.1_ND_MN2-2-34 AACATGTTAACAATGACACTTGACCATTACCAACAAACCAAACTTTTTACAGATGTATTAGAACGTCTTAACCAAATATTTATCGCCATATTTTCAACCGAATGTCTACTTAAAATATTC RES
12 MW759902.1_ND_MN2-9-34 AACATGTTAACAATGACACTTGACCATTACCAACAAACCAAACTTTTTACAGATGTATTAGAACGTCTTAACCAAATATTTATCGCCATATTTTCAACCGAATGTCTACTTAAAATATTC RES
13 MW759903.1_nd_Suth5-34 AACATGTTAACAATGACACTTGACCATTACCAACAAACCAAACTTTTTACAGATGTATTAGAACGTCTTAACCAAATATTTATCGCCATATTTTCAACCGAATGTCTACTTAAAATATTC RES
14 MW759904.1_X4_Suth6-34 AACATGTTAACAATGACACTTGACCATTACCAACAAACCAAACTTTTTACAGATGTATTAGAACGTCTTAACCAAATATTTATCGCCATATTTTCAACCGAATGTCTACTTAAAATATTC RES

                          ......                  ..................................DIV S3.................................
                            A  L  R  Y  Y  Y  F  K  E  P  W  N  L  F  D  F  V  V  V  I  L  S  L  A  G  L  V  L  S  D  L  I  S  K  Y  F  V  S  P  T   680
                     1921         .        .        :        .        .        .        .        0        .        .        .        . 2040
 1 MT379844.1_X1          GCATTGCGATATTATTATTTTAAAGAACCATGGAACTTATTTGACTTCGTGGTCGTTATACTTTCCTTAGCGGGTTTGGTGTTAAGTGATTTGATATCCAAGTATTTTGTGTCACCAACG
 2 MT379844.1_X2          GCATTGCGATATTATTATTTTAAAGAACCATGGAACTTATTTGACTTCGTGGTCGTTATACTTTCCTTAGCGGGTTTGGTGTTAAGTGATTTGATATCCAAGTATTTTGTGTCACCAACG
 3 MT379844.1_X3          GCATTGCGATATTATTATTTTAAAGAACCATGGAACTTATTTGACTTCGTGGTCGTTATACTTTCCTTAGCGGGTTTGGTGTTAAGTGATTTGATATCCAAGTATTTTGTGTCACCAACG
 4 MW759894.1_ND_B1A-34   GCATTGCGATATTATTATTTTAAAGAACCATGGAACTTATTTGACTTCGTGGTCGTTATACTTTCCTTAGCGGGTTTGGTGTTAAGTGATTTGATATCCAAGTATTTTGTGTCACCAACG SUS
 5 MW759895.1_X4_B1B-34   GCATTGCGATATTATTATTTTAAAGAACCATGGAACTTATTTGACTTCGTGGTCGTTATACTTTCCTTAGCGGGTTTGGTGTTAAGTGATTTGATATCCAAGTATTTTGTGTCACCAACG SUS
 6 MW759896.1_ND_B3B-34   GCATTGCGATATTATTATTTTAAAGAACCATGGAACTTATTTGACTTCGTGGTCGTTATACTTTCCTTAGCGGGTTTGGTGTTAAGTGATTTGATATCCAAGTATTTTGTGTCACCAACG SUS
 7 MW759897.1_ND_B4B-34   GCATTGCGATATTATTATTTTAAAGAACCATGGAACTTATTTGACTTCGTGGTCGTTATACTTTCCTTAGCGGGTTTGGTGTTAAGTGATTTGATATCCAAGTATTTTGTGTCACCAACG SUS
 8 MW759898.1_X4_MN1-4-34 GCATTGCGATATTATTATTTTAAAGAACCATGGAACTTATTTGACTTCGTGGTCGTTATACTTTCCTTAGCGGGTTTGGTGTTAAGTGATTTGATATCCAAGTATTTTGTGTCACCAACG RES
 9 MW759899.1_X4_MN1-5-34 GCATTGCGATATTATTATTTTAAAGAACCATGGAACTTATTTGACTTCGTGGTCGTTATACTTTCCTTAGCGGGTTTGGTGTTAAGTGATTTGATATCCAAGTATTTTGTGTCACCAACG RES
10 MW759900.1_ND_MN1-7-34 GCATTGCGATATTATTATTTTAAAGAACCATGGAACTTATTTGACTTCGTGGTCGTTATACTTTCCTTAGCGGGTTTGGTGTTAAGTGATTTGATATCCAAGTATTTTGTGTCACCAACG RES
11 MW759901.1_ND_MN2-2-34 GCATTGCGATATTATTATTTTAAAGAACCATGGAACTTATTGGACTTCGTGGTCGTTATACTTTCCTTAGCGGGTTTGGTGTTAAGTGATTTGATATCCAAGTATTTTGTGTCACCAACG RES
12 MW759902.1_ND_MN2-9-34 GCATTGCGATATTATTATTTTAAAGAACCATGGAACTTATTGGACTTCGTGGTCGTTATACTTTCCTTAGCGGGTTTGGTGTTAAGTGATTTGATATCCAAGTATTTTGTGTCACCAACG RES
13 MW759903.1_nd_Suth5-34 GCATTGCGATATTATTATTTTAAAGAACCATGGAACTTATTTGACTTCGTGGTCGTTATACTTTCCTTAGCGGGTTTGGTGTTAAGTGATTTGATATCCAAGTATTTTGTGTCACCAACG RES
14 MW759904.1_X4_Suth6-34 GCATTGCGATATTATTATTTTAAAGAACCATGGAACTTATTTGACTTCGTGGTCGTTATACTTTCCTTAGCGGGTTTGGTGTTAAGTGATTTGATATCCAAGTATTTTGTGTCACCAACG RES
```

```
                               ............................DIV S4............................                ...
                               L  L  R  V  V  R  V  A  K  V  G  R  V  L  R  L  V  K  G  A  K  G  I  R  T  L  L  F  A  L  A  M  S  L  P  A  L  F  N  I   720
                         2041           :        .        .        .        .        1        .        .        .        .        :        .  2160
 1 MT379844.1_X1          CTCCTTCGAGTGGTCAGAGTAGCCAAGGTGGGCCGAGTGTTACGTTTGGTAAAAGGAGCGAAAGGGATTAGAACTCTATTGTTCGCTTTAGCCATGTCACTTCCAGCGTTATTCAACATA
 2 MT379844.1_X2          CTCCTTCGAGTGGTCAGAGTAGCCAAGGTGGGCCGAGTGTTACGTTTGGTAAAAGGAGCGAAAGGGATTAGAACTCTATTGTTCGCTTTAGCCATGTCACTTCCAGCGTTATTCAACATA
 3 MT379844.1_X3          CTCCTTCGAGTGGTCAGAGTAGCCAAGGTGGGCCGAGTGTTACGTTTGGTAAAAGGAGCGAAAGGGATTAGAACTCTATTGTTCGCTTTAGCCATGTCACTTCCAGCGTTATTCAACATA
 4 MW759894.1_ND_B1A-34   CTCCTTCGAGTGGTCAGAGTAGCCAAGGTGGGCCGAGTGTTACGTTTGGTAAAAGGAGCGAAAGGGATTAGAACTCTATTGTTCGCTTTAGCCATGTCACTTCCAGCGTTATTCAACATA SUS
 5 MW759895.1_X4_B1B-34   CTCCTTCGAGTGGTCAGAGTAGCCAAGGTGGGCCGAGTGTTACGTTTGGTAAAAGGAGCGAAAGGGATTAGAACTCTATTGTTCGCTTTAGCCATGTCACTTCCAGCGTTATTCAACATA SUS
 6 MW759896.1_ND_B3B-34   CTCCTTCGAGTGGTCAGAGTAGCCAAGGTGGGCCGAGTGTTACGTTTGGTAAAAGGAGCGAAAGGGATTAGAACTCTATTGTTCGCTTTAGCCATGTCACTTCCAGCGTTATTCAACATA SUS
 7 MW759897.1_ND_B4B-34   CTCCTTCGAGTGGTCAGAGTAGCCAAGGTGGGCCGAGTGTTACGTTTGGTAAAAGGAGCGAAAGGGATTAGAACTCTATTGTTCGCTTTAGCCATGTCACTTCCAGCGTTATTCAACATA SUS
 8 MW759898.1_X4_MN1-4-34 CTCCTTCGAGTGGTCAGAGTAGCCAAGGTGGGCCGAGTGTTACGTTTGGTAAAAGGAGCGAAAGGGATTAGAACTCTATTGTTCGCTTTAGCCATGTCACTTCCAGCGTTATTCAACATA RES
 9 MW759899.1_X4_MN1-5-34 CTCCTTCGAGTGGTCAGAGTAGCCAAGGTGGGCCGAGTGTTACGTTTGGTAAAAGGAGCGAAAGGGATTAGAACTCTATTGTTCGCTTTAGCCATGTCACTTCCAGCGTTATTCAACATA RES
10 MW759900.1_ND_MN1-7-34 CTCCTTCGAGTGGTCAGAGTAGCCAAGGTGGGCCGAGTGTTACGTTTGGTAAAAGGAGCGAAAGGGATTAGAACTCTATTGTTCGCTTTAGCCATGTCACTTCCAGCGTTATTCAACATA RES
11 MW759901.1_ND_MN2-2-34 CTCCTTCGAGTGGTCAGAGTAGCCAAGGTGGGCCGAGTGTTACGTTTGGTAAAAGGAGCGAAAGGGATTAGAACTCTATTGTTCGCTTTAGCCATGTCACTTCCAGCGTTATTCAACATA RES
12 MW759902.1_ND_MN2-9-34 CTCCTTCGAGTGGTCAGAGTAGCCAAGGTGGGCCGAGTGTTACGTTTGGTAAAAGGAGCGAAAGGGATTAGAACTCTATTGTTCGCTTTAGCCATGTCACTTCCAGCGTTATTCAACATA RES
13 MW759903.1_nd_Suth5-34 CTCCTTCGAGTGGTCAGAGTAGCCAAGGTGGGCCGAGTGTTACGTTTGGTAAAAGGAGCGAAAGGGATTAGAACTCTATTGTTCGCTTTAGCCATGTCACTTCCAGCGTTATTCAACATA RES
14 MW759904.1_X4_Suth6-34 CTCCTTCGAGTGGTCAGAGTAGCCAAGGTGGGCCGAGTGTTACGTTTGGTAAAAGGAGCGAAAGGGATTAGAACTCTATTGTTCGCTTTAGCCATGTCACTTCCAGCGTTATTCAACATA RES

                          ............................DIV S5............................
                          C  L  L  L  F  L  V  M  F  I  F  A  I  F  G  M  S  F  F  M  N  V  D  N  H  G  G  L  D  E  D  Y  N  F  R  T  F  G  Q  S   760
                         2161           .        .        .        2        .        .        .        .        :        .        .        .  2280
 1 MT379844.1_X1          TGTTTGTTACTGTTTCTTGTCATGTTCATATTTGCTATATTTGGCATGTCGTTTTTCATGAACGTAGATAATCATGGAGGTTTGGATGAGGATTACAATTTCCGCACTTTCGGGCAATCG
 2 MT379844.1_X2          TGTTTGTTACTGTTTCTTGTCATGTTCATATTTGCTATATTTGGCATGTCGTTTTTCATGAACGTAGATAATCATGGAGGTTTGGATGAGGATTACAATTTCCGCACTTTCGGGCAATCG
 3 MT379844.1_X3          TGTTTGTTACTGTTTCTTGTCATGTTCATATTTGCTATATTTGGCATGTCGTTTTTCATGAACGTAGATAATCATGGAGGTTTGGATGAGGATTACAATTTCCGCACTTTCGGGCAATCG
 4 MW759894.1_ND_B1A-34   TGTTTGTTACTGTTTCTTGTCATGTTCATATTTGCTATATTTGGCATGTCGTTTTTCATGAACGTAGATAATCATGGAGGTTTGGATGAGGATTACAATTTCCGCACTTTCGGGCAATCG SUS
 5 MW759895.1_X4_B1B-34   TGTTTGTTACTGTTTCTTGTCATGTTCATATTTGCTATATTTGGCATGTCGTTTTTCATGAACGTAGATAATCATGGAGGTTTGGATGAGGATTACAATTTCCGCACTTTCGGGCAATCG SUS
 6 MW759896.1_ND_B3B-34   TGTTTGTTACTGTTTCTTGTCATGTTCATATTTGCTATATTTGGCATGTCGTTTTTCATGAACGTAGATAATCATGGAGGTTTGGATGAGGATTACAATTTCCGCACTTTCGGGCAATCG SUS
 7 MW759897.1_ND_B4B-34   TGTTTGTTACTGTTTCTTGTCATGTTCATATTTGCTATATTTGGCATGTCGTTTTTCATGAACGTAGATAATCATGGAGGTTTGGATGAGGATTACAATTTCCGCACTTTCGGGCAATCG SUS
 8 MW759898.1_X4_MN1-4-34 TGTTTGTTACTGTTTCTTGTCATGTTCATATTTGCTATATTTGGCATGTCGTTTTTCATGAACGTAGATAATCATGGAGGTTTGGATGAGGATTACAATTTCCGCACTTTCGGGCAATCG RES
 9 MW759899.1_X4_MN1-5-34 TGTTTGTTACTGTTTCTTGTCATGTTCATATTTGCTATATTTGGCATGTCGTTTTTCATGAACGTAGATAATCATGGAGGTTTGGATGAGGATTACAATTTCCGCACTTTCGGGCAATCG RES
10 MW759900.1_ND_MN1-7-34 TGTTTGTTACTGTTTCTTGTCATGTTCATATTTGCTATATTTGGCATGTCGTTTTTCATGAACGTAGATAATCATGGAGGTTTGGATGAGGATTACAATTTCCGCACTTTCGGGCAATCG RES
11 MW759901.1_ND_MN2-2-34 TGTTTGTTACTGTTTCTTGTCATGTTCATATTTGCTATATTTGGCATGTCGTTTTTCATGAACGTAGATAATCATGGAGGTTTGGATGAGGATTACAATTTCCGCACTTTCGGGCAATCG RES
12 MW759902.1_ND_MN2-9-34 TGTTTGTTACTGTTTCTTGTCATGTTCATATTTGCTATATTTGGCATGTCGTTTTTCATGAACGTAGATAATCATGGAGGTTTGGATGAGGATTACAATTTCCGCACTTTCGGGCAATCG RES
13 MW759903.1_nd_Suth5-34 TGTTTGTTACTGTTTCTTGTCATGTTCATATTTGCTATATTTGGCATGTCGTTTTTCATGAACGTAGATAATCATGGAGGTTTGGATGAGGATTACAATTTCCGCACTTTCGGGCAATCG RES
14 MW759904.1_X4_Suth6-34 TGTTTGTTACTGTTTCTTGTCATGTTCATATTTGCTATATTTGGCATGTCGTTTTTCATGAACGTAGATAATCATGGAGGTTTGGATGAGGATTACAATTTCCGCACTTTCGGGCAATCG RES

                          M  I  L  L  F  M  L  S  T  S  S  G  W  D  A  V  L  D  G  I  T  N  E  D  D  C  D  K  P  N  L  E  M  G  I  T  G  S  C  G   800
                         2281           .        3        .        .        .        .        :        .        .        .        .        4  2400
 1 MT379844.1_X1          ATGATACTTCTTTTCATGCTTTCTACGTCATCCGGTTGGGACGCCGTACTGGACGGTATAACGAACGAGGACGATTGTGATAAGCCAAATTTAGAAATGGGTATCACGGGAAGTTGTGGA
 2 MT379844.1_X2          ATGATACTTCTTTTCATGCTTTCTACGTCATCCGGTTGGGACGCCGTACTGGACGGTATAACGAACGAGGACGATTGTGATAAGCCAAATTTAGAAATGGGTATCACGGGAAGTTGTGGA
 3 MT379844.1_X3          ATGATACTTCTTTTCATGCTTTCTACGTCATCCGGTTGGGACGCCGTACTGGACGGTATAACGAACGAGGACGATTGTGATAAGCCAAATTTAGAAATGGGTATCACGGGAAGTTGTGGA
 4 MW759894.1_ND_B1A-34   ATGATACTTCTTTTCATGCTTTCTACGTCATCCGGTTGGGACGCCGTACTGGACGGTATAACGAACGAGGACGATTGTGATAAGCCAAATTTAGAAATGGGTATCACGGGAAGTTGTGGA SUS
 5 MW759895.1_X4_B1B-34   ATGATACTTCTTTTCATGCTTTCTACGTCATCCGGTTGGGACGCCGTACTAGACGGTATAACGAACGAGGACGATTGTGATAAGCCAAATTTAGAAATGGGTATCACGGGAAGTTGTGGA SUS
 6 MW759896.1_ND_B3B-34   ATGATACTTCTTTTCATGCTTTCTACGTCATCCGGTTGGGACGCCGTACTAGACGGTATAACGAACGAGGACGATTGTGATAAGCCAAATTTAGAAATGGGTATCACGGGAAGTTGTGGA SUS
 7 MW759897.1_ND_B4B-34   ATGATACTTCTTTTCATGCTTTCTACGTCATCCGGTTGGGACGCCGTACTAGACGGTATAACGAACGAGGACGATTGTGATAAGCCAAATTTAGAAATGGGTATCACGGGAAGTTGTGGA SUS
 8 MW759898.1_X4_MN1-4-34 ATGATACTTCTTTTCATGCTTTCTACGTCATCCGGTTGGGACGCCGTACTAGACGGTATAACGAACGAGGACGATTGTGATAAGCCAAATTTAGAAATGGGTATCACGGGAAGTTGTGGA RES
 9 MW759899.1_X4_MN1-5-34 ATGATACTTCTTTTCATGCTTTCTACGTCATCCGGTTGGGACGCCGTACTAGACGGTATAACGAACGAGGACGATTGTGATAAGCCAAATTTAGAAATGGGTATCACGGGAAGTTGTGGA RES
10 MW759900.1_ND_MN1-7-34 ATGATACTTCTTTTCATGCTTTCTACGTCATCCGGTTGGGACGCCGTACTAGACGGTATAACGAACGAGGACGATTGTGATAAGCCAAATTTAGAAATGGGTATCACGGGAAGTTGTGGA RES
11 MW759901.1_ND_MN2-2-34 ATGATACTTCTTTTCATGCTTTCTACGTCATCCGGTTGGGACGCCGTACTAGACGGTATAACGAACGAGGACGATTGTGATAAGCCAAATTTAGAAATGGGTATCACGGGAAGTTGTGGA RES
12 MW759902.1_ND_MN2-9-34 ATGATACTTCTTTTCATGCTTTCTACGTCATCCGGTTGGGACGCCGTACTAGACGGTATAACGAACGAGGACGATTGTGATAAGCCAAATTTAGAAATGGGTATCACGGGAAGTTGTGGA RES
13 MW759903.1_nd_Suth5-34 ATGATACTTCTTTTCATGCTTTCTACGTCATCCGGTTGGGACGCCGTACTAGACGGTATAACGAACGAGGACGATTGTGATAAGCCAAATTTAGAAATGGGTATCACGGGAAGTTGTGGA RES
14 MW759904.1_X4_Suth6-34 ATGATACTTCTTTTCATGCTTTCTACGTCATCCGGTTGGGACGCCGTACTAGACGGTATAACGAACGAGGACGATTGTGATAAGCCAAATTTAGAAATGGGTATCACGGGAAGTTGTGGA RES
```

```
                                           ..............................DIV S6...............................
                                           S  S  A  V  G  T  A  F  L  L  S  Y  L  V  I  N  F  L  I  V  I  N  M  Y  I  A  V  I  L  E  N  Y  S  Q  A  T  E  D  V  Q   840
                          2401            .         .         .         .         :         .         .         .         .         5         .         . 2520
 1 MT379844.1_X1           AGTAGTGCCGTGGGCACGGCATTTTTACTCTCGTACCTGGTGATCAATTTCCTCATCGTCATAAACATGTACATCGCAGTCATTCTGGAGAACTATTCACAGGCTACAGAGGACGTTCAG
 2 MT379844.1_X2           AGTAGTGCCGTGGGCACGGCATTTTTACTCTCGTACCTGGTGATCAATTTCCTCATCGTCATAAACATGTACATCGCAGTCATTCTGGAGAACTATTCACAGGCTACAGAGGACGTTCAG
 3 MT379844.1_X3           AGTAGTGCCGTGGGCACGGCATTTTTACTCTCGTACCTGGTGATCAATTTCCTCATCGTCATAAACATGTACATCGCAGTCATTCTGGAGAACTATTCACAGGCTACAGAGGACGTTCAG
 4 MW759894.1_ND_B1A-34    AGTAGTGCCGTGGGCACGGCATTTTTACTCTCGTACCTGGTGATCAATTTCCTCATCGTCATAAACATGTACATCGCAGTCATTCTGGAAAACTATTCACAGGCTACAGAGGACGTTCAG SUS
 5 MW759895.1_X4_B1B-34    AGTAGTGCCGTGGGCACGGCATTTTTACTCTCGTACCTGGTGATCAATTTCCTCATCGTCATAAACATGTACATCGCAGTCATTCTGGAGAACTATTCACAGGCTACAGAGGACGTTCAG SUS
 6 MW759896.1_ND_B3B-34    AGTAGTGCCGTGGGCACGGCATTTTTACTCTCGTACCTGGTGATCAATTTCCTCATCGTCATAAACATGTACATCGCAGTCATTCTGGAGAACTATTCACAGGCTACAGAGGACGTTCAG SUS
 7 MW759897.1_ND_B4B-34    AGTAGTGCCGTGGGCACGGCATTTTTACTCTCGTACCTGGTGATCAATTTCCTCATCGTCATAAACATGTACATCGCAGTCATTCTGGAGAACTATTCACAGGCTACAGAGGACGTTCAG SUS
 8 MW759898.1_X4_MN1-4-34  AGTAGTGCCGTGGGCACGGCATTTTTACTCTCGTACCTGGTGATCAATTTCCTCATCGTCATAAACATGTACATCGCAGTCATTCTGGAGAACTATTCACAGGCTACAGAGGACGTTCAG RES
 9 MW759899.1_X4_MN1-5-34  AGTAGTGCCGTGGGCACGGCATTTTTACTCTCGTACCTGGTGATCAATTTCCTCATCGTCATAAACATGTACATCGCAGTCATTCTGGAGAACTATTCACAGGCTACAGAGGACGTTCAG RES
10 MW759900.1_ND_MN1-7-34  AGTAGTGCCGTGGGCACGGCATTTTTACTCTCGTACCTGGTGATCAATTTCCTCATCGTCATAAACATGTACATCGCAGTCATTCTGGAGAACTATTCACAGGCTACAGAGGACGTTCAG RES
11 MW759901.1_ND_MN2-2-34  AGTAGTGCCGTGGGCACGGCATTTTTACTCTCGTACCTGGTGATCAATTTCCTCATCGTCATAAACATGTACATCGCAGTCATTCTGGAGAACTATTCACAGGCTACAGAGGACGTTCAG RES
12 MW759902.1_ND_MN2-9-34  AGTAGTGCCGTGGGCACGGCATTTTTACTCTCGTACCTGGTGATCAATTTCCTCATCGTCATAAACATGTACATCGCAGTCATTCTGGAGAACTATTCACAGGCTACAGAGGACGTTCAG RES
13 MW759903.1_nd_Suth5-34  AGTAGTGCCGTGGGCACGGCATTTTTACTCTCGTACCTGGTGATCAATTTCCTCATCGTCATAAACATGTACATCGCAGTCATTCTGGAGAACTATTCACAGGCTACAGAGGACGTTCAG RES
14 MW759904.1_X4_Suth6-34  AGTAGTGCCGTGGGCACGGCATTTTTACTCTCGTACCTGGTGATCAATTTCCTCATCGTCATAAACATGTACATCGCAGTCATTCTGGAGAACTATTCACAGGCTACAGAGGACGTTCAG RES

                           E  G  L  T  D  D  D  Y  D  M  Y  Y  E  I  W  Q  H  F  D  P  N  G  T  R  Y  I  R  Y  D  Q  L  S  D  F  L  D  I  L  E  P   880
                          2521            .         .         :         .         .         .         .         6         .         .         .         . 2640
 1 MT379844.1_X1           GAAGGTCTTACGGACGACGATTACGACATGTATTACGAGATATGGCAGCATTTCGACCCCAACGGGACCAGGTATATTCGATACGACCAACTGTCAGACTTCCTAGACATTCTAGAACCG
 2 MT379844.1_X2           GAAGGTCTTACGGACGACGATTACGACATGTATTACGAGATATGGCAGCATTTCGACCCCAACGGGACCAGGTATATTCGATACGACCAACTGTCAGACTTCCTAGACATTCTAGAACCG
 3 MT379844.1_X3           GAAGGTCTTACGGACGACGATTACGACATGTATTACGAGATATGGCAGCATTTCGACCCCAACGGGACCAGGTATATTCGATACGACCAACTGTCAGACTTCCTAGACATTCTAGAACCG
 4 MW759894.1_ND_B1A-34    GAAGGTCTTACGGACGACGATTACGACATGTATTACGAAATATGGCAGCATTTCGACCCCAACGGGACCAGGTATATTCGATACGACCAACTGTCAGACTTCCTAGACATTCTAGAACCG SUS
 5 MW759895.1_X4_B1B-34    GAAGGTCTTACGGACGACGATTACGACATGTATTACGAGATATGGCAGCATTTCGACCCCAACGGGACCAGGTATATTCGATACGACCAACTGTCAGACTTCCTAGACATTCTAGAACCG SUS
 6 MW759896.1_ND_B3B-34    GAAGGTCTTACGGACGACGATTACGACATGTATTACGAGATATGGCAGCATTTCGACCCCAACGGGACCAGGTATATTCGATACGACCAACTGTCAGACTTCCTAGACATTCTAGAACCG SUS
 7 MW759897.1_ND_B4B-34    GAAGGTCTTACGGACGACGATTACGACATGTATTACGAGATATGGCAGCATTTCGACCCCAACGGGACCAGGTATATTCGATACGACCAACTGTCAGACTTCCTAGACATTCTAGAACCG SUS
 8 MW759898.1_X4_MN1-4-34  GAAGGTCTTACGGACGACGATTACGACATGTATTACGAGATATGGCAGCATTTCGACCCCAACGGGACCAGGTATATTCGATACGACCAACTGTCAGACTTCCTAGACATTCTAGAACCG RES
 9 MW759899.1_X4_MN1-5-34  GAAGGTCTTACGGACGACGATTACGACATGTATTACGAGATATGGCAGCATTTCGACCCCAACGGGACCAGGTATATTCGATACGACCAACTGTCAGACTTCCTAGACATTCTAGAACCG RES
10 MW759900.1_ND_MN1-7-34  GAAGGTCTTACGGACGACGATTACGACATGTATTACGAGATATGGCAGCATTTCGACCCCAACGGGACCAGGTATATTCGATACGACCAACTGTCAGACTTCCTAGACATTCTAGAACCG RES
11 MW759901.1_ND_MN2-2-34  GAAGGTCTTACGGACGACGATTACGACATGTATTACGAGATATGGCAGCATTTCGACCCCAACGGGACCAGGTATATTCGATACGACCAACTGTCAGACTTCCTAGACATTCTAGAACCG RES
12 MW759902.1_ND_MN2-9-34  GAAGGTCTTACGGACGACGATTACGACATGTATTACGAGATATGGCAGCATTTCGACCCCAACGGGACCAGGTATATTCGATACGACCAACTGTCAGACTTCCTAGACATTCTAGAACCG RES
13 MW759903.1_nd_Suth5-34  GAAGGTCTTACGGACGACGATTACGACATGTATTACGAGATATGGCAGCATTTCGACCCCAACGGGACCAGGTATATTCGATACGACCAACTGTCAGACTTCCTAGACATTCTAGAACCG RES
14 MW759904.1_X4_Suth6-34  GAAGGTCTTACGGACGACGATTACGACATGTATTACGAGATATGGCAGCATTTCGACCCCAACGGGACCAGGTATATTCGATACGACCAACTGTCAGACTTCCTAGACATTCTAGAACCG RES

                           P  L  K  I  H  K  P  N  K  Y  K  I  V  S  M  D  I  P  I  C  K  G  D  L  I  Y  C  V  D  I  L  D  A  L  T  K  D  F  F  A   920
                          2641            :         .         .         .         .         7         .         .         .         .         :         . 2760
 1 MT379844.1_X1           CCGTTGAAGATCCACAAGCCGAACAAGTACAAGATCGTGTCTATGGATATACCGATTTGCAAAGGCGACCTAATCTACTGTGTAGATATACTGGACGCGTTGACCAAGGACTTCTTCGCG
 2 MT379844.1_X2           CCGTTGAAGATCCACAAGCCGAACAAGTACAAGATCGTGTCTATGGATATACCGATTTGCAAAGGCGACCTAATCTACTGTGTAGATATACTGGACGCGTTGACCAAGGACTTCTTCGCG
 3 MT379844.1_X3           CCGTTGAAGATCCACAAGCCGAACAAGTACAAGATCGTGTCTATGGATATACCGATTTGCAAAGGCGACCTAATCTACTGTGTAGATATACTGGACGCGTTGACCAAGGACTTCTTCGCG
 4 MW759894.1_ND_B1A-34    CCGTTGAAGATCCACAAGCCGAACAAGTACAAGATCGTGTCTATGGATATACCGATTTGCAAAGGCGACCTAATCTACTGTGTAGATATACTGGACGCGTTGACCAAGGACTTCTTCGCG SUS
 5 MW759895.1_X4_B1B-34    CCGTTGAAGATCCACAAGCCGAACAAGTACAAGATCGTGTCTATGGATATACCGATTTGCAAAGGCGACCTAATCTACTGTGTAGATATACTGGACGCGTTGACCAAGGACTTCTTCGCG SUS
 6 MW759896.1_ND_B3B-34    CCGTTGAAGATCCACAAGCCGAACAAGTACAAGATCGTGTCTATGGATATACCGATTTGCAAAGGCGACCTAATCTACTGTGTAGATATACTGGACGCGTTGACCAAGGACTTCTTCGCG SUS
 7 MW759897.1_ND_B4B-34    CCGTTGAAGATCCACAAGCCGAACAAGTACAAGATCGTGTCTATGGATATACCGATTTGCAAAGGCGACCTAATCTACTGTGTAGATATACTGGACGCGTTGACCAAGGACTTCTTCGCG SUS
 8 MW759898.1_X4_MN1-4-34  CCGTTGAAGATCCACAAGCCGAACAAGTACAAGATCGTGTCTATGGATATACCGATTTGCAAAGGCGACCTAATCTACTGTGTAGATATACTGGACGCGTTGACCAAGGACTTCTTCGCG RES
 9 MW759899.1_X4_MN1-5-34  CCGTTGAAGATCCACAAGCCGAACAAGTACAAGATCGTGTCTATGGATATACCGATTTGCAAAGGCGACCTAATCTACTGTGTAGATATACTGGACGCGTTGACCAAGGACTTCTTCGCG RES
10 MW759900.1_ND_MN1-7-34  CCGTTGAAGATCCACAAGCCGAACAAGTACAAGATCGTGTCTATGGATATACCGATTTGCAAAGGCGACCTAATCTACTGTGTAGATATACTGGACGCGTTGACCAAGGACTTCTTCGCG RES
11 MW759901.1_ND_MN2-2-34  CCGTTGAAGATCCACAAGCCGAACAAGTACAAGATCGTGTCTATGGATATACCGATTTGCAAAGGCGACCTAATCTACTGTGTAGATATACTGGACGCGTTGACCAAGGACTTCTTCGCG RES
12 MW759902.1_ND_MN2-9-34  CCGTTGAAGATCCACAAGCCGAACAAGTACAAGATCGTGTCTATGGATATACCGATTTGCAAAGGCGACCTAATCTACTGTGTAGATATACTGGACGCGTTGACCAAGGACTTCTTCGCG RES
13 MW759903.1_nd_Suth5-34  CCGTTGAAGATCCACAAGCCGAACAAGTACAAGATCGTGTCTATGGATATACCGATTTGCAAAGGCGACCTAATCTACTGTGTAGATATACTGGACGCGTTGACCAAGGACTTCTTCGCG RES
14 MW759904.1_X4_Suth6-34  CCGTTGAAGATCCACAAGCCGAACAAGTACAAGATCGTGTCTATGGATATACCGATTTGCAAAGGCGACCTAATCTACTGTGTAGATATACTGGACGCGTTGACCAAGGACTTCTTCGCG RES
```

```
                             R  K  G  N  P  I  I  E  T  V  A  E  I  G  E  M  Q  T  R  P  E  E  A  G  Y  E  P  I  S  S  S  L  W  R  M  R  E  V  Y  C   960
                        2761          .         .         .         8         .         .         .         .         :         .         .         . 2880
 1 MT379844.1_X1          AGAAAGGGCAATCCTATCATCGAGACCGTGGCCGAGATCGGAGAGATGCAAACGCGACCGGAAGAAGCTGGATACGAGCCCATAAGCTCATCTCTGTGGCGTATGCGTGAGGTTTATTGC
 2 MT379844.1_X2          AGAAAGGGCAATCCTATCATCGAGACCGTGGCCGAGATCGGAGAGATGCAAACGCGACCGGAAGAAGCTGGATACGAGCCCATAAGCTCATCTCTGTGGCGTATGCGTGAGGTTTATTGC
 3 MT379844.1_X3          AGAAAGGGCAATCCTATCATCGAGACCGTGGCCGAGATCGGAGAGATGCAAACGCGACCGGAAGAAGCTGGATACGAGCCCATAAGCTCATCTCTGTGGCGTATGCGTGAGGTTTATTGC
 4 MW759894.1_ND_B1A-34   AGAAAGGGCAATCCTATCATCGAGACCGTGGCCGAGATCGGAGAGATGCAAACGCGACCGGAAGAAGCTGGATACGAGCCCATAAGCTCATCTCTGTGGCGTATGCGTGAGGTTTATTGC SUS
 5 MW759895.1_X4_B1B-34   AGAAAGGGCAATCCTATCATCGAGACCGTGGCCGAGATCGGAGAGATGCAAACGCGACCGGAAGAAGCTGGATACGAGCCCATAAGCTCATCTCTGTGGCGTATGCGTGAGGTTTATTGC SUS
 6 MW759896.1_ND_B3B-34   AGAAAGGGCAATCCTATCATCGAGACCGTGGCCGAGATCGGAGAGATGCAAACGCGACCGGAAGAAGCTGGATACGAGCCCATAAGCTCATCTCTGTGGCGTATGCGTGAGGTTTATTGC SUS
 7 MW759897.1_ND_B4B-34   AGAAAGGGCAATCCTATCATCGAGACCGTGGCCGAGATCGGAGAGATGCAAACGCGACCGGAAGAAGCTGGATACGAGCCCATAAGCTCATCTCTGTGGCGTATGCGTGAGGTTTATTGC SUS
 8 MW759898.1_X4_MN1-4-34 AGAAAGGGCAATCCTATCATCGAGACCGTGGCCGAGATCGGAGAGATGCAAACGCGACCGGAAGAAGCTGGATACGAGCCCATAAGCTCATCTCTGTGGCGTATGCGTGAGGTTTATTGC RES
 9 MW759899.1_X4_MN1-5-34 AGAAAGGGCAATCCTATCATCGAGACCGTGGCCGAGATCGGAGAGATGCAAACGCGACCGGAAGAAGCTGGATACGAGCCCATAAGCTCATCTCTGTGGCGTATGCGTGAGGTTTATTGC RES
10 MW759900.1_ND_MN1-7-34 AGAAAGGGCAATCCTATCATCGAGACCGTGGCCGAGATCGGAGAGATGCAAACGCGACCGGAAGAAGCTGGATACGAGCCCATAAGCTCATCTCTGTGGCGTATGCGTGAGGTTTATTGC RES
11 MW759901.1_ND_MN2-2-34 AGAAAGGGCAATCCTATCATCGAGACCGTGGCCGAGATCGGAGAGATGCAAACGCGACCGGAAGAAGCTGGATACGAGCCCATAAGCTCATCTCTGTGGCGTATGCGTGAGGTTTATTGC RES
12 MW759902.1_ND_MN2-9-34 AGAAAGGGCAATCCTATCATCGAGACCGTGGCCGAGATCGGAGAGATGCAAACGCGACCGGAAGAAGCTGGATACGAGCCCATAAGCTCATCTCTGTGGCGTATGCGTGAGGTTTATTGC RES
13 MW759903.1_nd_Suth5-34 AGAAAGGGCAATCCTATCATCGAGACCGTGGCCGAGATCGGAGAGATGCAAACGCGACCGGAAGAAGCTGGATACGAGCCCATAAGCTCATCTCTGTGGCGTATGCGTGAGGTTTATTGC RES
14 MW759904.1_X4_Suth6-34 AGAAAGGGCAATCCTATCATCGAGACCGTGGCCGAGATCGGAGAGATGCAAACGCGACCGGAAGAAGCTGGATACGAGCCCATAAGCTCATCTCTGTGGCGTATGCGTGAGGTTTATTGC RES

                             A  T  I  I  Q  S  S  W  R  K  Y  A  A  A  A  K  Q  Q  T  A  D  D  D  R  S  D  G  A  A  S  P  D  G  R  E  T  A  V  L  V  1000
                        2881          .         9         .         .         .         .         :         .         .         .         .         0 3000
 1 MT379844.1_X1          GCAACAATCATACAGAGCTCATGGCGTAAGTACGCAGCGGCCGCCAAACAGCAGACCGCAGACGACGACCGGAGCGATGGAGCTGCTTCGCCAGACGGTCGTGAAACCGCCGTGTTGGTC
 2 MT379844.1_X2          GCAACAATCATACAGAGCTCATGGCGTAAGTACGCAGCGGCCGCCAAACAGCAGACCGCAGACGACGACCGGAGCGATGGAGCTGCTTCGCCAGACGGTCGTGAAACCGCCGTGTTGGTC
 3 MT379844.1_X3          GCAACAATCATACAGAGCTCATGGCGTAAGTACGCAGCGGCCGCCAAACAGCAGACCGCAGACGACGACCGGAGCGATGGAGCTGCTTCGCCAGACGGTCGTGAAACCGCCGTGTTGGTC
 4 MW759894.1_ND_B1A-34   GCAACAATCATACAGAGCTCATGGCGTAAGTACGCAGCGGCCGCCAAACAGCAGACCGCAGACGACGACCGGAGCGATGGAGCTGCTTCGCCAGACGGTCGTGAAACCGCCGTGTTGGTC SUS
 5 MW759895.1_X4_B1B-34   GCAACAATCATACAGAGCTCATGGCGTAAGTACGCAGCGGCCGCCAAACAGCAGACCGCAGACGACGACCGGAGCGATGGAGCTGCTTCGCCAGACGGTCGTGAAACCGCCGTGTTGGTC SUS
 6 MW759896.1_ND_B3B-34   GCAACAATCATACAGAGCTCATGGCGTAAGTACGCAGCGGCCGCCAAACAGCAGACCGCAGACGACGACCGGAGCGATGGAGCTGCTTCGCCAGACGGTCGTGAAACCGCCGTGTTGGTC SUS
 7 MW759897.1_ND_B4B-34   GCAACAATCATACAGAGCTCATGGCGTAAGTACGCAGCGGCCGCCAAACAGCAGACCGCAGACGACGACCGGAGCGATGGAGCTGCTTCGCCAGACGGTCGTGAAACCGCCGTGTTGGTC SUS
 8 MW759898.1_X4_MN1-4-34 GCAACAATCATACAGAGCTCATGGCGTAAGTACGCAGCGGCCGCCAAACAGCAGACCGCAGACGACGACCGGAGCGATGGAGCTGCTTCGCCAGACGGTCGTGAAACCGCCGTGTTGGTC RES
 9 MW759899.1_X4_MN1-5-34 GCAACAATCATACAGAGCTCATGGCGTAAGTACGCAGCGGCCGCCAAACAGCAGACCGCAGACGACGACCGGAGCGATGGAGCTGCTTCGCCAGACGGTCGTGAAACCGCCGTGTTGGTC RES
10 MW759900.1_ND_MN1-7-34 GCAACAATCATACAGAGCTCATGGCGTAAGTACGCAGCGGCCGCCAAACAGCAGACCGCAGACGACGACCGGAGCGATGGAGCTGCTTCGCCAGACGGTCGTGAAACCGCCGTGTTGGTC RES
11 MW759901.1_ND_MN2-2-34 GCAACAATCATACAGAGCTCATGGCGTAAGTACGCAGCGGCCGCCAAACAGCAGACCGCAGACGACGACCGGAGCGATGGAGCTGCTTCGCCAGACGGTCGTGAAACCGCCGTGTTGGTC RES
12 MW759902.1_ND_MN2-9-34 GCAACAATCATACAGAGCTCATGGCGTAAGTACGCAGCGGCCGCCAAACAGCAGACCGCAGACGACGACCGGAGCGATGGAGCTGCTTCGCCAGACGGTCGTGAAACCGCCGTGTTGGTC RES
13 MW759903.1_nd_Suth5-34 GCAACAATCATACAGAGCTCATGGCGTAAGTACGCAGCGGCCGCCAAACAGCAGACCGCAGACGACGACCGGAGCGATGGAGCTGCTTCGCCAGACGGTCGTGAAACCGCCGTGTTGGTC RES
14 MW759904.1_X4_Suth6-34 GCAACAATCATACAGAGCTCATGGCGTAAGTACGCAGCGGCCGCCAAACAGCAGACCGCAGACGACGACCGGAGCGATGGAGCTGCTTCGCCAGACGGTCGTGAAACCGCCGTGTTGGTC RES

                             E  S  D  G  F  V  T  K  N  G  H  K  V  V  I  H  S  R  S  S  S  K  S  S  R  L  A  D  V] 1029
                        3001          .         .         .         .         :         .         .         .  ] 3090
 1 MT379844.1_X1          GAAAGCGATGGATTTGTTACAAAAAACGGTCACAAGGTAGTCATTCATTCGAGGTCGTCGTCGAAAAGCTCAAGACTCGCCGATGTCTAA
 2 MT379844.1_X2          GAAAGCGATGGATTTGTTACAAAAAACGGTCACAAGGTAGTCATTCATTCGAGGTCGTCGTCGAAAAGCTCAAGACTCGCCGATGTCTAA
 3 MT379844.1_X3          GAAAGCGATGGATTTGTTACAAAAAACGGTCACAAGGTAGTCATTCATTCGAGGTCGTCGTCGAAAAGCTCAAGACTCGCCGATGTCTAA
 4 MW759894.1_ND_B1A-34   GAAAGCGAT--------------------------------------------------------------------------------- SUS
 5 MW759895.1_X4_B1B-34   GAAAGCGAGGGATTTGTTACAAAAAACGGTCACAAGGTAGTCATTCATTCGAGGTCGTCGTCGAAAAGCTCAAGACTCGCCGATGTCTAA SUS
 6 MW759896.1_ND_B3B-34   GAAAGCGATGGATTTGTTACAAAAAACGGTCACAAGGTAGTCATTCATTCGAGGTCGTCGTCGAAAAGCTCAAGACTCGCCGATGTCTAA SUS
 7 MW759897.1_ND_B4B-34   GAAAGC------------------------------------------------------------------------------------ SUS
 8 MW759898.1_X4_MN1-4-34 GAAAGCGATGGA------------------------------------------------------------------------------ RES
 9 MW759899.1_X4_MN1-5-34 GAAAGCGATGGATTTGTTACAAAAAACGGTCACAAGGTAGTCATTCATTCGAGGTCGTCGTCGAAAAGCTCAAGACTCGCCGATGTCTAA RES
10 MW759900.1_ND_MN1-7-34 GAAAGCGATGGATTTGTTACAAAAAACGGTCACAAGGTAGTCATTCATTCGAGGTCGTCGTCGAAAAGCTCAAGACTCGCCGATGTCTAA RES
11 MW759901.1_ND_MN2-2-34 GAAAGCGATGGATTTGTTA----------------------------------------------------------------------- RES
12 MW759902.1_ND_MN2-9-34 GAAAGCGAT--------------------------------------------------------------------------------- RES
13 MW759903.1_nd_Suth5-34 GAAAGCGATGGA------------------------------------------------------------------------------ RES
14 MW759904.1_X4_Suth6-34 GAAAGCGATGGATTTGTTA----------------------------------------------------------------------- RES
```

Figure S6. Multiple amino acid (aa) sequence alignment among *para*-type voltage gated sodium channel heterodimer 2 (VGSC-H2) isoforms among species of aphid (Family Aphididae). The 1031 aa consensus alignment was constructed among translations for isoforms (X1 to X6) among *Aphis glycines* (Aglycines), *Acyrthosiphon pisum* (Apisum), *Aphis gossypii* (Agossypii), *Melanaphis sacchari* (Msacchari), *Rhopalosiphum maidis* (Rmaidis), *Myzus persicae* (Mpersicae), *Diuraphis noxia* (Dnoxia), and *Sipha flava* (Sflava), appended to RefSeq accessions where available. The *A. glycines* sequences QTJ01841.1–QTJ01843.1 represent isoforms X1–X3 (Paula et al. 2021), and isoform X4 from MW759899.1 is representative of cDNA sequences in this study (GenBank accessions MW759894.1–MW759904.1). Putative structural annotation of domain III and IV (DIII and DIV) each comprised of six transmembrane α-helical segments (S1-6) are highlighted grey, and based on orthology between the *A. glycines* cDNA consensus AgVGSC-H2 and the *Musca domestica* VGSC (GenBank accession AAB47604.1; Williamson et al. 1996; **Figure S1**).

```
                              1 [         .         .         .         .         :         .         .         .         .         1 100
MW759899.1_X4_Aglycines         ------------------------------MSVYSSEELLDAGIIYRNKKEQLDVTIGDGMELLIRGEKNKKKKPPTSSSYNSFGMHQSSTDENYYLKDK
QTJ01841.1_X1_Aglycines         MEHPKITFGQKFIDYLGPKNYNLMPLNIKKDVFFTDEELLDAGIIYRNKKEQLDVTIGDGMELLIRGEKNKKKKPPTSSSYNSFGMHQSSTDENYYLKDK
QTJ01842.1_X2_Aglycines         MEHPKITFGQKFIDYLGPKNYNLMPLNIKKDVFFTDEELLDAGIIYRNKKEQLDVTIGDGMELLIRGEKNKKKKPPTSSSYNSFGMHQSSTDENYYLKDK
QTJ01843.1_X3_Aglycines         ---------------------------MDSSSDYNVEELLDAGIIYRNKKEQLDVTIGDGMELLIRGEKNKKKKPPTSSSYNSFGMHQSSTDENYYLKDK
XP_001949648.2_X1_Apisum        ------------------------------MSVYSSEELLDAGIIYRNKKEQLDVTIGDGMELLIRGEKNKKKKPPTSSSYNSFGMHQSSTDENYYLKDK
XP_008183361.1_X2_Apisum        ------------------------------MSVYSSEELLDAGIIYRNKKEQLDVTIGDGMELLIRGEKNKKKKPPTSSSYNSFGMHQSSTDENYYLKDK
XP_029344986.1_X3_Apisum        ----------------------------------------------------------------------------------------------------
XP_027840054.1_X1_Agossypii     ------------------------------MSVYSSEELLDAGIIYRNKKEQLDVTIGDGMELLIRGEKNKKKKPPTSSSYNSFGMHQSSTDENYYLKDK
XP_027840055.1_X2_Agossypii     ------------------------------MSVYSSEELLDAGIIYRNKKEQLDVTIGDGMELLIRGEKNKKKKPPTSSSYNSFGMHQSSTDENYYLKDK
XP_025206085.1_X1_Msacchari     ------------------------------MSVYSSEELLDAGIIYRNKKEQLDVTIGDGMELLIRGEKNKKKKPPASSSYNSFGMHQSSTDENYYLKDK
XP_025206086.1_X2_Msacchari     ------------------------------MSVYSSEELLDAGIIYRNKKEQLDVTIGDGMELLIRGEKNKKKKPPASSSYNSFGMHQSSTDENYYLKDK
XP_025206087.1_X3_Msacchari     ------------------------------MSVYSSEELLDAGIIYRNKKEQLDVTIGDGMELLIRGEKNKKKKPPASSSYNSFGMHQSSTDENYYLKDK
XP_025206088.1_X4_Msacchari     ------------------------------MSVYSSEELLDAGIIYRNKKEQLDVTIGDGMELLIRGEKNKKKKPPASSSYNSFGMHQSSTDENYYLKDK
XP_022174241.1_X1_Mpersicae     ------------------------------MSVYSSEELLDAGIIYRNKKEQLDVTIGDGMELLIRGEKNKKKKPPTSSSYNSFGMHQSSTDENYYLKDK
XP_022174242.1_X2_Mpersicae     ------------------------------MSVYSSEELLDAGIIYRNKKEQLDVTIGDGMELLIRGEKNKKKKPPTSSSYNSFGMHQSSTDENYYLKDK
XP_022174243.1_X3_Mpersicae     ------------------------------------------------------------MELLIRGEKNKKKKPPTSSSYNSFGMHQSSTDENYYLKDK
XP_026806613.1_X1_Rmaidis       ------------------------------MSVYSSEELLDAGIIYRNKKEQLDVTIGDGMELLIRGEKNKKKKPPTSSSYNSFGMHQSSTDENYYLKDK
XP_026806614.1_X2_Rmaidis       ------------------------------MSVYSSEELLDAGIIYRNKKEQLDVTIGDGMELLIRGEKNKKKKPPTSSSYNSFGMHQSSTDENYYLKDK
XP_026806615.1_X3_Rmaidis       ------------------------------MSVYSSEELLDAGIIYRNKKEQLDVTIGDGMELLIRGEKNKKKKPPTSSSYNSFGMHQSSTDENYYLKDK
XP_026806616.1_X4_Rmaidis       ------------------------------MSVYSSEELLDAGIIYRNKKEQLDVTIGDGMELLIRGEKNKKKKPPTSSSYNSFGMHQSSTDENYYLKDK
XP_015366158.1_X1_Dnoxia        ------------------------------MSVYSSEELLDAGIIYRNKKEQLDVTIGDGMELLIRGEKNKKKKPPTSSSYNSFGMHQSSTDENYYLKDK
XP_015366159.1_X2_Dnoxia        ------------------------------MSVYSSEELLDAGIIYRNKKEQLDVTIGDGMELLIRGEKNKKKKPPTSSSYNSFGMHQSSTDENYYLKDK
XP_025407713.1_X1_Sflava        ------------------------------MSMYSSDELLDAGIIYRNKREQLDVTIGDGMELLIRGDKNKKKKPPTSSSYNSFGMHQSSTDENCYLKDK
XP_025407714.1_X2_Sflava        ------------------------------MSMYSSDELLDAGIIYRNKREQLDVTIGDGMELLIRGDKNKKKKPPTSSSYNSFGMHQSSTDENCYLKDK
```

```
                                101       .         .         .         .         :         .         .         .         .        2 200
MW759899.1_X4_Aglycines        YEYDTRSTKSYGSHEHDPYDSESHRGSKRSLHNAEEKKDPSNEDVETNQNILGG-HNGIAAEELNGEEYKEQGPVEMVEDVFEEEEYPEDCFPPNCYKKF
QTJ01841.1_X1_Aglycines        YEYDTRSTKSYGSHEHDPYDSESHRGSKRSLHNAEEKKDPSNEDVETNQN--SG-HNGIAAEELNGEEYKEQGPVEMVEDVFEEEEYPEDCFPPNCYKKF
QTJ01842.1_X2_Aglycines        YEYDTRSTKSYGSHEHDPYDSESHRGSKRSLHNAEEKKDPSNEDVETNQN--SG-HNGIAAEELNGEEYKEQGPVEMVEDVFEEEEYPEDCFPPNCYKKF
QTJ01843.1_X3_Aglycines        YEYDTRSTKSYGSHEHDPYDSESHRGSKRSLHNAEEKKDPSNEDVETNQN--SG-HNGIAAEELNGEEYKEQGPVEMVEDVFEEEEYPEDCFPPNCYKKF
XP_001949648.2_X1_Apisum       YEYDTRSTKSYGSHEHDPYDSESHRGSKRSLHNAEEKKDPSKEDIEINQNVLGG-NDGIDGEEINDGEYKEQGTVEMVEDVFEEEEYPEDCFPPNCYKKF
XP_008183361.1_X2_Apisum       YEYDTRSTKSYGSHEHDPYDSESHRGSKRSLHNAEEKKDPSKEDIEINQNVLGG-NDGIDGEEINDGEYKEQGTVEMVEDVFEEEEYPEDCFPPNCYKKF
XP_029344986.1_X3_Apisum       ----------------------------------------------------------------------------MVEDVFEEEEYPEDCFPPNCYKKF
XP_027840054.1_X1_Agossypii    YEYDTRSTKSYGSHEHDPYDSESHRGSKRSLHNAEEKKDPSNEDVETNQNILGG-HDGIAAEELNGEEYKEQGPVEMVEDVFEEEEYPEDCFPPNCYKKF
XP_027840055.1_X2_Agossypii    YEYDTRSTKSYGSHEHDPYDSESHRGSKRSLHNAEEKKDPSNEDVETNQNILGG-HDGIAAEELNGEEYKEQGPVEMVEDVFEEEEYPEDCFPPNCYKKF
XP_025206085.1_X1_Msacchari    YEYDTRSTKSYGSHEHDPYDSESHRGSKRSLHNTEEKKDPIKEDIEINQDILGGGLDGIAAEEINDDEYKEQGPVEMVEDIFEEEEYPEDCFPPNCYKKF
XP_025206086.1_X2_Msacchari    YEYDTRSTKSYGSHEHDPYDSESHRGSKRSLHNTEEKKDPIKEDIEINQDILGGGLDGIAAEEINDDEYKEQGPVEMVEDIFEEEEYPEDCFPPNCYKKF
XP_025206087.1_X3_Msacchari    YEYDTRSTKSYGSHEHDPYDSESHRGSKRSLHNTEEKKDPIKEDIEINQD--SGGLDGIAAEEINDDEYKEQGPVEMVEDIFEEEEYPEDCFPPNCYKKF
XP_025206088.1_X4_Msacchari    YEYDTRSTKSYGSHEHDPYDSESHRGSKRSLHNTEEKKDPIKEDIEINQD--SGGLDGIAAEEINDDEYKEQGPVEMVEDIFEEEEYPEDCFPPNCYKKF
XP_022174241.1_X1_Mpersicae    YEYDTRSTKSYGSHEHDPYDSESHRGSKRSLHNAEEKKDPCKEDVETNQNVLGG-NDAIAAEEINDDEYKEQGPVEMVEDVFEEEEYPEDCFPPNCYKKF
XP_022174242.1_X2_Mpersicae    YEYDTRSTKSYGSHEHDPYDSESHRGSKRSLHNAEEKKDPCKEDVETNQNVLGG-NDAIAAEEINDDEYKEQGPVEMVEDVFEEEEYPEDCFPPNCYKKF
XP_022174243.1_X3_Mpersicae    YEYDTRSTKSYGSHEHDPYDSESHRGSKRSLHNAEEKKDPCKEDVETNQNVLGG-NDAIAAEEINDDEYKEQGPVEMVEDVFEEEEYPEDCFPPNCYKKF
XP_026806613.1_X1_Rmaidis      YEYDTRSTKSYGSHEHDPYDSESHRGSKRSLHNAEEKKDPSKEDVEINQNILGG-NDGIAAEEINGDEYKEQGPVEMVEDVFEEEEYPEDCFPPNCYKKF
XP_026806614.1_X2_Rmaidis      YEYDTRSTKSYGSHEHDPYDSESHRGSKRSLHNAEEKKDPSKEDVEINQNILGG-NDGIAAEEINGDEYKEQGPVEMVEDVFEEEEYPEDCFPPNCYKKF
XP_026806615.1_X3_Rmaidis      YEYDTRSTKSYGSHEHDPYDSESHRGSKRSLHNAEEKKDPSKEDVEINQN--SG-NDGIAAEEINGDEYKEQGPVEMVEDVFEEEEYPEDCFPPNCYKKF
XP_026806616.1_X4_Rmaidis      YEYDTRSTKSYGSHEHDPYDSESHRGSKRSLHNAEEKKDPSKEDVEINQN--SG-NDGIAAEEINGDEYKEQGPVEMVEDVFEEEEYPEDCFPPNCYKKF
XP_015366158.1_X1_Dnoxia       YEYDTRSTKSYGSHEHDPYDSESHRGSKRSLQNAEEKKDPSKEDIDTNQNVLGG-NDGIAAEEINEDEYKEQGPVEMVEDVFEEEEYPEDCFPPNCYKKF
XP_015366159.1_X2_Dnoxia       YEYDTRSTKSYGSHEHDPYDSESHRGSKRSLQNAEEKKDPSKEDIDTNQNVLGG-NDGIAAEEINEDEYKEQGPVEMVEDVFEEEEYPEDCFPPNCYKKF
XP_025407713.1_X1_Sflava       YEYDTRSTKSYGSHEHDPYDSESHRGSKRSLHNAEEKKDPSKEDVEIDPHGLE-GNDGITTDVLNDEEYKEQGQVEMVEDVFEEEEYPEDCFPPNCYKKF
XP_025407714.1_X2_Sflava       YEYDTRSTKSYGSHEHDPYDSESHRGSKRSLHNAEEKKDPSKEDVEIDPHGLE-GNDGITTDVLNDEEYKEQGQVEMVEDVFEEEEYPEDCFPPNCYKKF
```

```
                                                 .......DIII S1.......                ........DIII S2..........      ..DIII..>
                        201          .         .         .         .         :         .         .         .         .        3 300
MW759899.1_X4_Aglycines      PFLAGDDETPFWLGWGQLRLKTFQLIENKYFETAVITMILLSSLALALEDVHLQKRPVLQDILYYMDRIFTVIFFLEMLIKWLALGFRNYFTNAWCWLDF
QTJ01841.1_X1_Aglycines      PFLAGDDETPFWLGWGQLRLKTFQLIENKYFETAVITMILLSSLALALEDVHLQKRPVLQDILYYMDRIFTVIFFLEMLIKWLALGFRNYFTNAWCWLDF
QTJ01842.1_X2_Aglycines      PFLAGDDETPFWLGWGQLRLKTFQLIENKYFETAVITMILLSSLALALEDVHLQKRPVLQDILYYMDRIFTVIFFLEMLIKWLALGFRNYFTNAWCWLDF
QTJ01843.1_X3_Aglycines      PFLAGDDETPFWLGWGQLRLKTFQLIENKYFETAVITMILLSSLALALEDVHLQKRPVLQDILYYMDRIFTVIFFLEMLIKWLALGFRNYFTNAWCWLDF
XP_001949648.2_X1_Apisum     PFLAGDDETPFWLGWGQLRLKTFQLIENKYFETAVITMILLSSLALALEDVHLQHRPVLQDILYYMDRIFTVIFFLEMLIKWLALGFRNYFTNAWCWLDF
XP_008183361.1_X2_Apisum     PFLAGDDETPFWLGWGQLRLKTFQLIENKYFETAVITMILLSSLALALEDVHLQHRPVLQDILYYMDRIFTVIFFLEMLIKWLALGFRNYFTNAWCWLDF
XP_029344986.1_X3_Apisum     PFLAGDDETPFWLGWGQLRLKTFQLIENKYFETAVITMILLSSLALALEDVHLQHRPVLQDILYYMDRIFTVIFFLEMLIKWLALGFRNYFTNAWCWLDF
XP_027840054.1_X1_Agossypii  PFLAGDDETPFWLGWGQLRLKTFQLIENKYFETAVITMILLSSLALALEDVHLQKRPVLQDILYYMDRIFTVIFFLEMLIKWLALGFRNYFTNAWCWLDF
XP_027840055.1_X2_Agossypii  PFLAGDDETPFWLGWGQLRLKTFQLIENKYFETAVITMILLSSLALALEDVHLQKRPVLQDILYYMDRIFTVIFFLEMLIKWLALGFRNYFTNAWCWLDF
XP_025206085.1_X1_Msacchari  PFLAGDDETPFWLGWGQLRLKTFQLIENKYFETAVITMILLSSLALALEDVHLQHRPVLQDILYYMDRIFTVIFFLEMLIKWLALGFRNYFTNAWCWLDF
XP_025206086.1_X2_Msacchari  PFLAGDDETPFWLGWGQLRLKTFQLIENKYFETAVITMILLSSLALALEDVHLQHRPVLQDILYYMDRIFTVIFFLEMLIKWLALGFRNYFTNAWCWLDF
XP_025206087.1_X3_Msacchari  PFLAGDDETPFWLGWGQLRLKTFQLIENKYFETAVITMILLSSLALALEDVHLQHRPVLQDILYYMDRIFTVIFFLEMLIKWLALGFRNYFTNAWCWLDF
XP_025206088.1_X4_Msacchari  PFLAGDDETPFWLGWGQLRLKTFQLIENKYFETAVITMILLSSLALALEDVHLQHRPVLQDILYYMDRIFTVIFFLEMLIKWLALGFRNYFTNAWCWLDF
XP_022174241.1_X1_Mpersicae  PFLAGDDETPFWLGWGQLRLKTFQLIENKYFETAVITMILLSSLALALEDVHLQHRPVLQDILYYMDRIFTVIFFLEMLIKWLALGFRNYFTNAWCWLDF
XP_022174242.1_X2_Mpersicae  PFLAGDDETPFWLGWGQLRLKTFQLIENKYFETAVITMILLSSLALALEDVHLQHRPVLQDILYYMDRIFTVIFFLEMLIKWLALGFRNYFTNAWCWLDF
XP_022174243.1_X3_Mpersicae  PFLAGDDETPFWLGWGQLRLKTFQLIENKYFETAVITMILLSSLALALEDVHLQHRPVLQDILYYMDRIFTVIFFLEMLIKWLALGFRNYFTNAWCWLDF
XP_026806613.1_X1_Rmaidis    PFLAGDDETPFWLGWGQLRLKTFQLIENKYFETAVITMILLSSLALALEDVHLQHRPVLQDILYYMDRIFTVIFFLEMLIKWLALGFRNYFTNAWCWLDF
XP_026806614.1_X2_Rmaidis    PFLAGDDETPFWLGWGQLRLKTFQLIENKYFETAVITMILLSSLALALEDVHLQHRPVLQDILYYMDRIFTVIFFLEMLIKWLALGFRNYFTNAWCWLDF
XP_026806615.1_X3_Rmaidis    PFLAGDDETPFWLGWGQLRLKTFQLIENKYFETAVITMILLSSLALALEDVHLQHRPVLQDILYYMDRIFTVIFFLEMLIKWLALGFRNYFTNAWCWLDF
XP_026806616.1_X4_Rmaidis    PFLAGDDETPFWLGWGQLRLKTFQLIENKYFETAVITMILLSSLALALEDVHLQHRPVLQDILYYMDRIFTVIFFLEMLIKWLALGFRNYFTNAWCWLDF
XP_015366158.1_X1_Dnoxia     PFLAGDDETPFWLGWGQLRLKTFQLIENKYFETAVITMILLSSLALALEDVHLQHRPVLQDILYYMDRIFTVIFFLEMLIKWLALGFRNYFTNAWCWLDF
XP_015366159.1_X2_Dnoxia     PFLAGDDETPFWLGWGQLRLKTFQLIENKYFETAVITMILLSSLALALEDVHLQHRPVLQDILYYMDRIFTVIFFLEMLIKWLALGFRNYFTNAWCWLDF
XP_025407713.1_X1_Sflava     PFLAGDDETPFWLGWGQLRLKTFQLIENKYFETAVITMILLSSLALALEDVHLQHRPVLQDILYYMDRIFTVIFFLEMLIKWLALGFRNYFTNAWCWLDF
XP_025407714.1_X2_Sflava     PFLAGDDETPFWLGWGQLRLKTFQLIENKYFETAVITMILLSSLALALEDVHLQHRPVLQDILYYMDRIFTVIFFLEMLIKWLALGFRNYFTNAWCWLDF
```

```
                              <DIII.>                                         <.DIII.>    .......DIII S4.........
                           301         .         .         .         .         :         .         .         .         .         4 400
MW759899.1_X4_Aglycines       IIVMVSL-----------------------------------------INFVAALLGASGIQAFKTMRTLRALRPLRAMARMQGMRVVVNALVQAIPSIF
QTJ01841.1_X1_Aglycines       IIVMLSLLVNLAAIWFGAADIPAFRSMRTLRALRPLRAVSRWEGMRVSINFVAALLGASGIQAFKTMRTLRALRPLRAMARMQGMRVVVNALVQAIPSIF
QTJ01842.1_X2_Aglycines       IIVMVSL-----------------------------------------INFVAALLGASGIQAFKTMRTLRALRPLRAMARMQGMRVVVNALVQAIPSIF
QTJ01843.1_X3_Aglycines       IIVMLSLLVNLAAIWFGAADIPAFRSMRTLRALRPLRAVSRWEGMRVSINFVAALLGASGIQAFKTMRTLRALRPLRAMARMQGMRVVVNALVQAIPSIF
XP_001949648.2_X1_Apisum      IIVMVSL-----------------------------------------INFVAALLGASGIQAFKTMRTLRALRPLRAMARMQGMRVVVNALVQAIPSIF
XP_008183361.1_X2_Apisum      IIVMLSL-----------------------------------------VNLAAIWAGAADIPAFRSMRTLRALRPLRAVSRWEGMRVVVNALVQAIPSIF
XP_029344986.1_X3_Apisum      IIVMVSL-----------------------------------------INFVAALLGASGIQAFKTMRTLRALRPLRAMARMQGMRVVVNALVQAIPSIF
XP_027840054.1_X1_Agossypii   IIVMVSL-----------------------------------------INFVAALLGASGIQAFKTMRTLRALRPLRAMARMQGMRVVVNALVQAIPSIF
XP_027840055.1_X2_Agossypii   IIVMLSL-----------------------------------------VNLAAIWFGAADIPAFRSMRTLRALRPLRAVSRWEGMRVVVNALVQAIPSIF
XP_025206085.1_X1_Msacchari   IIVMVSL-----------------------------------------INFVAALLGASGVQAFKTMRTLRALRPLRAMARMQGMRVVVNALVQAIPSIF
XP_025206086.1_X2_Msacchari   IIVMLSL-----------------------------------------VNLAAIWFGAADIPAFRSMRTLRALRPLRAVSRWEGMRVVVNALVQAIPSIF
XP_025206087.1_X3_Msacchari   IIVMVSL-----------------------------------------INFVAALLGASGVQAFKTMRTLRALRPLRAMARMQGMRVVVNALVQAIPSIF
XP_025206088.1_X4_Msacchari   IIVMLSL-----------------------------------------VNLAAIWFGAADIPAFRSMRTLRALRPLRAVSRWEGMRVVVNALVQAIPSIF
XP_022174241.1_X1_Mpersicae   IIVMVSL-----------------------------------------INFVAALLGASGIQAFKTMRTLRALRPLRAMARMQGMRVVVNALVQAIPSIF
XP_022174242.1_X2_Mpersicae   IIVMLSL-----------------------------------------VNLAAIWAGAADIPAFRSMRTLRALRPLRAVSRWEGMRVVVNALVQAIPSIF
XP_022174243.1_X3_Mpersicae   IIVMVSL-----------------------------------------INFVAALLGASGIQAFKTMRTLRALRPLRAMARMQGMRVVVNALVQAIPSIF
XP_026806613.1_X1_Rmaidis     IIVMVSL-----------------------------------------INFVAALLGASGIQAFKTMRTLRALRPLRAMARMQGMRVVVNALVQAIPSIF
XP_026806614.1_X2_Rmaidis     IIVMLSL-----------------------------------------VNLAAIWFGAADIPAFRSMRTLRALRPLRAVSRWEGMRVVVNALVQAIPSIF
XP_026806615.1_X3_Rmaidis     IIVMVSL-----------------------------------------INFVAALLGASGIQAFKTMRTLRALRPLRAMARMQGMRVVVNALVQAIPSIF
XP_026806616.1_X4_Rmaidis     IIVMLSL-----------------------------------------VNLAAIWFGAADIPAFRSMRTLRALRPLRAVSRWEGMRVVVNALVQAIPSIF
XP_015366158.1_X1_Dnoxia      IIVMVSL-----------------------------------------INFVAALLGASGIQAFKTMRTLRALRPLRAMARMQGMRVVVNALVQAIPSIF
XP_015366159.1_X2_Dnoxia      IIVMLSL-----------------------------------------VNLAAIWAGMADIPAFRSMRTLRALRPLRAVSRWEGMRVVVNALVQAIPSIF
XP_025407713.1_X1_Sflava      IIVMVSL-----------------------------------------INFVAALLGASGIQAFKTMRTLRALRPLRAMARMQGMRVVVNALVQAIPSIF
XP_025407714.1_X2_Sflava      IIVMLSL-----------------------------------------VNLAAIWFGFADIPAFRSMRTLRALRPLRAVSRWEGMRVVVNALVQAIPSIF
```

```
                                  .......DIII S5.........
                            401           .          .          .          .          :          .          .          .          .          5 500
MW759899.1_X4_Aglycines       NVLLVCLIFWLIFAIMGVQLFAGKYYKCVDN-TGKTLNHEIIPDKNVCLAENYKWENSKMNFDHVGNAYLCLFQVATFNGWMEIMRDAVDSRDTHGKQPI
QTJ01841.1_X1_Aglycines       NVLLVCLIFWLIFAIMGVQLFAGKYYKCVDN-TGKTLNHEIIPDKNVCLAENYKWENSKMNFDHVGNAYLCLFQVATFNGWMEIMRDAVDSRDTHGKQPI
QTJ01842.1_X2_Aglycines       NVLLVCLIFWLIFAIMGVQLFAGKYYKCVDN-TGKTLNHEIIPDKNVCLAENYKWENSKMNFDHVGNAYLCLFQVATFNGWMEIMRDAVDSRDTHGKQPI
QTJ01843.1_X3_Aglycines       NVLLVCLIFWLIFAIMGVQLFAGKYYKCVDN-TGKTLNHEIIPDKNVCLAENYKWENSKMNFDHVGNAYLCLFQVATFNGWMEIMRDAVDSRDTHGKQPI
XP_001949648.2_X1_Apisum      NVLLVCLIFWLIFAIMGVQLFAGKYYKCVDS-EGNTLNHEIIPDRNVCINENYKWENSKMNFDHVGNAYLCLFQVATFNGWMEIMRDAVDSRDTYGKQPI
XP_008183361.1_X2_Apisum      NVLLVCLIFWLIFAIMGVQLFAGKYYKCVDS-EGNTLNHEIIPDRNVCINENYKWENSKMNFDHVGNAYLCLFQVATFNGWMEIMRDAVDSRDTYGKQPI
XP_029344986.1_X3_Apisum      NVLLVCLIFWLIFAIMGVQLFAGKYYKCVDS-EGNTLNHEIIPDRNVCINENYKWENSKMNFDHVGNAYLCLFQVATFNGWMEIMRDAVDSRDTYGKQPI
XP_027840054.1_X1_Agossypii   NVLLVCLIFWLIFAIMGVQLFAGKYYKCVDN-TGKTLNHEIIPDKNVCLAENYKWENSKMNFDHVGNAYLCLFQVATFNGWMEIMRDAVDSRDTHGKQPI
XP_027840055.1_X2_Agossypii   NVLLVCLIFWLIFAIMGVQLFAGKYYKCVDN-TGKTLNHEIIPDKNVCLAENYKWENSKMNFDHVGNAYLCLFQVATFNGWMEIMRDAVDSRDTHGKQPI
XP_025206085.1_X1_Msacchari   NVLLVCLIFWLIFAIMGVQLFAGKYYKCVDPKDGKMLNHEIIPDKNVCIAENYKWENSKMNFDHVGNAYLCLFQVATFNGWMEIMRDAVDSRDTHGKQPI
XP_025206086.1_X2_Msacchari   NVLLVCLIFWLIFAIMGVQLFAGKYYKCVDPKDGKMLNHEIIPDKNVCIAENYKWENSKMNFDHVGNAYLCLFQVATFNGWMEIMRDAVDSRDTHGKQPI
XP_025206087.1_X3_Msacchari   NVLLVCLIFWLIFAIMGVQLFAGKYYKCVDPKDGKMLNHEIIPDKNVCIAENYKWENSKMNFDHVGNAYLCLFQVATFNGWMEIMRDAVDSRDTHGKQPI
XP_025206088.1_X4_Msacchari   NVLLVCLIFWLIFAIMGVQLFAGKYYKCVDPKDGKMLNHEIIPDKNVCIAENYKWENSKMNFDHVGNAYLCLFQVATFNGWMEIMRDAVDSRDTHGKQPI
XP_022174241.1_X1_Mpersicae   NVLLVCLIFWLIFAIMGVQLFAGKYYKCVDI-DGKTLNHEIIPDKNVCIAENYKWENSKMNFDHVGNAYLCLFQVATFNGWMEIMRDAVDSRDTYGKQPI
XP_022174242.1_X2_Mpersicae   NVLLVCLIFWLIFAIMGVQLFAGKYYKCVDI-DGKTLNHEIIPDKNVCIAENYKWENSKMNFDHVGNAYLCLFQVATFNGWMEIMRDAVDSRDTYGKQPI
XP_022174243.1_X3_Mpersicae   NVLLVCLIFWLIFAIMGVQLFAGKYYKCVDI-DGKTLNHEIIPDKNVCIAENYKWENSKMNFDHVGNAYLCLFQVATFNGWMEIMRDAVDSRDTYGKQPI
XP_026806613.1_X1_Rmaidis     NVLLVCLIFWLIFAIMGVQLFAGKYYKCVDPKDGKTLNHEIIPDKNVCIAEMYKWENSKMNFDHVGNAYLCLFQVATFNGWMEIMRDAVDSRDTHGKQPI
XP_026806614.1_X2_Rmaidis     NVLLVCLIFWLIFAIMGVQLFAGKYYKCVDPKDGKTLNHEIIPDKNVCIAEMYKWENSKMNFDHVGNAYLCLFQVATFNGWMEIMRDAVDSRDTHGKQPI
XP_026806615.1_X3_Rmaidis     NVLLVCLIFWLIFAIMGVQLFAGKYYKCVDPKDGKTLNHEIIPDKNVCIAEMYKWENSKMNFDHVGNAYLCLFQVATFNGWMEIMRDAVDSRDTHGKQPI
XP_026806616.1_X4_Rmaidis     NVLLVCLIFWLIFAIMGVQLFAGKYYKCVDPKDGKTLNHEIIPDKNVCIAEMYKWENSKMNFDHVGNAYLCLFQVATFNGWMEIMRDAVDSRDTHGKQPI
XP_015366158.1_X1_Dnoxia      NVLLVCLIFWLIFAIMGVQLFAGKYYKCVDPKDGKTLNHEIIPDRNVCLAENYKWENSKMNFDHVGNAYLCLFQVATFNGWMEIMRDAVDSRDTYGKQPI
XP_015366159.1_X2_Dnoxia      NVLLVCLIFWLIFAIMGVQLFAGKYYKCVDPKDGKTLNHEIIPDRNVCLAENYKWENSKMNFDHVGNAYLCLFQVATFNGWMEIMRDAVDSRDTYGKQPI
XP_025407713.1_X1_Sflava      NVLLVCLIFWLIFAIMGVQLFAGKYYKCVDPKTDKMVNHEIIPDKNACIAENYTWQNSKMNFDHVGNAYLCLFQVATFNGWMEIMRDAVDSRDLHGKQPI
XP_025407714.1_X2_Sflava      NVLLVCLIFWLIFAIMGVQLFAGKYYKCVDPKTDKMVNHEIIPDKNACIAENYTWQNSKMNFDHVGNAYLCLFQVATFNGWMEIMRDAVDSRDLHGKQPI
```

```
                                   .........DIII S6.........                                                       ....DIV S1.....>
                              501       .         .         .         .         :         .         .         .         .       6 600
MW759899.1_X4_Aglycines       REINNYMYFYFVFFIIFGSFFTLNLFIGVIIDNFNEQKKKTGASLEMFMTEDQKKYYNAMKKMSSKKPLKAIPRPRWRPQSIVFQIVTDKKFDMLIMLFI
QTJ01841.1_X1_Aglycines       REINNYMYFYFVFFIIFGSFFTLNLFIGVIIDNFNEQKKKT-ASLEMFMTEDQKKYYNAMKKMSSKKPLKAIPRPRWRPQSIVFQIVTDKKFDMLIMLFI
QTJ01842.1_X2_Aglycines       REINNYMYFYFVFFIIFGSFFTLNLFIGVIIDNFNEQKKKTGASLEMFMTEDQKKYYNAMKKMSSKKPLKAIPRPRWRPQSIVFQIVTDKKFDMLIMLFI
QTJ01843.1_X3_Aglycines       REINNYMYFYFVFFIIFGSFFTLNLFIGVIIDNFNEQKKKTGASLEMFMTEDQKKYYNAMKKMSSKKPLKAIPRPRWRPQSIVFQIVTDKKFDMLIMLFI
XP_001949648.2_X1_Apisum      REINNYMYFYFVFFIIFGSFFTLNLFIGVIIDNFNEQKKKTGASLEMFMTEDQKKYYNAMKKMSSKKPLKAIPRPRWKPQSIVFQIVTDKKFDMLIMLFI
XP_008183361.1_X2_Apisum      REINNYMYFYFVFFIIFGSFFTLNLFIGVIIDNFNEQKKKTGASLEMFMTEDQKKYYNAMKKMSSKKPLKAIPRPRWKPQSIVFQIVTDKKFDMLIMLFI
XP_029344986.1_X3_Apisum      REINNYMYFYFVFFIIFGSFFTLNLFIGVIIDNFNEQKKKTGASLEMFMTEDQKKYYNAMKKMSSKKPLKAIPRPRWKPQSIVFQIVTDKKFDMLIMLFI
XP_027840054.1_X1_Agossypii   REINNYMYFYFVFFIIFGSFFTLNLFIGVIIDNFNEQKKKTGASLEMFMTEDQKKYYNAMKKMSSKKPLKAIPRPRWRPQSIVFQIVTDKKFDMLIMLFI
XP_027840055.1_X2_Agossypii   REINNYMYFYFVFFIIFGSFFTLNLFIGVIIDNFNEQKKKTGASLEMFMTEDQKKYYNAMKKMSSKKPLKAIPRPRWRPQSIVFQIVTDKKFDMLIMLFI
XP_025206085.1_X1_Msacchari   REINNYMYFYFVFFIIFGSFFTLNLFIGVIIDNFNEQKKKTGASLEMFMTEDQKKYYNAMKKMSSKKPLKAIPRPRWRPQSIVFQIVTDKKFDMLIMLFI
XP_025206086.1_X2_Msacchari   REINNYMYFYFVFFIIFGSFFTLNLFIGVIIDNFNEQKKKTGASLEMFMTEDQKKYYNAMKKMSSKKPLKAIPRPRWRPQSIVFQIVTDKKFDMLIMLFI
XP_025206087.1_X3_Msacchari   REINNYMYFYFVFFIIFGSFFTLNLFIGVIIDNFNEQKKKTGASLEMFMTEDQKKYYNAMKKMSSKKPLKAIPRPRWRPQSIVFQIVTDKKFDMLIMLFI
XP_025206088.1_X4_Msacchari   REINNYMYFYFVFFIIFGSFFTLNLFIGVIIDNFNEQKKKTGASLEMFMTEDQKKYYNAMKKMSSKKPLKAIPRPRWRPQSIVFQIVTDKKFDMLIMLFI
XP_022174241.1_X1_Mpersicae   REINNYMYFYFVFFIIFGSFFTLNLFIGVIIDNFNEQKKKTGASLEMFMTEDQKKYYNAMKKMSSKKPLKAIPRPRWKPQSIVFQIVTDKKFDMLIMLFI
XP_022174242.1_X2_Mpersicae   REINNYMYFYFVFFIIFGSFFTLNLFIGVIIDNFNEQKKKTGASLEMFMTEDQKKYYNAMKKMSSKKPLKAIPRPRWKPQSIVFQIVTDKKFDMLIMLFI
XP_022174243.1_X3_Mpersicae   REINNYMYFYFVFFIIFGSFFTLNLFIGVIIDNFNEQKKKTGASLEMFMTEDQKKYYNAMKKMSSKKPLKAIPRPRWKPQSIVFQIVTDKKFDMLIMLFI
XP_026806613.1_X1_Rmaidis     REINNYMYFYFVFFIIFGSFFTLNLFIGVIIDNFNEQKKKTGASLEMFMTEDQKKYYNAMKKMSSKKPLKAIPRPRWRPQSIVFQIVTDKKFDMLIMLFI
XP_026806614.1_X2_Rmaidis     REINNYMYFYFVFFIIFGSFFTLNLFIGVIIDNFNEQKKKTGASLEMFMTEDQKKYYNAMKKMSSKKPLKAIPRPRWRPQSIVFQIVTDKKFDMLIMLFI
XP_026806615.1_X3_Rmaidis     REINNYMYFYFVFFIIFGSFFTLNLFIGVIIDNFNEQKKKTGASLEMFMTEDQKKYYNAMKKMSSKKPLKAIPRPRWRPQSIVFQIVTDKKFDMLIMLFI
XP_026806616.1_X4_Rmaidis     REINNYMYFYFVFFIIFGSFFTLNLFIGVIIDNFNEQKKKTGASLEMFMTEDQKKYYNAMKKMSSKKPLKAIPRPRWRPQSIVFQIVTDKKFDMLIMLFI
XP_015366158.1_X1_Dnoxia      REINNYMYFYFVFFIIFGSFFTLNLFIGVIIDNFNEQKKKTGASLEMFMTEDQKKYYNAMKKMSSKKPLKAIPRPRWKPQSIVFQIVTDKKFDMLIMLFI
XP_015366159.1_X2_Dnoxia      REINNYMYFYFVFFIIFGSFFTLNLFIGVIIDNFNEQKKKTGASLEMFMTEDQKKYYNAMKKMSSKKPLKAIPRPRWKPQSIVFQIVTDKKFDMLIMLFI
XP_025407713.1_X1_Sflava      REINNYMYFYFVFFIIFGSFFTLNLFIGVIIDNFNEQKKKTGASLEMFMTEDQKKYYNAMKKMSSKKPLKAIPRPRWKPQSIVFQIVTDKKFDMLIMLFI
XP_025407714.1_X2_Sflava      REINNYMYFYFVFFIIFGSFFTLNLFIGVIIDNFNEQKKKTGASLEMFMTEDQKKYYNAMKKMSSKKPLKAIPRPRWKPQSIVFQIVTDKKFDMLIMLFI
```

```
                              <.DIV S1.          .........DIV S2..........      .......DIV S3...........          .....DIV S4....>
                         601           .         .         .         .         :         .         .         .         .        7 700
MW759899.1_X4_Aglycines       GLNMLTMTLDHYQQTKLFTDVLERLNQIFIAIFSTECLLKIFALRYYYFKEPWNLFDFVVVILSLAGLVLSDLISKYFVSPTLLRVVRVAKVGRVLRLVK
QTJ01841.1_X1_Aglycines       GLNMLTMTLDHYQQTKLFTDVLERLNQIFIAIFSTECLLKIFALRYYYFKEPWNLFDFVVVILSLAGLVLSDLISKYFVSPTLLRVVRVAKVGRVLRLVK
QTJ01842.1_X2_Aglycines       GLNMLTMTLDHYQQTKLFTDVLERLNQIFIAIFSTECLLKIFALRYYYFKEPWNLFDFVVVILSLAGLVLSDLISKYFVSPTLLRVVRVAKVGRVLRLVK
QTJ01843.1_X3_Aglycines       GLNMLTMTLDHYQQTKLFTDVLERLNQIFIAIFSTECLLKIFALRYYYFKEPWNLFDFVVVILSLAGLVLSDLISKYFVSPTLLRVVRVAKVGRVLRLVK
XP_001949648.2_X1_Apisum      GFNMLTMTLDHYQQTKLFTDVLERLNQIFIAIFSTECLLKIFALRYYYFKEPWNLFDFVVVILSLAGLVLSDLISKYFVSPTLLRVVRVAKVGRVLRLVK
XP_008183361.1_X2_Apisum      GFNMLTMTLDHYQQTKLFTDVLERLNQIFIAIFSTECLLKIFALRYYYFKEPWNLFDFVVVILSLAGLVLSDLISKYFVSPTLLRVVRVAKVGRVLRLVK
XP_029344986.1_X3_Apisum      GFNMLTMTLDHYQQTKLFTDVLERLNQIFIAIFSTECLLKIFALRYYYFKEPWNLFDFVVVILSLAGLVLSDLISKYFVSPTLLRVVRVAKVGRVLRLVK
XP_027840054.1_X1_Agossypii   GLNMLTMTLDHYQQTKLFTDVLERLNQIFIAIFSTECLLKIFALRYYYFKEPWNLFDFVVVILSLAGLVLSDLISKYFVSPTLLRVVRVAKVGRVLRLVK
XP_027840055.1_X2_Agossypii   GLNMLTMTLDHYQQTKLFTDVLERLNQIFIAIFSTECLLKIFALRYYYFKEPWNLFDFVVVILSLAGLVLSDLISKYFVSPTLLRVVRVAKVGRVLRLVK
XP_025206085.1_X1_Msacchari   GLNMLTMTLDHYQQTKLFTDVLERLNQIFIAIFSTECLLKIFALRYYYFKEPWNLFDFVVVILSLAGLVLSDLISKYFVSPTLLRVVRVAKVGRVLRLVK
XP_025206086.1_X2_Msacchari   GLNMLTMTLDHYQQTKLFTDVLERLNQIFIAIFSTECLLKIFALRYYYFKEPWNLFDFVVVILSLAGLVLSDLISKYFVSPTLLRVVRVAKVGRVLRLVK
XP_025206087.1_X3_Msacchari   GLNMLTMTLDHYQQTKLFTDVLERLNQIFIAIFSTECLLKIFALRYYYFKEPWNLFDFVVVILSLAGLVLSDLISKYFVSPTLLRVVRVAKVGRVLRLVK
XP_025206088.1_X4_Msacchari   GLNMLTMTLDHYQQTKLFTDVLERLNQIFIAIFSTECLLKIFALRYYYFKEPWNLFDFVVVILSLAGLVLSDLISKYFVSPTLLRVVRVAKVGRVLRLVK
XP_022174241.1_X1_Mpersicae   GFNMLTMTLDHYQQTKLFTDVLERLNQIFIAIFSTECLLKIFALRYYYFKEPWNLFDFVVVILSLAGLVLSDLISKYFVSPTLLRVVRVAKVGRVLRLVK
XP_022174242.1_X2_Mpersicae   GFNMLTMTLDHYQQTKLFTDVLERLNQIFIAIFSTECLLKIFALRYYYFKEPWNLFDFVVVILSLAGLVLSDLISKYFVSPTLLRVVRVAKVGRVLRLVK
XP_022174243.1_X3_Mpersicae   GFNMLTMTLDHYQQTKLFTDVLERLNQIFIAIFSTECLLKIFALRYYYFKEPWNLFDFVVVILSLAGLVLSDLISKYFVSPTLLRVVRVAKVGRVLRLVK
XP_026806613.1_X1_Rmaidis     GFNMLTMTLDHYQQTKLFTDVLERLNQIFIAIFSTECLLKIFALRYYYFKEPWNLFDFVVVILSLAGLVLSDLISKYFVSPTLLRVVRVAKVGRVLRLVK
XP_026806614.1_X2_Rmaidis     GFNMLTMTLDHYQQTKLFTDVLERLNQIFIAIFSTECLLKIFALRYYYFKEPWNLFDFVVVILSLAGLVLSDLISKYFVSPTLLRVVRVAKVGRVLRLVK
XP_026806615.1_X3_Rmaidis     GFNMLTMTLDHYQQTKLFTDVLERLNQIFIAIFSTECLLKIFALRYYYFKEPWNLFDFVVVILSLAGLVLSDLISKYFVSPTLLRVVRVAKVGRVLRLVK
XP_026806616.1_X4_Rmaidis     GFNMLTMTLDHYQQTKLFTDVLERLNQIFIAIFSTECLLKIFALRYYYFKEPWNLFDFVVVILSLAGLVLSDLISKYFVSPTLLRVVRVAKVGRVLRLVK
XP_015366158.1_X1_Dnoxia      GFNMLTMTLDHYQQTKLFTDVLERLNQIFIAIFSTECLLKIFALRYYYFKEPWNLFDFVVVILSLAGLVLSDLISKYFVSPTLLRVVRVAKVGRVLRLVK
XP_015366159.1_X2_Dnoxia      GFNMLTMTLDHYQQTKLFTDVLERLNQIFIAIFSTECLLKIFALRYYYFKEPWNLFDFVVVILSLAGLVLSDLISKYFVSPTLLRVVRVAKVGRVLRLVK
XP_025407713.1_X1_Sflava      GFNMLTMTLDHYQQTKLFTDVLELLNQIFIAIFSSECLLKIFALRYYYFKEPWNLFDFIVVILSLAGLVLSDLISKYFVSPTLLRVVRVAKVGRVLRLVK
XP_025407714.1_X2_Sflava      GFNMLTMTLDHYQQTKLFTDVLELLNQIFIAIFSSECLLKIFALRYYYFKEPWNLFDFIVVILSLAGLVLSDLISKYFVSPTLLRVVRVAKVGRVLRLVK
```

```
                                      DIV S4                      .......DIV S5..........
                         701            .         .         .         .         :         .         .         .         .    8 800
MW759899.1_X4_Aglycines        GAKGIRTLLFALAMSLPALFNICLLLFLVMFIFAIFGMSFFMNVDNHGGLDEDYNFRTFGQSMILLFMLSTSSGWDAVLDGITNEDDCDKPNLEMGITGS
QTJ01841.1_X1_Aglycines        GAKGIRTLLFALAMSLPALFNICLLLFLVMFIFAIFGMSFFMNVDNHGGLDEDYNFRTFGQSMILLFMLSTSSGWDAVLDGITNEDDCDKPNLEMGITGS
QTJ01842.1_X2_Aglycines        GAKGIRTLLFALAMSLPALFNICLLLFLVMFIFAIFGMSFFMNVDNHGGLDEDYNFRTFGQSMILLFMLSTSSGWDAVLDGITNEDDCDKPNLEMGITGS
QTJ01843.1_X3_Aglycines        GAKGIRTLLFALAMSLPALFNICLLLFLVMFIFAIFGMSFFMNVDNHGGLDEDYNFRTFGQSMILLFMLSTSSGWDAVLDGITNEDDCDKPNLEMGITGS
XP_001949648.2_X1_Apisum       GAKGIRTLLFALAMSLPALFNICLLLFLVMFIFAIFGMSFFMNVDSHGGLDEDYNFRTFGQSMILLFMLSTSSGWDAVLDGITNEENCEKQNLEMGITGS
XP_008183361.1_X2_Apisum       GAKGIRTLLFALAMSLPALFNICLLLFLVMFIFAIFGMSFFMNVDSHGGLDEDYNFRTFGQSMILLFMLSTSSGWDAVLDGITNEENCEKQNLEMGITGS
XP_029344986.1_X3_Apisum       GAKGIRTLLFALAMSLPALFNICLLLFLVMFIFAIFGMSFFMNVDSHGGLDEDYNFRTFGQSMILLFMLSTSSGWDAVLDGITNEENCEKQNLEMGITGS
XP_027840054.1_X1_Agossypii    GAKGIRTLLFALAMSLPALFNICLLLFLVMFIFAIFGMSFFMNVDNHGGLDEDYNFRTFGQSMILLFMLSTSSGWDAVLDGITNEEGCDKPNLEMGITGS
XP_027840055.1_X2_Agossypii    GAKGIRTLLFALAMSLPALFNICLLLFLVMFIFAIFGMSFFMNVDNHGGLDEDYNFRTFGQSMILLFMLSTSSGWDAVLDGITNEEGCDKPNLEMGITGS
XP_025206085.1_X1_Msacchari    GAKGIRTLLFALAMSLPALFNICLLLFLVMFIFAIFGMSFFMNVDSHGGLDDDYNFRTFGQSMILLFMLSTSSGWDAVLDGITNEENCDKPNLEMGITGS
XP_025206086.1_X2_Msacchari    GAKGIRTLLFALAMSLPALFNICLLLFLVMFIFAIFGMSFFMNVDSHGGLDDDYNFRTFGQSMILLFMLSTSSGWDAVLDGITNEENCDKPNLEMGITGS
XP_025206087.1_X3_Msacchari    GAKGIRTLLFALAMSLPALFNICLLLFLVMFIFAIFGMSFFMNVDSHGGLDDDYNFRTFGQSMILLFMLSTSSGWDAVLDGITNEENCDKPNLEMGITGS
XP_025206088.1_X4_Msacchari    GAKGIRTLLFALAMSLPALFNICLLLFLVMFIFAIFGMSFFMNVDSHGGLDDDYNFRTFGQSMILLFMLSTSSGWDAVLDGITNEENCDKPNLEMGITGS
XP_022174241.1_X1_Mpersicae    GAKGIRTLLFALAMSLPALFNICLLLFLVMFIFAIFGMSFFMNVDSHGGLDDDYNFRTFGQSMILLFMLSTSSGWDAVLDGITNEEDCDKPNLEMGITGS
XP_022174242.1_X2_Mpersicae    GAKGIRTLLFALAMSLPALFNICLLLFLVMFIFAIFGMSFFMNVDSHGGLDDDYNFRTFGQSMILLFMLSTSSGWDAVLDGITNEEDCDKPNLEMGITGS
XP_022174243.1_X3_Mpersicae    GAKGIRTLLFALAMSLPALFNICLLLFLVMFIFAIFGMSFFMNVDSHGGLDDDYNFRTFGQSMILLFMLSTSSGWDAVLDGITNEEDCDKPNLEMGITGS
XP_026806613.1_X1_Rmaidis      GAKGIRTLLFALAMSLPALFNICLLLFLVMFIFAIFGMSFFMNVDSHGGLDEDYNFRTFGQSMILLFMLSTSSGWDAVLDGITNEENCQKPNLEMGITGS
XP_026806614.1_X2_Rmaidis      GAKGIRTLLFALAMSLPALFNICLLLFLVMFIFAIFGMSFFMNVDSHGGLDEDYNFRTFGQSMILLFMLSTSSGWDAVLDGITNEENCQKPNLEMGITGS
XP_026806615.1_X3_Rmaidis      GAKGIRTLLFALAMSLPALFNICLLLFLVMFIFAIFGMSFFMNVDSHGGLDEDYNFRTFGQSMILLFMLSTSSGWDAVLDGITNEENCQKPNLEMGITGS
XP_026806616.1_X4_Rmaidis      GAKGIRTLLFALAMSLPALFNICLLLFLVMFIFAIFGMSFFMNVDSHGGLDEDYNFRTFGQSMILLFMLSTSSGWDAVLDGITNEENCQKPNLEMGITGS
XP_015366158.1_X1_Dnoxia       GAKGIRTLLFALAMSLPALFNICLLLFLVMFIFAIFGMSFFMNVDSHGGLDDDYNFRTFGQSMILLFMLSTSSGWDAVLDGITNEEDCDKPNLEMGITGS
XP_015366159.1_X2_Dnoxia       GAKGIRTLLFALAMSLPALFNICLLLFLVMFIFAIFGMSFFMNVDSHGGLDDDYNFRTFGQSMILLFMLSTSSGWDAVLDGITNEEDCDKPNLEMGITGS
XP_025407713.1_X1_Sflava       GAKGIRTLLFALAMSLPALFNICLLLFLVMFIFAIFGMSFFMNVDSHGGLDQDYNFRTFGQSMILLFMLSTSSGWDAVLDGITNEENCEKPNLEMGITGS
XP_025407714.1_X2_Sflava       GAKGIRTLLFALAMSLPALFNICLLLFLVMFIFAIFGMSFFMNVDSHGGLDQDYNFRTFGQSMILLFMLSTSSGWDAVLDGITNEENCEKPNLEMGITGS
```

```
                              .........DIV S6..........
                          801         .         .         .         .         :         .         .         .         .         9 900
MW759899.1_X4_Aglycines      CGSSAVGTAFLLSYLVINFLIVINMYIAVILENYSQATEDVQEGLTDDDYDMYYEIWQHFDPNGTRYIRYDQLSDFLDILEPPLKIHKPNKYKIVSMDIP
QTJ01841.1_X1_Aglycines      CGSSAVGTAFLLSYLVINFLIVINMYIAVILENYSQATEDVQEGLTDDDYDMYYEIWQHFDPNGTRYIRYDQLSDFLDILEPPLKIHKPNKYKIVSMDIP
QTJ01842.1_X2_Aglycines      CGSSAVGTAFLLSYLVINFLIVINMYIAVILENYSQATEDVQEGLTDDDYDMYYEIWQHFDPNGTRYIRYDQLSDFLDILEPPLKIHKPNKYKIVSMDIP
QTJ01843.1_X3_Aglycines      CGSSAVGTAFLLSYLVINFLIVINMYIAVILENYSQATEDVQEGLTDDDYDMYYEIWQHFDPNGTRYIRYDQLSDFLDILEPPLKIHKPNKYKIVSMDIP
XP_001949648.2_X1_Apisum     CGSSAVGTAFLLSYLVINFLIVINMYIAVILENYSQATEDVQEGLTDDDYDMYYEIWQHFDPDGTRYIRYDQLSDFLDILEPPLKIHKPNKYKIVSMDIP
XP_008183361.1_X2_Apisum     CGSSAVGTAFLLSYLVINFLIVINMYIAVILENYSQATEDVQEGLTDDDYDMYYEIWQHFDPDGTRYIRYDQLSDFLDILEPPLKIHKPNKYKIVSMDIP
XP_029344986.1_X3_Apisum     CGSSAVGTAFLLSYLVINFLIVINMYIAVILENYSQATEDVQEGLTDDDYDMYYEIWQHFDPDGTRYIRYDQLSDFLDILEPPLKIHKPNKYKIVSMDIP
XP_027840054.1_X1_Agossypii  CGSSAVGTAFLLSYLVINFLIVINMYIAVILENYSQATEDVQEGLTDDDYDMYYEIWQHFDPNGTRYIRYDQLSDFLDILEPPLKIHKPNKYKIVSMDIP
XP_027840055.1_X2_Agossypii  CGSSAVGTAFLLSYLVINFLIVINMYIAVILENYSQATEDVQEGLTDDDYDMYYEIWQHFDPNGTRYIRYDQLSDFLDILEPPLKIHKPNKYKIVSMDIP
XP_025206085.1_X1_Msacchari  CGSSAVGTAFLLSYLVINFLIVINMYIAVILENYSQATEDVQEGLTDDDYDMYYEIWQHFDPNGTRYIRYDQLSDFLDILEPPLKIHKPNKYKIVSMDIP
XP_025206086.1_X2_Msacchari  CGSSAVGTAFLLSYLVINFLIVINMYIAVILENYSQATEDVQEGLTDDDYDMYYEIWQHFDPNGTRYIRYDQLSDFLDILEPPLKIHKPNKYKIVSMDIP
XP_025206087.1_X3_Msacchari  CGSSAVGTAFLLSYLVINFLIVINMYIAVILENYSQATEDVQEGLTDDDYDMYYEIWQHFDPNGTRYIRYDQLSDFLDILEPPLKIHKPNKYKIVSMDIP
XP_025206088.1_X4_Msacchari  CGSSAVGTAFLLSYLVINFLIVINMYIAVILENYSQATEDVQEGLTDDDYDMYYEIWQHFDPNGTRYIRYDQLSDFLDILEPPLKIHKPNKYKIVSMDIP
XP_022174241.1_X1_Mpersicae  CGSSAVGTAFLLSYLVINFLIVINMYIAVILENYSQATEDVQEGLTDDDYDMYYEIWQHFDPNGTRYIRYDQLSDFLDILEPPLKIHKPNKYKIVSMDIP
XP_022174242.1_X2_Mpersicae  CGSSAVGTAFLLSYLVINFLIVINMYIAVILENYSQATEDVQEGLTDDDYDMYYEIWQHFDPNGTRYIRYDQLSDFLDILEPPLKIHKPNKYKIVSMDIP
XP_022174243.1_X3_Mpersicae  CGSSAVGTAFLLSYLVINFLIVINMYIAVILENYSQATEDVQEGLTDDDYDMYYEIWQHFDPNGTRYIRYDQLSDFLDILEPPLKIHKPNKYKIVSMDIP
XP_026806613.1_X1_Rmaidis    CGSSAVGTAFLLSYLVINFLIVINMYIAVILENYSQATEDVQEGLTDDDYDMYYEIWQHFDPNGTRYIRYDQLSDFLDILEPPLKIHKPNKYKIVSMDIP
XP_026806614.1_X2_Rmaidis    CGSSAVGTAFLLSYLVINFLIVINMYIAVILENYSQATEDVQEGLTDDDYDMYYEIWQHFDPNGTRYIRYDQLSDFLDILEPPLKIHKPNKYKIVSMDIP
XP_026806615.1_X3_Rmaidis    CGSSAVGTAFLLSYLVINFLIVINMYIAVILENYSQATEDVQEGLTDDDYDMYYEIWQHFDPNGTRYIRYDQLSDFLDILEPPLKIHKPNKYKIVSMDIP
XP_026806616.1_X4_Rmaidis    CGSSAVGTAFLLSYLVINFLIVINMYIAVILENYSQATEDVQEGLTDDDYDMYYEIWQHFDPNGTRYIRYDQLSDFLDILEPPLKIHKPNKYKIVSMDIP
XP_015366158.1_X1_Dnoxia     CGSSAVGTAFLLSYLVINFLIVINMYIAVILENYSQATEDVQEGLTDDDYDMYYEIWQHFDPNGTRYIRYDQLSDFLDILEPPLKIHKPNKYKIVSMDIP
XP_015366159.1_X2_Dnoxia     CGSSAVGTAFLLSYLVINFLIVINMYIAVILENYSQATEDVQEGLTDDDYDMYYEIWQHFDPNGTRYIRYDQLSDFLDILEPPLKIHKPNKYKIVSMDIP
XP_025407713.1_X1_Sflava     CGSTTVGTAFLLSYLVINFLIVINMYIAVILENYSQATEDVQEGLTDDDYDMYYEIWQHFDPDGTRYIRYDQLSDFLDILEPPLKIHKPNKYKIVSMDIP
XP_025407714.1_X2_Sflava     CGSTTVGTAFLLSYLVINFLIVINMYIAVILENYSQATEDVQEGLTDDDYDMYYEIWQHFDPDGTRYIRYDQLSDFLDILEPPLKIHKPNKYKIVSMDIP
```

```
                              901        .         .         .         .         :         .         .         .         .         0 1000
MW759899.1_X4_Aglycines       ICKGDLIYCVDILDALTKDFFARKGNPIIETVAEIGEMQTRPEEAGYEPISSSLWRMREVYCATIIQSSWRKYAAAAKQQTADDDRSDGAASPDGRETAV
QTJ01841.1_X1_Aglycines       ICKGDLIYCVDILDALTKDFFARKGNPIIETVAEIGEMQTRPEEAGYEPISSSLWRMREVYCATIIQSSWRKYAAAAKQQTADDDRSDGAASPDGRETAV
QTJ01842.1_X2_Aglycines       ICKGDLIYCVDILDALTKDFFARKGNPIIETVAEIGEMQTRPEEAGYEPISSSLWRMREVYCATIIQSSWRKYAAAAKQQTADDDRSDGAASPDGRETAV
QTJ01843.1_X3_Aglycines       ICKGDLIYCVDILDALTKDFFARKGNPIIETVAEIGEMQTRPEEAGYEPISSSLWRMREVYCATIIQSSWRKYAAAAKQQTADDDRSDGAASPDGRETAV
XP_001949648.2_X1_Apisum      ICKGDLIYCVDILDALTKDFFARKGNPIIETVAEIGEMQTRPEEAGYEPISSSLWRMREVYCAIIIQNSWRKYTKAAKEQTADDDRSDGAASPDGRETAV
XP_008183361.1_X2_Apisum      ICKGDLIYCVDILDALTKDFFARKGNPIIETVAEIGEMQTRPEEAGYEPISSSLWRMREVYCAIIIQNSWRKYTKAAKEQTADDDRSDGAASPDGRETAV
XP_029344986.1_X3_Apisum      ICKGDLIYCVDILDALTKDFFARKGNPIIETVAEIGEMQTRPEEAGYEPISSSLWRMREVYCAIIIQNSWRKYTKAAKEQTADDDRSDGAASPDGRETAV
XP_027840054.1_X1_Agossypii   ICKGDLIYCVDILDALTKDFFARKGNPIIETVAEIGEMQTRPEEAGYEPISSSLWRMREVYCATIIQSSWRKYAAAAKQQTADDDRSDGAASPDGRETAV
XP_027840055.1_X2_Agossypii   ICKGDLIYCVDILDALTKDFFARKGNPIIETVAEIGEMQTRPEEAGYEPISSSLWRMREVYCATIIQSSWRKYAAAAKQQTADDDRSDGAASPDGRETAV
XP_025206085.1_X1_Msacchari   ICKGDLIYCVDILDALTKDFFARKGNPIIETVAEIGEMQTRPEEADYEPISSSLWRMREVYCAMIIQKSWRKYVAAAKQKSADEDRSDGAASPDGRETAV
XP_025206086.1_X2_Msacchari   ICKGDLIYCVDILDALTKDFFARKGNPIIETVAEIGEMQTRPEEADYEPISSSLWRMREVYCAMIIQKSWRKYVAAAKQKSADEDRSDGAASPDGRETAV
XP_025206087.1_X3_Msacchari   ICKGDLIYCVDILDALTKDFFARKGNPIIETVAEIGEMQTRPEEADYEPISSSLWRMREVYCAMIIQKSWRKYVAAAKQKSADEDRSDGAASPDGRETAV
XP_025206088.1_X4_Msacchari   ICKGDLIYCVDILDALTKDFFARKGNPIIETVAEIGEMQTRPEEADYEPISSSLWRMREVYCAMIIQKSWRKYVAAAKQKSADEDRSDGAASPDGRETAV
XP_022174241.1_X1_Mpersicae   ICKGDLIYCVDILDALTKDFFARKGNPIIETVAEIGEMQTRPEEAGYEPISSSLWRMREVYCATIIQSSWRKYTMAAKQQTADDDRSDGAASPDGRETAV
XP_022174242.1_X2_Mpersicae   ICKGDLIYCVDILDALTKDFFARKGNPIIETVAEIGEMQTRPEEAGYEPISSSLWRMREVYCATIIQSSWRKYTMAAKQQTADDDRSDGAASPDGRETAV
XP_022174243.1_X3_Mpersicae   ICKGDLIYCVDILDALTKDFFARKGNPIIETVAEIGEMQTRPEEAGYEPISSSLWRMREVYCATIIQSSWRKYTMAAKQQTADDDRSDGAASPDGRETAV
XP_026806613.1_X1_Rmaidis     ICKGDLIYCVDILDALTKDFFARKGNPIIETVAEIGEMQTRPEEAGYEPISSSLWRMREVYCAIIIQNSWRKYRAAAKQQTADDERSDGAASPDGRETAV
XP_026806614.1_X2_Rmaidis     ICKGDLIYCVDILDALTKDFFARKGNPIIETVAEIGEMQTRPEEAGYEPISSSLWRMREVYCAIIIQNSWRKYRAAAKQQTADDERSDGAASPDGRETAV
XP_026806615.1_X3_Rmaidis     ICKGDLIYCVDILDALTKDFFARKGNPIIETVAEIGEMQTRPEEAGYEPISSSLWRMREVYCAIIIQNSWRKYRAAAKQQTADDERSDGAASPDGRETAV
XP_026806616.1_X4_Rmaidis     ICKGDLIYCVDILDALTKDFFARKGNPIIETVAEIGEMQTRPEEAGYEPISSSLWRMREVYCAIIIQNSWRKYRAAAKQQTADDERSDGAASPDGRETAV
XP_015366158.1_X1_Dnoxia      ICKGDLIYCVDILDALTKDFFARKGNPIIETVAEIGEMQTRPEEAGYEPISSSLWRMREVYCAIIIQKAWRKYVMAAKQQTAEDDRSDGAASPDGRETAV
XP_015366159.1_X2_Dnoxia      ICKGDLIYCVDILDALTKDFFARKGNPIIETVAEIGEMQTRPEEAGYEPISSSLWRMREVYCAIIIQKAWRKYVMAAKQQTAEDDRSDGAASPDGRETAV
XP_025407713.1_X1_Sflava      ICKGDLIYCVDILDALTKDFFARKGNPIIETVAEIGEIQTRPEEAGYEPISSTLWRMREVYCAIIIQSAWRKFARAAKEQTADEDRSDGAASPDGRETAV
XP_025407714.1_X2_Sflava      ICKGDLIYCVDILDALTKDFFARKGNPIIETVAEIGEIQTRPEEAGYEPISSTLWRMREVYCAIIIQSAWRKFARAAKEQTADEDRSDGAASPDGRETAV
```

```
                              1001          .         .         .] 1031
QTJ01841.1_X1_Aglycines       LVESDGFVTKNGHKVVIHSRSSSKSSRLADV
QTJ01842.1_X2_Aglycines       LVESDGFVTKNGHKVVIHSRSSSKSSRLADV
QTJ01843.1_X3_Aglycines       LVESDGFVTKNGHKVVIHSRSSSKSSRLADV
MW759899.1_X4_Aglycines       LVESDGFVTKNGHKVVIHSRSSSKSSRLADV
XP_001949648.2_X1_Apisum      LVESDGFVTKNGHKVVIHSRSSSKSSRLADV
XP_008183361.1_X2_Apisum      LVESDGFVTKNGHKVVIHSRSSSKSSRLADV
XP_029344986.1_X3_Apisum      LVESDGFVTKNGHKVVIHSRSSSKSSRLADV
XP_027840054.1_X1_Agossypii   LVESDGFVTKNGHKVVIHSRSSSKSSRLADV
XP_027840055.1_X2_Agossypii   LVESDGFVTKNGHKVVIHSRSSSKSSRLADV
XP_025206085.1_X1_Msacchari   LVESDGFVTKNGHKVVIHSRSSSKSSRLADV
XP_025206086.1_X2_Msacchari   LVESDGFVTKNGHKVVIHSRSSSKSSRLADV
XP_025206087.1_X3_Msacchari   LVESDGFVTKNGHKVVIHSRSSSKSSRLADV
XP_025206088.1_X4_Msacchari   LVESDGFVTKNGHKVVIHSRSSSKSSRLADV
XP_022174241.1_X1_Mpersicae   LVESDGFVTKNGHKVVIHSRSSSKSSRLADV
XP_022174242.1_X2_Mpersicae   LVESDGFVTKNGHKVVIHSRSSSKSSRLADV
XP_022174243.1_X3_Mpersicae   LVESDGFVTKNGHKVVIHSRSSSKSSRLADV
XP_026806613.1_X1_Rmaidis     LVESDGFVTKNGHKVVIHSRSSSKSSRLADV
XP_026806614.1_X2_Rmaidis     LVESDGFVTKNGHKVVIHSRSSSKSSRLADV
XP_026806615.1_X3_Rmaidis     LVESDGFVTKNGHKVVIHSRSSSKSSRLADV
XP_026806616.1_X4_Rmaidis     LVESDGFVTKNGHKVVIHSRSSSKSSRLADV
XP_015366158.1_X1_Dnoxia      LVESDGFVTKNGHKVVIHSRSSSKSSRLADV
XP_015366159.1_X2_Dnoxia      LVESDGFVTKNGHKVVIHSRSSSKSSRLADV
XP_025407713.1_X1_Sflava      LVESDGFVTKNGHKVVIHSRSSSKSSRLADV
XP_025407714.1_X2_Sflava      LVESDGFVTKNGHKVVIHSRSSSKSSRLADV
```

Figure S7: Partial alignment of Sanger sequence fragments from the *Aphis glycines* voltage-gated sodium channel subunit 1 (*vgsc-h1*) gene amplified using primers DIIS4-S6-F and -R among clones resistant (RES) field collections and susceptible laboratory colonies [SUS; biotypes 1, 3 and 4 from Iowa State University (ISU) or University of Illinois (UIL)]. **A)** A guanine (G) to adenosine (A) transition at AG6007485-RA position 2784 leads to a putative nonsynonymous methionine (M) to isoleucine (I) mutation at translated protein position 918 (M918I), and **B)** *kdr* mutation derived from a cytosine (C) to thymidine (T) transition at gene model AG6007485-RA position 3070 results in a putative leucine (L) to phenylalanine (P) change a protein position 1014 (L1014F). Putative heterozygous *A. glycines* genotypes predicted from Sanger sequences with co-occurring G and A nucleotides at alignment position 87 represented at an R, and co-occurrence of T and C nucleotides at alignment position 441 indicated as a Y. Aligned sequences named by GenBank accession:clone. Intron sequence is in small caps and canonical 5'-gt splice junction underlined, and genomic intron sequence gaps with aligned cDNA in asterisks (*). Positions of other mutations, L935M and T939S associated with pyrethroid resistance in other arthropods (Dong et al. 2014) are encodes in boxes. The L935M mutation is associated with resistance in *A. glycines* at two of eight locations from Minnesota (Paula et al., 2021), but not detected in this study.

A)

```
                                                     M918I                L935M        T939S
                            T  L  N  L  L  I  S  I  MI  G  R  T  I  G  A  L  G  N  L  T
AG6007485.1          2758   ACACTTAATCTTTTAATATCAATAATGGGTCGAACCATTGGTGCTTTGGGTAACCTAACG   2817
MW846869:ISU-B1_1      61   ACACTTAATCTTTTAATATCAATAATGGGTCGAACCATTGGTGCTTTGGGTAACCTAACG   120
MW846870:ISU-B1_5      61   ACACTTAATCTTTTAATATCAATAATGGGTCGAACCATTGGTGCTTTGGGTAACCTAACG   120
MW846871:ISU-B1_6      61   ACACTTAATCTTTTAATATCAATAATGGGTCGAACCATTGGTGCTTTGGGTAACCTAACG   120
MW846872:ISU-B1_17     61   ACACTTAATCTTTTAATATCAATAATGGGTCGAACCATTGGTGCTTTGGGTAACCTAACG   120
MW846873:ISU-B1_22     61   ACACTTAATCTTTTAATATCAATAATGGGTCGAACCATTGGTGCTTTGGGTAACCTAACG   120
MW846874:ISU-B3_4      61   ACACTTAATCTTTTAATATCAATAATGGGTCGAACCATTGGTGCTTTGGGTAACCTAACG   120
MW846875:ISU-B3_6      61   ACACTTAATCTTTTAATATCAATAATGGGTCGAACCATTGGTGCTTTGGGTAACCTAACG   120
MW846876:ISU-B3_8      61   ACACTTAATCTTTTAATATCAATAATGGGTCGAACCATTGGTGCTTTGGGTAACCTAACG   120
MW846877:ISU-B3_14     61   ACACTTAATCTTTTAATATCAATAATGGGTCGAACCATTGGTGCTTTGGGTAACCTAACG   120
MW846878:ISU-B3_15     61   ACACTTAATCTTTTAATATCAATAATGGGTCGAACCATTGGTGCTTTGGGTAACCTAACG   120
MW846879:ISU-B3_17     61   ACACTTAATCTTTTAATATCAATAATGGGTCGAACCATTGGTGCTTTGGGTAACCTAACG   120
MW846880:ISU-B3_21     61   ACACTTAATCTTTTAATATCAATAATGGGTCGAACCATTGGTGCTTTGGGTAACCTAACG   120
MW846881:ISU-B3_24     61   ACACTTAATCTTTTAATATCAATAATGGGTCGAACCATTGGTGCTTTGGGTAACCTAACG   120
MW846882:ISU-B4_5      61   ACACTTAATCTTTTAATATCAATAATGGGTCGAACCATTGGTGCTTTGGGTAACCTAACG   120
MW846883:ISU-B4_8      61   ACACTTAATCTTTTAATATCAATAATGGGTCGAACCATTGGTGCTTTGGGTAACCTAACG   120
MW846884:ISU-B4_13     61   ACACTTAATCTTTTAATATCAATAATGGGTCGAACCATTGGTGCTTTGGGTAACCTAACG   120
MW846885:ISU-B4_16     61   ACACTTAATCTTTTAATATCAATAATGGGTCGAACCATTGGTGCTTTGGGTAACCTAACG   120
MW846886:ISU-B4_17     61   ACACTTAATCTTTTAATATCAATAATGGGTCGAACCATTGGTGCTTTGGGTAACCTAACG   120
MW846887:ISU-B4_18     61   ACACTTAATCTTTTAATATCAATAATGGGTCGAACCATTGGTGCTTTGGGTAACCTAACG   120
MW846888:ISU-B4_19     61   ACACTTAATCTTTTAATATCAATAATGGGTCGAACCATTGGTGCTTTGGGTAACCTAACG   120
MW846889:ISU-B4_20     61   ACACTTAATCTTTTAATATCAATAATGGGTCGAACCATTGGTGCTTTGGGTAACCTAACG   120
MW846890:UIL-B1_2      61   ACACTTAATCTTTTAATATCAATAATGGGTCGAACCATTGGTGCTTTGGGTAACCTAACG   120
MW846891:UIL-B1_5      61   ACACTTAATCTTTTAATATCAATAATGGGTCGAACCATTGGTGCTTTGGGTAACCTAACG   120
MW846892:UIL-B1_10     61   ACACTTAATCTTTTAATATCAATAATGGGTCGAACCATTGGTGCTTTGGGTAACCTAACG   120
MW846893:UIL-B1_19     61   ACACTTAATCTTTTAATATCAATAATGGGTCGAACCATTGGTGCTTTGGGTAACCTAACG   120
MW846895:UIL-B1_21     61   ACACTTAATCTTTTAATATCAATAATGGGTCGAACCATTGGTGCTTTGGGTAACCTAACG   120
MW846896:UIL-B1_23     61   ACACTTAATCTTTTAATATCAATAATGGGTCGAACCATTGGTGCTTTGGGTAACCTAACG   120
MW846897:UIL-B1_24     61   ACACTTAATCTTTTAATATCAATAATGGGTCGAACCATTGGTGCTTTGGGTAACCTAACG   120
MW846902:UIL-B1_20     61   ACACTTAATCTTTTAATATCAATAATGGGTCGAACCATTGGTGCTTTGGGTAACCTAACG   120
MW846921:9R1A-1        61   ACACTTAATCTTTTAATATCAATAATRGGTCGAACCATTGGTGCTTTGGGTAACCTAACG   120
MW846922:9R2A-1        61   ACACTTAATCTTTTAATATCAATAATRGGTCGAACCATTGGTGCTTTGGGTAACCTAACG   120
MW846923:9R3A-1        61   ACACTTAATCTTTTAATATCAATAATGGGTCGAACCATTGGTGCTTTGGGTAACCTAACG   120
MW846924:9R3A-2        61   ACACTTAATCTTTTAATATCAATAATGGGTCGAACCATTGGTGCTTTGGGTAACCTAACG   120
MW846925:9R1D-1        61   ACACTTAATCTTTTAATATCAATAATGGGTCGAACCATTGGTGCTTTGGGTAACCTAACG   120
MW846926:9R1D-2        61   ACACTTAATCTTTTAATATCAATAATRGGTCGAACCATTGGTGCTTTGGGTAACCTAACG   120
MW846927:9R1D-3        61   ACACTTAATCTTTTAATATCAATAATGGGTCGAACCATTGGTGCTTTGGGTAACCTAACG   120
MW846928:9R1D-5        61   ACACTTAATCTTTTAATATCAATAATGGGTCGAACCATTGGTGCTTTGGGTAACCTAACG   120
MW846929:MN1-1-1       61   ACACTTAATCTTTTAATATCAATAATGGGTCGAACCATTGGTGCTTTGGGTAACCTAACG   120
MW846930:MN1-1-2       61   ACACTTAATCTTTTAATATCAATAATGGGTCGAACCATTGGTGCTTTGGGTAACCTAACG   120
MW846931:MN1-1-3       61   ACACTTAATCTTTTAATATCAATAATGGGTCGAACCATTGGTGCTTTGGGTAACCTAACG   120
MW846932:MN1-1-4       61   ACACTTAATCTTTTAATATCAATAATGGGTCGAACCATTGGTGCTTTGGGTAACCTAACG   120
MW846933:MN1-2-1       61   ACACTTAATCTTTTAATATCAATAATRGGTCGAACCATTGGTGCTTTGGGTAACCTAACG   120
MW846934:MN1-2-2       61   ACACTTAATCTTTTAATATCAATAATGGGTCGAACCATTGGTGCTTTGGGTAACCTAACG   120
MW846935:MN1-2-3       61   ACACTTAATCTTTTAATATCAATAATGGGTCGAACCATTGGTGCTTTGGGTAACCTAACG   120
MW846936:MN1-2-4       61   ACACTTAATCTTTTAATATCAATAATGGGTCGAACCATTGGTGCTTTGGGTAACCTAACG   120
MW846937:MN2-1-1       61   ACACTTAATCTTTTAATATCAATAATGGGTCGAACCATTGGTGCTTTGGGTAACCTAACG   120
MW846938:MN2-1-2       61   ACACTTAATCTTTTAATATCAATAATGGGTCGAACCATTGGTGCTTTGGGTAACCTAACG   120
MW846939:MN2-1-3       61   ACACTTAATCTTTTAATATCAATAATGGGTCGAACCATTGGTGCTTTGGGTAACCTAACG   120
MW846940:MN2-2-1       61   ACACTTAATCTTTTAATATCAATAATGGGTCGAACCATTGGTGCTTTGGGTAACCTAACG   120
MW846941:MN2-2-2       61   ACACTTAATCTTTTAATATCAATAATRGGTCGAACCATTGGTGCTTTGGGTAACCTAACG   120
MW846942:MN2-2-3       61   ACACTTAATCTTTTAATATCAATAATGGGTCGAACCATTGGTGCTTTGGGTAACCTAACG   120
MW846943:MN2-2-4       61   ACACTTAATCTTTTAATATCAATAATRGGTCGAACCATTGGTGCTTTGGGTAACCTAACG   120
MW846944:Nashua-1      61   ACACTTAATCTTTTAATATCAATAATGGGTCGAACCATTGGTGCTTTGGGTAACCTAACG   120
MW846945:Nashua-2      61   ACACTTAATCTTTTAATATCAATAATRGGTCGAACCATTGGTGCTTTGGGTAACCTAACG   120
MW846946:Nashua-3      61   ACACTTAATCTTTTAATATCAATAATRGGTCGAACCATTGGTGCTTTGGGTAACCTAACG   120
MW846947:Suth-4        61   ACACTTAATCTTTTAATATCAATAATGGGTCGAACCATTGGTGCTTTGGGTAACCTAACG   120
MW846948:Nashua-5      61   ACACTTAATCTTTTAATATCAATAATGGGTCGAACCATTGGTGCTTTGGGTAACCTAACG   120
MW846949:Nashua-7      61   ACACTTAATCTTTTAATATCAATAATRGGTCGAACCATTGGTGCTTTGGGTAACCTAACG   120
MW846950:Nashua-8      61   ACACTTAATCTTTTAATATCAATAATGGGTCGAACCATTGGTGCTTTGGGTAACCTAACG   120
MW846951:Suth-1        61   ACACTTAATCTTTTAATATCAATAATRGGTCGAACCATTGGTGCTTTGGGTAACCTAACG   120
MW846952:Suth-2        61   ACACTTAATCTTTTAATATCAATAATRGGTCGAACCATTGGTGCTTTGGGTAACCTAACG   120
MW846953:Suth-3        61   ACACTTAATCTTTTAATATCAATAATRGGTCGAACCATTGGTGCTTTGGGTAACCTAACG   120
MW846954:Nashua-4      61   ACACTTAATCTTTTAATATCAATAATRGGTCGAACCATTGGTGCTTTGGGTAACCTAACG   120
MW846955:Suth-5        61   ACACTTAATCTTTTAATATCAATAATRGGTCGAACCATTGGTGCTTTGGGTAACCTAACG   120
MW846956:Suth-6        61   ACACTTAATCTTTTAATATCAATAATRGGTCGAACCATTGGTGCTTTGGGTAACCTAACG   120
MW846957:Suth-7        61   ACACTTAATCTTTTAATATCAATAATRGGTCGAACCATTGGTGCTTTGGGTAACCTAACG   120
MW846958:Suth-8        61   ACACTTAATCTTTTAATATCAATAATRGGTCGAACCATTGGTGCTTTGGGTAACCTAACG   120
```

Susceptible (SUS) laboratory colonies

Resistant (RES) field collections

B)

```
                                   .........DII S5 .........
                                     T  V  V  I  G  N  LF V |............. intron ............>
AG6007485.1           3051   TACTGTTGTCATCGGTAACCTTGTG***********************************    3076
MW846869:ISU-B1_1      421   TACTGTTGTCATCGGTAACCTTGTGgtatgtataagtactgacaatgcataaatgtgtat     480
MW846870:ISU-B1_5      421   TACTGTTGTCATCGGTAACCTTGTGgtatgtataagtactgacaatgcataaatgtgtat     480
MW846871:ISU-B1_6      421   TACTGTTGTCATCGGTAACCTTGTGgtatgtataagtactgacaatgcataaatgtgtat     480
MW846872:ISU-B1_17     421   TACTGTTGTCATCGGTAACCTTGTGgtatgtataagtactgacaatgcataaatgtgtat     480
MW846873:ISU-B1_22     421   TACTGTTGTCATCGGTAACCTTGTGgtatgtataagtactgacaatgcataaatgtgtat     480
MW846874:ISU-B3_4      421   TACTGTTGTCATCGGTAACCTTGTGgtatgtataagtactgacaatgcataaatgtgtat     480
MW846875:ISU-B3_6      421   TACTGTTGTCATCGGTAACCTTGTGgtatgtataagtactgacaatgcataaatgtgtat     480
MW846876:ISU-B3_8      421   TACTGTTGTCATCGGTAACCTTGTGgtatgtataagtactgacaatgcataaatgtgtat     480
MW846877:ISU-B3_14     421   TACTGTTGTCATCGGTAACCTTGTGgtatgtataagtactgacaatgcataaatgtgtat     480
MW846878:ISU-B3_15     421   TACTGTTGTCATCGGTAACCTTGTGgtatgtataagtactgacaatgcataaatgtgtat     480
MW846879:ISU-B3_17     421   TACTGTTGTCATCGGTAACCTTGTGgtatgtataagtactgacaatgcataaatgtgtat     480
MW846880:ISU-B3_21     421   TACTGTTGTCATCGGTAACCTTGTGgtatgtataagtactgacaatgcataaatgtgtat     480
MW846881:ISU-B3_24     421   TACTGTTGTCATCGGTAACCTTGTGgtatgtataagtactgacaatgcataaatgtgtat     480
MW846882:ISU-B4_5      421   TACTGTTGTCATCGGTAACCTTGTGgtatgtataagtactgacaatgcataaatgtgtat     480
MW846883:ISU-B4_8      421   TACTGTTGTCATCGGTAACCTTGTGgtatgtataagtactgacaatgcataaatgtgtat     480
MW846884:ISU-B4_13     421   TACTGTTGTCATCGGTAACCTTGTGgtatgtataagtactgacaatgcataaatgtgtat     480
MW846885:ISU-B4_16     421   TACTGTTGTCATCGGTAACCTTGTGgtatgtataagtactgacaatgcataaatgtgtat     480
MW846886:ISU-B4_17     421   TACTGTTGTCATCGGTAACCTTGTGgtatgtataagtactgacaatgcataaatgtgtat     480
MW846887:ISU-B4_18     421   TACTGTTGTCATCGGTAACCTTGTGgtatgtataagtactgacaatgcataaatgtgtat     480
MW846888:ISU-B4_19     421   TACTGTTGTCATCGGTAACCTTGTGgtatgtataagtactgacaatgcataaatgtgtat     480
MW846889:ISU-B4_20     421   TACTGTTGTCATCGGTAACCTTGTGgtatgtataagtactgacaatgcataaatgtgtat     480
MW846890:UIL-B1_2      421   TACTGTTGTCATCGGTAACCTTGTGgtatgtataagtactgacaatgcataaatgtgtat     480
MW846891:UIL-B1_5      421   TACTGTTGTCATCGGTAACCTTGTGgtatgtataagtactgacaatgcataaatgtgtat     480
MW846892:UIL-B1_10     421   TACTGTTGTCATCGGTAACCTTGTGgtatgtataagtactgacaatgcataaatgtgtat     480
MW846893:UIL-B1_19     421   TACTGTTGTCATCGGTAACCTTGTGgtatgtataagtactgacaatgcataaatgtgtat     480
MW846895:UIL-B1_21     421   TACTGTTGTCATCGGTAACCTTGTGgtatgtataagtactgacaatgcataaatgtgtat     480
MW846896:UIL-B1_23     421   TACTGTTGTCATCGGTAACCTTGTGgtatgtataagtactgacaatgcataaatgtgtat     480
MW846897:UIL-B1_24     421   TACTGTTGTCATCGGTAACCTTGTGgtatgtataagtactgacaatgcataaatgtgtat     480
MW846902:UIL-B1_20     421   TACTGTTGTCATCGGTAACCTTGTGgtatgtataagtactgacaatgcataaatgtgtat     480
MW846921:9R1A-1        421   TACTGTTGTCATCGGTAACTTTGTGgtatgtataagtactgacaatgcataaatgtgtat     480
MW846922:9R2A-1        421   TACTGTTGTCATCGGTAACYTTGTGgtatgtataagtactgacaatgcataaatgtgtat     480
MW846923:9R3A-1        421   TACTGTTGTCATCGGTAACYTTGTGgtatgtataagtactgacaatgcataaatgtgtat     480
MW846924:9R3A-2        421   TACTGTTGTCATCGGTAACTTTGTGgtatgtataagtactgacaatgcataaatgtgtat     480
MW846925:9R1D-1        421   TACTGTTGTCATCGGTAACTTTGTGgtatgtataagtactgacaatgcataaatgtgtat     480
MW846926:9R1D-2        421   TACTGTTGTCATCGGTAACYTTGTGgtatgtataagtactgacaatgcataaatgtgtat     480
MW846927:9R1D-3        421   TACTGTTGTCATCGGTAACYTTGTGgtatgtataagtactgacaatgcataaatgtgtat     480
MW846928:9R1D-5        421   TACTGTTGTCATCGGTAACYTTGTGgtatgtataagtactgacaatgcataaatgtgtat     480
MW846929:MN1-1-1       421   TACTGTTGTCATCGGTAACYTTGTGgtatgtataagtactgacaatgcataaatgtgtat     480
MW846930:MN1-1-2       421   TACTGTTGTCATCGGTAACYTTGTGgtatgtataagtactgacaatgcataaatgtgtat     480
MW846931:MN1-1-3       421   TACTGTTGTCATCGGTAACYTTGTGgtatgtataagtactgacaatgcataaatgtgtat     480
MW846932:MN1-1-4       421   TACTGTTGTCATCGGTAACTTTGTGgtatgtataagtactgacaatgcataaatgtgtat     480
MW846933:MN1-2-1       421   TACTGTTGTCATCGGTAACTTTGTGgtatgtataagtactgacaatgcataaatgtgtat     480
MW846934:MN1-2-2       421   TACTGTTGTCATCGGTAACYTTGTGgtatgtataagtactgacaatgcataaatgtgtat     480
MW846935:MN1-2-3       421   TACTGTTGTCATCGGTAACYTTGTGgtatgtataagtactgacaatgcataaatgtgtat     480
MW846936:MN1-2-4       421   TACTGTTGTCATCGGTAACYTTGTGgtatgtataagtactgacaatgcataaatgtgtat     480
MW846937:MN2-1-1       421   TACTGTTGTCATCGGTAACYTTGTGgtatgtataagtactgacaatgcataaatgtgtat     480
MW846938:MN2-1-2       421   TACTGTTGTCATCGGTAACYTTGTGgtatgtataagtactgacaatgcataaatgtgtat     480
MW846939:MN2-1-3       421   TACTGTTGTCATCGGTAACYTTGTGgtatgtataagtactgacaatgcataaatgtgtat     480
MW846940:MN2-2-1       421   TACTGTTGTCATCGGTAACTTTGTGgtatgtataagtactgacaatgcataaatgtgtat     480
MW846941:MN2-2-2       421   TACTGTTGTCATCGGTAACYTTGTGgtatgtataagtactgacaatgcataaatgtgtat     480
MW846942:MN2-2-3       421   TACTGTTGTCATCGGTAACYTTGTGgtatgtataagtactgacaatgcataaatgtgtat     480
MW846943:MN2-2-4       421   TACTGTTGTCATCGGTAACYTTGTGgtatgtataagtactgacaatgcataaatgtgtat     480
MW846944:Nashua-1      421   TACTGTTGTCATCGGTAACYTTGTGgtatgtataagtactgacaatgcataaatgtgtat     480
MW846945:Nashua-2      421   TACTGTTGTCATCGGTAACYTTGTGgtatgtataagtactgacaatgcataaatgtgtat     480
MW846946:Nashua-3      421   TACTGTTGTCATCGGTAACYTTGTGgtatgtataagtactgacaatgcataaatgtgtat     480
MW846947:Suth-4        421   TACTGTTGTCATCGGTAACYTTGTGgtatgtataagtactgacaatgcataaatgtgtat     480
MW846948:Nashua-5      421   TACTGTTGTCATCGGTAACYTTGTGgtatgtataagtactgacaatgcataaatgtgtat     480
MW846949:Nashua-7      421   TACTGTTGTCATCGGTAACYTTGTGgtatgtataagtactgacaatgcataaatgtgtat     480
MW846950:Nashua-8      421   TACTGTTGTCATCGGTAACYTTGTGgtatgtataagtactgacaatgcataaatgtgtat     480
MW846951:Suth-1        421   TACTGTTGTCATCGGTAACYTTGTGgtatgtataagtactgacaatgcataaatgtgtat     480
MW846952:Suth-2        421   TACTGTTGTCATCGGTAACYTTGTGgtatgtataagtactgacaatgcataaatgtgtat     480
MW846953:Suth-3        421   TACTGTTGTCATCGGTAACYTTGTGgtatgtataagtactgacaatgcataaatgtgtat     480
MW846954:Nashua-4      421   TACTGTTGTCATCGGTAACYTTGTGgtatgtataagtactgacaatgcataaatgtgtat     480
MW846955:Suth-5        421   TACTGTTGTCATCGGTAACYTTGTGgtatgtataagtactgacaatgcataaatgtgtat     480
MW846956:Suth-6        421   TACTGTTGTCATCGGTAACYTTGTGgtatgtataagtactgacaatgcataaatgtgtat     480
MW846957:Suth-7        421   TACTGTTGTCATCGGTAACYTTGTGgtatgtataagtactgacaatgcataaatgtgtat     480
MW846958:Suth-8        421   TACTGTTGTCATCGGTAACYTTGTGgtatgtataagtactgacaatgcataaatgtgtat     480
```

Susceptible (SUS) laboratory colonies (MW846869–MW846902)

Resistant (RES) field collections (MW846921–MW846958)

Figure S8: Alignment of Sanger sequence fragments from the *Aphis glycines* voltage-gated sodium channel subunit 2 (*vgsc-h2*) gene domain III (DIII) segment S6 to DIV S1 (underlined) among individual aphids from resistant (RES) field collections and susceptible laboratory colonies [SUS; biotypes 1, 3 and 4 from Iowa State University (ISU) or University of Illinois (UIL)]. Two synonymous (non-amino acid) changes were predicted positions 161 (G to A transition) and 257 (T to G transversion) of the 414 bp alignment, that result 3rd position changes in glycine codon from GGG to GGA and GGT to GGG, respectively. Putative heterozygous *A. glycines* genotypes predicted from Sanger sequences with co-occurring G and A nucleotides at position 161 represented at an R, and co-occurrence of T and G nucleotides as position 258 (K). No changes were predicted at *A. glycines* amino acids orthologous to *Musca domestica* VGSC positions 1524, 1528, 1538, 1594, or 1553 (encoded in boxes) where mutations M1524I, F1528L, F1538I, D1549V, and E1553G, respectively, were previously associated with pyrethroid resistance in other insect species (Dong et al. 2014). Aligned sequences named by GenBank accession: clone. Intron sequence is in small caps with canonical 5'-gt and 3'-ag splice junctions underlined, and genomic intron sequence gaps with aligned cDNA in asterisks (*).

```
                                  <------------------------------------------- intron ----------------------------------------------
AgVGSC-H2_cDNA                      ****************************************************************************************************
MW847052: ISU-B1_1             1   gggacgcagtagattcaagagacgtaagtatttaaaatgttagaaatacataaaaaaataaaaatatattatataagtatacctataaatgtcttttttt   100
MW847053: ISU-B1_5             1   gggacgcagtagattcaagagacgtaagtatttaaaatgttagaaatacataaaaaaataaaaatatattatataagtatacctataaatgtcttttttt   100
MW847054: ISU-B1_6             1   gggacgcagtagattcaagagacgtaagtatttaaaatgttagaaatacataaaaaaataaaaatatattatataagtatacctataaatgtcttttttt   100
MW847055: ISU-B1_14            1   gggacgcagtagattcaagagacgtaagtatttaaaatgttagaaatacataaaaaaataaaaatatattatataagtatacctataaatgtcttttttt   100
MW847056: UIL-B1_2             1   gggacgcagtagattcaagagacgtaagtatttaaaatgttagaaatacataaaaaaataaaaatatattatataagtatacctataaatgtcttttttt   100
MW847057: UIL-B1_5             1   gggacgcagtagattcaagagacgtaagtatttaaaatgttagaaatacataaaaaaataaaaatatattatataagtatacctataaatgtcttttttt   100
MW847058: UIL-B1_10            1   gggacgcagtagattcaagagacgtaagtatttaaaatgttagaaatacataaaaaaataaaaatatattatataagtatacctataaatgtcttttttt   100
MW847059: UIL-B1_19            1   gggacgcagtagattcaagagacgtaagtatttaaaatgttagaaatacataaaaaaataaaaatatattatataagtatacctataaatgtcttttttt   100
MW847068: ISU-B3_4             1   gggacgcagtagattcaagagacgtaagtatttaaaatgttagaaatacataaaaaaataaaaatatattatataagtatacctataaatgtcttttttt   100
MW847069: ISU-B3_6             1   gggacgcagtagattcaagagacgtaagtatttaaaatgttagaaatacataaaaaaataaaaatatattatataagtatacctataaatgtcttttttt   100
MW847070: ISU-B3_8             1   gggacgcagtagattcaagagacgtaagtatttaaaatgttagaaatacataaaaaaataaaaatatattatataagtatacctataaatgtcttttttt   100
MW847071: ISU-B3_14            1   gggacgcagtagattcaagagacgtaagtatttaaaatgttagaaatacataaaaaaataaaaatatattatataagtatacctataaatgtcttttttt   100
MW847076: ISU-B4_5             1   gggacgcagtagattcaagagacgtaagtatttaaaatgttagaaatacataaaaaaataaaaatatattatataagtatacctataaatgtcttttttt   100
MW847077: ISU-B4_8             1   gggacgcagtagattcaagagacgtaagtatttaaaatgttagaaatacataaaaaaataaaaatatattatataagtatacctataaatgtcttttttt   100
MW847078: ISU-B4_13            1   gggacgcagtagattcaagagacgtaagtatttaaaatgttagaaatacataaaaaaataaaaatatattatataagtatacctataaatgtcttttttt   100
MW847079: ISU-B4_16            1   gggacgcagtagattcaagagacgtaagtatttaaaatgttagaaatacataaaaaaataaaaatatattatataagtatacctataaatgtcttttttt   100
MW847084: Suth-1               1   gggacgcagtagattcaagagacgtaagtatttaaaatgttagaaatacataaaaaaataaaaatatattatataagtatacctataaatgtcttttttt   100
MW847085: Suth-2               1   gggacgcagtagattcaagagacgtaagtatttaaaatgttagaaatacataaaaaaataaaaatatattatataagtatacctataaatgtcttttttt   100
MW847086: Suth-3               1   gggacgcagtagattcaagagacgtaagtatttaaaatgttagaaatacataaaaaaataaaaatatattatataagtatacctataaatgtcttttttt   100
MW847087: Suth-4               1   gggacgcagtagattcaagagacgtaagtatttaaaatgttagaaatacataaaaaaataaaaatatattatataagtatacctataaatgtcttttttt   100
MW847088: Suth-5               1   gggacgcagtagattcaagagacgtaagtatttaaaatgttagaaatacataaaaaaataaaaatatattatataagtatacctataaatgtcttttttt   100
MW847089: Suth-6               1   gggacgcagtagattcaagagacgtaagtatttaaaatgttagaaatacataaaaaaataaaaatatattatataagtatacctataaatgtcttttttt   100
MW847090: Suth-7               1   gggacgcagtagattcaagagacgtaagtatttaaaatgttagaaatacataaaaaaataaaaatatattatataagtatacctataaatgtcttttttt   100
MW847091: Suth-8               1   gggacgcagtagattcaagagacgtaagtatttaaaatgttagaaatacataaaaaaataaaaatatattatataagtatacctataaatgtcttttttt   100
MW847092: Nash-1               1   gggacgcagtagattcaagagacgtaagtatttaaaatgttagaaatacataaaaaaataaaaatatattatataagtatacctataaatgtcttttttt   100
MW847093: Nash-2               1   gggacgcagtagattcaagagacgtaagtatttaaaatgttagaaatacataaaaaaataaaaatatattatataagtatacctataaatgtcttttttt   100
MW847094: Nash-3               1   gggacgcagtagattcaagagacgtaagtatttaaaatgttagaaatacataaaaaaataaaaatatattatataagtatacctataaatgtcttttttt   100
MW847095: Nash-4               1   gggacgcagtagattcaagagacgtaagtatttaaaatgttagaaatacataaaaaaataaaaatatattatataagtatacctataaatgtcttttttt   100
MW847096: Nash-5               1   gggacgcagtagattcaagagacgtaagtatttaaaatgttagaaatacataaaaaaataaaaatatattatataagtatacctataaatgtcttttttt   100
MW847097: Nash-6               1   gggacgcagtagattcaagagacgtaagtatttaaaatgttagaaatacataaaaaaataaaaatatattatataagtatacctataaatgtcttttttt   100
MW847098: Nash-7               1   gggacgcagtagattcaagagacgtaagtatttaaaatgttagaaatacataaaaaaataaaaatatattatataagtatacctataaatgtcttttttt   100
MW847099: Nash-8               1   gggacgcagtagattcaagagacgtaagtatttaaaatgttagaaatacataaaaaaataaaaatatattatataagtatacctataaatgtcttttttt   100
MW847100: MN1-1-1              1   gggacgcagtagattcaagagacgtaagtatttaaaatgttagaaatacataaaaaaataaaaatatattatataagtatacctataaatgtcttttttt   100
MW847101: MN1-1-2              1   gggacgcagtagattcaagagacgtaagtatttaaaatgttagaaatacataaaaaaataaaaatatattatataagtatacctataaatgtcttttttt   100
MW847102: MN1-1-3              1   gggacgcagtagattcaagagacgtaagtatttaaaatgttagaaatacataaaaaaataaaaatatattatataagtatacctataaatgtcttttttt   100
MW847103: MN1-1-4              1   gggacgcagtagattcaagagacgtaagtatttaaaatgttagaaatacataaaaaaataaaaatatattatataagtatacctataaatgtcttttttt   100
MW847104: MN1-2-1              1   gggacgcagtagattcaagagacgtaagtatttaaaatgttagaaatacataaaaaaataaaaatatattatataagtatacctataaatgtcttttttt   100
MW847105: MN1-2-2              1   gggacgcagtagattcaagagacgtaagtatttaaaatgttagaaatacataaaaaaataaaaatatattatataagtatacctataaatgtcttttttt   100
MW847106: MN1-2-3              1   gggacgcagtagattcaagagacgtaagtatttaaaatgttagaaatacataaaaaaataaaaatatattatataagtatacctataaatgtcttttttt   100
MW847107: MN1-2-4              1   gggacgcagtagattcaagagacgtaagtatttaaaatgttagaaatacataaaaaaataaaaatatattatataagtatacctataaatgtcttttttt   100
MW847108: MN2-1-1              1   gggacgcagtagattcaagagacgtaagtatttaaaatgttagaaatacataaaaaaataaaaatatattatataagtatacctataaatgtcttttttt   100
MW847109: MN2-1-2              1   gggacgcagtagattcaagagacgtaagtatttaaaatgttagaaatacataaaaaaataaaaatatattatataagtatacctataaatgtcttttttt   100
MW847110: MN2-1-3              1   gggacgcagtagattcaagagacgtaagtatttaaaatgttagaaatacataaaaaaataaaaatatattatataagtatacctataaatgtcttttttt   100
MW847111: MN2-2-1              1   gggacgcagtagattcaagagacgtaagtatttaaaatgttagaaatacataaaaaaataaaaatatattatataagtatacctataaatgtcttttttt   100
MW847112: MN2-2-2              1   gggacgcagtagattcaagagacgtaagtatttaaaatgttagaaatacataaaaaaataaaaatatattatataagtatacctataaatgtcttttttt   100
MW847113: MN2-2-3              1   gggacgcagtagattcaagagacgtaagtatttaaaatgttagaaatacataaaaaaataaaaatatattatataagtatacctataaatgtcttttttt   100
MW847114: MN2-2-4              1   gggacgcagtagattcaagagacgtaagtatttaaaatgttagaaatacataaaaaaataaaaatatattatataagtatacctataaatgtcttttttt   100
MW847115: 9R1A-1               1   gggacgcagtagattcaagagacgtaagtatttaaaatgttagaaatacataaaaaaataaaaatatattatataagtatacctataaatgtcttttttt   100
MW847116: 9R2A-1               1   gggacgcagtagattcaagagacgtaagtatttaaaatgttagaaatacataaaaaaataaaaatatattatataagtatacctataaatgtcttttttt   100
MW847117: 9R3A-1               1   gggacgcagtagattcaagagacgtaagtatttaaaatgttagaaatacataaaaaaataaaaatatattatataagtatacctataaatgtcttttttt   100
MW847118: 9R3A-2               1   gggacgcagtagattcaagagacgtaagtatttaaaatgttagaaatacataaaaaaataaaaatatattatataagtatacctataaatgtcttttttt   100
MW847119: 9R1D-1               1   gggacgcagtagattcaagagacgtaagtatttaaaatgttagaaatacataaaaaaataaaaatatattatataagtatacctataaatgtcttttttt   100
MW847120: 9R1D-2               1   gggacgcagtagattcaagagacgtaagtatttaaaatgttagaaatacataaaaaaataaaaatatattatataagtatacctataaatgtcttttttt   100
MW847121: 9R1D-3               1   gggacgcagtagattcaagagacgtaagtatttaaaatgttagaaatacataaaaaaataaaaatatattatataagtatacctataaatgtcttttttt   100
MW847122: 9R1D-5               1   gggacgcagtagattcaagagacgtaagtatttaaaatgttagaaatacataaaaaaataaaaatatattatataagtatacctataaatgtcttttttt   100
MW847123: 9R2D-1               1   gggacgcagtagattcaagagacgtaagtatttaaaatgttagaaatacataaaaaaataaaaatatattatataagtatacctataaatgtcttttttt   100
MW847124: 9R2D-2               1   gggacgcagtagattcaagagacgtaagtatttaaaatgttagaaatacataaaaaaataaaaatatattatataagtatacctataaatgtcttttttt   100
MW847125: 9R2D-3               1   gggacgcagtagattcaagagacgtaagtatttaaaatgttagaaatacataaaaaaataaaaatatattatataagtatacctataaatgtcttttttt   100
MW847126: 9R2D-4               1   gggacgcagtagattcaagagacgtaagtatttaaaatgttagaaatacataaaaaaataaaaatatattatataagtatacctataaatgtcttttttt   100
MW847127: 9R2D-5               1   gggacgcagtagattcaagagacgtaagtatttaaaatgttagaaatacataaaaaaataaaaatatattatataagtatacctataaatgtcttttttt   100
MW847128: 9R2D-6               1   gggacgcagtagattcaagagacgtaagtatttaaaatgttagaaatacataaaaaaataaaaatatattatataagtatacctataaatgtcttttttt   100
MW847129: 9R2D-7               1   gggacgcagtagattcaagagacgtaagtatttaaaatgttagaaatacataaaaaaataaaaatatattatataagtatacctataaatgtcttttttt   100
MW847130: 9R2D-8               1   gggacgcagtagattcaagagacgtaagtatttaaaatgttagaaatacataaaaaaataaaaatatattatataagtatacctataaatgtcttttttt   100
MW847131: 7R3D-1               1   gggacgcagtagattcaagagacgtaagtatttaaaatgttagaaatacataaaaaaataaaaatatattatataagtatacctataaatgtcttttttt   100
MW847132: 7R3D-2               1   gggacgcagtagattcaagagacgtaagtatttaaaatgttagaaatacataaaaaaataaaaatatattatataagtatacctataaatgtcttttttt   100
MW847133: 7R3D-3               1   gggacgcagtagattcaagagacgtaagtatttaaaatgttagaaatacataaaaaaataaaaatatattatataagtatacctataaatgtcttttttt   100
MW847134: 7R3D-4               1   gggacgcagtagattcaagagacgtaagtatttaaaatgttagaaatacataaaaaaataaaaatatattatataagtatacctataaatgtcttttttt   100
MW847135: 7R3D-5               1   gggacgcagtagattcaagagacgtaagtatttaaaatgttagaaatacataaaaaaataaaaatatattatataagtatacctataaatgtcttttttt   100
MW847136: 7R3D-6               1   gggacgcagtagattcaagagacgtaagtatttaaaatgttagaaatacataaaaaaataaaaatatattatataagtatacctataaatgtcttttttt   100
MW847137: 7R3D-7               1   gggacgcagtagattcaagagacgtaagtatttaaaatgttagaaatacataaaaaaataaaaatatattatataagtatacctataaatgtcttttttt   100
MW847138: 7R3D-8               1   gggacgcagtagattcaagagacgtaagtatttaaaatgttagaaatacataaaaaaataaaaatatattatataagtatacctataaatgtcttttttt   100
MW847139: 7R3A-1               1   gggacgcagtagattcaagagacgtaagtatttaaaatgttagaaatacataaaaaaataaaaatatattatataagtatacctataaatgtcttttttt   100
MW847140: 7R3A-2               1   gggacgcagtagattcaagagacgtaagtatttaaaatgttagaaatacataaaaaaataaaaatatattatataagtatacctataaatgtcttttttt   100
MW847141: 7R3A-3               1   gggacgcagtagattcaagagacgtaagtatttaaaatgttagaaatacataaaaaaataaaaatatattatataagtatacctataaatgtcttttttt   100
MW847142: 7R3A-4               1   gggacgcagtagattcaagagacgtaagtatttaaaatgttagaaatacataaaaaaataaaaatatattatataagtatacctataaatgtcttttttt   100
MW847143: 7R3A-5               1   gggacgcagtagattcaagagacgtaagtatttaaaatgttagaaatacataaaaaaataaaaatatattatataagtatacctataaatgtcttttttt   100
MW847144: 7R3A-6               1   gggacgcagtagattcaagagacgtaagtatttaaaatgttagaaatacataaaaaaataaaaatatattatataagtatacctataaatgtcttttttt   100
MW847145: 7R3A-7               1   gggacgcagtagattcaagagacgtaagtatttaaaatgttagaaatacataaaaaaataaaaatatattatataagtatacctataaatgtcttttttt   100
MW847146: 7R2A-1               1   gggacgcagtagattcaagagacgtaagtatttaaaatgttagaaatacataaaaaaataaaaatatattatataagtatacctataaatgtcttttttt   100
```

Susceptible (SUS) laboratory colonies (MW847052–MW847079)

Resistant (RES) field collections (MW847084–MW847146)

```
                                                                                                                M1524I
                                                                                                              ..DIII S6...
                                   ---------------------- intron ---------------------| T  H  G  K  Q  P  I  R  E  I  N  N  Y  M  Y  F
AgVGSC-H2_cDNA       1261   ****************************************************ACGCACGGGAAACAGCCAATTCGTGAAATCAATAATTATATGTATTTT   1308
MW847052: ISU-B1_1    101   gacatacatttataaaatactgatgtatatttgttattatatgtttttttagACGCACGGGAAACAGCCAATTCGTGAAATCAATAATTATATGTATTTT   200
MW847053: ISU-B1_5    101   GACATACATTTATAAAATACTGATGTATATTTGTTATTATATGTTTTTTTAGACGCACGGGAAACAGCCAATTCGTGAAATCAATAATTATATGTATTTT   200
MW847054: ISU-B1_6    101   GACATACATTTATAAAATACTGATGTATATTTGTTATTATATGTTTTTTTAGACGCACGGGAAACAGCCAATTCGTGAAATCAATAATTATATGTATTTT   200
MW847055: ISU-B1_14   101   GACATACATTTATAAAATACTGATGTATATTTGTTATTATATGTTTTTTTAGACGCACGGGAAACAGCCAATTCGTGAAATCAATAATTATATGTATTTT   200
MW847068: ISU-B3_4    101   GACATACATTTATAAAATACTGATGTATATTTGTTATTATATGTTTTTTTAGACGCACGGGAAACAGCCAATTCGTGAAATCAATAATTATATGTATTTT   200
MW847069: ISU-B3_6    101   GACATACATTTATAAAATACTGATGTATATTTGTTATTATATGTTTTTTTAGACGCACGGGAAACAGCCAATTCGTGAAATCAATAATTATATGTATTTT   200
MW847070: ISU-B3_8    101   GACATACATTTATAAAATACTGATGTATATTTGTTATTATATGTTTTTTTAGACGCACGGGAAACAGCCAATTCGTGAAATCAATAATTATATGTATTTT   200
MW847071: ISU-B3_14   101   GACATACATTTATAAAATACTGATGTATATTTGTTATTATATGTTTTTTTAGACGCACGGGAAACAGCCAATTCGTGAAATCAATAATTATATGTATTTT   200
MW847056: UIL-B1_2    101   GACATACATTTATAAAATACTGATGTATATTTGTTATTATATGTTTTTTTAGACGCACGGGAAACAGCCAATTCGTGAAATCAATAATTATATGTATTTT   200
MW847057: UIL-B1_5    101   GACATACATTTATAAAATACTGATGTATATTTGTTATTATATGTTTTTTTAGACGCACGGGAAACAGCCAATTCGTGAAATCAATAATTATATGTATTTT   200
MW847058: UIL-B1_10   101   GACATACATTTATAAAATACTGATGTATATTTGTTATTATATGTTTTTTTAGACGCACGGGAAACAGCCAATTCGTGAAATCAATAATTATATGTATTTT   200
MW847059: UIL-B1_19   101   GACATACATTTATAAAATACTGATGTATATTTGTTATTATATGTTTTTTTAGACGCACGGGAAACAGCCAATTCGTGAAATCAATAATTATATGTATTTT   200
MW847076: ISU-B4_5    101   GACATACATTTATAAAATACTGATGTATATTTGTTATTATATGTTTTTTTAGACGCACGGGAAACAGCCAATTCGTGAAATCAATAATTATATGTATTTT   200
MW847077: ISU-B4_8    101   GACATACATTTATAAAATACTGATGTATATTTGTTATTATATGTTTTTTTAGACGCACGGGAAACAGCCAATTCGTGAAATCAATAATTATATGTATTTT   200
MW847078: ISU-B4_13   101   GACATACATTTATAAAATACTGATGTATATTTGTTATTATATGTTTTTTTAGACGCACGGRAAACAGCCAATTCGTGAAATCAATAATTATATGTATTTT   200
MW847079: ISU-B4_16   101   GACATACATTTATAAAATACTGATGTATATTTGTTATTATATGTTTTTTTAGACGCACGGGAAACAGCCAATTCGTGAAATCAATAATTATATGTATTTT   200
MW847084: Suth-1      101   GACATACATTTATAAAATACTGATGTATATTTGTTATTATATGTTTTTTTAGACGCACGGGAAACAGCCAATTCGTGAAATCAATAATTATATGTATTTT   200
MW847085: Suth-2      101   GACATACATTTATAAAATACTGATGTATATTTGTTATTATATGTTTTTTTAGACGCACGGGAAACAGCCAATTCGTGAAATCAATAATTATATGTATTTT   200
MW847086: Suth-3      101   GACATACATTTATAAAATACTGATGTATATTTGTTATTATATGTTTTTTTAGACGCACGGGAAACAGCCAATTCGTGAAATCAATAATTATATGTATTTT   200
MW847087: Suth-4      101   GACATACATTTATAAAATACTGATGTATATTTGTTATTATATGTTTTTTTAGACGCACGGGAAACAGCCAATTCGTGAAATCAATAATTATATGTATTTT   200
MW847088: Suth-5      101   GACATACATTTATAAAATACTGATGTATATTTGTTATTATATGTTTTTTTAGACGCACGGRAAACAGCCAATTCGTGAAATCAATAATTATATGTATTTT   200
MW847089: Suth-6      101   GACATACATTTATAAAATACTGATGTATATTTGTTATTATATGTTTTTTTAGACGCACGGGAAACAGCCAATTCGTGAAATCAATAATTATATGTATTTT   200
MW847090: Suth-7      101   GACATACATTTATAAAATACTGATGTATATTTGTTATTATATGTTTTTTTAGACGCACGGGAAACAGCCAATTCGTGAAATCAATAATTATATGTATTTT   200
MW847091: Suth-8      101   GACATACATTTATAAAATACTGATGTATATTTGTTATTATATGTTTTTTTAGACGCACGGGAAACAGCCAATTCGTGAAATCAATAATTATATGTATTTT   200
MW847092: Nash-1      101   GACATACATTTATAAAATACTGATGTATATTTGTTATTATATGTTTTTTTAGACGCACGGGAAACAGCCAATTCGTGAAATCAATAATTATATGTATTTT   200
MW847093: Nash-2      101   GACATACATTTATAAAATACTGATGTATATTTGTTATTATATGTTTTTTTAGACGCACGGGAAACAGCCAATTCGTGAAATCAATAATTATATGTATTTT   200
MW847094: Nash-3      101   GACATACATTTATAAAATACTGATGTATATTTGTTATTATATGTTTTTTTAGACGCACGGRAAACAGCCAATTCGTGAAATCAATAATTATATGTATTTT   200
MW847095: Nash-4      101   GACATACATTTATAAAATACTGATGTATATTTGTTATTATATGTTTTTTTAGACGCACGGGAAACAGCCAATTCGTGAAATCAATAATTATATGTATTTT   200
MW847096: Nash-5      101   GACATACATTTATAAAATACTGATGTATATTTGTTATTATATGTTTTTTTAGACGCACGGGAAACAGCCAATTCGTGAAATCAATAATTATATGTATTTT   200
MW847097: Nash-6      101   GACATACATTTATAAAATACTGATGTATATTTGTTATTATATGTTTTTTTAGACGCACGGGAAACAGCCAATTCGTGAAATCAATAATTATATGTATTTT   200
MW847098: Nash-7      101   GACATACATTTATAAAATACTGATGTATATTTGTTATTATATGTTTTTTTAGACGCACGGGAAACAGCCAATTCGTGAAATCAATAATTATATGTATTTT   200
MW847099: Nash-8      101   GACATACATTTATAAAATACTGATGTATATTTGTTATTATATGTTTTTTTAGACGCACGGGAAACAGCCAATTCGTGAAATCAATAATTATATGTATTTT   200
MW847100: MN1-1-1     101   GACATACATTTATAAAATACTGATGTATATTTGTTATTATATGTTTTTTTAGACGCACGGGAAACAGCCAATTCGTGAAATCAATAATTATATGTATTTT   200
MW847101: MN1-1-2     101   GACATACATTTATAAAATACTGATGTATATTTGTTATTATATGTTTTTTTAGACGCACGGGAAACAGCCAATTCGTGAAATCAATAATTATATGTATTTT   200
MW847102: MN1-1-3     101   GACATACATTTATAAAATACTGATGTATATTTGTTATTATATGTTTTTTTAGACGCACGGGAAACAGCCAATTCGTGAAATCAATAATTATATGTATTTT   200
MW847103: MN1-1-4     101   GACATACATTTATAAAATACTGATGTATATTTGTTATTATATGTTTTTTTAGACGCACGGGAAACAGCCAATTCGTGAAATCAATAATTATATGTATTTT   200
MW847104: MN1-2-1     101   GACATACATTTATAAAATACTGATGTATATTTGTTATTATATGTTTTTTTAGACGCACGGRAAACAGCCAATTCGTGAAATCAATAATTATATGTATTTT   200
MW847105: MN1-2-2     101   GACATACATTTATAAAATACTGATGTATATTTGTTATTATATGTTTTTTTAGACGCACGGGAAACAGCCAATTCGTGAAATCAATAATTATATGTATTTT   200
MW847106: MN1-2-3     101   GACATACATTTATAAAATACTGATGTATATTTGTTATTATATGTTTTTTTAGACGCACGGGAAACAGCCAATTCGTGAAATCAATAATTATATGTATTTT   200
MW847107: MN1-2-4     101   GACATACATTTATAAAATACTGATGTATATTTGTTATTATATGTTTTTTTAGACGCACGGGAAACAGCCAATTCGTGAAATCAATAATTATATGTATTTT   200
MW847108: MN2-1-1     101   GACATACATTTATAAAATACTGATGTATATTTGTTATTATATGTTTTTTTAGACGCACGGGAAACAGCCAATTCGTGAAATCAATAATTATATGTATTTT   200
MW847109: MN2-1-2     101   GACATACATTTATAAAATACTGATGTATATTTGTTATTATATGTTTTTTTAGACGCACGGGAAACAGCCAATTCGTGAAATCAATAATTATATGTATTTT   200
MW847110: MN2-1-3     101   GACATACATTTATAAAATACTGATGTATATTTGTTATTATATGTTTTTTTAGACGCACGGGAAACAGCCAATTCGTGAAATCAATAATTATATGTATTTT   200
MW847111: MN2-2-1     101   GACATACATTTATAAAATACTGATGTATATTTGTTATTATATGTTTTTTTAGACGCACGGGAAACAGCCAATTCGTGAAATCAATAATTATATGTATTTT   200
MW847112: MN2-2-2     101   GACATACATTTATAAAATACTGATGTATATTTGTTATTATATGTTTTTTTAGACGCACGGGAAACAGCCAATTCGTGAAATCAATAATTATATGTATTTT   200
MW847113: MN2-2-3     101   GACATACATTTATAAAATACTGATGTATATTTGTTATTATATGTTTTTTTAGACGCACGGGAAACAGCCAATTCGTGAAATCAATAATTATATGTATTTT   200
MW847114: MN2-2-4     101   GACATACATTTATAAAATACTGATGTATATTTGTTATTATATGTTTTTTTAGACGCACGGGAAACAGCCAATTCGTGAAATCAATAATTATATGTATTTT   200
MW847115: 9R1A-1      101   GACATACATTTATAAAATACTGATGTATATTTGTTATTATATGTTTTTTTAGACGCACGGGAAACAGCCAATTCGTGAAATCAATAATTATATGTATTTT   200
MW847116: 9R2A-1      101   GACATACATTTATAAAATACTGATGTATATTTGTTATTATATGTTTTTTTAGACGCACGGGAAACAGCCAATTCGTGAAATCAATAATTATATGTATTTT   200
MW847117: 9R3A-1      101   GACATACATTTATAAAATACTGATGTATATTTGTTATTATATGTTTTTTTAGACGCACGGGAAACAGCCAATTCGTGAAATCAATAATTATATGTATTTT   200
MW847118: 9R3A-2      101   GACATACATTTATAAAATACTGATGTATATTTGTTATTATATGTTTTTTTAGACGCACGGGAAACAGCCAATTCGTGAAATCAATAATTATATGTATTTT   200
MW847119: 9R1D-1      101   GACATACATTTATAAAATACTGATGTATATTTGTTATTATATGTTTTTTTAGACGCACGGGAAACAGCCAATTCGTGAAATCAATAATTATATGTATTTT   200
MW847120: 9R1D-2      101   GACATACATTTATAAAATACTGATGTATATTTGTTATTATATGTTTTTTTAGACGCACGGRAAACAGCCAATTCGTGAAATCAATAATTATATGTATTTT   200
MW847121: 9R1D-3      101   GACATACATTTATAAAATACTGATGTATATTTGTTATTATATGTTTTTTTAGACGCACGGGAAACAGCCAATTCGTGAAATCAATAATTATATGTATTTT   200
MW847122: 9R1D-5      101   GACATACATTTATAAAATACTGATGTATATTTGTTATTATATGTTTTTTTAGACGCACGGGAAACAGCCAATTCGTGAAATCAATAATTATATGTATTTT   200
MW847123: 9R2D-1      101   GACATACATTTATAAAATACTGATGTATATTTGTTATTATATGTTTTTTTAGACGCACGGGAAACAGCCAATTCGTGAAATCAATAATTATATGTATTTT   200
MW847124: 9R2D-2      101   GACATACATTTATAAAATACTGATGTATATTTGTTATTATATGTTTTTTTAGACGCACGGGAAACAGCCAATTCGTGAAATCAATAATTATATGTATTTT   200
MW847125: 9R2D-3      101   GACATACATTTATAAAATACTGATGTATATTTGTTATTATATGTTTTTTTAGACGCACGGGAAACAGCCAATTCGTGAAATCAATAATTATATGTATTTT   200
MW847126: 9R2D-4      101   GACATACATTTATAAAATACTGATGTATATTTGTTATTATATGTTTTTTTAGACGCACGGGAAACAGCCAATTCGTGAAATCAATAATTATATGTATTTT   200
MW847127: 9R2D-5      101   GACATACATTTATAAAATACTGATGTATATTTGTTATTATATGTTTTTTTAGACGCACGGGAAACAGCCAATTCGTGAAATCAATAATTATATGTATTTT   200
MW847128: 9R2D-6      101   GACATACATTTATAAAATACTGATGTATATTTGTTATTATATGTTTTTTTAGACGCACGGGAAACAGCCAATTCGTGAAATCAATAATTATATGTATTTT   200
MW847129: 9R2D-7      101   GACATACATTTATAAAATACTGATGTATATTTGTTATTATATGTTTTTTTAGACGCACGGGAAACAGCCAATTCGTGAAATCAATAATTATATGTATTTT   200
MW847130: 9R2D-8      101   GACATACATTTATAAAATACTGATGTATATTTGTTATTATATGTTTTTTTAGACGCACGGRAAACAGCCAATTCGTGAAATCAATAATTATATGTATTTT   200
MW847131: 7R3D-1      101   GACATACATTTATAAAATACTGATGTATATTTGTTATTATATGTTTTTTTAGACGCACGGGAAACAGCCAATTCGTGAAATCAATAATTATATGTATTTT   200
MW847132: 7R3D-2      101   GACATACATTTATAAAATACTGATGTATATTTGTTATTATATGTTTTTTTAGACGCACGGGAAACAGCCAATTCGTGAAATCAATAATTATATGTATTTT   200
MW847133: 7R3D-3      101   GACATACATTTATAAAATACTGATGTATATTTGTTATTATATGTTTTTTTAGACGCACGGGAAACAGCCAATTCGTGAAATCAATAATTATATGTATTTT   200
MW847134: 7R3D-4      101   GACATACATTTATAAAATACTGATGTATATTTGTTATTATATGTTTTTTTAGACGCACGGGAAACAGCCAATTCGTGAAATCAATAATTATATGTATTTT   200
MW847135: 7R3D-5      101   GACATACATTTATAAAATACTGATGTATATTTGTTATTATATGTTTTTTTAGACGCACGGGAAACAGCCAATTCGTGAAATCAATAATTATATGTATTTT   200
MW847136: 7R3D-6      101   GACATACATTTATAAAATACTGATGTATATTTGTTATTATATGTTTTTTTAGACGCACGGGAAACAGCCAATTCGTGAAATCAATAATTATATGTATTTT   200
MW847137: 7R3D-7      101   GACATACATTTATAAAATACTGATGTATATTTGTTATTATATGTTTTTTTAGACGCACGGGAAACAGCCAATTCGTGAAATCAATAATTATATGTATTTT   200
MW847138: 7R3D-8      101   GACATACATTTATAAAATACTGATGTATATTTGTTATTATATGTTTTTTTAGACGCACGGGAAACAGCCAATTCGTGAAATCAATAATTATATGTATTTT   200
MW847139: 7R3A-1      101   GACATACATTTATAAAATACTGATGTATATTTGTTATTATATGTTTTTTTAGACGCACGGGAAACAGCCAATTCGTGAAATCAATAATTATATGTATTTT   200
MW847140: 7R3A-2      101   GACATACATTTATAAAATACTGATGTATATTTGTTATTATATGTTTTTTTAGACGCACGGGAAACAGCCAATTCGTGAAATCAATAATTATATGTATTTT   200
MW847141: 7R3A-3      101   GACATACATTTATAAAATACTGATGTATATTTGTTATTATATGTTTTTTTAGACGCACGGGAAACAGCCAATTCGTGAAATCAATAATTATATGTATTTT   200
MW847142: 7R3A-4      101   GACATACATTTATAAAATACTGATGTATATTTGTTATTATATGTTTTTTTAGACGCACGGGAAACAGCCAATTCGTGAAATCAATAATTATATGTATTTT   200
MW847143: 7R3A-5      101   GACATACATTTATAAAATACTGATGTATATTTGTTATTATATGTTTTTTTAGACGCACGGGAAACAGCCAATTCGTGAAATCAATAATTATATGTATTTT   200
MW847144: 7R3A-6      101   GACATACATTTATAAAATACTGATGTATATTTGTTATTATATGTTTTTTTAGACGCACGGGAAACAGCCAATTCGTGAAATCAATAATTATATGTATTTT   200
MW847145: 7R3A-7      101   GACATACATTTATAAAATACTGATGTATATTTGTTATTATATGTTTTTTTAGACGCACGGGAAACAGCCAATTCGTGAAATCAATAATTATATGTATTTT   200
MW847146: 7R2A-1      101   GACATACATTTATAAAATACTGATGTATATTTGTTATTATATGTTTTTTTAGACGCACGGGAAACAGCCAATTCGTGAAATCAATAATTATATGTATTTT   200
```

Susceptible (SUS) laboratory colonies: MW847052–MW847079

Resistant (RES) field collections: MW847084–MW847146

```
                                           F1528L                                   F1538I                                      D1549V          E1553G
                                          ...............................DIII S6..............................
                                           Y  F  V  F  F  I  I  F  G  S  F  F  T  L  N  L  F  I  G  V  I  I  D  N  F  N  E  Q  K  K  K  T  |--
AgVGSC-H2_cDNA          1309   TATTTTGTTTTTTTTATTATTTTTGGTTCATTTTTTACTCTTAATTTATTCATTGGTGTGATCATTGACAATTTCAACGAACAGAAGAAAAAAACAGgtg   1405
MW847052: ISU-B1_1       201   TATTTTGTTTTTTTTATTATTTTTGGTTCATTTTTTACTCTTAATTTATTCATTGGTGTGATCATTGACAATTTCAACGAACAGAAGAAAAAAACAGgtg    300
MW847053: ISU-B1_5       201   TATTTTGTTTTTTTTATTATTTTTGGTTCATTTTTTACTCTTAATTTATTCATTGGKGTGATCATTGACAATTTCAACGAACAGAAGAAAAAAACAGgtg    300
MW847054: ISU-B1_6       201   TATTTTGTTTTTTTTATTATTTTTGGTTCATTTTTTACTCTTAATTTATTCATTGGKGTGATCATTGACAATTTCAACGAACAGAAGAAAAAAACAGgtg    300
MW847055: ISU-B1_14      201   TATTTTGTTTTTTTTATTATTTTTGGTTCATTTTTTACTCTTAATTTATTCATTGGTGTGATCATTGACAATTTCAACGAACAGAAGAAAAAAACAGgtg    300
MW847056: UIL-B1_2       201   TATTTTGTTTTTTTTATTATTTTTGGTTCATTTTTTACTCTTAATTTATTCATTGGKGTGATCATTGACAATTTCAACGAACAGAAGAAAAAAACAGgtg    300
MW847057: UIL-B1_5       201   TATTTTGTTTTTTTTATTATTTTTGGTTCATTTTTTACTCTTAATTTATTCATTGGTGTGATCATTGACAATTTCAACGAACAGAAGAAAAAAACAGgtg    300
MW847058: UIL-B1_10      201   TATTTTGTTTTTTTTATTATTTTTGGTTCATTTTTTACTCTTAATTTATTCATTGGTGTGATCATTGACAATTTCAACGAACAGAAGAAAAAAACAGgtg    300
MW847059: UIL-B1_19      201   TATTTTGTTTTTTTTATTATTTTTGGTTCATTTTTTACTCTTAATTTATTCATTGGKGTGATCATTGACAATTTCAACGAACAGAAGAAAAAAACAGgtg    300
MW847068: ISU-B3_4       201   TATTTTGTTTTTTTTATTATTTTTGGTTCATTTTTTACTCTTAATTTATTCATTGGKGTGATCATTGACAATTTCAACGAACAGAAGAAAAAAACAGgtg    300
MW847069: ISU-B3_6       201   TATTTTGTTTTTTTTATTATTTTTGGTTCATTTTTTACTCTTAATTTATTCATTGGKGTGATCATTGACAATTTCAACGAACAGAAGAAAAAAACAGgtg    300
MW847070: ISU-B3_8       201   TATTTTGTTTTTTTTATTATTTTTGGTTCATTTTTTACTCTTAATTTATTCATTGGKGTGATCATTGACAATTTCAACGAACAGAAGAAAAAAACAGgtg    300
MW847071: ISU-B3_14      201   TATTTTGTTTTTTTTATTATTTTTGGTTCATTTTTTACTCTTAATTTATTCATTGGTGTGATCATTGACAATTTCAACGAACAGAAGAAAAAAACAGgtg    300
MW847076: ISU-B4_5       201   TATTTTGTTTTTTTTATTATTTTTGGTTCATTTTTTACTCTTAATTTATTCATTGGTGTGATCATTGACAATTTCAACGAACAGAAGAAAAAAACAGgtg    300
MW847077: ISU-B4_8       201   TATTTTGTTTTTTTTATTATTTTTGGTTCATTTTTTACTCTTAATTTATTCATTGGKGTGATCATTGACAATTTCAACGAACAGAAGAAAAAAACAGgtg    300
MW847078: ISU-B4_13      201   TATTTTGTTTTTTTTATTATTTTTGGTTCATTTTTTACTCTTAATTTATTCATTGGKGTGATCATTGACAATTTCAACGAACAGAAGAAAAAAACAGgtg    300
MW847079: ISU-B4_16      201   TATTTTGTTTTTTTTATTATTTTTGGTTCATTTTTTACTCTTAATTTATTCATTGGKGTGATCATTGACAATTTCAACGAACAGAAGAAAAAAACAGgtg    300
MW847084: Suth-1         201   TATTTTGTTTTTTTTATTATTTTTGGTTCATTTTTTACTCTTAATTTATTCATTGGKGTGATCATTGACAATTTCAACGAACAGAAGAAAAAAACAGgtg    300
MW847085: Suth-2         201   TATTTTGTTTTTTTTATTATTTTTGGTTCATTTTTTACTCTTAATTTATTCATTGGKGTGATCATTGACAATTTCAACGAACAGAAGAAAAAAACAGgtg    300
MW847086: Suth-3         201   TATTTTGTTTTTTTTATTATTTTTGGTTCATTTTTTACTCTTAATTTATTCATTGGKGTGATCATTGACAATTTCAACGAACAGAAGAAAAAAACAGgtg    300
MW847087: Suth-4         201   TATTTTGTTTTTTTTATTATTTTTGGTTCATTTTTTACTCTTAATTTATTCATTGGKGTGATCATTGACAATTTCAACGAACAGAAGAAAAAAACAGgtg    300
MW847088: Suth-5         201   TATTTTGTTTTTTTTATTATTTTTGGTTCATTTTTTACTCTTAATTTATTCATTGGKGTGATCATTGACAATTTCAACGAACAGAAGAAAAAAACAGgtg    300
MW847089: Suth-6         201   TATTTTGTTTTTTTTATTATTTTTGGTTCATTTTTTACTCTTAATTTATTCATTGGKGTGATCATTGACAATTTCAACGAACAGAAGAAAAAAACAGgtg    300
MW847090: Suth-7         201   TATTTTGTTTTTTTTATTATTTTTGGTTCATTTTTTACTCTTAATTTATTCATTGGTGTGATCATTGACAATTTCAACGAACAGAAGAAAAAAACAGgtg    300
MW847091: Suth-8         201   TATTTTGTTTTTTTTATTATTTTTGGTTCATTTTTTACTCTTAATTTATTCATTGGTGTGATCATTGACAATTTCAACGAACAGAAGAAAAAAACAGgtg    300
MW847092: Nash-1         201   TATTTTGTTTTTTTTATTATTTTTGGTTCATTTTTTACTCTTAATTTATTCATTGGKGTGATCATTGACAATTTCAACGAACAGAAGAAAAAAACAGgtg    300
MW847093: Nash-2         201   TATTTTGTTTTTTTTATTATTTTTGGTTCATTTTTTACTCTTAATTTATTCATTGGKGTGATCATTGACAATTTCAACGAACAGAAGAAAAAAACAGgtg    300
MW847094: Nash-3         201   TATTTTGTTTTTTTTATTATTTTTGGTTCATTTTTTACTCTTAATTTATTCATTGGKGTGATCATTGACAATTTCAACGAACAGAAGAAAAAAACAGgtg    300
MW847095: Nash-4         201   TATTTTGTTTTTTTTATTATTTTTGGTTCATTTTTTACTCTTAATTTATTCATTGGKGTGATCATTGACAATTTCAACGAACAGAAGAAAAAAACAGgtg    300
MW847096: Nash-5         201   TATTTTGTTTTTTTTATTATTTTTGGTTCATTTTTTACTCTTAATTTATTCATTGGKGTGATCATTGACAATTTCAACGAACAGAAGAAAAAAACAGgtg    300
MW847097: Nash-6         201   TATTTTGTTTTTTTTATTATTTTTGGTTCATTTTTTACTCTTAATTTATTCATTGGTGTGATCATTGACAATTTCAACGAACAGAAGAAAAAAACAGgtg    300
MW847098: Nash-7         201   TATTTTGTTTTTTTTATTATTTTTGGTTCATTTTTTACTCTTAATTTATTCATTGGTGTGATCATTGACAATTTCAACGAACAGAAGAAAAAAACAGgtg    300
MW847099: Nash-8         201   TATTTTGTTTTTTTTATTATTTTTGGTTCATTTTTTACTCTTAATTTATTCATTGGKGTGATCATTGACAATTTCAACGAACAGAAGAAAAAAACAGgtg    300
MW847100: MN1-1-1        201   TATTTTGTTTTTTTTATTATTTTTGGTTCATTTTTTACTCTTAATTTATTCATTGGKGTGATCATTGACAATTTCAACGAACAGAAGAAAAAAACAGgtg    300
MW847101: MN1-1-2        201   TATTTTGTTTTTTTTATTATTTTTGGTTCATTTTTTACTCTTAATTTATTCATTGGKGTGATCATTGACAATTTCAACGAACAGAAGAAAAAAACAGgtg    300
MW847102: MN1-1-3        201   TATTTTGTTTTTTTTATTATTTTTGGTTCATTTTTTACTCTTAATTTATTCATTGGKGTGATCATTGACAATTTCAACGAACAGAAGAAAAAAACAGgtg    300
MW847103: MN1-1-4        201   TATTTTGTTTTTTTTATTATTTTTGGTTCATTTTTTACTCTTAATTTATTCATTGGKGTGATCATTGACAATTTCAACGAACAGAAGAAAAAAACAGgtg    300
MW847104: MN1-2-1        201   TATTTTGTTTTTTTTATTATTTTTGGTTCATTTTTTACTCTTAATTTATTCATTGGKGTGATCATTGACAATTTCAACGAACAGAAGAAAAAAACAGgtg    300
MW847105: MN1-2-2        201   TATTTTGTTTTTTTTATTATTTTTGGTTCATTTTTTACTCTTAATTTATTCATTGGTGTGATCATTGACAATTTCAACGAACAGAAGAAAAAAACAGgtg    300
MW847106: MN1-2-3        201   TATTTTGTTTTTTTTATTATTTTTGGTTCATTTTTTACTCTTAATTTATTCATTGGKGTGATCATTGACAATTTCAACGAACAGAAGAAAAAAACAGgtg    300
MW847107: MN1-2-4        201   TATTTTGTTTTTTTTATTATTTTTGGTTCATTTTTTACTCTTAATTTATTCATTGGKGTGATCATTGACAATTTCAACGAACAGAAGAAAAAAACAGgtg    300
MW847108: MN2-1-1        201   TATTTTGTTTTTTTTATTATTTTTGGTTCATTTTTTACTCTTAATTTATTCATTGGKGTGATCATTGACAATTTCAACGAACAGAAGAAAAAAACAGgtg    300
MW847109: MN2-1-2        201   TATTTTGTTTTTTTTATTATTTTTGGTTCATTTTTTACTCTTAATTTATTCATTGGKGTGATCATTGACAATTTCAACGAACAGAAGAAAAAAACAGgtg    300
MW847110: MN2-1-3        201   TATTTTGTTTTTTTTATTATTTTTGGTTCATTTTTTACTCTTAATTTATTCATTGGKGTGATCATTGACAATTTCAACGAACAGAAGAAAAAAACAGgtg    300
MW847111: MN2-2-1        201   TATTTTGTTTTTTTTATTATTTTTGGTTCATTTTTTACTCTTAATTTATTCATTGGTGTGATCATTGACAATTTCAACGAACAGAAGAAAAAAACAGgtg    300
MW847112: MN2-2-2        201   TATTTTGTTTTTTTTATTATTTTTGGTTCATTTTTTACTCTTAATTTATTCATTGGKGTGATCATTGACAATTTCAACGAACAGAAGAAAAAAACAGgtg    300
MW847113: MN2-2-3        201   TATTTTGTTTTTTTTATTATTTTTGGTTCATTTTTTACTCTTAATTTATTCATTGGKGTGATCATTGACAATTTCAACGAACAGAAGAAAAAAACAGgtg    300
MW847114: MN2-2-4        201   TATTTTGTTTTTTTTATTATTTTTGGTTCATTTTTTACTCTTAATTTATTCATTGGKGTGATCATTGACAATTTCAACGAACAGAAGAAAAAAACAGgtg    300
MW847115: 9R1A-1         201   TATTTTGTTTTTTTTATTATTTTTGGTTCATTTTTTACTCTTAATTTATTCATTGGKGTGATCATTGACAATTTCAACGAACAGAAGAAAAAAACAGgtg    300
MW847116: 9R2A-1         201   TATTTTGTTTTTTTTATTATTTTTGGTTCATTTTTTACTCTTAATTTATTCATTGGKGTGATCATTGACAATTTCAACGAACAGAAGAAAAAAACAGgtg    300
MW847117: 9R3A-1         201   TATTTTGTTTTTTTTATTATTTTTGGTTCATTTTTTACTCTTAATTTATTCATTGGKGTGATCATTGACAATTTCAACGAACAGAAGAAAAAAACAGgtg    300
MW847118: 9R3A-2         201   TATTTTGTTTTTTTTATTATTTTTGGTTCATTTTTTACTCTTAATTTATTCATTGGKGTGATCATTGACAATTTCAACGAACAGAAGAAAAAAACAGgtg    300
MW847119: 9R1D-1         201   TATTTTGTTTTTTTTATTATTTTTGGTTCATTTTTTACTCTTAATTTATTCATTGGKGTGATCATTGACAATTTCAACGAACAGAAGAAAAAAACAGgtg    300
MW847120: 9R1D-2         201   TATTTTGTTTTTTTTATTATTTTTGGTTCATTTTTTACTCTTAATTTATTCATTGGTGTGATCATTGACAATTTCAACGAACAGAAGAAAAAAACAGgtg    300
MW847121: 9R1D-3         201   TATTTTGTTTTTTTTATTATTTTTGGTTCATTTTTTACTCTTAATTTATTCATTGGTGTGATCATTGACAATTTCAACGAACAGAAGAAAAAAACAGgtg    300
MW847122: 9R1D-5         201   TATTTTGTTTTTTTTATTATTTTTGGTTCATTTTTTACTCTTAATTTATTCATTGGTGTGATCATTGACAATTTCAACGAACAGAAGAAAAAAACAGgtg    300
MW847123: 9R2D-1         201   TATTTTGTTTTTTTTATTATTTTTGGTTCATTTTTTACTCTTAATTTATTCATTGGKGTGATCATTGACAATTTCAACGAACAGAAGAAAAAAACAGgtg    300
MW847124: 9R2D-2         201   TATTTTGTTTTTTTTATTATTTTTGGTTCATTTTTTACTCTTAATTTATTCATTGGKGTGATCATTGACAATTTCAACGAACAGAAGAAAAAAACAGgtg    300
MW847125: 9R2D-3         201   TATTTTGTTTTTTTTATTATTTTTGGTTCATTTTTTACTCTTAATTTATTCATTGGKGTGATCATTGACAATTTCAACGAACAGAAGAAAAAAACAGgtg    300
MW847126: 9R2D-4         201   TATTTTGTTTTTTTTATTATTTTTGGTTCATTTTTTACTCTTAATTTATTCATTGGTGTGATCATTGACAATTTCAACGAACAGAAGAAAAAAACAGgtg    300
MW847127: 9R2D-5         201   TATTTTGTTTTTTTTATTATTTTTGGTTCATTTTTTACTCTTAATTTATTCATTGGKGTGATCATTGACAATTTCAACGAACAGAAGAAAAAAACAGgtg    300
MW847128: 9R2D-6         201   TATTTTGTTTTTTTTATTATTTTTGGTTCATTTTTTACTCTTAATTTATTCATTGGTGTGATCATTGACAATTTCAACGAACAGAAGAAAAAAACAGgtg    300
MW847129: 9R2D-7         201   TATTTTGTTTTTTTTATTATTTTTGGTTCATTTTTTACTCTTAATTTATTCATTGGKGTGATCATTGACAATTTCAACGAACAGAAGAAAAAAACAGgtg    300
MW847130: 9R2D-8         201   TATTTTGTTTTTTTTATTATTTTTGGTTCATTTTTTACTCTTAATTTATTCATTGGKGTGATCATTGACAATTTCAACGAACAGAAGAAAAAAACAGgtg    300
MW847131: 7R3D-1         201   TATTTTGTTTTTTTTATTATTTTTGGTTCATTTTTTACTCTTAATTTATTCATTGGKGTGATCATTGACAATTTCAACGAACAGAAGAAAAAAACAGgtg    300
MW847132: 7R3D-2         201   TATTTTGTTTTTTTTATTATTTTTGGTTCATTTTTTACTCTTAATTTATTCATTGGKGTGATCATTGACAATTTCAACGAACAGAAGAAAAAAACAGgtg    300
MW847133: 7R3D-3         201   TATTTTGTTTTTTTTATTATTTTTGGTTCATTTTTTACTCTTAATTTATTCATTGGKGTGATCATTGACAATTTCAACGAACAGAAGAAAAAAACAGgtg    300
MW847134: 7R3D-4         201   TATTTTGTTTTTTTTATTATTTTTGGTTCATTTTTTACTCTTAATTTATTCATTGGKGTGATCATTGACAATTTCAACGAACAGAAGAAAAAAACAGgtg    300
MW847135: 7R3D-5         201   TATTTTGTTTTTTTTATTATTTTTGGTTCATTTTTTACTCTTAATTTATTCATTGGKGTGATCATTGACAATTTCAACGAACAGAAGAAAAAAACAGgtg    300
MW847136: 7R3D-6         201   TATTTTGTTTTTTTTATTATTTTTGGTTCATTTTTTACTCTTAATTTATTCATTGGTGTGATCATTGACAATTTCAACGAACAGAAGAAAAAAACAGgtg    300
MW847137: 7R3D-7         201   TATTTTGTTTTTTTTATTATTTTTGGTTCATTTTTTACTCTTAATTTATTCATTGGKGTGATCATTGACAATTTCAACGAACAGAAGAAAAAAACAGgtg    300
MW847138: 7R3D-8         201   TATTTTGTTTTTTTTATTATTTTTGGTTCATTTTTTACTCTTAATTTATTCATTGGKGTGATCATTGACAATTTCAACGAACAGAAGAAAAAAACAGgtg    300
MW847139: 7R3A-1         201   TATTTTGTTTTTTTTATTATTTTTGGTTCATTTTTTACTCTTAATTTATTCATTGGKGTGATCATTGACAATTTCAACGAACAGAAGAAAAAAACAGgtg    300
MW847140: 7R3A-2         201   TATTTTGTTTTTTTTATTATTTTTGGTTCATTTTTTACTCTTAATTTATTCATTGGKGTGATCATTGACAATTTCAACGAACAGAAGAAAAAAACAGgtg    300
MW847141: 7R3A-3         201   TATTTTGTTTTTTTTATTATTTTTGGTTCATTTTTTACTCTTAATTTATTCATTGGKGTGATCATTGACAATTTCAACGAACAGAAGAAAAAAACAGgtg    300
MW847142: 7R3A-4         201   TATTTTGTTTTTTTTATTATTTTTGGTTCATTTTTTACTCTTAATTTATTCATTGGKGTGATCATTGACAATTTCAACGAACAGAAGAAAAAAACAGgtg    300
MW847143: 7R3A-5         201   TATTTTGTTTTTTTTATTATTTTTGGTTCATTTTTTACTCTTAATTTATTCATTGGTGTGATCATTGACAATTTCAACGAACAGAAGAAAAAAACAGgtg    300
MW847144: 7R3A-6         201   TATTTTGTTTTTTTTATTATTTTTGGTTCATTTTTTACTCTTAATTTATTCATTGGTGTGATCATTGACAATTTCAACGAACAGAAGAAAAAAACAGgtg    300
MW847145: 7R3A-7         201   TATTTTGTTTTTTTTATTATTTTTGGTTCATTTTTTACTCTTAATTTATTCATTGGKGTGATCATTGACAATTTCAACGAACAGAAGAAAAAAACAGgtg    300
MW847146: 7R2A-1         201   TATTTTGTTTTTTTTATTATTTTTGGTTCATTTTTTACTCTTAATTTATTCATTGGKGTGATCATTGACAATTTCAACGAACAGAAGAAAAAAACAGgtg    300
```

Susceptible (SUS) laboratory colonies (MW847052–MW847079)

Resistant (RES) field collections (MW847084–MW847146)

			--- intron --		
AgVGSC-H2_cDNA			**		
MW847052:	ISU-B1_1	301	acttattttaaacatatagaatcaattagtattaattattttttttttttaaggtactcaagaaatatcgatataagttcataactatattatgggttacca	400	Susceptible (SUS) laboratory colonies
MW847053:	ISU-B1_5	301	acttattttaaacatatagaatcaattagtattaattattttttttttttaaggtactcaagaaatatcgatataagttcataactatattatgggttacca	400	
MW847054:	ISU-B1_6	301	acttattttaaacatatagaatcaattagtattaattattttttttttttaaggtactcaagaaatatcgatataagttcataactatattatgggttacca	400	
MW847055:	ISU-B1_14	301	acttattttaaacatatagaatcaattagtattaattattttttttttttaaggtactcaagaaatatcgatataagttcataactatattatgggttacca	400	
MW847056:	UIL-B1_2	301	acttattttaaacatatagaatcaattagtattaattattttttttttttaaggtactcaagaaatatcgatataagttcataactatattatgggttacca	400	
MW847057:	UIL-B1_5	301	acttattttaaacatatagaatcaattagtattaattattttttttttttaaggtactcaagaaatatcgatataagttcataactatattatgggttacca	400	
MW847058:	UIL-B1_10	301	acttattttaaacatatagaatcaattagtattaattattttttttttttaaggtactcaagaaatatcgatataagttcataactatattatgggttacca	400	
MW847059:	UIL-B1_19	301	acttattttaaacatatagaatcaattagtattaattattttttttttttaaggtactcaagaaatatcgatataagttcataactatattatgggttacca	400	
MW847068:	ISU-B3_4	301	acttattttaaacatatagaatcaattagtattaattattttttttttttaaggtactcaagaaatatcgatataagttcataactatattatgggttacca	400	
MW847069:	ISU-B3_6	301	acttattttaaacatatagaatcaattagtattaattattttttttttttaaggtactcaagaaatatcgatataagttcataactatattatgggttacca	400	
MW847070:	ISU-B3_8	301	acttattttaaacatatagaatcaattagtattaattattttttttttttaaggtactcaagaaatatcgatataagttcataactatattatgggttacca	400	
MW847071:	ISU-B3_14	301	acttattttaaacatatagaatcaattagtattaattattttttttttttaaggtactcaagaaatatcgatataagttcataactatattatgggttacca	400	
MW847076:	ISU-B4_5	301	acttattttaaacatatagaatcaattagtattaattattttttttttttaaggtactcaagaaatatcgatataagttcataactatattatgggttacca	400	
MW847077:	ISU-B4_8	301	acttattttaaacatatagaatcaattagtattaattattttttttttttaaggtactcaagaaatatcgatataagttcataactatattatgggttacca	400	
MW847078:	ISU-B4_13	301	acttattttaaacatatagaatcaattagtattaattattttttttttttaaggtactcaagaaatatcgatataagttcataactatattatgggttacca	400	
MW847079:	ISU-B4_16	301	acttattttaaacatatagaatcaattagtattaattattttttttttttaaggtactcaagaaatatcgatataagttcataactatattatgggttacca	400	
MW847084:	Suth-1	301	acttattttaaacatatagaatcaattagtattaattattttttttttttaaggtactcaagaaatatcgatataagttcataactatattatgggttacca	400	Resistant (RES) field collections
MW847085:	Suth-2	301	acttattttaaacatatagaatcaattagtattaattattttttttttttaaggtactcaagaaatatcgatataagttcataactatattatgggttacca	400	
MW847086:	Suth-3	301	acttattttaaacatatagaatcaattagtattaattattttttttttttaaggtactcaagaaatatcgatataagttcataactatattatgggttacca	400	
MW847087:	Suth-4	301	acttattttaaacatatagaatcaattagtattaattattttttttttttaaggtactcaagaaatatcgatataagttcataactatattatgggttacca	400	
MW847088:	Suth-5	301	acttattttaaacatatagaatcaattagtattaattattttttttttttaaggtactcaagaaatatcgatataagttcataactatattatgggttacca	400	
MW847089:	Suth-6	301	acttattttaaacatatagaatcaattagtattaattattttttttttttaaggtactcaagaaatatcgatataagttcataactatattatgggttacca	400	
MW847090:	Suth-7	301	acttattttaaacatatagaatcaattagtattaattattttttttttttaaggtactcaagaaatatcgatataagttcataactatattatgggttacca	400	
MW847091:	Suth-8	301	acttattttaaacatatagaatcaattagtattaattattttttttttttaaggtactcaagaaatatcgatataagttcataactatattatgggttacca	400	
MW847092:	Nash-1	301	acttattttaaacatatagaatcaattagtattaattattttttttttttaaggtactcaagaaatatcgatataagttcataactatattatgggttacca	400	
MW847093:	Nash-2	301	acttattttaaacatatagaatcaattagtattaattattttttttttttaaggtactcaagaaatatcgatataagttcataactatattatgggttacca	400	
MW847094:	Nash-3	301	acttattttaaacatatagaatcaattagtattaattattttttttttttaaggtactcaagaaatatcgatataagttcataactatattatgggttacca	400	
MW847095:	Nash-4	301	acttattttaaacatatagaatcaattagtattaattattttttttttttaaggtactcaagaaatatcgatataagttcataactatattatgggttacca	400	
MW847096:	Nash-5	301	acttattttaaacatatagaatcaattagtattaattattttttttttttaaggtactcaagaaatatcgatataagttcataactatattatgggttacca	400	
MW847097:	Nash-6	301	acttattttaaacatatagaatcaattagtattaattattttttttttttaaggtactcaagaaatatcgatataagttcataactatattatgggttacca	400	
MW847098:	Nash-7	301	acttattttaaacatatagaatcaattagtattaattattttttttttttaaggtactcaagaaatatcgatataagttcataactatattatgggttacca	400	
MW847099:	Nash-8	301	acttattttaaacatatagaatcaattagtattaattattttttttttttaaggtactcaagaaatatcgatataagttcataactatattatgggttacca	400	
MW847100:	MN1-1-1	301	acttattttaaacatatagaatcaattagtattaattattttttttttttaaggtactcaagaaatatcgatataagttcataactatattatgggttacca	400	
MW847101:	MN1-1-2	301	acttattttaaacatatagaatcaattagtattaattattttttttttttaaggtactcaagaaatatcgatataagttcataactatattatgggttacca	400	
MW847102:	MN1-1-3	301	acttattttaaacatatagaatcaattagtattaattattttttttttttaaggtactcaagaaatatcgatataagttcataactatattatgggttacca	400	
MW847103:	MN1-1-4	301	acttattttaaacatatagaatcaattagtattaattattttttttttttaaggtactcaagaaatatcgatataagttcataactatattatgggttacca	400	
MW847104:	MN1-2-1	301	acttattttaaacatatagaatcaattagtattaattattttttttttttaaggtactcaagaaatatcgatataagttcataactatattatgggttacca	400	
MW847105:	MN1-2-2	301	acttattttaaacatatagaatcaattagtattaattattttttttttttaaggtactcaagaaatatcgatataagttcataactatattatgggttacca	400	
MW847106:	MN1-2-3	301	acttattttaaacatatagaatcaattagtattaattattttttttttttaaggtactcaagaaatatcgatataagttcataactatattatgggttacca	400	
MW847107:	MN1-2-4	301	acttattttaaacatatagaatcaattagtattaattattttttttttttaaggtactcaagaaatatcgatataagttcataactatattatgggttacca	400	
MW847108:	MN2-1-1	301	acttattttaaacatatagaatcaattagtattaattattttttttttttaaggtactcaagaaatatcgatataagttcataactatattatgggttacca	400	
MW847109:	MN2-1-2	301	acttattttaaacatatagaatcaattagtattaattattttttttttttaaggtactcaagaaatatcgatataagttcataactatattatgggttacca	400	
MW847110:	MN2-1-3	301	acttattttaaacatatagaatcaattagtattaattattttttttttttaaggtactcaagaaatatcgatataagttcataactatattatgggttacca	400	
MW847111:	MN2-2-1	301	acttattttaaacatatagaatcaattagtattaattattttttttttttaaggtactcaagaaatatcgatataagttcataactatattatgggttacca	400	
MW847112:	MN2-2-2	301	acttattttaaacatatagaatcaattagtattaattattttttttttttaaggtactcaagaaatatcgatataagttcataactatattatgggttacca	400	
MW847113:	MN2-2-3	301	acttattttaaacatatagaatcaattagtattaattattttttttttttaaggtactcaagaaatatcgatataagttcataactatattatgggttacca	400	
MW847114:	MN2-2-4	301	acttattttaaacatatagaatcaattagtattaattattttttttttttaaggtactcaagaaatatcgatataagttcataactatattatgggttacca	400	
MW847115:	9R1A-1	301	acttattttaaacatatagaatcaattagtattaattattttttttttttaaggtactcaagaaatatcgatataagttcataactatattatgggttacca	400	
MW847116:	9R2A-1	301	acttattttaaacatatagaatcaattagtattaattattttttttttttaaggtactcaagaaatatcgatataagttcataactatattatgggttacca	400	
MW847117:	9R3A-1	301	acttattttaaacatatagaatcaattagtattaattattttttttttttaaggtactcaagaaatatcgatataagttcataactatattatgggttacca	400	
MW847118:	9R3A-2	301	acttattttaaacatatagaatcaattagtattaattattttttttttttaaggtactcaagaaatatcgatataagttcataactatattatgggttacca	400	
MW847119:	9R1D-1	301	acttattttaaacatatagaatcaattagtattaattattttttttttttaaggtactcaagaaatatcgatataagttcataactatattatgggttacca	400	
MW847120:	9R1D-2	301	acttattttaaacatatagaatcaattagtattaattattttttttttttaaggtactcaagaaatatcgatataagttcataactatattatgggttacca	400	
MW847121:	9R1D-3	301	acttattttaaacatatagaatcaattagtattaattattttttttttttaaggtactcaagaaatatcgatataagttcataactatattatgggttacca	400	
MW847122:	9R1D-5	301	acttattttaaacatatagaatcaattagtattaattattttttttttttaaggtactcaagaaatatcgatataagttcataactatattatgggttacca	400	
MW847123:	9R2D-1	301	acttattttaaacatatagaatcaattagtattaattattttttttttttaaggtactcaagaaatatcgatataagttcataactatattatgggttacca	400	
MW847124:	9R2D-2	301	acttattttaaacatatagaatcaattagtattaattattttttttttttaaggtactcaagaaatatcgatataagttcataactatattatgggttacca	400	
MW847125:	9R2D-3	301	acttattttaaacatatagaatcaattagtattaattattttttttttttaaggtactcaagaaatatcgatataagttcataactatattatgggttacca	400	
MW847126:	9R2D-4	301	acttattttaaacatatagaatcaattagtattaattattttttttttttaaggtactcaagaaatatcgatataagttcataactatattatgggttacca	400	
MW847127:	9R2D-5	301	acttattttaaacatatagaatcaattagtattaattattttttttttttaaggtactcaagaaatatcgatataagttcataactatattatgggttacca	400	
MW847128:	9R2D-6	301	acttattttaaacatatagaatcaattagtattaattattttttttttttaaggtactcaagaaatatcgatataagttcataactatattatgggttacca	400	
MW847129:	9R2D-7	301	acttattttaaacatatagaatcaattagtattaattattttttttttttaaggtactcaagaaatatcgatataagttcataactatattatgggttacca	400	
MW847130:	9R2D-8	301	acttattttaaacatatagaatcaattagtattaattattttttttttttaaggtactcaagaaatatcgatataagttcataactatattatgggttacca	400	
MW847131:	7R3D-1	301	acttattttaaacatatagaatcaattagtattaattattttttttttttaaggtactcaagaaatatcgatataagttcataactatattatgggttacca	400	
MW847132:	7R3D-2	301	acttattttaaacatatagaatcaattagtattaattattttttttttttaaggtactcaagaaatatcgatataagttcataactatattatgggttacca	400	
MW847133:	7R3D-3	301	acttattttaaacatatagaatcaattagtattaattattttttttttttaaggtactcaagaaatatcgatataagttcataactatattatgggttacca	400	
MW847134:	7R3D-4	301	acttattttaaacatatagaatcaattagtattaattattttttttttttaaggtactcaagaaatatcgatataagttcataactatattatgggttacca	400	
MW847135:	7R3D-5	301	acttattttaaacatatagaatcaattagtattaattattttttttttttaaggtactcaagaaatatcgatataagttcataactatattatgggttacca	400	
MW847136:	7R3D-6	301	acttattttaaacatatagaatcaattagtattaattattttttttttttaaggtactcaagaaatatcgatataagttcataactatattatgggttacca	400	
MW847137:	7R3D-7	301	acttattttaaacatatagaatcaattagtattaattattttttttttttaaggtactcaagaaatatcgatataagttcataactatattatgggttacca	400	
MW847138:	7R3D-8	301	acttattttaaacatatagaatcaattagtattaattattttttttttttaaggtactcaagaaatatcgatataagttcataactatattatgggttacca	400	
MW847139:	7R3A-1	301	acttattttaaacatatagaatcaattagtattaattattttttttttttaaggtactcaagaaatatcgatataagttcataactatattatgggttacca	400	
MW847140:	7R3A-2	301	acttattttaaacatatagaatcaattagtattaattattttttttttttaaggtactcaagaaatatcgatataagttcataactatattatgggttacca	400	
MW847141:	7R3A-3	301	acttattttaaacatatagaatcaattagtattaattattttttttttttaaggtactcaagaaatatcgatataagttcataactatattatgggttacca	400	
MW847142:	7R3A-4	301	acttattttaaacatatagaatcaattagtattaattattttttttttttaaggtactcaagaaatatcgatataagttcataactatattatgggttacca	400	
MW847143:	7R3A-5	301	acttattttaaacatatagaatcaattagtattaattattttttttttttaaggtactcaagaaatatcgatataagttcataactatattatgggttacca	400	
MW847144:	7R3A-6	301	acttattttaaacatatagaatcaattagtattaattattttttttttttaaggtactcaagaaatatcgatataagttcataactatattatgggttacca	400	
MW847145:	7R3A-7	301	acttattttaaacatatagaatcaattagtattaattattttttttttttaaggtactcaagaaatatcgatataagttcataactatattatgggttacca	400	
MW847146:	7R2A-1	301	acttattttaaacatatagaatcaattagtattaattattttttttttttaaggtactcaagaaatatcgatataagttcataactatattatgggttacca	400	

		--- intron --->		
AgVGSC-H2_cDNA		**************		
MW847052: ISU-B1_1	401	ttttaaaaacaata	414	Susceptible (SUS) laboratory colonies
MW847053: ISU-B1_5	401	ttttaaaaacaata	414	
MW847054: ISU-B1_6	401	ttttaaaaacaata	414	
MW847055: ISU-B1_14	401	ttttaaaaacaata	414	
MW847056: UIL-B1_2	401	ttttaaaaacaata	414	
MW847057: UIL-B1_5	401	ttttaaaaacaata	414	
MW847058: UIL-B1_10	401	ttttaaaaacaata	414	
MW847059: UIL-B1_19	401	ttttaaaaacaata	414	
MW847068: ISU-B3_4	401	ttttaaaaacaata	414	
MW847069: ISU-B3_6	401	ttttaaaaacaata	414	
MW847070: ISU-B3_8	401	ttttaaaaacaata	414	
MW847071: ISU-B3_14	401	ttttaaaaacaata	414	
MW847076: ISU-B4_5	401	ttttaaaaacaata	414	
MW847077: ISU-B4_8	401	ttttaaaaacaata	414	
MW847078: ISU-B4_13	401	ttttaaaaacaata	414	
MW847079: ISU-B4_16	401	ttttaaaaacaata	414	
MW847084: Suth-1	401	ttttaaaaacaata	414	Resistant (RES) field collections
MW847085: Suth-2	401	ttttaaaaacaata	414	
MW847086: Suth-3	401	ttttaaaaacaata	414	
MW847087: Suth-4	401	ttttaaaaacaata	414	
MW847088: Suth-5	401	ttttaaaaacaata	414	
MW847089: Suth-6	401	ttttaaaaacaata	414	
MW847090: Suth-7	401	ttttaaaaacaata	414	
MW847091: Suth-8	401	ttttaaaaacaata	414	
MW847092: Nash-1	401	ttttaaaaacaata	414	
MW847093: Nash-2	401	ttttaaaaacaata	414	
MW847094: Nash-3	401	ttttaaaaacaata	414	
MW847095: Nash-4	401	ttttaaaaacaata	414	
MW847096: Nash-5	401	ttttaaaaacaata	414	
MW847097: Nash-6	401	ttttaaaaacaata	414	
MW847098: Nash-7	401	ttttaaaaacaata	414	
MW847099: Nash-8	401	ttttaaaaacaata	414	
MW847100: MN1-1-1	401	ttttaaaaacaata	414	
MW847101: MN1-1-2	401	ttttaaaaacaata	414	
MW847102: MN1-1-3	401	ttttaaaaacaata	414	
MW847103: MN1-1-4	401	ttttaaaaacaata	414	
MW847104: MN1-2-1	401	ttttaaaaacaata	414	
MW847105: MN1-2-2	401	ttttaaaaacaata	414	
MW847106: MN1-2-3	401	ttttaaaaacaata	414	
MW847107: MN1-2-4	401	ttttaaaaacaata	414	
MW847108: MN2-1-1	401	ttttaaaaacaata	414	
MW847109: MN2-1-2	401	ttttaaaaacaata	414	
MW847110: MN2-1-3	401	ttttaaaaacaata	414	
MW847111: MN2-2-1	401	ttttaaaaacaata	414	
MW847112: MN2-2-2	401	ttttaaaaacaata	414	
MW847113: MN2-2-3	401	ttttaaaaacaata	414	
MW847114: MN2-2-4	401	ttttaaaaacaata	414	
MW847115: 9R1A-1	401	ttttaaaaacaata	414	
MW847116: 9R2A-1	401	ttttaaaaacaata	414	
MW847117: 9R3A-1	401	ttttaaaaacaata	414	
MW847118: 9R3A-2	401	ttttaaaaacaata	414	
MW847119: 9R1D-1	401	ttttaaaaacaata	414	
MW847120: 9R1D-2	401	ttttaaaaacaata	414	
MW847121: 9R1D-3	401	ttttaaaaacaata	414	
MW847122: 9R1D-5	401	ttttaaaaacaata	414	
MW847123: 9R2D-1	401	ttttaaaaacaata	414	
MW847124: 9R2D-2	401	ttttaaaaacaata	414	
MW847125: 9R2D-3	401	ttttaaaaacaata	414	
MW847126: 9R2D-4	401	ttttaaaaacaata	414	
MW847127: 9R2D-5	401	ttttaaaaacaata	414	
MW847128: 9R2D-6	401	ttttaaaaacaata	414	
MW847129: 9R2D-7	401	ttttaaaaacaata	414	
MW847130: 9R2D-8	401	ttttaaaaacaata	414	
MW847131: 7R3D-1	401	ttttaaaaacaata	414	
MW847132: 7R3D-2	401	ttttaaaaacaata	414	
MW847133: 7R3D-3	401	ttttaaaaacaata	414	
MW847134: 7R3D-4	401	ttttaaaaacaata	414	
MW847135: 7R3D-5	401	ttttaaaaacaata	414	
MW847136: 7R3D-6	401	ttttaaaaacaata	414	
MW847137: 7R3D-7	401	ttttaaaaacaata	414	
MW847138: 7R3D-8	401	ttttaaaaacaata	414	
MW847139: 7R3A-1	401	ttttaaaaacaata	414	
MW847140: 7R3A-2	401	ttttaaaaacaata	414	
MW847141: 7R3A-3	401	ttttaaaaacaata	414	
MW847142: 7R3A-4	401	ttttaaaaacaata	414	
MW847143: 7R3A-5	401	ttttaaaaacaata	414	
MW847144: 7R3A-6	401	ttttaaaaacaata	414	
MW847145: 7R3A-7	401	ttttaaaaacaata	414	
MW847146: 7R2A-1	401	ttttaaaaacaata	414	

Figure S9: Alignment of Sanger sequence fragments from the *Aphis glycines* voltage-gated sodium channel domain IV (DIV) segments S4-S6 (underlined) among individual aphids from resistant (RES) field collections and susceptible laboratory colonies [SUS; biotypes 1, 3 and 4 from Iowa State University (ISU) or University of Illinois (UIL)]. A single synonymous (non-amino acid changing) G to A transition mutation was detected at position 362 of the 536 bp alignment (2118 of the cDNA consensus; **Figure S1**) resulted in a leucine 3rd codon position change from CTG to CTA. The substitution was predicted as a fixed difference between homozygote genotypes. Amino acid orthologous to *Musca domestica* VGSC positions 1752 and 1823 where mutations I1752V and M1823I, respectively, were previously associated with pyrethroid resistance in other insect species (Dong et al., 2014) are enclosed in boxes. Variation at these positions was not detected in *A. glycines*. Aligned sequences named as GenBank accession: clone. Intron sequence is in small caps with canonical 5'-gt and 3'-ag splice junctions underlined, and genomic intron sequence gaps with aligned cDNA in asterisks (*).

```
                                   .......DIV S4.......                                         ...............DIV S5...............
                                     A  K  G  I  R  T  L  L  F  A  L  A  M  S  L  P  A  L  F  N  I  C  L  L  L  F  L  V  M  F  I  A
AgVGSC2_cDNA               1884   GAGCGAAAGGGATTAGAACTCTATTGTTCGCTTTAGCCATGTCACTTCCAGCGTTATTCAACATATGTTTGTTACTGTTTCTTGTCATGTTCATATTTGC   1983
MW846959: ISU-B1_1            1   GAGCGAAAGGGATTAGAACTCTATTGTTCGCTTTAGCCATGTCACTTCCAGCGTTATTCAACATATGTTTGTTACTGTTTCTTGTCATGTTCATATTTGC    100
MW846960: ISU-B1_5            1   GAGCGAAAGGGATTAGAACTCTATTGTTCGCTTTAGCCATGTCACTTCCAGCGTTATTCAACATATGTTTGTTACTGTTTCTTGTCATGTTCATATTTGC    100
MW846961: ISU-B1_6            1   GAGCGAAAGGGATTAGAACTCTATTGTTCGCTTTAGCCATGTCACTTCCAGCGTTATTCAACATATGTTTGTTACTGTTTCTTGTCATGTTCATATTTGC    100
MW846962: UIL-B1_2            1   GAGCGAAAGGGATTAGAACTCTATTGTTCGCTTTAGCCATGTCACTTCCAGCGTTATTCAACATATGTTTGTTACTGTTTCTTGTCATGTTCATATTTGC    100
MW846963: UIL-B1_5            1   GAGCGAAAGGGATTAGAACTCTATTGTTCGCTTTAGCCATGTCACTTCCAGCGTTATTCAACATATGTTTGTTACTGTTTCTTGTCATGTTCATATTTGC    100
MW846964: UIL-B1_10           1   GAGCGAAAGGGATTAGAACTCTATTGTTCGCTTTAGCCATGTCACTTCCAGCGTTATTCAACATATGTTTGTTACTGTTTCTTGTCATGTTCATATTTGC    100
MW846965: UIL-B1_19           1   GAGCGAAAGGGATTAGAACTCTATTGTTCGCTTTAGCCATGTCACTTCCAGCGTTATTCAACATATGTTTGTTACTGTTTCTTGTCATGTTCATATTTGC    100
MW846974: ISU-B3_4            1   GAGCGAAAGGGATTAGAACTCTATTGTTCGCTTTAGCCATGTCACTTCCAGCGTTATTCAACATATGTTTGTTACTGTTTCTTGTCATGTTCATATTTGC    100
MW846975: ISU-B3_6            1   GAGCGAAAGGGATTAGAACTCTATTGTTCGCTTTAGCCATGTCACTTCCAGCGTTATTCAACATATGTTTGTTACTGTTTCTTGTCATGTTCATATTTGC    100
MW846976: ISU-B3_8            1   GAGCGAAAGGGATTAGAACTCTATTGTTCGCTTTAGCCATGTCACTTCCAGCGTTATTCAACATATGTTTGTTACTGTTTCTTGTCATGTTCATATTTGC    100
MW846977: ISU-B3_14           1   GAGCGAAAGGGATTAGAACTCTATTGTTCGCTTTAGCCATGTCACTTCCAGCGTTATTCAACATATGTTTGTTACTGTTTCTTGTCATGTTCATATTTGC    100
MW846982: ISU-B4_5            1   GAGCGAAAGGGATTAGAACTCTATTGTTCGCTTTAGCCATGTCACTTCCAGCGTTATTCAACATATGTTTGTTACTGTTTCTTGTCATGTTCATATTTGC    100
MW846983: ISU-B4_8            1   GAGCGAAAGGGATTAGAACTCTATTGTTCGCTTTAGCCATGTCACTTCCAGCGTTATTCAACATATGTTTGTTACTGTTTCTTGTCATGTTCATATTTGC    100
MW846984: ISU-B4_13           1   GAGCGAAAGGGATTAGAACTCTATTGTTCGCTTTAGCCATGTCACTTCCAGCGTTATTCAACATATGTTTGTTACTGTTTCTTGTCATGTTCATATTTGC    100
MW846985: ISU-B4_16           1   GAGCGAAAGGGATTAGAACTCTATTGTTCGCTTTAGCCATGTCACTTCCAGCGTTATTCAACATATGTTTGTTACTGTTTCTTGTCATGTTCATATTTGC    100
MW846990: Suth-1              1   GAGCGAAAGGGATTAGAACTCTATTGTTCGCTTTAGCCATGTCACTTCCAGCGTTATTCAACATATGTTTGTTACTGTTTCTTGTCATGTTCATATTTGC    100
MW846991: Suth-2              1   GAGCGAAAGGGATTAGAACTCTATTGTTCGCTTTAGCCATGTCACTTCCAGCGTTATTCAACATATGTTTGTTACTGTTTCTTGTCATGTTCATATTTGC    100
MW846992: Suth-3              1   GAGCGAAAGGGATTAGAACTCTATTGTTCGCTTTAGCCATGTCACTTCCAGCGTTATTCAACATATGTTTGTTACTGTTTCTTGTCATGTTCATATTTGC    100
MW846993: Suth-4              1   GAGCGAAAGGGATTAGAACTCTATTGTTCGCTTTAGCCATGTCACTTCCAGCGTTATTCAACATATGTTTGTTACTGTTTCTTGTCATGTTCATATTTGC    100
MW846994: Suth-5              1   GAGCGAAAGGGATTAGAACTCTATTGTTCGCTTTAGCCATGTCACTTCCAGCGTTATTCAACATATGTTTGTTACTGTTTCTTGTCATGTTCATATTTGC    100
MW846995: Suth-6              1   GAGCGAAAGGGATTAGAACTCTATTGTTCGCTTTAGCCATGTCACTTCCAGCGTTATTCAACATATGTTTGTTACTGTTTCTTGTCATGTTCATATTTGC    100
MW846996: Suth-7              1   GAGCGAAAGGGATTAGAACTCTATTGTTCGCTTTAGCCATGTCACTTCCAGCGTTATTCAACATATGTTTGTTACTGTTTCTTGTCATGTTCATATTTGC    100
MW846997: Suth-8              1   GAGCGAAAGGGATTAGAACTCTATTGTTCGCTTTAGCCATGTCACTTCCAGCGTTATTCAACATATGTTTGTTACTGTTTCTTGTCATGTTCATATTTGC    100
MW846998: Nashua-1            1   GAGCGAAAGGGATTAGAACTCTATTGTTCGCTTTAGCCATGTCACTTCCAGCGTTATTCAACATATGTTTGTTACTGTTTCTTGTCATGTTCATATTTGC    100
MW846999: Nashua-2            1   GAGCGAAAGGGATTAGAACTCTATTGTTCGCTTTAGCCATGTCACTTCCAGCGTTATTCAACATATGTTTGTTACTGTTTCTTGTCATGTTCATATTTGC    100
MW847000: Nashua-3            1   GAGCGAAAGGGATTAGAACTCTATTGTTCGCTTTAGCCATGTCACTTCCAGCGTTATTCAACATATGTTTGTTACTGTTTCTTGTCATGTTCATATTTGC    100
MW847001: Nashua-4            1   GAGCGAAAGGGATTAGAACTCTATTGTTCGCTTTAGCCATGTCACTTCCAGCGTTATTCAACATATGTTTGTTACTGTTTCTTGTCATGTTCATATTTGC    100
MW847002: Nashua-5            1   GAGCGAAAGGGATTAGAACTCTATTGTTCGCTTTAGCCATGTCACTTCCAGCGTTATTCAACATATGTTTGTTACTGTTTCTTGTCATGTTCATATTTGC    100
MW847003: Nashua-6            1   GAGCGAAAGGGATTAGAACTCTATTGTTCGCTTTAGCCATGTCACTTCCAGCGTTATTCAACATATGTTTGTTACTGTTTCTTGTCATGTTCATATTTGC    100
MW847004: Nashua-7            1   GAGCGAAAGGGATTAGAACTCTATTGTTCGCTTTAGCCATGTCACTTCCAGCGTTATTCAACATATGTTTGTTACTGTTTCTTGTCATGTTCATATTTGC    100
MW847005: MN1-1-1             1   GAGCGAAAGGGATTAGAACTCTATTGTTCGCTTTAGCCATGTCACTTCCAGCGTTATTCAACATATGTTTGTTACTGTTTCTTGTCATGTTCATATTTGC    100
MW847006: MN1-1-2             1   GAGCGAAAGGGATTAGAACTCTATTGTTCGCTTTAGCCATGTCACTTCCAGCGTTATTCAACATATGTTTGTTACTGTTTCTTGTCATGTTCATATTTGC    100
MW847007: MN1-1-3             1   GAGCGAAAGGGATTAGAACTCTATTGTTCGCTTTAGCCATGTCACTTCCAGCGTTATTCAACATATGTTTGTTACTGTTTCTTGTCATGTTCATATTTGC    100
MW847008: MN1-1-4             1   GAGCGAAAGGGATTAGAACTCTATTGTTCGCTTTAGCCATGTCACTTCCAGCGTTATTCAACATATGTTTGTTACTGTTTCTTGTCATGTTCATATTTGC    100
MW847009: MN1-2-1             1   GAGCGAAAGGGATTAGAACTCTATTGTTCGCTTTAGCCATGTCACTTCCAGCGTTATTCAACATATGTTTGTTACTGTTTCTTGTCATGTTCATATTTGC    100
MW847010: MN1-2-2             1   GAGCGAAAGGGATTAGAACTCTATTGTTCGCTTTAGCCATGTCACTTCCAGCGTTATTCAACATATGTTTGTTACTGTTTCTTGTCATGTTCATATTTGC    100
MW847011: MN1-2-3             1   GAGCGAAAGGGATTAGAACTCTATTGTTCGCTTTAGCCATGTCACTTCCAGCGTTATTCAACATATGTTTGTTACTGTTTCTTGTCATGTTCATATTTGC    100
MW847012: MN1-2-4             1   GAGCGAAAGGGATTAGAACTCTATTGTTCGCTTTAGCCATGTCACTTCCAGCGTTATTCAACATATGTTTGTTACTGTTTCTTGTCATGTTCATATTTGC    100
MW847013: MN2-1-1             1   GAGCGAAAGGGATTAGAACTCTATTGTTCGCTTTAGCCATGTCACTTCCAGCGTTATTCAACATATGTTTGTTACTGTTTCTTGTCATGTTCATATTTGC    100
MW847014: MN2-1-2             1   GAGCGAAAGGGATTAGAACTCTATTGTTCGCTTTAGCCATGTCACTTCCAGCGTTATTCAACATATGTTTGTTACTGTTTCTTGTCATGTTCATATTTGC    100
MW847015: MN2-1-3             1   GAGCGAAAGGGATTAGAACTCTATTGTTCGCTTTAGCCATGTCACTTCCAGCGTTATTCAACATATGTTTGTTACTGTTTCTTGTCATGTTCATATTTGC    100
MW847016: MN2-2-1             1   GAGCGAAAGGGATTAGAACTCTATTGTTCGCTTTAGCCATGTCACTTCCAGCGTTATTCAACATATGTTTGTTACTGTTTCTTGTCATGTTCATATTTGC    100
MW847017: MN2-2-2             1   GAGCGAAAGGGATTAGAACTCTATTGTTCGCTTTAGCCATGTCACTTCCAGCGTTATTCAACATATGTTTGTTACTGTTTCTTGTCATGTTCATATTTGC    100
MW847018: MN2-2-3             1   GAGCGAAAGGGATTAGAACTCTATTGTTCGCTTTAGCCATGTCACTTCCAGCGTTATTCAACATATGTTTGTTACTGTTTCTTGTCATGTTCATATTTGC    100
MW847019: MN2-2-4             1   GAGCGAAAGGGATTAGAACTCTATTGTTCGCTTTAGCCATGTCACTTCCAGCGTTATTCAACATATGTTTGTTACTGTTTCTTGTCATGTTCATATTTGC    100
MW847020: 9R1A-1              1   GAGCGAAAGGGATTAGAACTCTATTGTTCGCTTTAGCCATGTCACTTCCAGCGTTATTCAACATATGTTTGTTACTGTTTCTTGTCATGTTCATATTTGC    100
MW847021: 9R2A-1              1   GAGCGAAAGGGATTAGAACTCTATTGTTCGCTTTAGCCATGTCACTTCCAGCGTTATTCAACATATGTTTGTTACTGTTTCTTGTCATGTTCATATTTGC    100
MW847022: 9R3A-1              1   GAGCGAAAGGGATTAGAACTCTATTGTTCGCTTTAGCCATGTCACTTCCAGCGTTATTCAACATATGTTTGTTACTGTTTCTTGTCATGTTCATATTTGC    100
MW847023: 9R3A-2              1   GAGCGAAAGGGATTAGAACTCTATTGTTCGCTTTAGCCATGTCACTTCCAGCGTTATTCAACATATGTTTGTTACTGTTTCTTGTCATGTTCATATTTGC    100
MW847024: 9R1D-1              1   GAGCGAAAGGGATTAGAACTCTATTGTTCGCTTTAGCCATGTCACTTCCAGCGTTATTCAACATATGTTTGTTACTGTTTCTTGTCATGTTCATATTTGC    100
MW847025: 9R1D-2              1   GAGCGAAAGGGATTAGAACTCTATTGTTCGCTTTAGCCATGTCACTTCCAGCGTTATTCAACATATGTTTGTTACTGTTTCTTGTCATGTTCATATTTGC    100
MW847026: 9R1D-3              1   GAGCGAAAGGGATTAGAACTCTATTGTTCGCTTTAGCCATGTCACTTCCAGCGTTATTCAACATATGTTTGTTACTGTTTCTTGTCATGTTCATATTTGC    100
MW847027: 9R1D-5              1   GAGCGAAAGGGATTAGAACTCTATTGTTCGCTTTAGCCATGTCACTTCCAGCGTTATTCAACATATGTTTGTTACTGTTTCTTGTCATGTTCATATTTGC    100
MW847028: 9R2D-1              1   GAGCGAAAGGGATTAGAACTCTATTGTTCGCTTTAGCCATGTCACTTCCAGCGTTATTCAACATATGTTTGTTACTGTTTCTTGTCATGTTCATATTTGC    100
MW847029: 9R2D-2              1   GAGCGAAAGGGATTAGAACTCTATTGTTCGCTTTAGCCATGTCACTTCCAGCGTTATTCAACATATGTTTGTTACTGTTTCTTGTCATGTTCATATTTGC    100
MW847030: 9R2D-3              1   GAGCGAAAGGGATTAGAACTCTATTGTTCGCTTTAGCCATGTCACTTCCAGCGTTATTCAACATATGTTTGTTACTGTTTCTTGTCATGTTCATATTTGC    100
MW847031: 9R2D-4              1   GAGCGAAAGGGATTAGAACTCTATTGTTCGCTTTAGCCATGTCACTTCCAGCGTTATTCAACATATGTTTGTTACTGTTTCTTGTCATGTTCATATTTGC    100
MW847032: 9R2D-5              1   GAGCGAAAGGGATTAGAACTCTATTGTTCGCTTTAGCCATGTCACTTCCAGCGTTATTCAACATATGTTTGTTACTGTTTCTTGTCATGTTCATATTTGC    100
MW847033: 9R2D-6              1   GAGCGAAAGGGATTAGAACTCTATTGTTCGCTTTAGCCATGTCACTTCCAGCGTTATTCAACATATGTTTGTTACTGTTTCTTGTCATGTTCATATTTGC    100
MW847034: 9R2D-7              1   GAGCGAAAGGGATTAGAACTCTATTGTTCGCTTTAGCCATGTCACTTCCAGCGTTATTCAACATATGTTTGTTACTGTTTCTTGTCATGTTCATATTTGC    100
MW847035: 9R2D-8              1   GAGCGAAAGGGATTAGAACTCTATTGTTCGCTTTAGCCATGTCACTTCCAGCGTTATTCAACATATGTTTGTTACTGTTTCTTGTCATGTTCATATTTGC    100
MW847036: 7R3D-1              1   GAGCGAAAGGGATTAGAACTCTATTGTTCGCTTTAGCCATGTCACTTCCAGCGTTATTCAACATATGTTTGTTACTGTTTCTTGTCATGTTCATATTTGC    100
MW847037: 7R3D-2              1   GAGCGAAAGGGATTAGAACTCTATTGTTCGCTTTAGCCATGTCACTTCCAGCGTTATTCAACATATGTTTGTTACTGTTTCTTGTCATGTTCATATTTGC    100
MW847038: 7R3D-3              1   GAGCGAAAGGGATTAGAACTCTATTGTTCGCTTTAGCCATGTCACTTCCAGCGTTATTCAACATATGTTTGTTACTGTTTCTTGTCATGTTCATATTTGC    100
MW847039: 7R3D-4              1   GAGCGAAAGGGATTAGAACTCTATTGTTCGCTTTAGCCATGTCACTTCCAGCGTTATTCAACATATGTTTGTTACTGTTTCTTGTCATGTTCATATTTGC    100
MW847040: 7R3D-5              1   GAGCGAAAGGGATTAGAACTCTATTGTTCGCTTTAGCCATGTCACTTCCAGCGTTATTCAACATATGTTTGTTACTGTTTCTTGTCATGTTCATATTTGC    100
MW847041: 7R3D-6              1   GAGCGAAAGGGATTAGAACTCTATTGTTCGCTTTAGCCATGTCACTTCCAGCGTTATTCAACATATGTTTGTTACTGTTTCTTGTCATGTTCATATTTGC    100
MW847042: 7R3D-7              1   GAGCGAAAGGGATTAGAACTCTATTGTTCGCTTTAGCCATGTCACTTCCAGCGTTATTCAACATATGTTTGTTACTGTTTCTTGTCATGTTCATATTTGC    100
MW847043: 7R3D-8              1   GAGCGAAAGGGATTAGAACTCTATTGTTCGCTTTAGCCATGTCACTTCCAGCGTTATTCAACATATGTTTGTTACTGTTTCTTGTCATGTTCATATTTGC    100
MW847044: 7R3A-1              1   GAGCGAAAGGGATTAGAACTCTATTGTTCGCTTTAGCCATGTCACTTCCAGCGTTATTCAACATATGTTTGTTACTGTTTCTTGTCATGTTCATATTTGC    100
MW847045: 7R3A-2              1   GAGCGAAAGGGATTAGAACTCTATTGTTCGCTTTAGCCATGTCACTTCCAGCGTTATTCAACATATGTTTGTTACTGTTTCTTGTCATGTTCATATTTGC    100
MW847046: 7R3A-3              1   GAGCGAAAGGGATTAGAACTCTATTGTTCGCTTTAGCCATGTCACTTCCAGCGTTATTCAACATATGTTTGTTACTGTTTCTTGTCATGTTCATATTTGC    100
MW847047: 7R3A-4              1   GAGCGAAAGGGATTAGAACTCTATTGTTCGCTTTAGCCATGTCACTTCCAGCGTTATTCAACATATGTTTGTTACTGTTTCTTGTCATGTTCATATTTGC    100
MW847048: 7R3A-5              1   GAGCGAAAGGGATTAGAACTCTATTGTTCGCTTTAGCCATGTCACTTCCAGCGTTATTCAACATATGTTTGTTACTGTTTCTTGTCATGTTCATATTTGC    100
MW847049: 7R3A-6              1   GAGCGAAAGGGATTAGAACTCTATTGTTCGCTTTAGCCATGTCACTTCCAGCGTTATTCAACATATGTTTGTTACTGTTTCTTGTCATGTTCATATTTGC    100
MW847050: 7R3A-7              1   GAGCGAAAGGGATTAGAACTCTATTGTTCGCTTTAGCCATGTCACTTCCAGCGTTATTCAACATATGTTTGTTACTGTTTCTTGTCATGTTCATATTTGC    100
MW847051: 7R2A-1              1   GAGCGAAAGGGATTAGAACTCTATTGTTCGCTTTAGCCATGTCACTTCCAGCGTTATTCAACATATGTTTGTTACTGTTTCTTGTCATGTTCATATTTGC    100
```

Susceptible (SUS) laboratory colonies (MW846959 – MW846985)

Resistant (RES) field collections (MW846990 – MW847051)

```
                                   I1752V
                                   ..........DIV S5...............
                                   [I] F  G  M  S  F  F  M  N  V  D  N  H  G  G  L  D  E  D  Y  N  F  R  T  F  G  Q  S  M  I  L  L  F
AgVGSC2_cDNA              1984  TATATTTGGCATGTCGTTTTTCATGAACGTAGATAATCATGGAGGTTTGGATGAGGATTACAATTTCCGCACTTTCGGGCAATCGATGATACTTCTTTTC  1083
MW846959: ISU-B1_1         101  TATATTTGGCATGTCGTTTTTCATGAACGTAGATAATCATGGAGGTTTGGATGAGGATTACAATTTCCGCACTTTCGGGCAATCGATGATACTTCTTTTC  200
MW846960: ISU-B1_5         101  TATATTTGGCATGTCGTTTTTCATGAACGTAGATAATCATGGAGGTTTGGATGAGGATTACAATTTCCGCACTTTCGGGCAATCGATGATACTTCTTTTC  200
MW846961: ISU-B1_6         101  TATATTTGGCATGTCGTTTTTCATGAACGTAGATAATCATGGAGGTTTGGATGAGGATTACAATTTCCGCACTTTCGGGCAATCGATGATACTTCTTTTC  200
MW846962: UIL-B1_2         101  TATATTTGGCATGTCGTTTTTCATGAACGTAGATAATCATGGAGGTTTGGATGAGGATTACAATTTCCGCACTTTCGGGCAATCGATGATACTTCTTTTC  200
MW846963: UIL-B1_5         101  TATATTTGGCATGTCGTTTTTCATGAACGTAGATAATCATGGAGGTTTGGATGAGGATTACAATTTCCGCACTTTCGGGCAATCGATGATACTTCTTTTC  200
MW846964: UIL-B1_10        101  TATATTTGGCATGTCGTTTTTCATGAACGTAGATAATCATGGAGGTTTGGATGAGGATTACAATTTCCGCACTTTCGGGCAATCGATGATACTTCTTTTC  200
MW846965: UIL-B1_19        101  TATATTTGGCATGTCGTTTTTCATGAACGTAGATAATCATGGAGGTTTGGATGAGGATTACAATTTCCGCACTTTCGGGCAATCGATGATACTTCTTTTC  200
MW846974: ISU-B3_4         101  TATATTTGGCATGTCGTTTTTCATGAACGTAGATAATCATGGAGGTTTGGATGAGGATTACAATTTCCGCACTTTCGGGCAATCGATGATACTTCTTTTC  200
MW846975: ISU-B3_6         101  TATATTTGGCATGTCGTTTTTCATGAACGTAGATAATCATGGAGGTTTGGATGAGGATTACAATTTCCGCACTTTCGGGCAATCGATGATACTTCTTTTC  200
MW846976: ISU-B3_8         101  TATATTTGGCATGTCGTTTTTCATGAACGTAGATAATCATGGAGGTTTGGATGAGGATTACAATTTCCGCACTTTCGGGCAATCGATGATACTTCTTTTC  200
MW846977: ISU-B3_14        101  TATATTTGGCATGTCGTTTTTCATGAACGTAGATAATCATGGAGGTTTGGATGAGGATTACAATTTCCGCACTTTCGGGCAATCGATGATACTTCTTTTC  200
MW846982: ISU-B4_5         101  TATATTTGGCATGTCGTTTTTCATGAACGTAGATAATCATGGAGGTTTGGATGAGGATTACAATTTCCGCACTTTCGGGCAATCGATGATACTTCTTTTC  200
MW846983: ISU-B4_8         101  TATATTTGGCATGTCGTTTTTCATGAACGTAGATAATCATGGAGGTTTGGATGAGGATTACAATTTCCGCACTTTCGGGCAATCGATGATACTTCTTTTC  200
MW846984: ISU-B4_13        101  TATATTTGGCATGTCGTTTTTCATGAACGTAGATAATCATGGAGGTTTGGATGAGGATTACAATTTCCGCACTTTCGGGCAATCGATGATACTTCTTTTC  200
MW846985: ISU-B4_16        101  TATATTTGGCATGTCGTTTTTCATGAACGTAGATAATCATGGAGGTTTGGATGAGGATTACAATTTCCGCACTTTCGGGCAATCGATGATACTTCTTTTC  200
MW846990: Suth-1           101  TATATTTGGCATGTCGTTTTTCATGAACGTAGATAATCATGGAGGTTTGGATGAGGATTACAATTTCCGCACTTTCGGGCAATCGATGATACTTCTTTTC  200
MW846991: Suth-2           101  TATATTTGGCATGTCGTTTTTCATGAACGTAGATAATCATGGAGGTTTGGATGAGGATTACAATTTCCGCACTTTCGGGCAATCGATGATACTTCTTTTC  200
MW846992: Suth-3           101  TATATTTGGCATGTCGTTTTTCATGAACGTAGATAATCATGGAGGTTTGGATGAGGATTACAATTTCCGCACTTTCGGGCAATCGATGATACTTCTTTTC  200
MW846993: Suth-4           101  TATATTTGGCATGTCGTTTTTCATGAACGTAGATAATCATGGAGGTTTGGATGAGGATTACAATTTCCGCACTTTCGGGCAATCGATGATACTTCTTTTC  200
MW846994: Suth-5           101  TATATTTGGCATGTCGTTTTTCATGAACGTAGATAATCATGGAGGTTTGGATGAGGATTACAATTTCCGCACTTTCGGGCAATCGATGATACTTCTTTTC  200
MW846995: Suth-6           101  TATATTTGGCATGTCGTTTTTCATGAACGTAGATAATCATGGAGGTTTGGATGAGGATTACAATTTCCGCACTTTCGGGCAATCGATGATACTTCTTTTC  200
MW846996: Suth-7           101  TATATTTGGCATGTCGTTTTTCATGAACGTAGATAATCATGGAGGTTTGGATGAGGATTACAATTTCCGCACTTTCGGGCAATCGATGATACTTCTTTTC  200
MW846997: Suth-8           101  TATATTTGGCATGTCGTTTTTCATGAACGTAGATAATCATGGAGGTTTGGATGAGGATTACAATTTCCGCACTTTCGGGCAATCGATGATACTTCTTTTC  200
MW846998: Nashua-1         101  TATATTTGGCATGTCGTTTTTCATGAACGTAGATAATCATGGAGGTTTGGATGAGGATTACAATTTCCGCACTTTCGGGCAATCGATGATACTTCTTTTC  200
MW846999: Nashua-2         101  TATATTTGGCATGTCGTTTTTCATGAACGTAGATAATCATGGAGGTTTGGATGAGGATTACAATTTCCGCACTTTCGGGCAATCGATGATACTTCTTTTC  200
MW847000: Nashua-3         101  TATATTTGGCATGTCGTTTTTCATGAACGTAGATAATCATGGAGGTTTGGATGAGGATTACAATTTCCGCACTTTCGGGCAATCGATGATACTTCTTTTC  200
MW847001: Nashua-4         101  TATATTTGGCATGTCGTTTTTCATGAACGTAGATAATCATGGAGGTTTGGATGAGGATTACAATTTCCGCACTTTCGGGCAATCGATGATACTTCTTTTC  200
MW847002: Nashua-5         101  TATATTTGGCATGTCGTTTTTCATGAACGTAGATAATCATGGAGGTTTGGATGAGGATTACAATTTCCGCACTTTCGGGCAATCGATGATACTTCTTTTC  200
MW847003: Nashua-6         101  TATATTTGGCATGTCGTTTTTCATGAACGTAGATAATCATGGAGGTTTGGATGAGGATTACAATTTCCGCACTTTCGGGCAATCGATGATACTTCTTTTC  200
MW847004: Nashua-7         101  TATATTTGGCATGTCGTTTTTCATGAACGTAGATAATCATGGAGGTTTGGATGAGGATTACAATTTCCGCACTTTCGGGCAATCGATGATACTTCTTTTC  200
MW847005: MN1-1-1          101  TATATTTGGCATGTCGTTTTTCATGAACGTAGATAATCATGGAGGTTTGGATGAGGATTACAATTTCCGCACTTTCGGGCAATCGATGATACTTCTTTTC  200
MW847006: MN1-1-2          101  TATATTTGGCATGTCGTTTTTCATGAACGTAGATAATCATGGAGGTTTGGATGAGGATTACAATTTCCGCACTTTCGGGCAATCGATGATACTTCTTTTC  200
MW847007: MN1-1-3          101  TATATTTGGCATGTCGTTTTTCATGAACGTAGATAATCATGGAGGTTTGGATGAGGATTACAATTTCCGCACTTTCGGGCAATCGATGATACTTCTTTTC  200
MW847008: MN1-1-4          101  TATATTTGGCATGTCGTTTTTCATGAACGTAGATAATCATGGAGGTTTGGATGAGGATTACAATTTCCGCACTTTCGGGCAATCGATGATACTTCTTTTC  200
MW847009: MN1-2-1          101  TATATTTGGCATGTCGTTTTTCATGAACGTAGATAATCATGGAGGTTTGGATGAGGATTACAATTTCCGCACTTTCGGGCAATCGATGATACTTCTTTTC  200
MW847010: MN1-2-2          101  TATATTTGGCATGTCGTTTTTCATGAACGTAGATAATCATGGAGGTTTGGATGAGGATTACAATTTCCGCACTTTCGGGCAATCGATGATACTTCTTTTC  200
MW847011: MN1-2-3          101  TATATTTGGCATGTCGTTTTTCATGAACGTAGATAATCATGGAGGTTTGGATGAGGATTACAATTTCCGCACTTTCGGGCAATCGATGATACTTCTTTTC  200
MW847012: MN1-2-4          101  TATATTTGGCATGTCGTTTTTCATGAACGTAGATAATCATGGAGGTTTGGATGAGGATTACAATTTCCGCACTTTCGGGCAATCGATGATACTTCTTTTC  200
MW847013: MN2-1-1          101  TATATTTGGCATGTCGTTTTTCATGAACGTAGATAATCATGGAGGTTTGGATGAGGATTACAATTTCCGCACTTTCGGGCAATCGATGATACTTCTTTTC  200
MW847014: MN2-1-2          101  TATATTTGGCATGTCGTTTTTCATGAACGTAGATAATCATGGAGGTTTGGATGAGGATTACAATTTCCGCACTTTCGGGCAATCGATGATACTTCTTTTC  200
MW847015: MN2-1-3          101  TATATTTGGCATGTCGTTTTTCATGAACGTAGATAATCATGGAGGTTTGGATGAGGATTACAATTTCCGCACTTTCGGGCAATCGATGATACTTCTTTTC  200
MW847016: MN2-2-1          101  TATATTTGGCATGTCGTTTTTCATGAACGTAGATAATCATGGAGGTTTGGATGAGGATTACAATTTCCGCACTTTCGGGCAATCGATGATACTTCTTTTC  200
MW847017: MN2-2-2          101  TATATTTGGCATGTCGTTTTTCATGAACGTAGATAATCATGGAGGTTTGGATGAGGATTACAATTTCCGCACTTTCGGGCAATCGATGATACTTCTTTTC  200
MW847018: MN2-2-3          101  TATATTTGGCATGTCGTTTTTCATGAACGTAGATAATCATGGAGGTTTGGATGAGGATTACAATTTCCGCACTTTCGGGCAATCGATGATACTTCTTTTC  200
MW847019: MN2-2-4          101  TATATTTGGCATGTCGTTTTTCATGAACGTAGATAATCATGGAGGTTTGGATGAGGATTACAATTTCCGCACTTTCGGGCAATCGATGATACTTCTTTTC  200
MW847020: 9R1A-1           101  TATATTTGGCATGTCGTTTTTCATGAACGTAGATAATCATGGAGGTTTGGATGAGGATTACAATTTCCGCACTTTCGGGCAATCGATGATACTTCTTTTC  200
MW847021: 9R2A-1           101  TATATTTGGCATGTCGTTTTTCATGAACGTAGATAATCATGGAGGTTTGGATGAGGATTACAATTTCCGCACTTTCGGGCAATCGATGATACTTCTTTTC  200
MW847022: 9R3A-1           101  TATATTTGGCATGTCGTTTTTCATGAACGTAGATAATCATGGAGGTTTGGATGAGGATTACAATTTCCGCACTTTCGGGCAATCGATGATACTTCTTTTC  200
MW847023: 9R3A-2           101  TATATTTGGCATGTCGTTTTTCATGAACGTAGATAATCATGGAGGTTTGGATGAGGATTACAATTTCCGCACTTTCGGGCAATCGATGATACTTCTTTTC  200
MW847024: 9R1D-1           101  TATATTTGGCATGTCGTTTTTCATGAACGTAGATAATCATGGAGGTTTGGATGAGGATTACAATTTCCGCACTTTCGGGCAATCGATGATACTTCTTTTC  200
MW847025: 9R1D-2           101  TATATTTGGCATGTCGTTTTTCATGAACGTAGATAATCATGGAGGTTTGGATGAGGATTACAATTTCCGCACTTTCGGGCAATCGATGATACTTCTTTTC  200
MW847026: 9R1D-3           101  TATATTTGGCATGTCGTTTTTCATGAACGTAGATAATCATGGAGGTTTGGATGAGGATTACAATTTCCGCACTTTCGGGCAATCGATGATACTTCTTTTC  200
MW847027: 9R1D-5           101  TATATTTGGCATGTCGTTTTTCATGAACGTAGATAATCATGGAGGTTTGGATGAGGATTACAATTTCCGCACTTTCGGGCAATCGATGATACTTCTTTTC  200
MW847028: 9R2D-1           101  TATATTTGGCATGTCGTTTTTCATGAACGTAGATAATCATGGAGGTTTGGATGAGGATTACAATTTCCGCACTTTCGGGCAATCGATGATACTTCTTTTC  200
MW847029: 9R2D-2           101  TATATTTGGCATGTCGTTTTTCATGAACGTAGATAATCATGGAGGTTTGGATGAGGATTACAATTTCCGCACTTTCGGGCAATCGATGATACTTCTTTTC  200
MW847030: 9R2D-3           101  TATATTTGGCATGTCGTTTTTCATGAACGTAGATAATCATGGAGGTTTGGATGAGGATTACAATTTCCGCACTTTCGGGCAATCGATGATACTTCTTTTC  200
MW847031: 9R2D-4           101  TATATTTGGCATGTCGTTTTTCATGAACGTAGATAATCATGGAGGTTTGGATGAGGATTACAATTTCCGCACTTTCGGGCAATCGATGATACTTCTTTTC  200
MW847032: 9R2D-5           101  TATATTTGGCATGTCGTTTTTCATGAACGTAGATAATCATGGAGGTTTGGATGAGGATTACAATTTCCGCACTTTCGGGCAATCGATGATACTTCTTTTC  200
MW847033: 9R2D-6           101  TATATTTGGCATGTCGTTTTTCATGAACGTAGATAATCATGGAGGTTTGGATGAGGATTACAATTTCCGCACTTTCGGGCAATCGATGATACTTCTTTTC  200
MW847034: 9R2D-7           101  TATATTTGGCATGTCGTTTTTCATGAACGTAGATAATCATGGAGGTTTGGATGAGGATTACAATTTCCGCACTTTCGGGCAATCGATGATACTTCTTTTC  200
MW847035: 9R2D-8           101  TATATTTGGCATGTCGTTTTTCATGAACGTAGATAATCATGGAGGTTTGGATGAGGATTACAATTTCCGCACTTTCGGGCAATCGATGATACTTCTTTTC  200
MW847036: 7R3D-1           101  TATATTTGGCATGTCGTTTTTCATGAACGTAGATAATCATGGAGGTTTGGATGAGGATTACAATTTCCGCACTTTCGGGCAATCGATGATACTTCTTTTC  200
MW847037: 7R3D-2           101  TATATTTGGCATGTCGTTTTTCATGAACGTAGATAATCATGGAGGTTTGGATGAGGATTACAATTTCCGCACTTTCGGGCAATCGATGATACTTCTTTTC  200
MW847038: 7R3D-3           101  TATATTTGGCATGTCGTTTTTCATGAACGTAGATAATCATGGAGGTTTGGATGAGGATTACAATTTCCGCACTTTCGGGCAATCGATGATACTTCTTTTC  200
MW847039: 7R3D-4           101  TATATTTGGCATGTCGTTTTTCATGAACGTAGATAATCATGGAGGTTTGGATGAGGATTACAATTTCCGCACTTTCGGGCAATCGATGATACTTCTTTTC  200
MW847040: 7R3D-5           101  TATATTTGGCATGTCGTTTTTCATGAACGTAGATAATCATGGAGGTTTGGATGAGGATTACAATTTCCGCACTTTCGGGCAATCGATGATACTTCTTTTC  200
MW847041: 7R3D-6           101  TATATTTGGCATGTCGTTTTTCATGAACGTAGATAATCATGGAGGTTTGGATGAGGATTACAATTTCCGCACTTTCGGGCAATCGATGATACTTCTTTTC  200
MW847042: 7R3D-7           101  TATATTTGGCATGTCGTTTTTCATGAACGTAGATAATCATGGAGGTTTGGATGAGGATTACAATTTCCGCACTTTCGGGCAATCGATGATACTTCTTTTC  200
MW847043: 7R3D-8           101  TATATTTGGCATGTCGTTTTTCATGAACGTAGATAATCATGGAGGTTTGGATGAGGATTACAATTTCCGCACTTTCGGGCAATCGATGATACTTCTTTTC  200
MW847044: 7R3A-1           101  TATATTTGGCATGTCGTTTTTCATGAACGTAGATAATCATGGAGGTTTGGATGAGGATTACAATTTCCGCACTTTCGGGCAATCGATGATACTTCTTTTC  200
MW847045: 7R3A-2           101  TATATTTGGCATGTCGTTTTTCATGAACGTAGATAATCATGGAGGTTTGGATGAGGATTACAATTTCCGCACTTTCGGGCAATCGATGATACTTCTTTTC  200
MW847046: 7R3A-3           101  TATATTTGGCATGTCGTTTTTCATGAACGTAGATAATCATGGAGGTTTGGATGAGGATTACAATTTCCGCACTTTCGGGCAATCGATGATACTTCTTTTC  200
MW847047: 7R3A-4           101  TATATTTGGCATGTCGTTTTTCATGAACGTAGATAATCATGGAGGTTTGGATGAGGATTACAATTTCCGCACTTTCGGGCAATCGATGATACTTCTTTTC  200
MW847048: 7R3A-5           101  TATATTTGGCATGTCGTTTTTCATGAACGTAGATAATCATGGAGGTTTGGATGAGGATTACAATTTCCGCACTTTCGGGCAATCGATGATACTTCTTTTC  200
MW847049: 7R3A-6           101  TATATTTGGCATGTCGTTTTTCATGAACGTAGATAATCATGGAGGTTTGGATGAGGATTACAATTTCCGCACTTTCGGGCAATCGATGATACTTCTTTTC  200
MW847050: 7R3A-7           101  TATATTTGGCATGTCGTTTTTCATGAACGTAGATAATCATGGAGGTTTGGATGAGGATTACAATTTCCGCACTTTCGGGCAATCGATGATACTTCTTTTC  200
MW847051: 7R2A-1           101  TATATTTGGCATGTCGTTTTTCATGAACGTAGATAATCATGGAGGTTTGGATGAGGATTACAATTTCCGCACTTTCGGGCAATCGATGATACTTCTTTTC  200
```

Susceptible (SUS) laboratory colonies (MW846959–MW846985)

Resistant (RES) field collections (MW846990–MW847051)

Sequence	Start	Alignment	End	Group
		M |-- intron --		
AgVGSC2_cDNA	2083	ATG***	2085	
MW846959: ISU-B1_1	201	ATGgtaagcttataatcggtcctatcaatgtcacgcctgatgaaaatagtgattctataatattttagtgtgccataataatatcacctagtctatcagg	300	Susceptible (SUS) laboratory colonies
MW846960: ISU-B1_5	201	ATGgtaagcttataatcggtcctatcaatgtcacgcctgatgaaaatagtgattctataatattttagtgtgccataataatatcacctagtctatcagg	300	
MW846961: ISU-B1_6	201	ATGgtaagcttataatcggtcctatcaatgtcacgcctgatgaaaatagtgattctataatattttagtgtgccataataatatcacctagtctatcagg	300	
MW846962: UIL-B1_2	201	ATGgtaagcttataatcggtcctatcaatgtcacgcctgatgaaaatagtgattctataatattttagtgtgccataataatatcacctagtctatcagg	300	
MW846963: UIL-B1_5	201	ATGgtaagcttataatcggtcctatcaatgtcacgcctgatgaaaatagtgattctataatattttagtgtgccataataatatcacctagtctatcagg	300	
MW846964: UIL-B1_10	201	ATGgtaagcttataatcggtcctatcaatgtcacgcctgatgaaaatagtgattctataatattttagtgtgccataataatatcacctagtctatcagg	300	
MW846965: UIL-B1_19	201	ATGgtaagcttataatcggtcctatcaatgtcacgcctgatgaaaatagtgattctataatattttagtgtgccataataatatcacctagtctatcagg	300	
MW846974: ISU-B3_4	201	ATGgtaagcttataatcggtcctatcaatgtcacgcctgatgaaaatagtgattctataatattttagtgtgccataataatatcacctagtctatcagg	300	
MW846975: ISU-B3_6	201	ATGgtaagcttataatcggtcctatcaatgtcacgcctgatgaaaatagtgattctataatattttagtgtgccataataatatcacctagtctatcagg	300	
MW846976: ISU-B3_8	201	ATGgtaagcttataatcggtcctatcaatgtcacgcctgatgaaaatagtgattctataatattttagtgtgccataataatatcacctagtctatcagg	300	
MW846977: ISU-B3_14	201	ATGgtaagcttataatcggtcctatcaatgtcacgcctgatgaaaatagtgattctataatattttagtgtgccataataatatcacctagtctatcagg	300	
MW846982: ISU-B4_5	201	ATGgtaagcttataatcggtcctatcaatgtcacgcctgatgaaaatagtgattctataatattttagtgtgccataataatatcacctagtctatcagg	300	
MW846983: ISU-B4_8	201	ATGgtaagcttataatcggtcctatcaatgtcacgcctgatgaaaatagtgattctataatattttagtgtgccataataatatcacctagtctatcagg	300	
MW846984: ISU-B4_13	201	ATGgtaagcttataatcggtcctatcaatgtcacgcctgatgaaaatagtgattctataatattttagtgtgccataataatatcacctagtctatcagg	300	
MW846985: ISU-B4_16	201	ATGgtaagcttataatcggtcctatcaatgtcacgcctgatgaaaatagtgattctataatattttagtgtgccataataatatcacctagtctatcagg	300	
MW846990: Suth-1	201	ATGgtaagcttataatcggtcctatcaatgtcacgcctgatgaaaatagtgattctataatattttagtgtgccataataatatcacctagtctatcagg	300	Resistant (RES) field collections
MW846991: Suth-2	201	ATGgtaagcttataatcggtcctatcaatgtcacgcctgatgaaaatagtgattctataatattttagtgtgccataataatatcacctagtctatcagg	300	
MW846992: Suth-3	201	ATGgtaagcttataatcggtcctatcaatgtcacgcctgatgaaaatagtgattctataatattttagtgtgccataataatatcacctagtctatcagg	300	
MW846993: Suth-4	201	ATGgtaagcttataatcggtcctatcaatgtcacgcctgatgaaaatagtgattctataatattttagtgtgccataataatatcacctagtctatcagg	300	
MW846994: Suth-5	201	ATGgtaagcttataatcggtcctatcaatgtcacgcctgatgaaaatagtgattctataatattttagtgtgccataataatatcacctagtctatcagg	300	
MW846995: Suth-6	201	ATGgtaagcttataatcggtcctatcaatgtcacgcctgatgaaaatagtgattctataatattttagtgtgccataataatatcacctagtctatcagg	300	
MW846996: Suth-7	201	ATGgtaagcttataatcggtcctatcaatgtcacgcctgatgaaaatagtgattctataatattttagtgtgccataataatatcacctagtctatcagg	300	
MW846997: Suth-8	201	ATGgtaagcttataatcggtcctatcaatgtcacgcctgatgaaaatagtgattctataatattttagtgtgccataataatatcacctagtctatcagg	300	
MW846998: Nashua-1	201	ATGgtaagcttataatcggtcctatcaatgtcacgcctgatgaaaatagtgattctataatattttagtgtgccataataatatcacctagtctatcagg	300	
MW846999: Nashua-2	201	ATGgtaagcttataatcggtcctatcaatgtcacgcctgatgaaaatagtgattctataatattttagtgtgccataataatatcacctagtctatcagg	300	
MW847000: Nashua-3	201	ATGgtaagcttataatcggtcctatcaatgtcacgcctgatgaaaatagtgattctataatattttagtgtgccataataatatcacctagtctatcagg	300	
MW847001: Nashua-4	201	ATGgtaagcttataatcggtcctatcaatgtcacgcctgatgaaaatagtgattctataatattttagtgtgccataataatatcacctagtctatcagg	300	
MW847002: Nashua-5	201	ATGgtaagcttataatcggtcctatcaatgtcacgcctgatgaaaatagtgattctataatattttagtgtgccataataatatcacctagtctatcagg	300	
MW847003: Nashua-6	201	ATGgtaagcttataatcggtcctatcaatgtcacgcctgatgaaaatagtgattctataatattttagtgtgccataataatatcacctagtctatcagg	300	
MW847004: Nashua-7	201	ATGgtaagcttataatcggtcctatcaatgtcacgcctgatgaaaatagtgattctataatattttagtgtgccataataatatcacctagtctatcagg	300	
MW847005: MN1-1-1	201	ATGgtaagcttataatcggtcctatcaatgtcacgcctgatgaaaatagtgattctataatattttagtgtgccataataatatcacctagtctatcagg	300	
MW847006: MN1-1-2	201	ATGgtaagcttataatcggtcctatcaatgtcacgcctgatgaaaatagtgattctataatattttagtgtgccataataatatcacctagtctatcagg	300	
MW847007: MN1-1-3	201	ATGgtaagcttataatcggtcctatcaatgtcacgcctgatgaaaatagtgattctataatattttagtgtgccataataatatcacctagtctatcagg	300	
MW847008: MN1-1-4	201	ATGgtaagcttataatcggtcctatcaatgtcacgcctgatgaaaatagtgattctataatattttagtgtgccataataatatcacctagtctatcagg	300	
MW847009: MN1-2-1	201	ATGgtaagcttataatcggtcctatcaatgtcacgcctgatgaaaatagtgattctataatattttagtgtgccataataatatcacctagtctatcagg	300	
MW847010: MN1-2-2	201	ATGgtaagcttataatcggtcctatcaatgtcacgcctgatgaaaatagtgattctataatattttagtgtgccataataatatcacctagtctatcagg	300	
MW847011: MN1-2-3	201	ATGgtaagcttataatcggtcctatcaatgtcacgcctgatgaaaatagtgattctataatattttagtgtgccataataatatcacctagtctatcagg	300	
MW847012: MN1-2-4	201	ATGgtaagcttataatcggtcctatcaatgtcacgcctgatgaaaatagtgattctataatattttagtgtgccataataatatcacctagtctatcagg	300	
MW847013: MN2-1-1	201	ATGgtaagcttataatcggtcctatcaatgtcacgcctgatgaaaatagtgattctataatattttagtgtgccataataatatcacctagtctatcagg	300	
MW847014: MN2-1-2	201	ATGgtaagcttataatcggtcctatcaatgtcacgcctgatgaaaatagtgattctataatattttagtgtgccataataatatcacctagtctatcagg	300	
MW847015: MN2-1-3	201	ATGgtaagcttataatcggtcctatcaatgtcacgcctgatgaaaatagtgattctataatattttagtgtgccataataatatcacctagtctatcagg	300	
MW847016: MN2-2-1	201	ATGgtaagcttataatcggtcctatcaatgtcacgcctgatgaaaatagtgattctataatattttagtgtgccataataatatcacctagtctatcagg	300	
MW847017: MN2-2-2	201	ATGgtaagcttataatcggtcctatcaatgtcacgcctgatgaaaatagtgattctataatattttagtgtgccataataatatcacctagtctatcagg	300	
MW847018: MN2-2-3	201	ATGgtaagcttataatcggtcctatcaatgtcacgcctgatgaaaatagtgattctataatattttagtgtgccataataatatcacctagtctatcagg	300	
MW847019: MN2-2-4	201	ATGgtaagcttataatcggtcctatcaatgtcacgcctgatgaaaatagtgattctataatattttagtgtgccataataatatcacctagtctatcagg	300	
MW847020: 9R1A-1	201	ATGgtaagcttataatcggtcctatcaatgtcacgcctgatgaaaatagtgattctataatattttagtgtgccataataatatcacctagtctatcagg	300	
MW847021: 9R2A-1	201	ATGgtaagcttataatcggtcctatcaatgtcacgcctgatgaaaatagtgattctataatattttagtgtgccataataatatcacctagtctatcagg	300	
MW847022: 9R3A-1	201	ATGgtaagcttataatcggtcctatcaatgtcacgcctgatgaaaatagtgattctataatattttagtgtgccataataatatcacctagtctatcagg	300	
MW847023: 9R3A-2	201	ATGgtaagcttataatcggtcctatcaatgtcacgcctgatgaaaatagtgattctataatattttagtgtgccataataatatcacctagtctatcagg	300	
MW847024: 9R1D-1	201	ATGgtaagcttataatcggtcctatcaatgtcacgcctgatgaaaatagtgattctataatattttagtgtgccataataatatcacctagtctatcagg	300	
MW847025: 9R1D-2	201	ATGgtaagcttataatcggtcctatcaatgtcacgcctgatgaaaatagtgattctataatattttagtgtgccataataatatcacctagtctatcagg	300	
MW847026: 9R1D-3	201	ATGgtaagcttataatcggtcctatcaatgtcacgcctgatgaaaatagtgattctataatattttagtgtgccataataatatcacctagtctatcagg	300	
MW847027: 9R1D-5	201	ATGgtaagcttataatcggtcctatcaatgtcacgcctgatgaaaatagtgattctataatattttagtgtgccataataatatcacctagtctatcagg	300	
MW847028: 9R2D-1	201	ATGgtaagcttataatcggtcctatcaatgtcacgcctgatgaaaatagtgattctataatattttagtgtgccataataatatcacctagtctatcagg	300	
MW847029: 9R2D-2	201	ATGgtaagcttataatcggtcctatcaatgtcacgcctgatgaaaatagtgattctataatattttagtgtgccataataatatcacctagtctatcagg	300	
MW847030: 9R2D-3	201	ATGgtaagcttataatcggtcctatcaatgtcacgcctgatgaaaatagtgattctataatattttagtgtgccataataatatcacctagtctatcagg	300	
MW847031: 9R2D-4	201	ATGgtaagcttataatcggtcctatcaatgtcacgcctgatgaaaatagtgattctataatattttagtgtgccataataatatcacctagtctatcagg	300	
MW847032: 9R2D-5	201	ATGgtaagcttataatcggtcctatcaatgtcacgcctgatgaaaatagtgattctataatattttagtgtgccataataatatcacctagtctatcagg	300	
MW847033: 9R2D-6	201	ATGgtaagcttataatcggtcctatcaatgtcacgcctgatgaaaatagtgattctataatattttagtgtgccataataatatcacctagtctatcagg	300	
MW847034: 9R2D-7	201	ATGgtaagcttataatcggtcctatcaatgtcacgcctgatgaaaatagtgattctataatattttagtgtgccataataatatcacctagtctatcagg	300	
MW847035: 9R2D-8	201	ATGgtaagcttataatcggtcctatcaatgtcacgcctgatgaaaatagtgattctataatattttagtgtgccataataatatcacctagtctatcagg	300	
MW847036: 7R3D-1	201	ATGgtaagcttataatcggtcctatcaatgtcacgcctgatgaaaatagtgattctataatattttagtgtgccataataatatcacctagtctatcagg	300	
MW847037: 7R3D-2	201	ATGgtaagcttataatcggtcctatcaatgtcacgcctgatgaaaatagtgattctataatattttagtgtgccataataatatcacctagtctatcagg	300	
MW847038: 7R3D-3	201	ATGgtaagcttataatcggtcctatcaatgtcacgcctgatgaaaatagtgattctataatattttagtgtgccataataatatcacctagtctatcagg	300	
MW847039: 7R3D-4	201	ATGgtaagcttataatcggtcctatcaatgtcacgcctgatgaaaatagtgattctataatattttagtgtgccataataatatcacctagtctatcagg	300	
MW847040: 7R3D-5	201	ATGgtaagcttataatcggtcctatcaatgtcacgcctgatgaaaatagtgattctataatattttagtgtgccataataatatcacctagtctatcagg	300	
MW847041: 7R3D-6	201	ATGgtaagcttataatcggtcctatcaatgtcacgcctgatgaaaatagtgattctataatattttagtgtgccataataatatcacctagtctatcagg	300	
MW847042: 7R3D-7	201	ATGgtaagcttataatcggtcctatcaatgtcacgcctgatgaaaatagtgattctataatattttagtgtgccataataatatcacctagtctatcagg	300	
MW847043: 7R3D-8	201	ATGgtaagcttataatcggtcctatcaatgtcacgcctgatgaaaatagtgattctataatattttagtgtgccataataatatcacctagtctatcagg	300	
MW847044: 7R3A-1	201	ATGgtaagcttataatcggtcctatcaatgtcacgcctgatgaaaatagtgattctataatattttagtgtgccataataatatcacctagtctatcagg	300	
MW847045: 7R3A-2	201	ATGgtaagcttataatcggtcctatcaatgtcacgcctgatgaaaatagtgattctataatattttagtgtgccataataatatcacctagtctatcagg	300	
MW847046: 7R3A-3	201	ATGgtaagcttataatcggtcctatcaatgtcacgcctgatgaaaatagtgattctataatattttagtgtgccataataatatcacctagtctatcagg	300	
MW847047: 7R3A-4	201	ATGgtaagcttataatcggtcctatcaatgtcacgcctgatgaaaatagtgattctataatattttagtgtgccataataatatcacctagtctatcagg	300	
MW847048: 7R3A-5	201	ATGgtaagcttataatcggtcctatcaatgtcacgcctgatgaaaatagtgattctataatattttagtgtgccataataatatcacctagtctatcagg	300	
MW847049: 7R3A-6	201	ATGgtaagcttataatcggtcctatcaatgtcacgcctgatgaaaatagtgattctataatattttagtgtgccataataatatcacctagtctatcagg	300	
MW847050: 7R3A-7	201	ATGgtaagcttataatcggtcctatcaatgtcacgcctgatgaaaatagtgattctataatattttagtgtgccataataatatcacctagtctatcagg	300	
MW847051: 7R2A-1	201	ATGgtaagcttataatcggtcctatcaatgtcacgcctgatgaaaatagtgattctataatattttagtgtgccataataatatcacctagtctatcagg	300	

```
                                         --------- intron -----------| L  S  T  S  S  G  W  D  A  V  L  D  G  O  T  M  E  D  D  C  D  L  P  N
AgVGSC2_cDNA                    2086     *****************************CTTTCTACGTCATCCGGTTGGGACGCCGTACTGGACGGTATAACGAACGAGGACGATTGTGATAAGCCAAA   2156
MW846959: ISU-B1_1               301     ttgctcatcagttaaattaattctttcagCTTTCTACGTCATCCGGTTGGGACGCCGTACTGGACGGTATAACGAACGAGGACGATTGTGATAAGCCAAA   400
MW846960: ISU-B1_5               301     ttgctcatcagttaaattaattctttcagCTTTCTACGTCATCCGGTTGGGACGCCGTACTGGACGGTATAACGAACGAGGACGATTGTGATAAGCCAAA   400
MW846961: ISU-B1_6               301     ttgctcatcagttaaattaattctttcagCTTTCTACGTCATCCGGTTGGGACGCCGTACTGGACGGTATAACGAACGAGGACGATTGTGATAAGCCAAA   400
MW846962: UIL-B1_2               301     ttgctcatcagttaaattaattctttcagCTTTCTACGTCATCCGGTTGGGACGCCGTACTGGACGGTATAACGAACGAGGACGATTGTGATAAGCCAAA   400
MW846963: UIL-B1_5               301     ttgctcatcagttaaattaattctttcagCTTTCTACGTCATCCGGTTGGGACGCCGTACTGGACGGTATAACGAACGAGGACGATTGTGATAAGCCAAA   400
MW846964: UIL-B1_10              301     ttgctcatcagttaaattaattctttcagCTTTCTACGTCATCCGGTTGGGACGCCGTACTGGACGGTATAACGAACGAGGACGATTGTGATAAGCCAAA   400
MW846965: UIL-B1_19              301     ttgctcatcagttaaattaattctttcagCTTTCTACGTCATCCGGTTGGGACGCCGTACTGGACGGTATAACGAACGAGGACGATTGTGATAAGCCAAA   400
MW846974: ISU-B3_4               301     ttgctcatcagttaaattaattctttcagCTTTCTACGTCATCCGGTTGGGACGCCGTACTGGACGGTATAACGAACGAGGACGATTGTGATAAGCCAAA   400
MW846975: ISU-B3_6               301     ttgctcatcagttaaattaattctttcagCTTTCTACGTCATCCGGTTGGGACGCCGTACTGGACGGTATAACGAACGAGGACGATTGTGATAAGCCAAA   400
MW846976: ISU-B3_8               301     ttgctcatcagttaaattaattctttcagCTTTCTACGTCATCCGGTTGGGACGCCGTACTGGACGGTATAACGAACGAGGACGATTGTGATAAGCCAAA   400
MW846977: ISU-B3_14              301     ttgctcatcagttaaattaattctttcagCTTTCTACGTCATCCGGTTGGGACGCCGTACTGGACGGTATAACGAACGAGGACGATTGTGATAAGCCAAA   400
MW846982: ISU-B4_5               301     ttgctcatcagttaaattaattctttcagCTTTCTACGTCATCCGGTTGGGACGCCGTACTAGACGGTATAACGAACGAGGACGATTGTGATAAGCCAAA   400
MW846983: ISU-B4_8               301     ttgctcatcagttaaattaattctttcagCTTTCTACGTCATCCGGTTGGGACGCCGTACTAGACGGTATAACGAACGAGGACGATTGTGATAAGCCAAA   400
MW846984: ISU-B4_13              301     ttgctcatcagttaaattaattctttcagCTTTCTACGTCATCCGGTTGGGACGCCGTACTAGACGGTATAACGAACGAGGACGATTGTGATAAGCCAAA   400
MW846985: ISU-B4_16              301     ttgctcatcagttaaattaattctttcagCTTTCTACGTCATCCGGTTGGGACGCCGTACTAGACGGTATAACGAACGAGGACGATTGTGATAAGCCAAA   400
MW846990: Suth-1                 301     ttgctcatcagttaaattaattctttcagCTTTCTACGTCATCCGGTTGGGACGCCGTACTGGACGGTATAACGAACGAGGACGATTGTGATAAGCCAAA   400
MW846991: Suth-2                 301     ttgctcatcagttaaattaattctttcagCTTTCTACGTCATCCGGTTGGGACGCCGTACTGGACGGTATAACGAACGAGGACGATTGTGATAAGCCAAA   400
MW846992: Suth-3                 301     ttgctcatcagttaaattaattctttcagCTTTCTACGTCATCCGGTTGGGACGCCGTACTGGACGGTATAACGAACGAGGACGATTGTGATAAGCCAAA   400
MW846993: Suth-4                 301     ttgctcatcagttaaattaattctttcagCTTTCTACGTCATCCGGTTGGGACGCCGTACTGGACGGTATAACGAACGAGGACGATTGTGATAAGCCAAA   400
MW846994: Suth-5                 301     ttgctcatcagttaaattaattctttcagCTTTCTACGTCATCCGGTTGGGACGCCGTACTGGACGGTATAACGAACGAGGACGATTGTGATAAGCCAAA   400
MW846995: Suth-6                 301     ttgctcatcagttaaattaattctttcagCTTTCTACGTCATCCGGTTGGGACGCCGTACTGGACGGTATAACGAACGAGGACGATTGTGATAAGCCAAA   400
MW846996: Suth-7                 301     ttgctcatcagttaaattaattctttcagCTTTCTACGTCATCCGGTTGGGACGCCGTACTGGACGGTATAACGAACGAGGACGATTGTGATAAGCCAAA   400
MW846997: Suth-8                 301     ttgctcatcagttaaattaattctttcagCTTTCTACGTCATCCGGTTGGGACGCCGTACTGGACGGTATAACGAACGAGGACGATTGTGATAAGCCAAA   400
MW846998: Nashua-1               301     ttgctcatcagttaaattaattctttcagCTTTCTACGTCATCCGGTTGGGACGCCGTACTGGACGGTATAACGAACGAGGACGATTGTGATAAGCCAAA   400
MW846999: Nashua-2               301     ttgctcatcagttaaattaattctttcagCTTTCTACGTCATCCGGTTGGGACGCCGTACTGGACGGTATAACGAACGAGGACGATTGTGATAAGCCAAA   400
MW847000: Nashua-3               301     ttgctcatcagttaaattaattctttcagCTTTCTACGTCATCCGGTTGGGACGCCGTACTGGACGGTATAACGAACGAGGACGATTGTGATAAGCCAAA   400
MW847001: Nashua-4               301     ttgctcatcagttaaattaattctttcagCTTTCTACGTCATCCGGTTGGGACGCCGTACTGGACGGTATAACGAACGAGGACGATTGTGATAAGCCAAA   400
MW847002: Nashua-5               301     ttgctcatcagttaaattaattctttcagCTTTCTACGTCATCCGGTTGGGACGCCGTACTGGACGGTATAACGAACGAGGACGATTGTGATAAGCCAAA   400
MW847003: Nashua-6               301     ttgctcatcagttaaattaattctttcagCTTTCTACGTCATCCGGTTGGGACGCCGTACTGGACGGTATAACGAACGAGGACGATTGTGATAAGCCAAA   400
MW847004: Nashua-7               301     ttgctcatcagttaaattaattctttcagCTTTCTACGTCATCCGGTTGGGACGCCGTACTGGACGGTATAACGAACGAGGACGATTGTGATAAGCCAAA   400
MW847005: MN1-1-1                301     ttgctcatcagttaaattaattctttcagCTTTCTACGTCATCCGGTTGGGACGCCGTACTGGACGGTATAACGAACGAGGACGATTGTGATAAGCCAAA   400
MW847006: MN1-1-2                301     ttgctcatcagttaaattaattctttcagCTTTCTACGTCATCCGGTTGGGACGCCGTACTGGACGGTATAACGAACGAGGACGATTGTGATAAGCCAAA   400
MW847007: MN1-1-3                301     ttgctcatcagttaaattaattctttcagCTTTCTACGTCATCCGGTTGGGACGCCGTACTGGACGGTATAACGAACGAGGACGATTGTGATAAGCCAAA   400
MW847008: MN1-1-4                301     ttgctcatcagttaaattaattctttcagCTTTCTACGTCATCCGGTTGGGACGCCGTACTGGACGGTATAACGAACGAGGACGATTGTGATAAGCCAAA   400
MW847009: MN1-2-1                301     ttgctcatcagttaaattaattctttcagCTTTCTACGTCATCCGGTTGGGACGCCGTACTGGACGGTATAACGAACGAGGACGATTGTGATAAGCCAAA   400
MW847010: MN1-2-2                301     ttgctcatcagttaaattaattctttcagCTTTCTACGTCATCCGGTTGGGACGCCGTACTGGACGGTATAACGAACGAGGACGATTGTGATAAGCCAAA   400
MW847011: MN1-2-3                301     ttgctcatcagttaaattaattctttcagCTTTCTACGTCATCCGGTTGGGACGCCGTACTGGACGGTATAACGAACGAGGACGATTGTGATAAGCCAAA   400
MW847012: MN1-2-4                301     ttgctcatcagttaaattaattctttcagCTTTCTACGTCATCCGGTTGGGACGCCGTACTGGACGGTATAACGAACGAGGACGATTGTGATAAGCCAAA   400
MW847013: MN2-1-1                301     ttgctcatcagttaaattaattctttcagCTTTCTACGTCATCCGGTTGGGACGCCGTACTGGACGGTATAACGAACGAGGACGATTGTGATAAGCCAAA   400
MW847014: MN2-1-2                301     ttgctcatcagttaaattaattctttcagCTTTCTACGTCATCCGGTTGGGACGCCGTACTGGACGGTATAACGAACGAGGACGATTGTGATAAGCCAAA   400
MW847015: MN2-1-3                301     ttgctcatcagttaaattaattctttcagCTTTCTACGTCATCCGGTTGGGACGCCGTACTGGACGGTATAACGAACGAGGACGATTGTGATAAGCCAAA   400
MW847016: MN2-2-1                301     ttgctcatcagttaaattaattctttcagCTTTCTACGTCATCCGGTTGGGACGCCGTACTGGACGGTATAACGAACGAGGACGATTGTGATAAGCCAAA   400
MW847017: MN2-2-2                301     ttgctcatcagttaaattaattctttcagCTTTCTACGTCATCCGGTTGGGACGCCGTACTGGACGGTATAACGAACGAGGACGATTGTGATAAGCCAAA   400
MW847018: MN2-2-3                301     ttgctcatcagttaaattaattctttcagCTTTCTACGTCATCCGGTTGGGACGCCGTACTGGACGGTATAACGAACGAGGACGATTGTGATAAGCCAAA   400
MW847019: MN2-2-4                301     ttgctcatcagttaaattaattctttcagCTTTCTACGTCATCCGGTTGGGACGCCGTACTGGACGGTATAACGAACGAGGACGATTGTGATAAGCCAAA   400
MW847020: 9R1A-1                 301     ttgctcatcagttaaattaattctttcagCTTTCTACGTCATCCGGTTGGGACGCCGTACTGGACGGTATAACGAACGAGGACGATTGTGATAAGCCAAA   400
MW847021: 9R2A-1                 301     ttgctcatcagttaaattaattctttcagCTTTCTACGTCATCCGGTTGGGACGCCGTACTGGACGGTATAACGAACGAGGACGATTGTGATAAGCCAAA   400
MW847022: 9R3A-1                 301     ttgctcatcagttaaattaattctttcagCTTTCTACGTCATCCGGTTGGGACGCCGTACTGGACGGTATAACGAACGAGGACGATTGTGATAAGCCAAA   400
MW847023: 9R3A-2                 301     ttgctcatcagttaaattaattctttcagCTTTCTACGTCATCCGGTTGGGACGCCGTACTGGACGGTATAACGAACGAGGACGATTGTGATAAGCCAAA   400
MW847024: 9R1D-1                 301     ttgctcatcagttaaattaattctttcagCTTTCTACGTCATCCGGTTGGGACGCCGTACTGGACGGTATAACGAACGAGGACGATTGTGATAAGCCAAA   400
MW847025: 9R1D-2                 301     ttgctcatcagttaaattaattctttcagCTTTCTACGTCATCCGGTTGGGACGCCGTACTGGACGGTATAACGAACGAGGACGATTGTGATAAGCCAAA   400
MW847026: 9R1D-3                 301     ttgctcatcagttaaattaattctttcagCTTTCTACGTCATCCGGTTGGGACGCCGTACTGGACGGTATAACGAACGAGGACGATTGTGATAAGCCAAA   400
MW847027: 9R1D-5                 301     ttgctcatcagttaaattaattctttcagCTTTCTACGTCATCCGGTTGGGACGCCGTACTGGACGGTATAACGAACGAGGACGATTGTGATAAGCCAAA   400
MW847028: 9R2D-1                 301     ttgctcatcagttaaattaattctttcagCTTTCTACGTCATCCGGTTGGGACGCCGTACTGGACGGTATAACGAACGAGGACGATTGTGATAAGCCAAA   400
MW847029: 9R2D-2                 301     ttgctcatcagttaaattaattctttcagCTTTCTACGTCATCCGGTTGGGACGCCGTACTGGACGGTATAACGAACGAGGACGATTGTGATAAGCCAAA   400
MW847030: 9R2D-3                 301     ttgctcatcagttaaattaattctttcagCTTTCTACGTCATCCGGTTGGGACGCCGTACTGGACGGTATAACGAACGAGGACGATTGTGATAAGCCAAA   400
MW847031: 9R2D-4                 301     ttgctcatcagttaaattaattctttcagCTTTCTACGTCATCCGGTTGGGACGCCGTACTGGACGGTATAACGAACGAGGACGATTGTGATAAGCCAAA   400
MW847032: 9R2D-5                 301     ttgctcatcagttaaattaattctttcagCTTTCTACGTCATCCGGTTGGGACGCCGTACTGGACGGTATAACGAACGAGGACGATTGTGATAAGCCAAA   400
MW847033: 9R2D-6                 301     ttgctcatcagttaaattaattctttcagCTTTCTACGTCATCCGGTTGGGACGCCGTACTGGACGGTATAACGAACGAGGACGATTGTGATAAGCCAAA   400
MW847034: 9R2D-7                 301     ttgctcatcagttaaattaattctttcagCTTTCTACGTCATCCGGTTGGGACGCCGTACTGGACGGTATAACGAACGAGGACGATTGTGATAAGCCAAA   400
MW847035: 9R2D-8                 301     ttgctcatcagttaaattaattctttcagCTTTCTACGTCATCCGGTTGGGACGCCGTACTGGACGGTATAACGAACGAGGACGATTGTGATAAGCCAAA   400
MW847036: 7R3D-1                 301     ttgctcatcagttaaattaattctttcagCTTTCTACGTCATCCGGTTGGGACGCCGTACTGGACGGTATAACGAACGAGGACGATTGTGATAAGCCAAA   400
MW847037: 7R3D-2                 301     ttgctcatcagttaaattaattctttcagCTTTCTACGTCATCCGGTTGGGACGCCGTACTGGACGGTATAACGAACGAGGACGATTGTGATAAGCCAAA   400
MW847038: 7R3D-3                 301     ttgctcatcagttaaattaattctttcagCTTTCTACGTCATCCGGTTGGGACGCCGTACTGGACGGTATAACGAACGAGGACGATTGTGATAAGCCAAA   400
MW847039: 7R3D-4                 301     ttgctcatcagttaaattaattctttcagCTTTCTACGTCATCCGGTTGGGACGCCGTACTGGACGGTATAACGAACGAGGACGATTGTGATAAGCCAAA   400
MW847040: 7R3D-5                 301     ttgctcatcagttaaattaattctttcagCTTTCTACGTCATCCGGTTGGGACGCCGTACTGGACGGTATAACGAACGAGGACGATTGTGATAAGCCAAA   400
MW847041: 7R3D-6                 301     ttgctcatcagttaaattaattctttcagCTTTCTACGTCATCCGGTTGGGACGCCGTACTGGACGGTATAACGAACGAGGACGATTGTGATAAGCCAAA   400
MW847042: 7R3D-7                 301     ttgctcatcagttaaattaattctttcagCTTTCTACGTCATCCGGTTGGGACGCCGTACTGGACGGTATAACGAACGAGGACGATTGTGATAAGCCAAA   400
MW847043: 7R3D-8                 301     ttgctcatcagttaaattaattctttcagCTTTCTACGTCATCCGGTTGGGACGCCGTACTGGACGGTATAACGAACGAGGACGATTGTGATAAGCCAAA   400
MW847044: 7R3A-1                 301     ttgctcatcagttaaattaattctttcagCTTTCTACGTCATCCGGTTGGGACGCCGTACTGGACGGTATAACGAACGAGGACGATTGTGATAAGCCAAA   400
MW847045: 7R3A-2                 301     ttgctcatcagttaaattaattctttcagCTTTCTACGTCATCCGGTTGGGACGCCGTACTGGACGGTATAACGAACGAGGACGATTGTGATAAGCCAAA   400
MW847046: 7R3A-3                 301     ttgctcatcagttaaattaattctttcagCTTTCTACGTCATCCGGTTGGGACGCCGTACTGGACGGTATAACGAACGAGGACGATTGTGATAAGCCAAA   400
MW847047: 7R3A-4                 301     ttgctcatcagttaaattaattctttcagCTTTCTACGTCATCCGGTTGGGACGCCGTACTGGACGGTATAACGAACGAGGACGATTGTGATAAGCCAAA   400
MW847048: 7R3A-5                 301     ttgctcatcagttaaattaattctttcagCTTTCTACGTCATCCGGTTGGGACGCCGTACTGGACGGTATAACGAACGAGGACGATTGTGATAAGCCAAA   400
MW847049: 7R3A-6                 301     ttgctcatcagttaaattaattctttcagCTTTCTACGTCATCCGGTTGGGACGCCGTACTGGACGGTATAACGAACGAGGACGATTGTGATAAGCCAAA   400
MW847050: 7R3A-7                 301     ttgctcatcagttaaattaattctttcagCTTTCTACGTCATCCGGTTGGGACGCCGTACTGGACGGTATAACGAACGAGGACGATTGTGATAAGCCAAA   400
MW847051: 7R2A-1                 301     ttgctcatcagttaaattaattctttcagCTTTCTACGTCATCCGGTTGGGACGCCGTACTGGACGGTATAACGAACGAGGACGATTGTGATAAGCCAAA   400
```

Susceptible (SUS) laboratory colonies

Resistant (RES) field collections

```
                                                                                                                  M1823I
                                                                      ..............................DIV S6......................
                                    L  E  M  G  I  T  G  S  C  G  S  S  A  V  G  T  A  F  L  L  S  Y  L  V  I  N  F  L  I  V  I  N [M]
AgVGSC2_cDNA            2157  TTTAGAAATGGGTATCACGGGAAGTTGTGGAAGTAGTGCCGTGGGCACGGCATTTTTACTCTCGTACCTGGTGATCAATTTCCTCATCGTCATAAAC[ATG]  2256
MW846959: ISU-B1_1       401  TTTAGAAATGGGTATCACGGGAAGTTGTGGAAGTAGTGCCGTGGGCACGGCATTTTTACTCTCGTACCTGGTGATCAATTTCCTCATCGTCATAAAC[ATG]  500
MW846960: ISU-B1_5       401  TTTAGAAATGGGTATCACGGGAAGTTGTGGAAGTAGTGCCGTGGGCACGGCATTTTTACTCTCGTACCTGGTGATCAATTTCCTCATCGTCATAAAC[ATG]  500
MW846961: ISU-B1_6       401  TTTAGAAATGGGTATCACGGGAAGTTGTGGAAGTAGTGCCGTGGGCACGGCATTTTTACTCTCGTACCTGGTGATCAATTTCCTCATCGTCATAAAC[ATG]  500
MW846962: UIL-B1_2       401  TTTAGAAATGGGTATCACGGGAAGTTGTGGAAGTAGTGCCGTGGGCACGGCATTTTTACTCTCGTACCTGGTGATCAATTTCCTCATCGTCATAAAC[ATG]  500
MW846963: UIL-B1_5       401  TTTAGAAATGGGTATCACGGGAAGTTGTGGAAGTAGTGCCGTGGGCACGGCATTTTTACTCTCGTACCTGGTGATCAATTTCCTCATCGTCATAAAC[ATG]  500
MW846964: UIL-B1_10      401  TTTAGAAATGGGTATCACGGGAAGTTGTGGAAGTAGTGCCGTGGGCACGGCATTTTTACTCTCGTACCTGGTGATCAATTTCCTCATCGTCATAAAC[ATG]  500
MW846965: UIL-B1_19      401  TTTAGAAATGGGTATCACGGGAAGTTGTGGAAGTAGTGCCGTGGGCACGGCATTTTTACTCTCGTACCTGGTGATCAATTTCCTCATCGTCATAAAC[ATG]  500
MW846974: ISU-B3_4       401  TTTAGAAATGGGTATCACGGGAAGTTGTGGAAGTAGTGCCGTGGGCACGGCATTTTTACTCTCGTACCTGGTGATCAATTTCCTCATCGTCATAAAC[ATG]  500
MW846975: ISU-B3_6       401  TTTAGAAATGGGTATCACGGGAAGTTGTGGAAGTAGTGCCGTGGGCACGGCATTTTTACTCTCGTACCTGGTGATCAATTTCCTCATCGTCATAAAC[ATG]  500
MW846976: ISU-B3_8       401  TTTAGAAATGGGTATCACGGGAAGTTGTGGAAGTAGTGCCGTGGGCACGGCATTTTTACTCTCGTACCTGGTGATCAATTTCCTCATCGTCATAAAC[ATG]  500
MW846977: ISU-B3_14      401  TTTAGAAATGGGTATCACGGGAAGTTGTGGAAGTAGTGCCGTGGGCACGGCATTTTTACTCTCGTACCTGGTGATCAATTTCCTCATCGTCATAAAC[ATG]  500
MW846982: ISU-B4_5       401  TTTAGAAATGGGTATCACGGGAAGTTGTGGAAGTAGTGCCGTGGGCACGGCATTTTTACTCTCGTACCTGGTGATCAATTTCCTCATCGTCATAAAC[ATG]  500
MW846983: ISU-B4_8       401  TTTAGAAATGGGTATCACGGGAAGTTGTGGAAGTAGTGCCGTGGGCACGGCATTTTTACTCTCGTACCTGGTGATCAATTTCCTCATCGTCATAAAC[ATG]  500
MW846984: ISU-B4_13      401  TTTAGAAATGGGTATCACGGGAAGTTGTGGAAGTAGTGCCGTGGGCACGGCATTTTTACTCTCGTACCTGGTGATCAATTTCCTCATCGTCATAAAC[ATG]  500
MW846985: ISU-B4_16      401  TTTAGAAATGGGTATCACGGGAAGTTGTGGAAGTAGTGCCGTGGGCACGGCATTTTTACTCTCGTACCTGGTGATCAATTTCCTCATCGTCATAAAC[ATG]  500
MW846990: Suth-1         401  TTTAGAAATGGGTATCACGGGAAGTTGTGGAAGTAGTGCCGTGGGCACGGCATTTTTACTCTCGTACCTGGTGATCAATTTCCTCATCGTCATAAAC[ATG]  500
MW846991: Suth-2         401  TTTAGAAATGGGTATCACGGGAAGTTGTGGAAGTAGTGCCGTGGGCACGGCATTTTTACTCTCGTACCTGGTGATCAATTTCCTCATCGTCATAAAC[ATG]  500
MW846992: Suth-3         401  TTTAGAAATGGGTATCACGGGAAGTTGTGGAAGTAGTGCCGTGGGCACGGCATTTTTACTCTCGTACCTGGTGATCAATTTCCTCATCGTCATAAAC[ATG]  500
MW846993: Suth-4         401  TTTAGAAATGGGTATCACGGGAAGTTGTGGAAGTAGTGCCGTGGGCACGGCATTTTTACTCTCGTACCTGGTGATCAATTTCCTCATCGTCATAAAC[ATG]  500
MW846994: Suth-5         401  TTTAGAAATGGGTATCACGGGAAGTTGTGGAAGTAGTGCCGTGGGCACGGCATTTTTACTCTCGTACCTGGTGATCAATTTCCTCATCGTCATAAAC[ATG]  500
MW846995: Suth-6         401  TTTAGAAATGGGTATCACGGGAAGTTGTGGAAGTAGTGCCGTGGGCACGGCATTTTTACTCTCGTACCTGGTGATCAATTTCCTCATCGTCATAAAC[ATG]  500
MW846996: Suth-7         401  TTTAGAAATGGGTATCACGGGAAGTTGTGGAAGTAGTGCCGTGGGCACGGCATTTTTACTCTCGTACCTGGTGATCAATTTCCTCATCGTCATAAAC[ATG]  500
MW846997: Suth-8         401  TTTAGAAATGGGTATCACGGGAAGTTGTGGAAGTAGTGCCGTGGGCACGGCATTTTTACTCTCGTACCTGGTGATCAATTTCCTCATCGTCATAAAC[ATG]  500
MW846998: Nashua-1       401  TTTAGAAATGGGTATCACGGGAAGTTGTGGAAGTAGTGCCGTGGGCACGGCATTTTTACTCTCGTACCTGGTGATCAATTTCCTCATCGTCATAAAC[ATG]  500
MW846999: Nashua-2       401  TTTAGAAATGGGTATCACGGGAAGTTGTGGAAGTAGTGCCGTGGGCACGGCATTTTTACTCTCGTACCTGGTGATCAATTTCCTCATCGTCATAAAC[ATG]  500
MW847000: Nashua-3       401  TTTAGAAATGGGTATCACGGGAAGTTGTGGAAGTAGTGCCGTGGGCACGGCATTTTTACTCTCGTACCTGGTGATCAATTTCCTCATCGTCATAAAC[ATG]  500
MW847001: Nashua-4       401  TTTAGAAATGGGTATCACGGGAAGTTGTGGAAGTAGTGCCGTGGGCACGGCATTTTTACTCTCGTACCTGGTGATCAATTTCCTCATCGTCATAAAC[ATG]  500
MW847002: Nashua-5       401  TTTAGAAATGGGTATCACGGGAAGTTGTGGAAGTAGTGCCGTGGGCACGGCATTTTTACTCTCGTACCTGGTGATCAATTTCCTCATCGTCATAAAC[ATG]  500
MW847003: Nashua-6       401  TTTAGAAATGGGTATCACGGGAAGTTGTGGAAGTAGTGCCGTGGGCACGGCATTTTTACTCTCGTACCTGGTGATCAATTTCCTCATCGTCATAAAC[ATG]  500
MW847004: Nashua-7       401  TTTAGAAATGGGTATCACGGGAAGTTGTGGAAGTAGTGCCGTGGGCACGGCATTTTTACTCTCGTACCTGGTGATCAATTTCCTCATCGTCATAAAC[ATG]  500
MW847005: MN1-1-1        401  TTTAGAAATGGGTATCACGGGAAGTTGTGGAAGTAGTGCCGTGGGCACGGCATTTTTACTCTCGTACCTGGTGATCAATTTCCTCATCGTCATAAAC[ATG]  500
MW847006: MN1-1-2        401  TTTAGAAATGGGTATCACGGGAAGTTGTGGAAGTAGTGCCGTGGGCACGGCATTTTTACTCTCGTACCTGGTGATCAATTTCCTCATCGTCATAAAC[ATG]  500
MW847007: MN1-1-3        401  TTTAGAAATGGGTATCACGGGAAGTTGTGGAAGTAGTGCCGTGGGCACGGCATTTTTACTCTCGTACCTGGTGATCAATTTCCTCATCGTCATAAAC[ATG]  500
MW847008: MN1-1-4        401  TTTAGAAATGGGTATCACGGGAAGTTGTGGAAGTAGTGCCGTGGGCACGGCATTTTTACTCTCGTACCTGGTGATCAATTTCCTCATCGTCATAAAC[ATG]  500
MW847009: MN1-2-1        401  TTTAGAAATGGGTATCACGGGAAGTTGTGGAAGTAGTGCCGTGGGCACGGCATTTTTACTCTCGTACCTGGTGATCAATTTCCTCATCGTCATAAAC[ATG]  500
MW847010: MN1-2-2        401  TTTAGAAATGGGTATCACGGGAAGTTGTGGAAGTAGTGCCGTGGGCACGGCATTTTTACTCTCGTACCTGGTGATCAATTTCCTCATCGTCATAAAC[ATG]  500
MW847011: MN1-2-3        401  TTTAGAAATGGGTATCACGGGAAGTTGTGGAAGTAGTGCCGTGGGCACGGCATTTTTACTCTCGTACCTGGTGATCAATTTCCTCATCGTCATAAAC[ATG]  500
MW847012: MN1-2-4        401  TTTAGAAATGGGTATCACGGGAAGTTGTGGAAGTAGTGCCGTGGGCACGGCATTTTTACTCTCGTACCTGGTGATCAATTTCCTCATCGTCATAAAC[ATG]  500
MW847013: MN2-1-1        401  TTTAGAAATGGGTATCACGGGAAGTTGTGGAAGTAGTGCCGTGGGCACGGCATTTTTACTCTCGTACCTGGTGATCAATTTCCTCATCGTCATAAAC[ATG]  500
MW847014: MN2-1-2        401  TTTAGAAATGGGTATCACGGGAAGTTGTGGAAGTAGTGCCGTGGGCACGGCATTTTTACTCTCGTACCTGGTGATCAATTTCCTCATCGTCATAAAC[ATG]  500
MW847015: MN2-1-3        401  TTTAGAAATGGGTATCACGGGAAGTTGTGGAAGTAGTGCCGTGGGCACGGCATTTTTACTCTCGTACCTGGTGATCAATTTCCTCATCGTCATAAAC[ATG]  500
MW847016: MN2-2-1        401  TTTAGAAATGGGTATCACGGGAAGTTGTGGAAGTAGTGCCGTGGGCACGGCATTTTTACTCTCGTACCTGGTGATCAATTTCCTCATCGTCATAAAC[ATG]  500
MW847017: MN2-2-2        401  TTTAGAAATGGGTATCACGGGAAGTTGTGGAAGTAGTGCCGTGGGCACGGCATTTTTACTCTCGTACCTGGTGATCAATTTCCTCATCGTCATAAAC[ATG]  500
MW847018: MN2-2-3        401  TTTAGAAATGGGTATCACGGGAAGTTGTGGAAGTAGTGCCGTGGGCACGGCATTTTTACTCTCGTACCTGGTGATCAATTTCCTCATCGTCATAAAC[ATG]  500
MW847019: MN2-2-4        401  TTTAGAAATGGGTATCACGGGAAGTTGTGGAAGTAGTGCCGTGGGCACGGCATTTTTACTCTCGTACCTGGTGATCAATTTCCTCATCGTCATAAAC[ATG]  500
MW847020: 9R1A-1         401  TTTAGAAATGGGTATCACGGGAAGTTGTGGAAGTAGTGCCGTGGGCACGGCATTTTTACTCTCGTACCTGGTGATCAATTTCCTCATCGTCATAAAC[ATG]  500
MW847021: 9R2A-1         401  TTTAGAAATGGGTATCACGGGAAGTTGTGGAAGTAGTGCCGTGGGCACGGCATTTTTACTCTCGTACCTGGTGATCAATTTCCTCATCGTCATAAAC[ATG]  500
MW847022: 9R3A-1         401  TTTAGAAATGGGTATCACGGGAAGTTGTGGAAGTAGTGCCGTGGGCACGGCATTTTTACTCTCGTACCTGGTGATCAATTTCCTCATCGTCATAAAC[ATG]  500
MW847023: 9R3A-2         401  TTTAGAAATGGGTATCACGGGAAGTTGTGGAAGTAGTGCCGTGGGCACGGCATTTTTACTCTCGTACCTGGTGATCAATTTCCTCATCGTCATAAAC[ATG]  500
MW847024: 9R1D-1         401  TTTAGAAATGGGTATCACGGGAAGTTGTGGAAGTAGTGCCGTGGGCACGGCATTTTTACTCTCGTACCTGGTGATCAATTTCCTCATCGTCATAAAC[ATG]  500
MW847025: 9R1D-2         401  TTTAGAAATGGGTATCACGGGAAGTTGTGGAAGTAGTGCCGTGGGCACGGCATTTTTACTCTCGTACCTGGTGATCAATTTCCTCATCGTCATAAAC[ATG]  500
MW847026: 9R1D-3         401  TTTAGAAATGGGTATCACGGGAAGTTGTGGAAGTAGTGCCGTGGGCACGGCATTTTTACTCTCGTACCTGGTGATCAATTTCCTCATCGTCATAAAC[ATG]  500
MW847027: 9R1D-5         401  TTTAGAAATGGGTATCACGGGAAGTTGTGGAAGTAGTGCCGTGGGCACGGCATTTTTACTCTCGTACCTGGTGATCAATTTCCTCATCGTCATAAAC[ATG]  500
MW847028: 9R2D-1         401  TTTAGAAATGGGTATCACGGGAAGTTGTGGAAGTAGTGCCGTGGGCACGGCATTTTTACTCTCGTACCTGGTGATCAATTTCCTCATCGTCATAAAC[ATG]  500
MW847029: 9R2D-2         401  TTTAGAAATGGGTATCACGGGAAGTTGTGGAAGTAGTGCCGTGGGCACGGCATTTTTACTCTCGTACCTGGTGATCAATTTCCTCATCGTCATAAAC[ATG]  500
MW847030: 9R2D-3         401  TTTAGAAATGGGTATCACGGGAAGTTGTGGAAGTAGTGCCGTGGGCACGGCATTTTTACTCTCGTACCTGGTGATCAATTTCCTCATCGTCATAAAC[ATG]  500
MW847031: 9R2D-4         401  TTTAGAAATGGGTATCACGGGAAGTTGTGGAAGTAGTGCCGTGGGCACGGCATTTTTACTCTCGTACCTGGTGATCAATTTCCTCATCGTCATAAAC[ATG]  500
MW847032: 9R2D-5         401  TTTAGAAATGGGTATCACGGGAAGTTGTGGAAGTAGTGCCGTGGGCACGGCATTTTTACTCTCGTACCTGGTGATCAATTTCCTCATCGTCATAAAC[ATG]  500
MW847033: 9R2D-6         401  TTTAGAAATGGGTATCACGGGAAGTTGTGGAAGTAGTGCCGTGGGCACGGCATTTTTACTCTCGTACCTGGTGATCAATTTCCTCATCGTCATAAAC[ATG]  500
MW847034: 9R2D-7         401  TTTAGAAATGGGTATCACGGGAAGTTGTGGAAGTAGTGCCGTGGGCACGGCATTTTTACTCTCGTACCTGGTGATCAATTTCCTCATCGTCATAAAC[ATG]  500
MW847035: 9R2D-8         401  TTTAGAAATGGGTATCACGGGAAGTTGTGGAAGTAGTGCCGTGGGCACGGCATTTTTACTCTCGTACCTGGTGATCAATTTCCTCATCGTCATAAAC[ATG]  500
MW847036: 7R3D-1         401  TTTAGAAATGGGTATCACGGGAAGTTGTGGAAGTAGTGCCGTGGGCACGGCATTTTTACTCTCGTACCTGGTGATCAATTTCCTCATCGTCATAAAC[ATG]  500
MW847037: 7R3D-2         401  TTTAGAAATGGGTATCACGGGAAGTTGTGGAAGTAGTGCCGTGGGCACGGCATTTTTACTCTCGTACCTGGTGATCAATTTCCTCATCGTCATAAAC[ATG]  500
MW847038: 7R3D-3         401  TTTAGAAATGGGTATCACGGGAAGTTGTGGAAGTAGTGCCGTGGGCACGGCATTTTTACTCTCGTACCTGGTGATCAATTTCCTCATCGTCATAAAC[ATG]  500
MW847039: 7R3D-4         401  TTTAGAAATGGGTATCACGGGAAGTTGTGGAAGTAGTGCCGTGGGCACGGCATTTTTACTCTCGTACCTGGTGATCAATTTCCTCATCGTCATAAAC[ATG]  500
MW847040: 7R3D-5         401  TTTAGAAATGGGTATCACGGGAAGTTGTGGAAGTAGTGCCGTGGGCACGGCATTTTTACTCTCGTACCTGGTGATCAATTTCCTCATCGTCATAAAC[ATG]  500
MW847041: 7R3D-6         401  TTTAGAAATGGGTATCACGGGAAGTTGTGGAAGTAGTGCCGTGGGCACGGCATTTTTACTCTCGTACCTGGTGATCAATTTCCTCATCGTCATAAAC[ATG]  500
MW847042: 7R3D-7         401  TTTAGAAATGGGTATCACGGGAAGTTGTGGAAGTAGTGCCGTGGGCACGGCATTTTTACTCTCGTACCTGGTGATCAATTTCCTCATCGTCATAAAC[ATG]  500
MW847043: 7R3D-8         401  TTTAGAAATGGGTATCACGGGAAGTTGTGGAAGTAGTGCCGTGGGCACGGCATTTTTACTCTCGTACCTGGTGATCAATTTCCTCATCGTCATAAAC[ATG]  500
MW847044: 7R3A-1         401  TTTAGAAATGGGTATCACGGGAAGTTGTGGAAGTAGTGCCGTGGGCACGGCATTTTTACTCTCGTACCTGGTGATCAATTTCCTCATCGTCATAAAC[ATG]  500
MW847045: 7R3A-2         401  TTTAGAAATGGGTATCACGGGAAGTTGTGGAAGTAGTGCCGTGGGCACGGCATTTTTACTCTCGTACCTGGTGATCAATTTCCTCATCGTCATAAAC[ATG]  500
MW847046: 7R3A-3         401  TTTAGAAATGGGTATCACGGGAAGTTGTGGAAGTAGTGCCGTGGGCACGGCATTTTTACTCTCGTACCTGGTGATCAATTTCCTCATCGTCATAAAC[ATG]  500
MW847047: 7R3A-4         401  TTTAGAAATGGGTATCACGGGAAGTTGTGGAAGTAGTGCCGTGGGCACGGCATTTTTACTCTCGTACCTGGTGATCAATTTCCTCATCGTCATAAAC[ATG]  500
MW847048: 7R3A-5         401  TTTAGAAATGGGTATCACGGGAAGTTGTGGAAGTAGTGCCGTGGGCACGGCATTTTTACTCTCGTACCTGGTGATCAATTTCCTCATCGTCATAAAC[ATG]  500
MW847049: 7R3A-6         401  TTTAGAAATGGGTATCACGGGAAGTTGTGGAAGTAGTGCCGTGGGCACGGCATTTTTACTCTCGTACCTGGTGATCAATTTCCTCATCGTCATAAAC[ATG]  500
MW847050: 7R3A-7         401  TTTAGAAATGGGTATCACGGGAAGTTGTGGAAGTAGTGCCGTGGGCACGGCATTTTTACTCTCGTACCTGGTGATCAATTTCCTCATCGTCATAAAC[ATG]  500
MW847051: 7R2A-1         401  TTTAGAAATGGGTATCACGGGAAGTTGTGGAAGTAGTGCCGTGGGCACGGCATTTTTACTCTCGTACCTGGTGATCAATTTCCTCATCGTCATAAAC[ATG]  500
```

Susceptible (SUS) laboratory colonies (MW846959 – MW846985)

Resistant (RES) field collections (MW846990 – MW847051)

		......DIV S6.....<br>Y I A V I L E N Y S Q A		
AgVGSC2_cDNA	2257	TACATCGCAGTCATTCTGGAGAACTATTCACAGGCT	2292	
MW846959: ISU-B1_1	501	TACATCGCAGTCATTCTGGAGAACTATTCACAGGCT	536	**Susceptible (SUS) laboratory colonies**
MW846960: ISU-B1_5	501	TACATCGCAGTCATTCTGGAGAACTATTCACAGGCT	536	
MW846961: ISU-B1_6	501	TACATCGCAGTCATTCTGGAGAACTATTCACAGGCT	536	
MW846962: UIL-B1_2	501	TACATCGCAGTCATTCTGGAGAACTATTCACAGGCT	536	
MW846963: UIL-B1_5	501	TACATCGCAGTCATTCTGGAGAACTATTCACAGGCT	536	
MW846964: UIL-B1_10	501	TACATCGCAGTCATTCTGGAGAACTATTCACAGGCT	536	
MW846965: UIL-B1_19	501	TACATCGCAGTCATTCTGGAGAACTATTCACAGGCT	536	
MW846974: ISU-B3_4	501	TACATCGCAGTCATTCTGGAGAACTATTCACAGGCT	536	
MW846975: ISU-B3_6	501	TACATCGCAGTCATTCTGGAGAACTATTCACAGGCT	536	
MW846976: ISU-B3_8	501	TACATCGCAGTCATTCTGGAGAACTATTCACAGGCT	536	
MW846977: ISU-B3_14	501	TACATCGCAGTCATTCTGGAGAACTATTCACAGGCT	536	
MW846982: ISU-B4_5	501	TACATCGCAGTCATTCTGGAGAACTATTCACAGGCT	536	
MW846983: ISU-B4_8	501	TACATCGCAGTCATTCTGGAGAACTATTCACAGGCT	536	
MW846984: ISU-B4_13	501	TACATCGCAGTCATTCTGGAGAACTATTCACAGGCT	536	
MW846985: ISU-B4_16	501	TACATCGCAGTCATTCTGGAGAACTATTCACAGGCT	536	
MW846990: Suth-1	501	TACATCGCAGTCATTCTGGAGAACTATTCACAGGCT	536	**Resistant (RES) field collections**
MW846991: Suth-2	501	TACATCGCAGTCATTCTGGAGAACTATTCACAGGCT	536	
MW846992: Suth-3	501	TACATCGCAGTCATTCTGGAGAACTATTCACAGGCT	536	
MW846993: Suth-4	501	TACATCGCAGTCATTCTGGAGAACTATTCACAGGCT	536	
MW846994: Suth-5	501	TACATCGCAGTCATTCTGGAGAACTATTCACAGGCT	536	
MW846995: Suth-6	501	TACATCGCAGTCATTCTGGAGAACTATTCACAGGCT	536	
MW846996: Suth-7	501	TACATCGCAGTCATTCTGGAGAACTATTCACAGGCT	536	
MW846997: Suth-8	501	TACATCGCAGTCATTCTGGAGAACTATTCACAGGCT	536	
MW846998: Nashua-1	501	TACATCGCAGTCATTCTGGAGAACTATTCACAGGCT	536	
MW846999: Nashua-2	501	TACATCGCAGTCATTCTGGAGAACTATTCACAGGCT	536	
MW847000: Nashua-3	501	TACATCGCAGTCATTCTGGAGAACTATTCACAGGCT	536	
MW847001: Nashua-4	501	TACATCGCAGTCATTCTGGAGAACTATTCACAGGCT	536	
MW847002: Nashua-5	501	TACATCGCAGTCATTCTGGAGAACTATTCACAGGCT	536	
MW847003: Nashua-6	501	TACATCGCAGTCATTCTGGAGAACTATTCACAGGCT	536	
MW847004: Nashua-7	501	TACATCGCAGTCATTCTGGAGAACTATTCACAGGCT	536	
MW847005: MN1-1-1	501	TACATCGCAGTCATTCTGGAGAACTATTCACAGGCT	536	
MW847006: MN1-1-2	501	TACATCGCAGTCATTCTGGAGAACTATTCACAGGCT	536	
MW847007: MN1-1-3	501	TACATCGCAGTCATTCTGGAGAACTATTCACAGGCT	536	
MW847008: MN1-1-4	501	TACATCGCAGTCATTCTGGAGAACTATTCACAGGCT	536	
MW847009: MN1-2-1	501	TACATCGCAGTCATTCTGGAGAACTATTCACAGGCT	536	
MW847010: MN1-2-2	501	TACATCGCAGTCATTCTGGAGAACTATTCACAGGCT	536	
MW847011: MN1-2-3	501	TACATCGCAGTCATTCTGGAGAACTATTCACAGGCT	536	
MW847012: MN1-2-4	501	TACATCGCAGTCATTCTGGAGAACTATTCACAGGCT	536	
MW847013: MN2-1-1	501	TACATCGCAGTCATTCTGGAGAACTATTCACAGGCT	536	
MW847014: MN2-1-2	501	TACATCGCAGTCATTCTGGAGAACTATTCACAGGCT	536	
MW847015: MN2-1-3	501	TACATCGCAGTCATTCTGGAGAACTATTCACAGGCT	536	
MW847016: MN2-2-1	501	TACATCGCAGTCATTCTGGAGAACTATTCACAGGCT	536	
MW847017: MN2-2-2	501	TACATCGCAGTCATTCTGGAGAACTATTCACAGGCT	536	
MW847018: MN2-2-3	501	TACATCGCAGTCATTCTGGAGAACTATTCACAGGCT	536	
MW847019: MN2-2-4	501	TACATCGCAGTCATTCTGGAGAACTATTCACAGGCT	536	
MW847020: 9R1A-1	501	TACATCGCAGTCATTCTGGAGAACTATTCACAGGCT	536	
MW847021: 9R2A-1	501	TACATCGCAGTCATTCTGGAGAACTATTCACAGGCT	536	
MW847022: 9R3A-1	501	TACATCGCAGTCATTCTGGAGAACTATTCACAGGCT	536	
MW847023: 9R3A-2	501	TACATCGCAGTCATTCTGGAGAACTATTCACAGGCT	536	
MW847024: 9R1D-1	501	TACATCGCAGTCATTCTGGAGAACTATTCACAGGCT	536	
MW847025: 9R1D-2	501	TACATCGCAGTCATTCTGGAGAACTATTCACAGGCT	536	
MW847026: 9R1D-3	501	TACATCGCAGTCATTCTGGAGAACTATTCACAGGCT	536	
MW847027: 9R1D-5	501	TACATCGCAGTCATTCTGGAGAACTATTCACAGGCT	536	
MW847028: 9R2D-1	501	TACATCGCAGTCATTCTGGAGAACTATTCACAGGCT	536	
MW847029: 9R2D-2	501	TACATCGCAGTCATTCTGGAGAACTATTCACAGGCT	536	
MW847030: 9R2D-3	501	TACATCGCAGTCATTCTGGAGAACTATTCACAGGCT	536	
MW847031: 9R2D-4	501	TACATCGCAGTCATTCTGGAGAACTATTCACAGGCT	536	
MW847032: 9R2D-5	501	TACATCGCAGTCATTCTGGAGAACTATTCACAGGCT	536	
MW847033: 9R2D-6	501	TACATCGCAGTCATTCTGGAGAACTATTCACAGGCT	536	
MW847034: 9R2D-7	501	TACATCGCAGTCATTCTGGAGAACTATTCACAGGCT	536	
MW847035: 9R2D-8	501	TACATCGCAGTCATTCTGGAGAACTATTCACAGGCT	536	
MW847036: 7R3D-1	501	TACATCGCAGTCATTCTGGAGAACTATTCACAGGCT	536	
MW847037: 7R3D-2	501	TACATCGCAGTCATTCTGGAGAACTATTCACAGGCT	536	
MW847038: 7R3D-3	501	TACATCGCAGTCATTCTGGAGAACTATTCACAGGCT	536	
MW847039: 7R3D-4	501	TACATCGCAGTCATTCTGGAGAACTATTCACAGGCT	536	
MW847040: 7R3D-5	501	TACATCGCAGTCATTCTGGAGAACTATTCACAGGCT	536	
MW847041: 7R3D-6	501	TACATCGCAGTCATTCTGGAGAACTATTCACAGGCT	536	
MW847042: 7R3D-7	501	TACATCGCAGTCATTCTGGAGAACTATTCACAGGCT	536	
MW847043: 7R3D-8	501	TACATCGCAGTCATTCTGGAGAACTATTCACAGGCT	536	
MW847044: 7R3A-1	501	TACATCGCAGTCATTCTGGAGAACTATTCACAGGCT	536	
MW847045: 7R3A-2	501	TACATCGCAGTCATTCTGGAGAACTATTCACAGGCT	536	
MW847046: 7R3A-3	501	TACATCGCAGTCATTCTGGAGAACTATTCACAGGCT	536	
MW847047: 7R3A-4	501	TACATCGCAGTCATTCTGGAGAACTATTCACAGGCT	536	
MW847048: 7R3A-5	501	TACATCGCAGTCATTCTGGAGAACTATTCACAGGCT	536	
MW847049: 7R3A-6	501	TACATCGCAGTCATTCTGGAGAACTATTCACAGGCT	536	
MW847050: 7R3A-7	501	TACATCGCAGTCATTCTGGAGAACTATTCACAGGCT	536	
MW847051: 7R2A-1	501	TACATCGCAGTCATTCTGGAGAACTATTCACAGGCT	536	

Table S1. Oligonucleotide primers used to amplify the *Aphis glycines* voltage-gated sodium channel (*vgsc*). **A)** Full-length cDNAs for *vgsc* subunit h1 (AG6007485.1) and h2 (AG6007488.1 and AG6007489.1). Primer sequence corresponding to ATG start and reverse-complement of TAG (CTA) or TAA (TTA) stop codons are indicated. **B)** putative variable regions of *vgsc* domain II (DII) segments 4-6 (S4-6), DIIIS6 to DIVS1, and DIVS4-S6, and **C)** regions with L1014F knockdown resistance (*kdr;* C to T transition at AG6007485-RA position 3070) and M918I (G to A transition at AG6007485-RA position 2784) mutations detected by a *Bst*EII PCR-RFLP assay (*Bst*EII recognition sequence 5'-GGTNACC-3') and ligase chain reaction (LCR) assays, respectively. LCR assays consisted of three separate oligonucleotide probes; alternate up-stream allele-specific wildtype susceptible P1-Met_G_*susc* and mutant P1-Ile_A_*res* probes (variant nucleotide positions at 3' terminal ends (**bold**). The M13F region at the 5' end of P1 probes is non-template sequence corresponding to the M13 forward primer and the SP6 region is filler sequence, incorporated for amplification of LCR products and introduction of size differences for allele discrimination in subsequent assay steps, respectively. Specifically, the P1-Ile_A_*res* allele-specific probe was 24 nt longer compared to the P1-Met_G_*susc* probe. The universal 5'-phosphorylated P2-Phos probe anneals downstream and immediately adjacent to P1 probes, and incorporated non-template the reverse complement of the M13 reverse primer sequence [M13Rrc] for subsequent reamplification using primers M13_5p17nt-F and M13_5p18nt-R. All non-genomic template sequence is in lowercase.

A) Full-length cDNA amplification

Primer name	Primer sequence
AG6007485-F	5′-ATG TCC ATT GCT GAC ACC GAT T-3′
AG6007485-R	5′-CTA GCA TTT TAA ACT ATC CAG GTC G-3′
AG6007489-F	5′-ATG AGT GTG TAT AGT AGT GAG GAA CTC C-3′
AG6007488-R	5′-TTA GAC ATC GGC GAG TCT TGA G-3′

B) Putative variable regions; genomic DNA amplicons

Primer name	Primer sequence 5'-3'
DIIS4-S6-F	5′-CCG TAT TGC GTT CAT TTC GTT-3′
DIIS4-S6-R	5′-CGT TCA AAT GCT TCT GTG ATC TT-3′
DIIIS6-DIVS1-F	5′-GGG ACG CAG TAG ATT CAA GAG AC-3′
DIIIS6-DIVS1-R	5′-GGT AAT GGT CAA GTG TCA TTG TTA AC-3′
DIVS4-S6-F	5′-GTG TCA CCA ACG CTC CTT CG-3′
DIVS4-S6-R	5′-CCT CTG TAG CCT GTG AAT AGT TCT C-3′

C) Genotyping assayed regions; genomic DNA amplicons

Primer name	Primer sequence 5'-3'		
AG*kdr*-F	5′-CGC CGT TAT GGG TAT GCA GT-3′	*Bst*EII PCR-RFLP	
AG*kdr*-R	5′-GGA TGA TGG TTG TAA ATT CTG GAG AG-3′		
AG*skdr*-F	5′-CTT CGA GTA TTT AAG TTG GCA AAA TC-3′	Initial PCR amplification	LCR assays
AG*skdr*-R	5′-TTC CAA ATA ACT GCA TAC CCA TAA C-3′		
P1-918_Met_G	5′-[M13F]CTT GGC CCA CAC TTA ATC TTT TAA TAT CAA TAA T**G**-3′	Probes	
P1-918_Ile_A	5′-[M13F-SP6]CTT GGC CCA CAC TTA ATC TTT TAA TAT CAA TAA T**A**-3′		
P2-918_Phos	5′-Phos-GGT CGA ACC ATT GGT GCT TTG GG-[M13Rrc]-3′		
M13_5p17nt-F	5′-gac tgc cgt acc aat tcC GCC AGG GTT TTC CCA GTC ACG AC-3′	PCR Reamplification	
M13_5p18nt-R	5′-cga tga gtc cct gag taa AGC GGA TAA CAA TTT CAC ACA GGA-3′		

M13F: 5′-cgc cag ggt ttt ccc agt cac gac-3′
SP6: 5′-cat acg att tag gtg aca gta tag-3′ (filler sequence to increase amplicon size)
M13Rrc: 5′-tcc tgt gtg aaa ttg tta tcc gct-3′

Table S2. Oligonucleotide primers used for internal "walking" across amplified *Aphis glycines* cDNAs corresponding voltage-gated sodium channel (*vgsc*) subunits h1 (gene model AG6007485-RA) and h2 (merger of AG6007488-RA and AG6007489-RA). Design and synthesis carried out at the Iowa State University DNA Facility (Ames, IA, USA), and names correspond to structural domains, DI and DII (12) or DIII and DIV (34), predicted within the cDNA amplicon. Initial amplification of cDNAs for *vgsc* subunit h1 using the primer pair AG6007485-F and -R, and *vgsc* subunit h2 using AG6007489-F and AG6007488-R is described in the main text.

Subunit h1 corresponding to gene model AG6007485-RA; **Domains I and II**

**Primer name**	**Primer sequence**
Ag_vgsc_12-F1	5′-CTC AGG GAT GTC ATT ATA T-3′
Ag_vgsc_12-R1	5′-TTT GCC AAC TTA AAT ACT C-3′
Ag_vgsc_12-F2	5′-GGA TCG CCG TTC AAC ATT C-3′
Ag_vgsc_12-R2	5′-AAC CGC ACT CGT CCA CTT T-3′
Ag_vgsc_12b-R2	5′-GGA CGT GTG TGA CGT GTA A-3′
Ag_vgsc_12-F3	5′-GGA TTT CAT ACT CAA CAA G-3′
Ag_vgsc_12-R3	5′-GGC TAA TAT CAA ATT GAC A-3′
Ag_vgsc_12b-R3	5′-CTT GTG TAT CCA TAG TTA G-3′
Ag_vgsc_12-F4	5′-TGG CCG ATT CAA TAA ATG G-3′
Ag_vgsc_12-R4	5′-GGG TGG ACT AAT ATG TAA A-3′

Subunit h2 corresponding to gene models AG6007488-RA and AG6007489-RA; **Domains III and IV**

**Primer name**	**Primer sequence 5'-3'**
Ag_vgsc_34-F1	5′-AGT GGT TGG CAT TGG GTT T-3′
Ag_vgsc_34b-F1	5′-GCT GTG ATT ACT ATG ATT T-3′
Ag_vgsc_34-R1	5′-CGA AAG TGC GGA AAT TGT A-3′
Ag_vgsc_34-F2	5′-CAA GAG ACA CGC ACG GGA A-3′
Ag_vgsc_34b-F2	5′-GCT TAA AGC AAT TCC AAG A-3′
Ag_vgsc_34c-F2	5′-GGT GCT TCT CTT GAA ATG T-3′
Ag_vgsc_34b-R2	5′-GAT TTC ACG AAT TGG CTG T-3′
Ag_vgsc_34-R2	5′-CGC CAC CTT GGT CTT GGA A-3′
Ag_vgsc_34-F3	5′-AGG AAG GTC TTA CGG ACG A-3′
Ag_vgsc_34b-F3	5′-GCT ATA TTT GGC ATG TCG T-3′
Ag_vgsc_34-R3	5′-GGG ATA TTC TTC TTC CTC A-3′
Ag_vgsc_34b-R3	5′-CGC CAG CCA GGA ATG GAA A-3′

Table S3. Locations of *Aphis glycines* collections, phenotypic assays conducted, and assay methods used to detect voltage gated sodium channel (*vgsc*) mutations (1014RFLP = *Bst*EII PCR-RFLP differentiating L1014F mutations; 918LCR = ligase chain reaction differentiating M918I mutations.

**Location**	**Origin**	**Latitude**	**Longitude**	**Before spray collection**	**Glass vial assay**	**Leaf-dip bioassay**	**Field spray**	**After spray collection**	**Detection of *vgsc* mutations**
Boone, IA	Field	42.007940	-93.789638	2-Aug-19	2-Aug-19	n/a	9-Aug-19	12-Aug-19	1014RFLP/918LCR
Sutherland, IA	Field	42.927862	-95.538465	15-Aug-19	16-Aug-19	n/a	16-Aug-19	20-Aug-19	1014RFLP/918LCR
Kanawha, IA	Field	42.915374	-93.791427	22-Aug-19	28-Aug-19	n/a	28-Aug-19	30-Aug-19	1014RFLP/918LCR
Darwin, MN	Field	45.037493	-94.450962	11-Aug-19	16-Aug-19	n/a	n/a	n/a	1014RFLP/918LCR
Nashua, IA	Field	42.939608	-92.569824	27-Jul-20	31-Jul-20	n/a	27-Jul-20	30-Jul-20	1014RFLP/918LCR
Sutherland, IA	Field	42.929873	-95.543407	10-Aug-20	11-Aug-20	n/a	11-Aug-20	12-Aug-20	1014RFLP/918LCR
SBA-ISU-B1	Laboratory	n/a	n/a	n/a	n/a	This study	n/a	n/a	1014RFLP/918LCR<br>cDNA sequencing<br>Sanger sequencing
SBA-ISU-B3	Laboratory	n/a	n/a	n/a	n/a	This study	n/a	n/a	1014RFLP/918LCR<br>cDNA sequencing<br>Sanger sequencing
SBA-ISU-B4	Laboratory	n/a	n/a	n/a	n/a	Valmorbida et al., 2021	n/a	n/a	1014RFLP/918LCR<br>cDNA sequencing<br>Sanger sequencing
Biotype 1 UIL USDA-ARS	Laboratory	n/a	n/a	n/a	Paula et al., 2021	n/a	n/a	n/a	Sanger sequencing
SBA-MN1-2017	Field	n/a	n/a	n/a	n/a	This study	2017	n/a	1014RFLP/918LCR<br>cDNA sequencing<br>Sanger sequencing
SBA-MN2-2017	Field	n/a	n/a	n/a	n/a	This study	2017	n/a	1014RFLP/918LCR<br>cDNA sequencing
SBA-Nashua-2018	Field	42.943008	-92.569733		n/a	This study	2018	n/a	1014RFLP/918LCR<br>cDNA sequencing<br>Sanger sequencing
SBA-Sutherland-2017	Field	42.927913	-95.538668		n/a	This study	2017	n/a	1014RFLP/918LCR<br>cDNA sequencing<br>Sanger sequencing

Table S4. Effect of synergists on the toxicity of lambda-cyhalothrin on susceptible and resistant *Aphis glycines* in leaf-dip bioassays.

Treatment	Susceptible SBA-ISU-B1				Resistant SBA-MN1-2017				
	LC_{50}	95% CI	Slope	SR[a]	LC_{50}	95% CI	Slope	SR[a]	RR[b]
Lambda–cyhalothrin	0.38	0.29-0.46	2.51 ± 0.45	-	18.33	13.92-22.74	2.08 ± 0.40	-	48.23
Lambda–cyhalothrin + PBO[c]	0.29	0.23-0.35	1.97 ± 0.26	1.31	14.38	9.53-19.22	1.56 ± 0.31	1.27	49.58
Lambda–cyhalothrin + TPP[d]	0.30	0.19-0.41	1.37 ± 0.19	1.26	14.25	10.28-18.23	1.03 ± 0.11	1.28	47.50
Lambda–cyhalothrin + DEF[e]	0.29	0.19-0.39	1.56 ± 0.23	1.31	14.02	9.43-18.61	1.77 ± 0.39	1.30	48.34

[a] Synergistic ratio: LC_{50} of the insecticide alone divided by the LC_{50} of the insecticide + synergist.

[b] Resistance ratio (RR): LC_{50} of resistant SBA-MN1-2017 divided by LC_{50} of susceptible SBA-ISU-B1.

[c] Piperonyl butoxide: cytochrome P450 inhibitor

[d] Triphenyl phosphate: carboxylesterase inhibitor

[e] *S,S,S*-tributyl phosphorotrithioate: esterase inhibitor

Table S5. Frequencies of predicted amino acid changes among *Aphis glycines* collected pre- and post-application of a foliar pyrethroid insecticide spray. Reported as absolute number (proportion) of *A. glycines* individuals predicted to give rise to amino acid mutations L1014F and M918I based on *Bst*EII PCR-RFLP and LCR assays, respectively. Fisher Exact test *P*-values showing significant differences between pre- and post-application genotypes are indicated with an asterisk (*). Data for pre- and post-application *super-kdr* heterozygote genotypes (L/F:M/I) across locations are highlighted grey in both 2019 and 2020.

**2019**											
**Location**	**Collection time**	**Genotype (L1014F:M918I)**									
		**L/L:M/M**	**L/L:M/I**	**L/L:I/I**	**L/F:M/M**	**L/F:M/I**	**L/F:I/I**	**F/F:M/M**	**F/F:M/I**	**F/F:I/I**	***P*-value**
Boone-IA	Pre	33 (27.5)	18 (15)	0 (0.0)	59 (49.2)	10 (8.3)	0 (0.0)	0 (0.0)	0 (0.0)	0 (0.0)	0.0397*
	Post	1 (2.1)	3 (6.4)	0 (0.0)	22 (46.8)	13 (27.7)	0 (0.0)	8 (17.0)	0 (0.0)	0 (0.0)	
Kanawha-IA	Pre	0 (0.0)	0 (0.0)	0 (0.0)	95 (79.2)	7 (5.8)	0 (0.0)	18 (15.0)	0 (0.0)	0 (0.0)	0.0476*
	Post	1 (1.0)	0 (0.0)	0 (0.0)	47 (45.6)	32 (31.1)	0 (0.0)	23 (22.3)	0 (0.0)	0 (0.0)	
Sutherland-IA	Pre	20 (17.4)	5 (4.3)	0 (0.0)	61 (53.0)	29 (25.2)	0 (0.0)	0 (0.0)	0 (0.0)	0 (0.0)	0.1667
	Post	0 (0.0)	0 (0.0)	0 (0.0)	66 (69.5)	29 (30.5)	0 (0.0)	0 (0.0)	0 (0.0)	0 (0.0)	
All locations	Pre	53 (14.9)	23 (6.5)	0 (0.0)	215 (60.6)	46 (13.0)	0 (0.0)	18 (5.1)	0 (0.0)	0 (0.0)	0.0079*
	Post	2 (0.8)	3 (1.2)	0 (0.0)	135 (55.1)	74 (30.2)	0 (0.0)	31 (12.7)	0 (0.0)	0 (0.0)	
**2020**											
**Location**	**Collection time**	**Genotype (L1014F:M918I)**									
		**L/L:M/M**	**L/L:M/I**	**L/L:I/I**	**L/F:M/M**	**L/F:M/I**	**L/F:I/I**	**F/F:M/M**	**F/F:M/I**	**F/F:I/I**	***P*-value**
Nashua-IA	Pre	59 (53.6)	2 (1.8)	0 (0.0)	37 (33.6)	10 (9.1)	0 (0.0)	2 (1.8)	0 (0.0)	0 (0.0)	0.3333
	Post	0 (0.0)	0 (0.0)	0 (0.0)	0 (0.0)	38 (100)	0 (0.0)	0 (0.0)	0 (0.0)	0 (0.0)	
Sutherland-IA	Pre	3 (2.7)	11 (10.0)	0 (0.0)	6 (5.5)	4 (3.6)	0 (0.0)	86 (78.2)	0 (0.0)	0 (0.0)	0.0397*
	Post	1 (1.1)	0 (0.0)	0 (0.0)	30 (31.6)	10 (10.5)	0 (0.0)	54 (56.8)	0 (0.0)	0 (0.0)	
All locations	Pre	62 (28.2)	13 (5.9)	0 (0.0)	43 (19.5)	14 (6.4)	0 (0.0)	88 (40.0)	0 (0.0)	0 (0.0)	0.0397*
	Post	1 (0.8)	0 (0.0)	0 (0.0)	30 (22.6)	48 (36.1)	0 (0.0)	54 (40.6)	0 (0.0)	0 (0.0)	
